# Supplementary material for: Biomimetic catalytic transformation of toxic α-oxoaldehydes to high-value chiral α-hydroxythioesters using artificial glyoxalase I
Source: Nat Commun. 2017 Apr 4;8:14877. doi: 10.1038/ncomms14877 (PMC5382281; doi:10.1038/ncomms14877)
Supplement: Supplementary Information — Supplementary figures, supplementary tables, supplementary methods and supplementary references. [file ncomms14877-s1.pdf]

Supplementary Figures

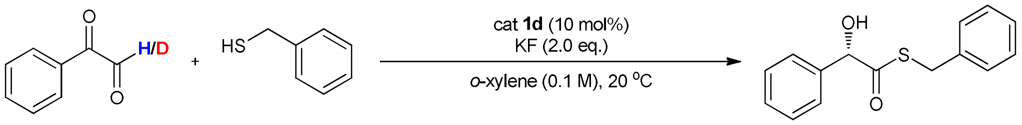

time [h]		0	6	12	24	36	48
with **2a**	Conv. of **5a** [%]	0	46.2	67.6	80.6	91.1	>99
with **2a-*d*$_1$**	Conv. of **5a** [%]	0	15.9	26.5	43.1	57.5	68.9

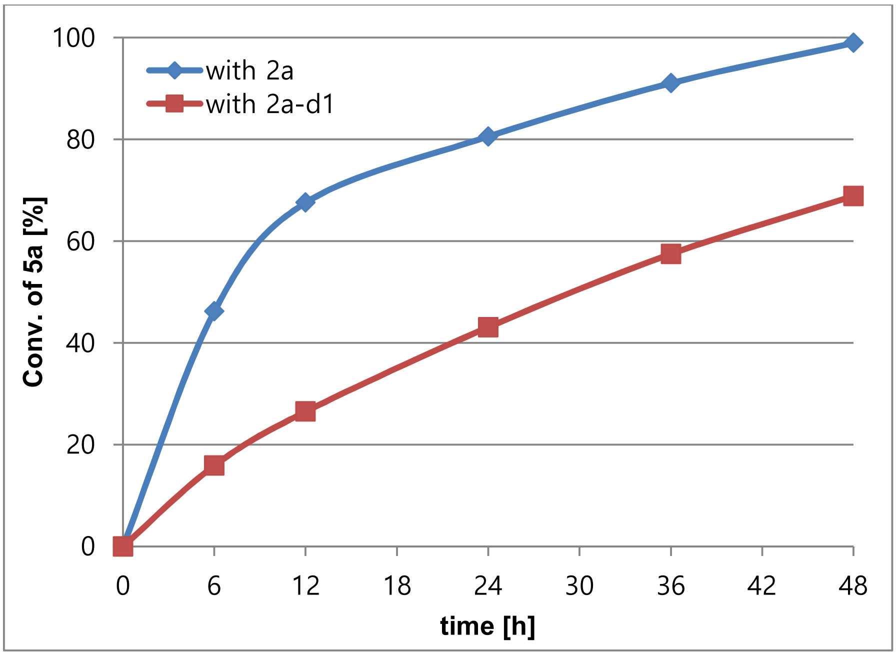

Supplementary Figure 1. Study of the rate determining step for isomerization of hemithioacetals.

Comments: Supplementary Fig. 1 shows the primary isotope effect on the reaction kinetics, suggesting that the deprotonation of the α-proton of hemithioacetal is the rate determining step.

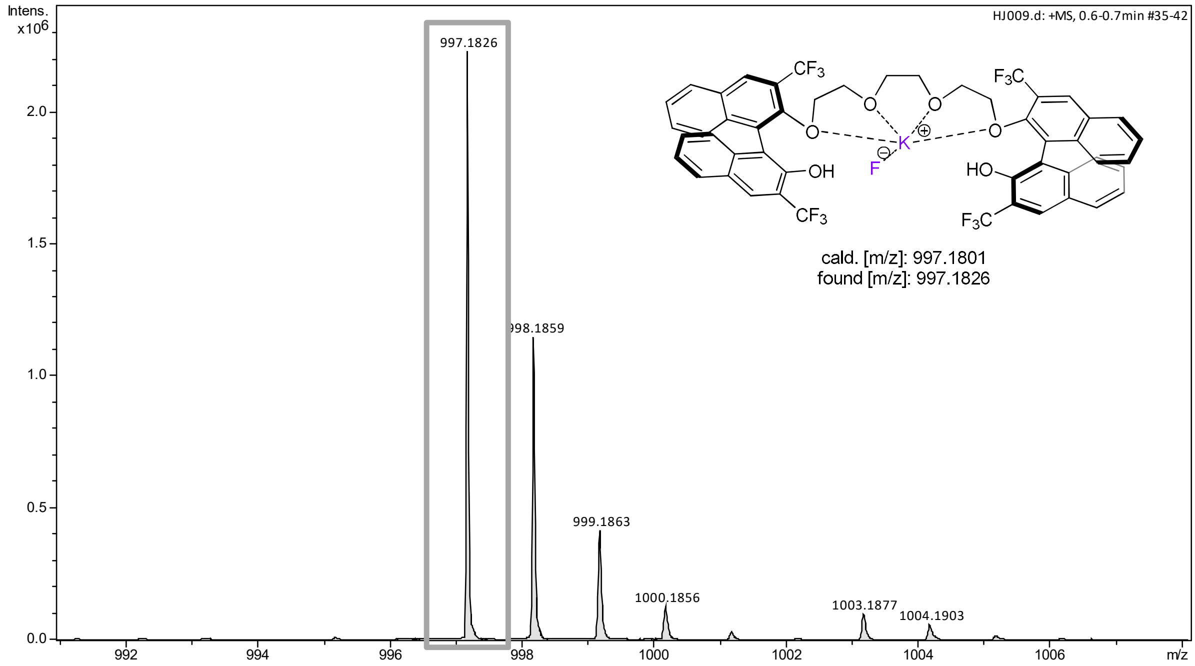

Supplementary Figure 2. ESI-HRMS spectrum of the mixture of catalyst (*R*)-1d and potassium fluoride.

Comments: Direct evidence of the complex formation of catalyst (*R*)-**1d** with potassium fluoride was obtained by ESI-HRMS (positive ion mode).

Supplementary Figure 3. ^{1}H and ^{13}C NMR spectra of compound **5a**.

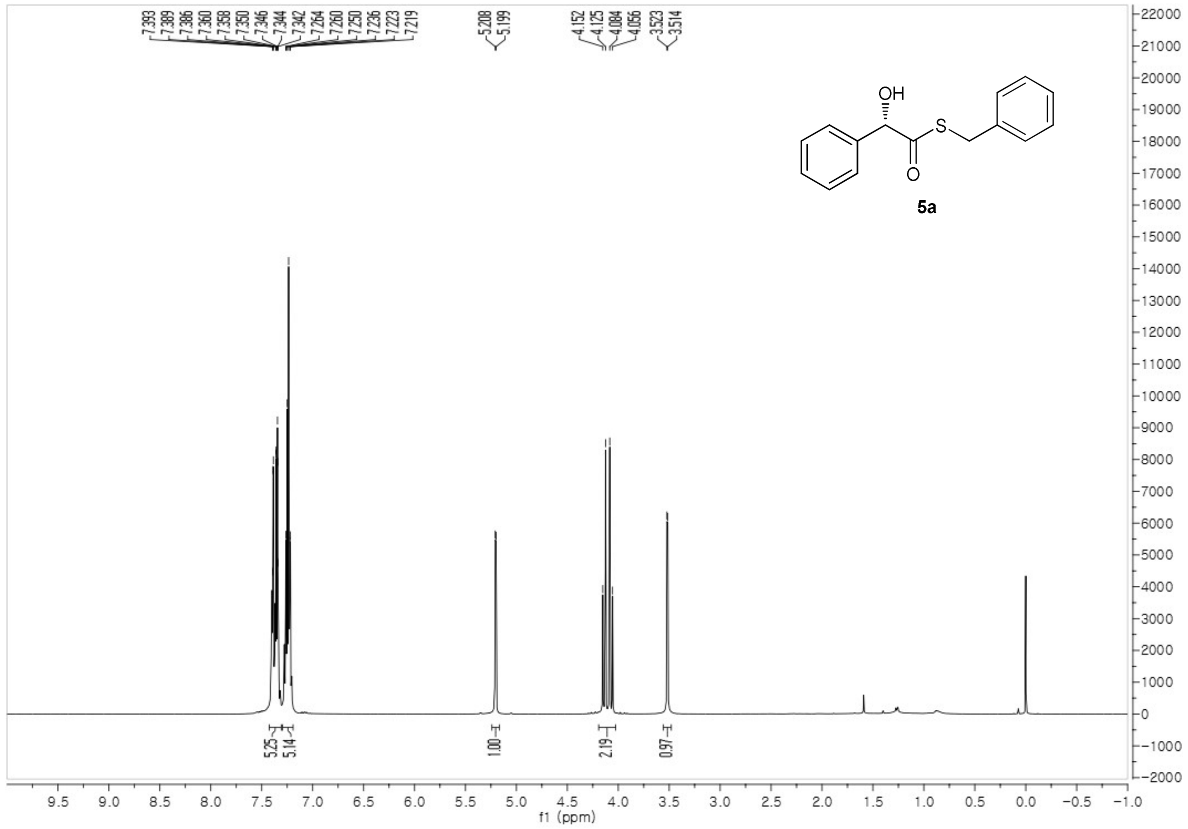

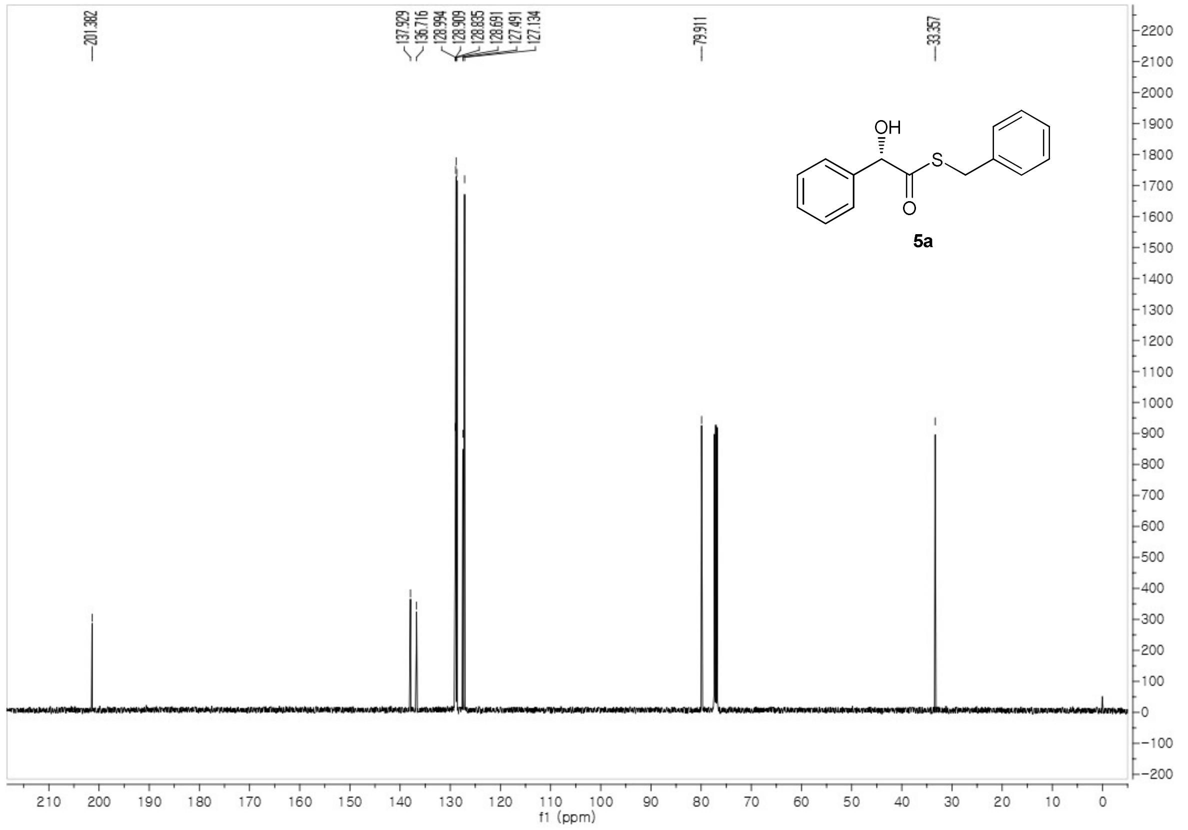

Supplementary Figure 4. ^{1}H and ^{13}C NMR spectra of compound **5ab**.

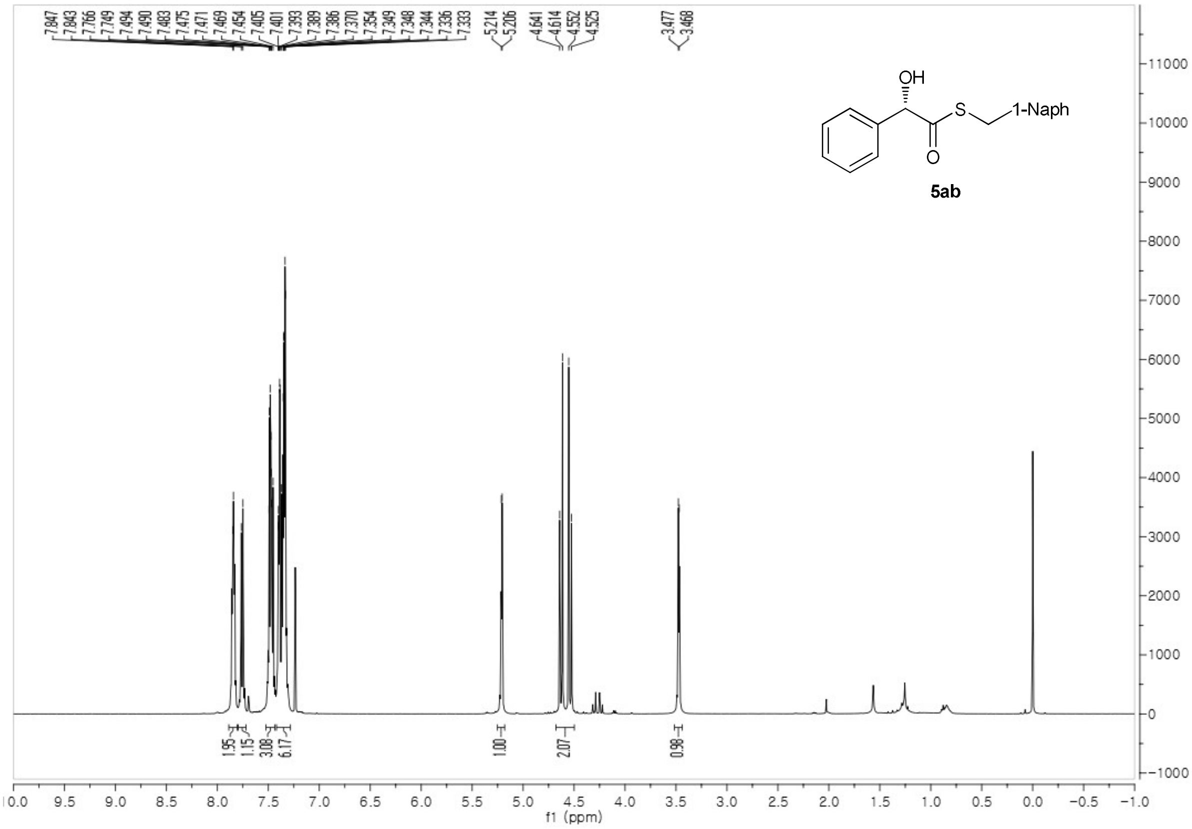

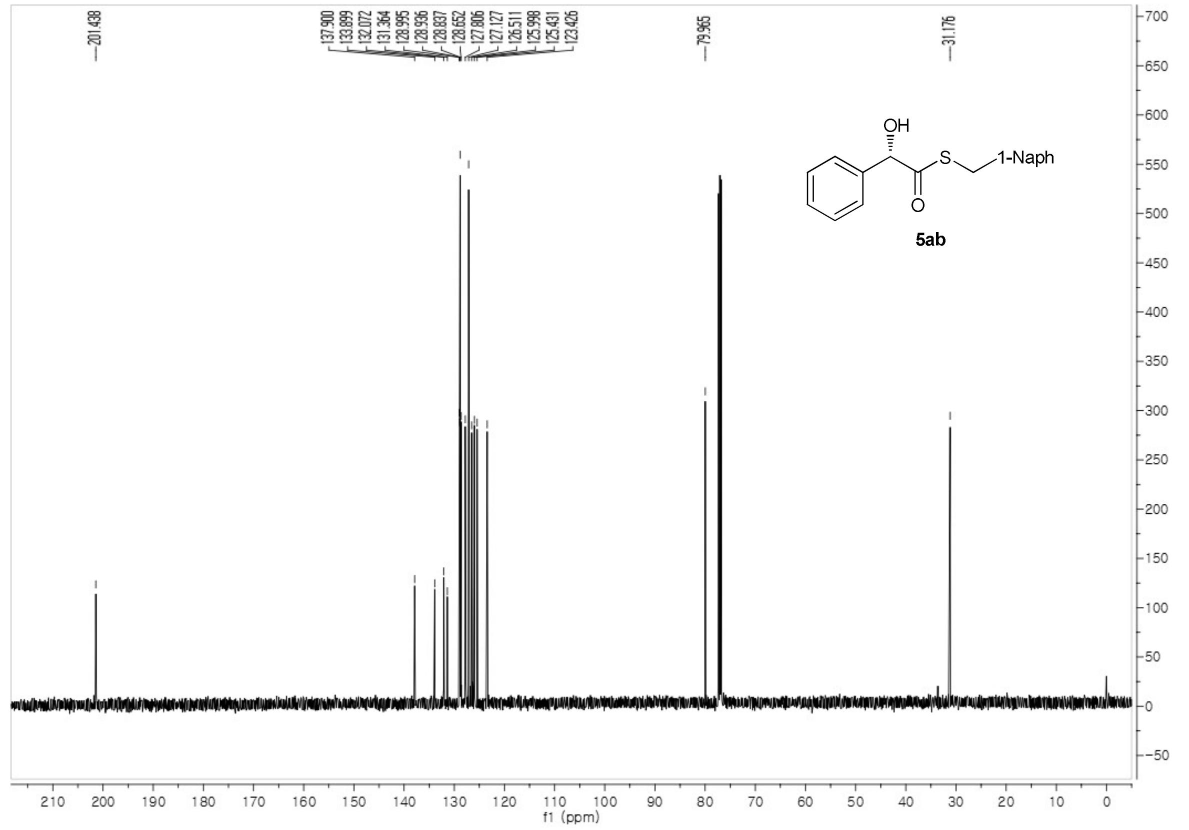

Supplementary Figure 5. ^{1}H and ^{13}C NMR spectra of compound **5ac**.

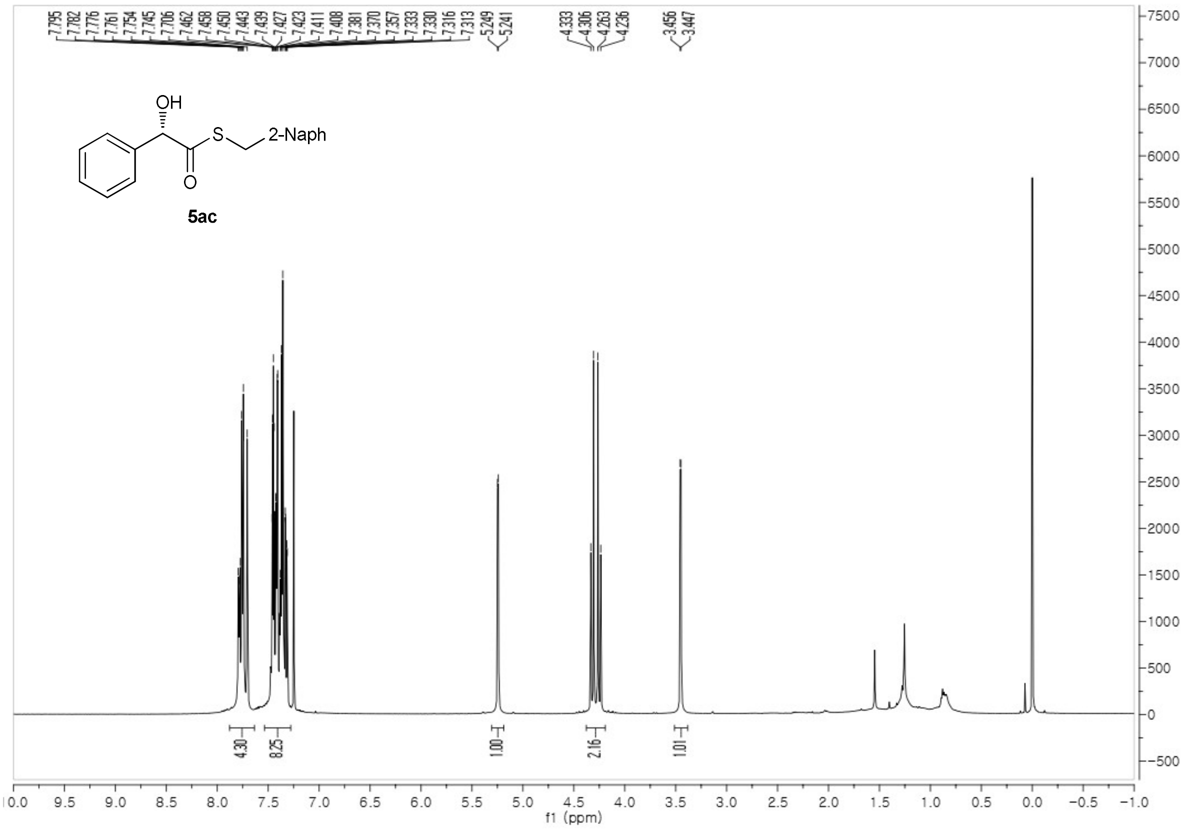

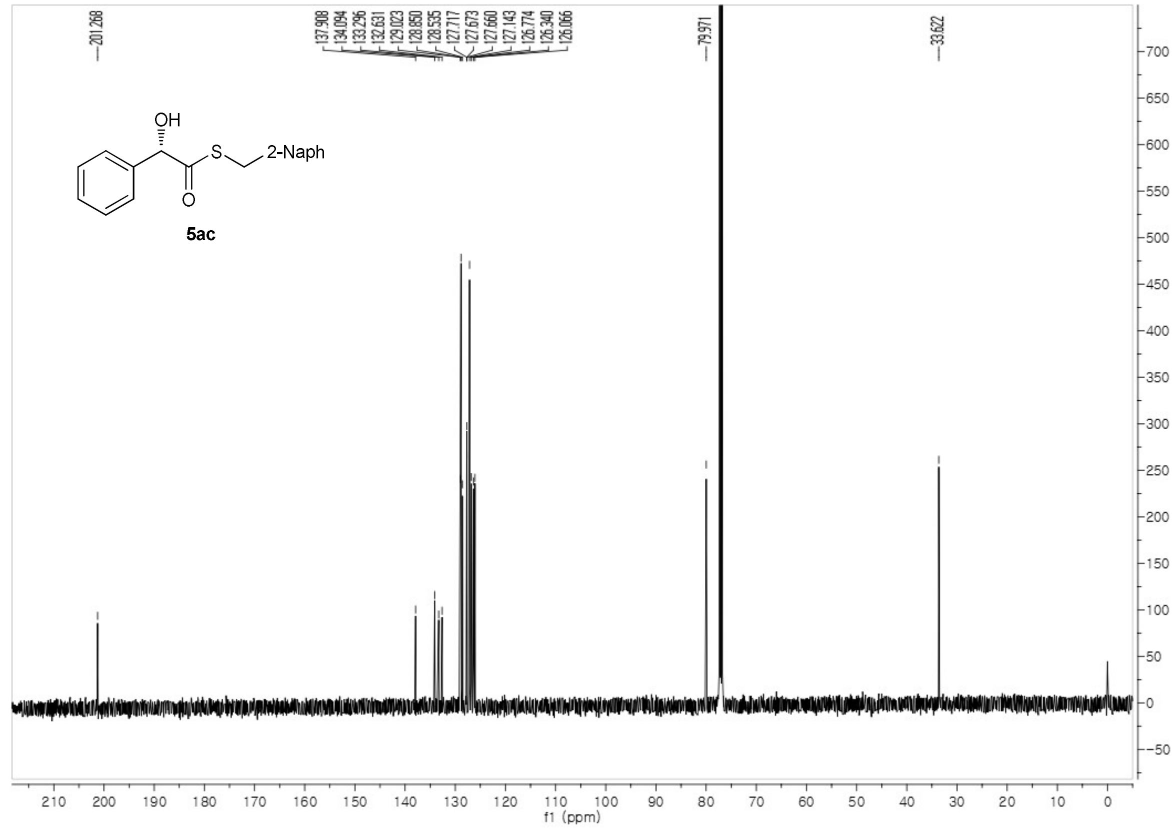

Supplementary Figure 6. ^{1}H and ^{13}C NMR spectra of compound **5ad**.

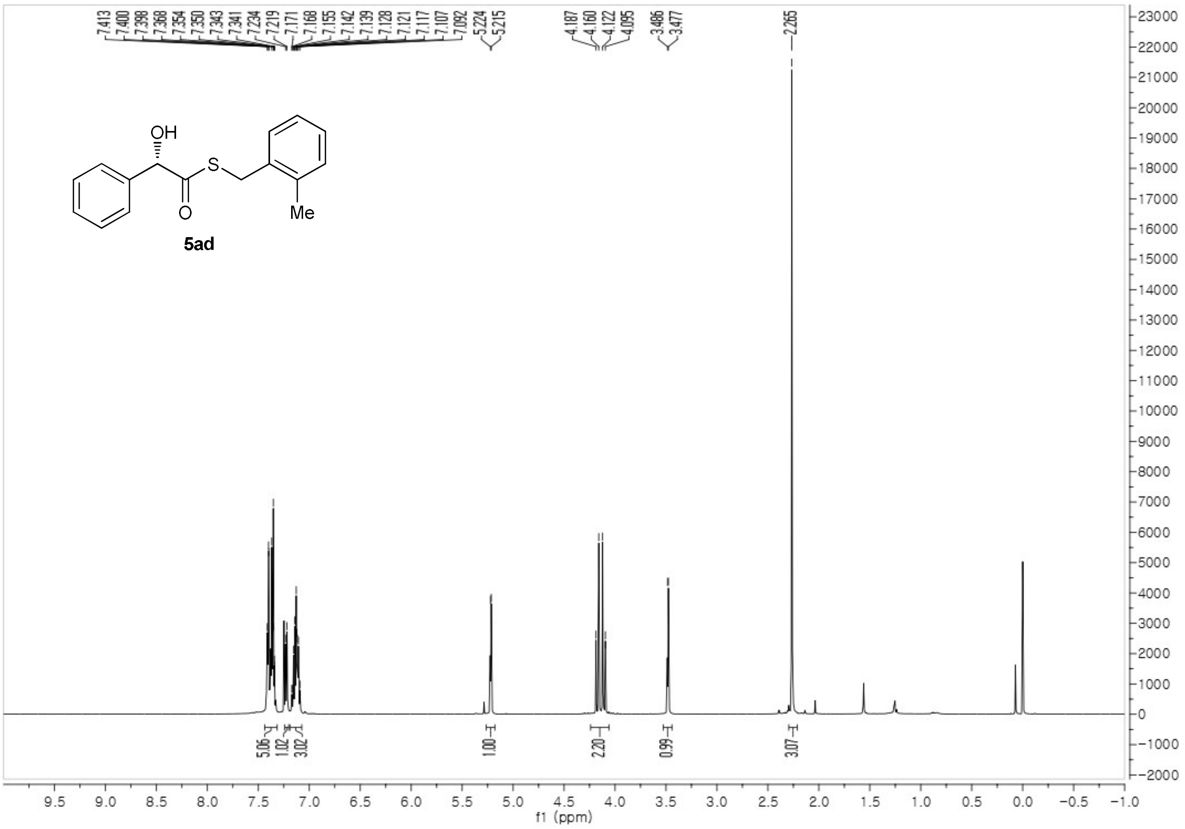

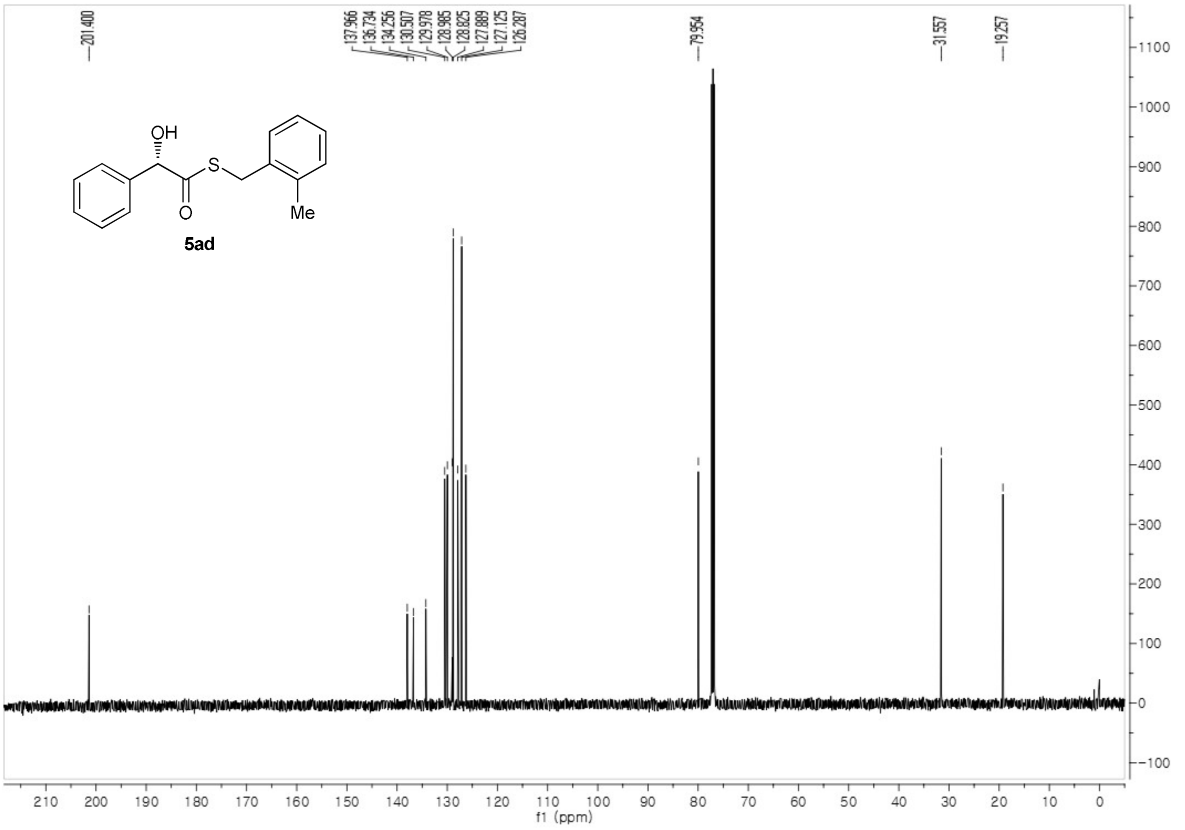

Supplementary Figure 7. ^{1}H and ^{13}C NMR spectra of compound **5ae**.

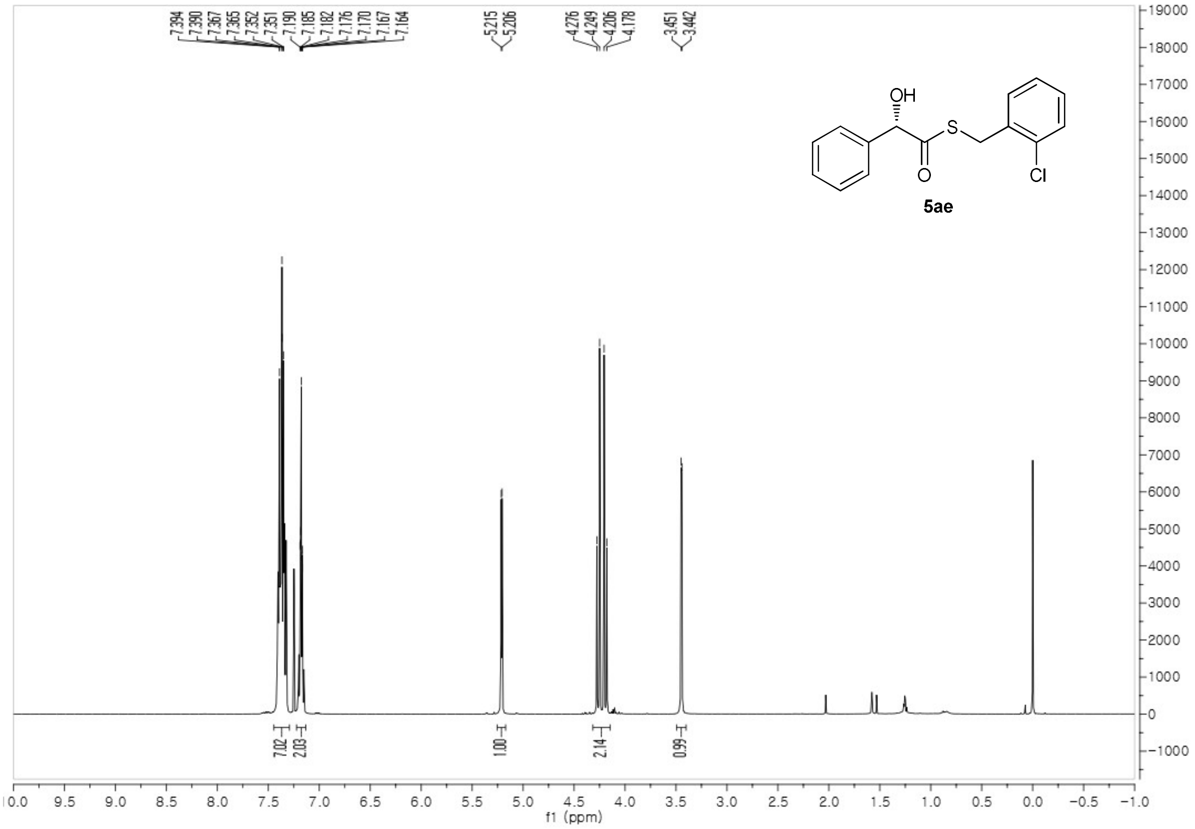

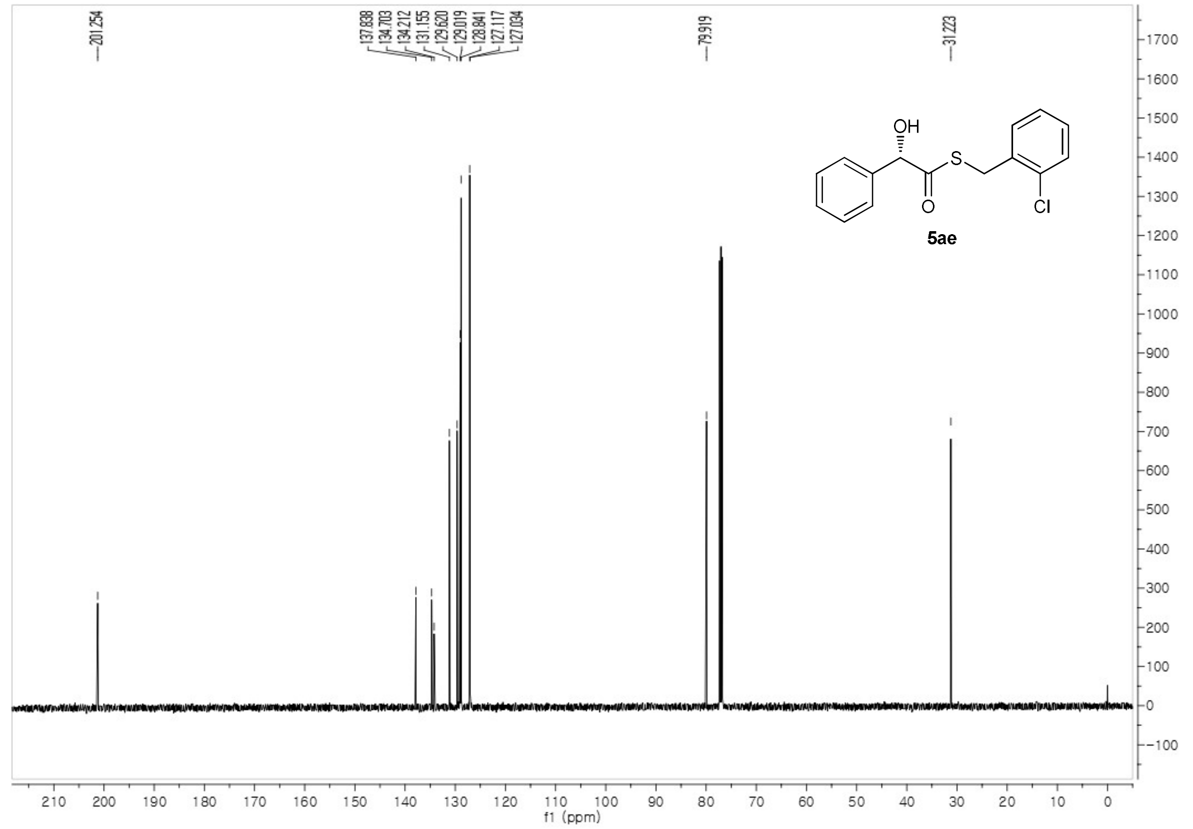

Supplementary Figure 8. ^{1}H and ^{13}C NMR spectra of compound **5af**.

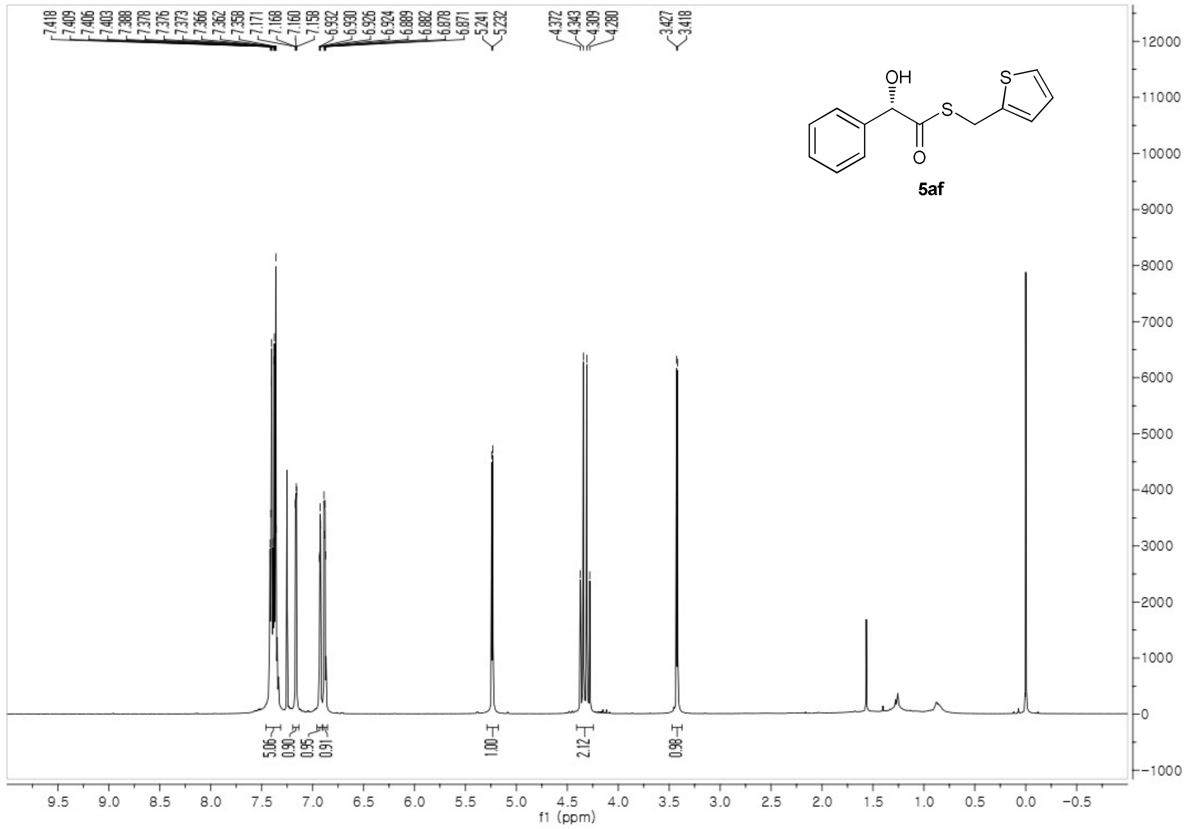

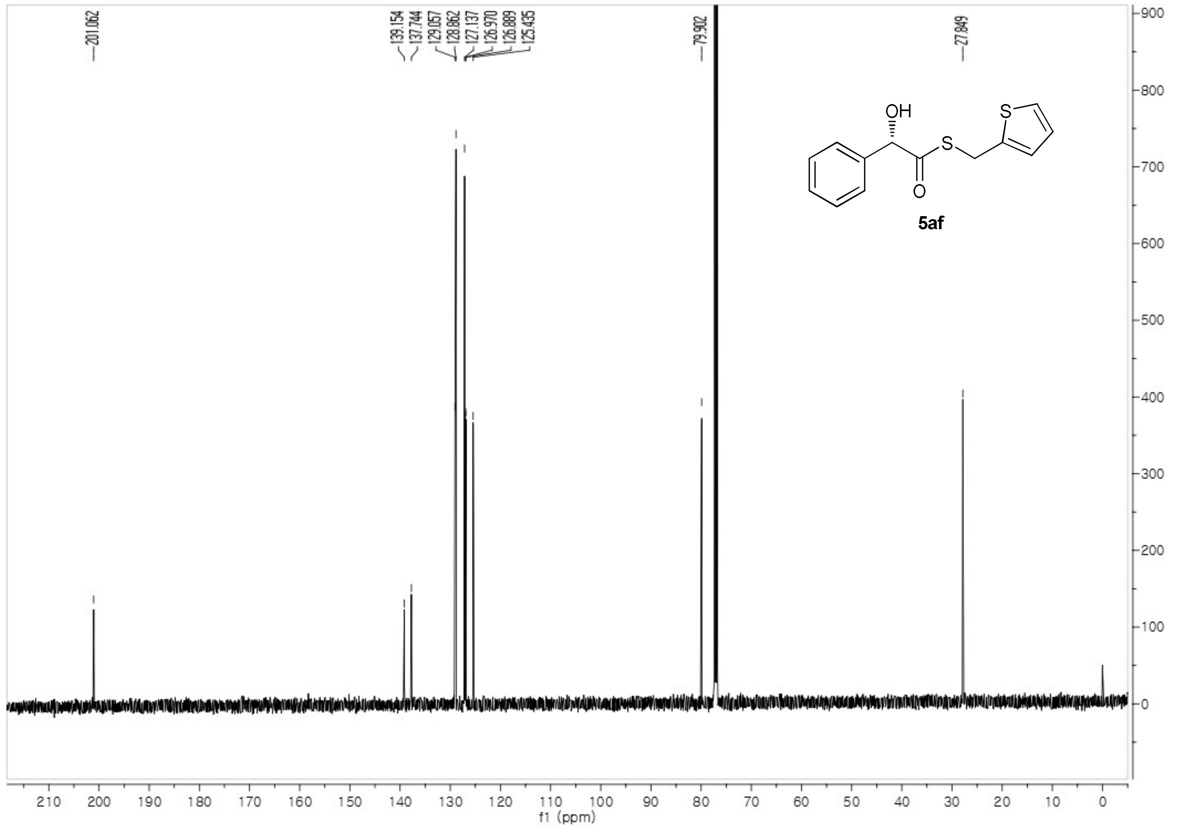

Supplementary Figure 9. ^{1}H and ^{13}C NMR spectra of compound **5ag**.

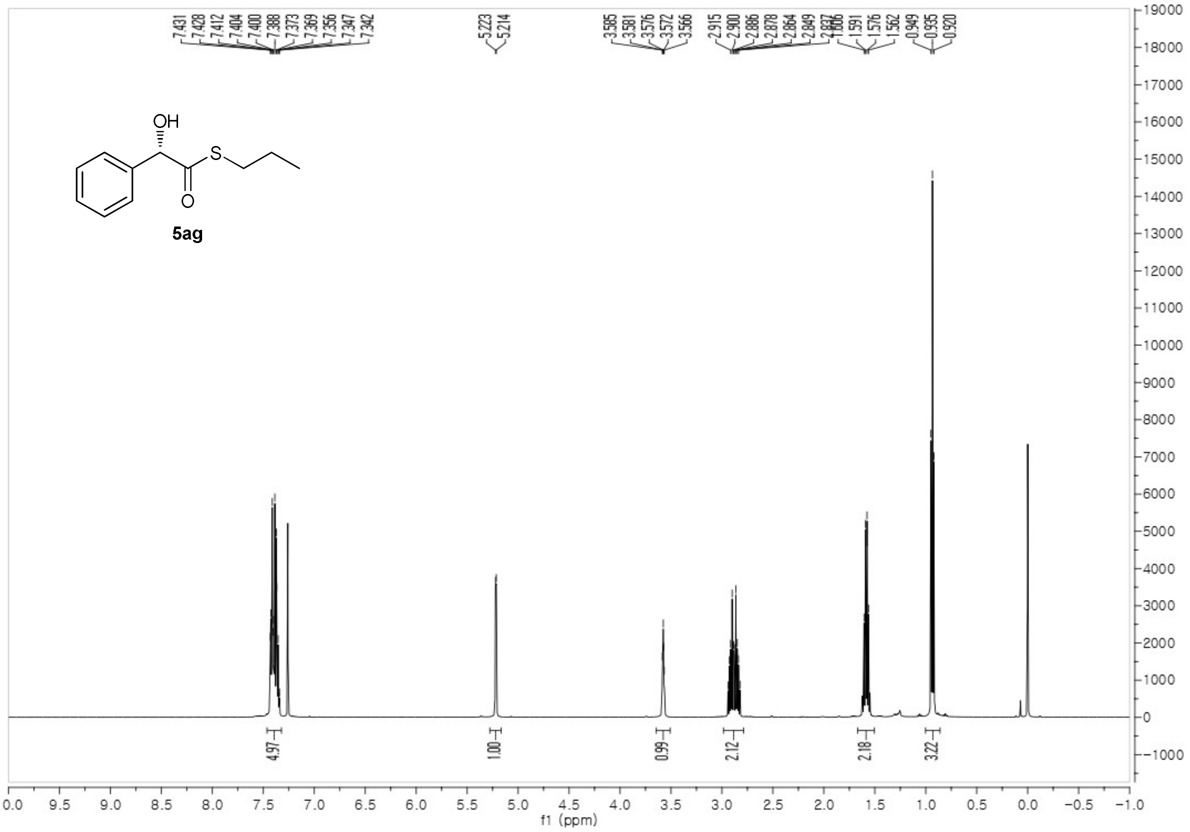

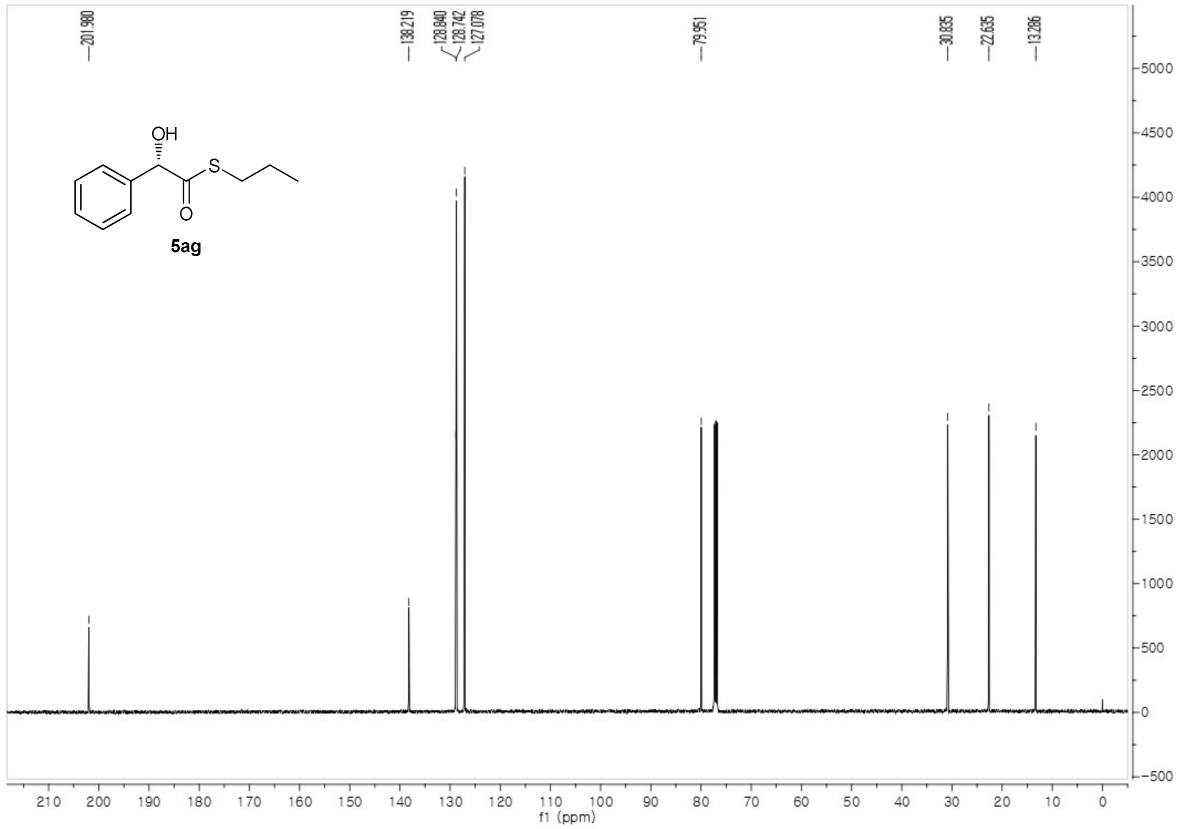

Supplementary Figure 10. ^{1}H and ^{13}C NMR spectra of compound **5ah**.

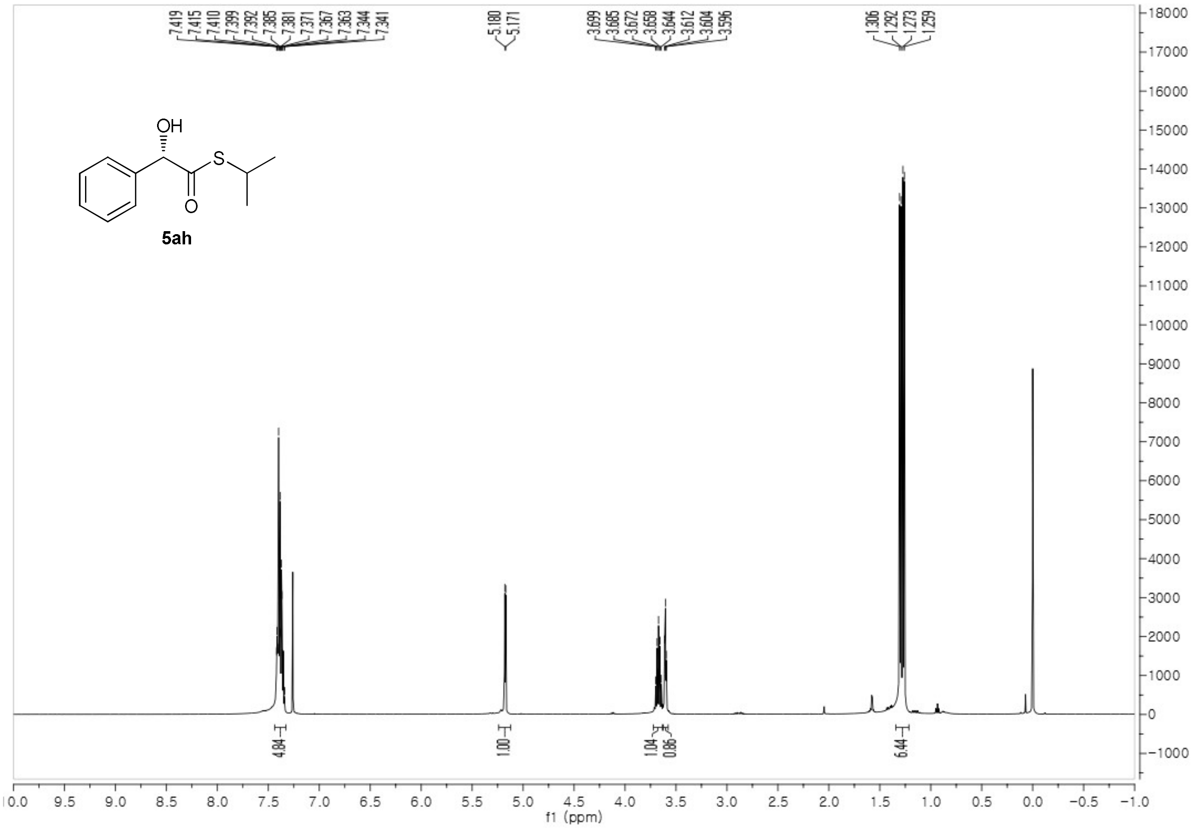

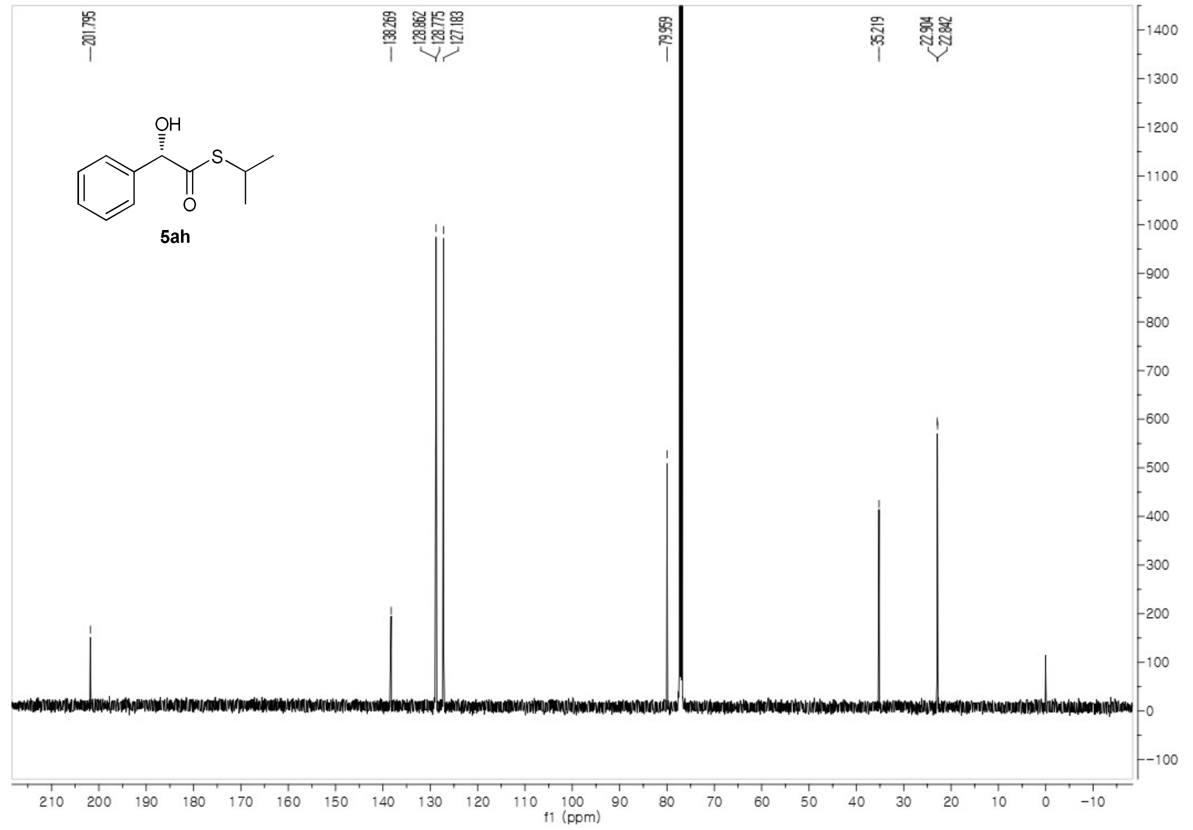

Supplementary Figure 11. ^{1}H and ^{13}C NMR spectra of compound **5ai**.

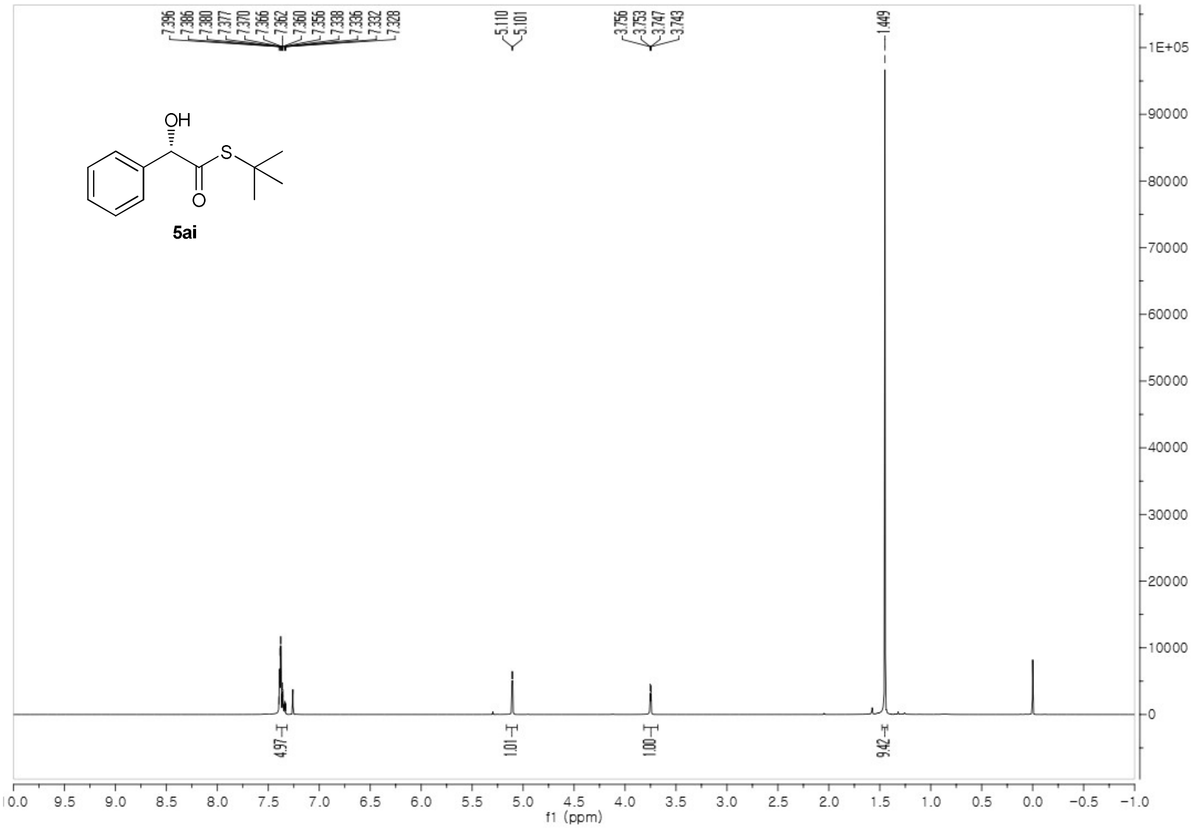

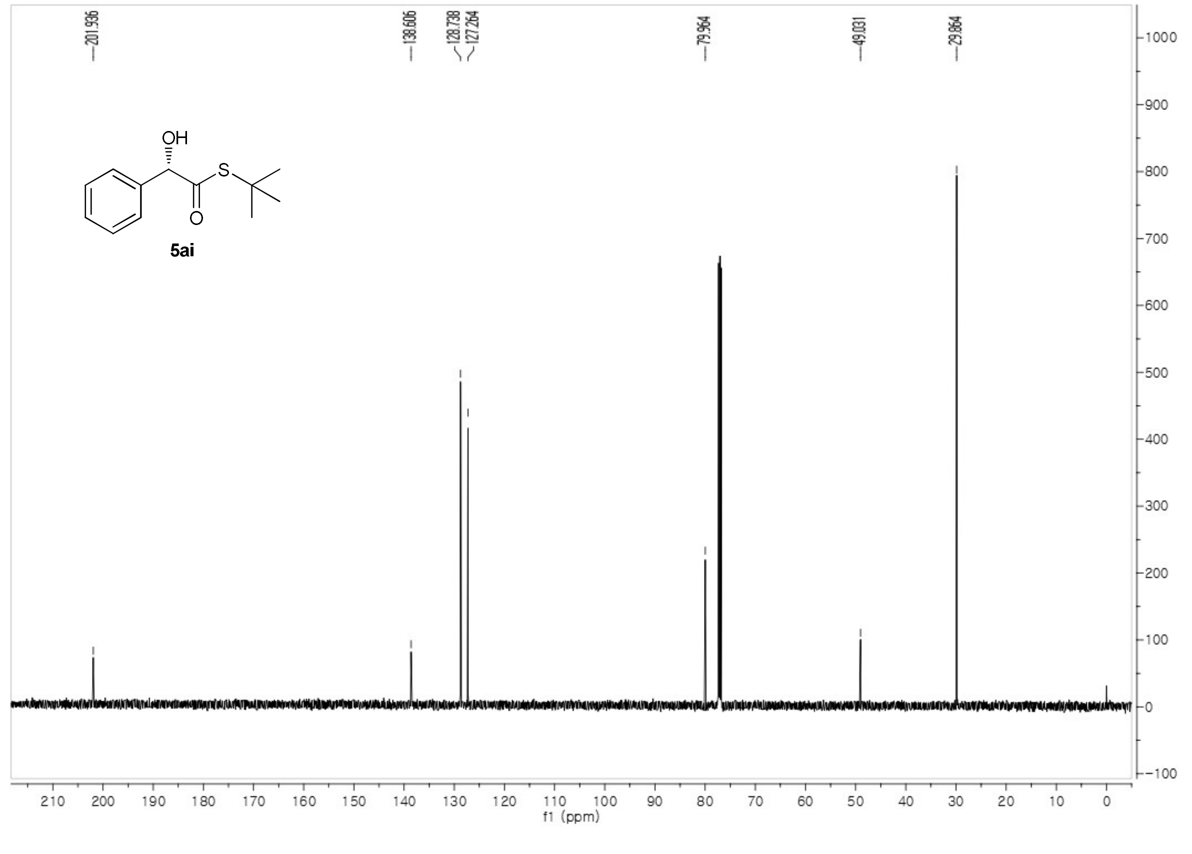

Supplementary Figure 12. ^{1}H and ^{13}C NMR spectra of compound **5aj**.

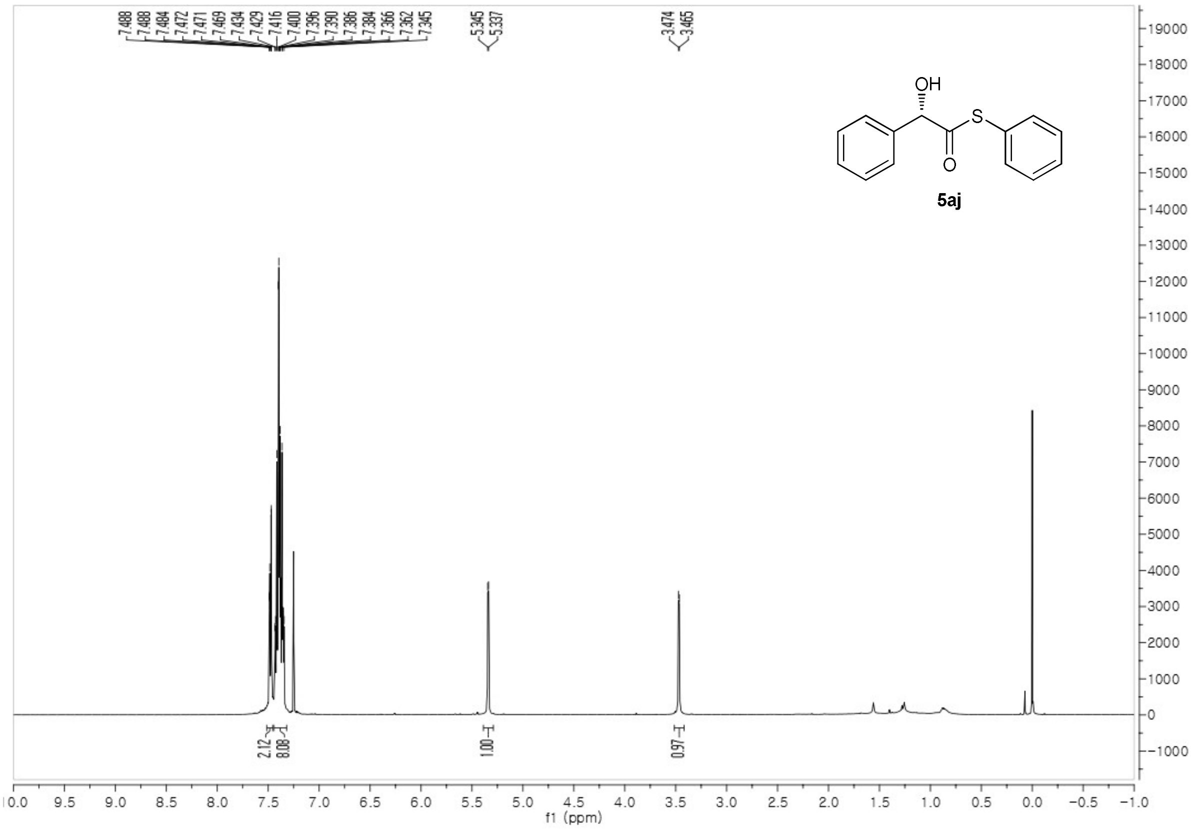

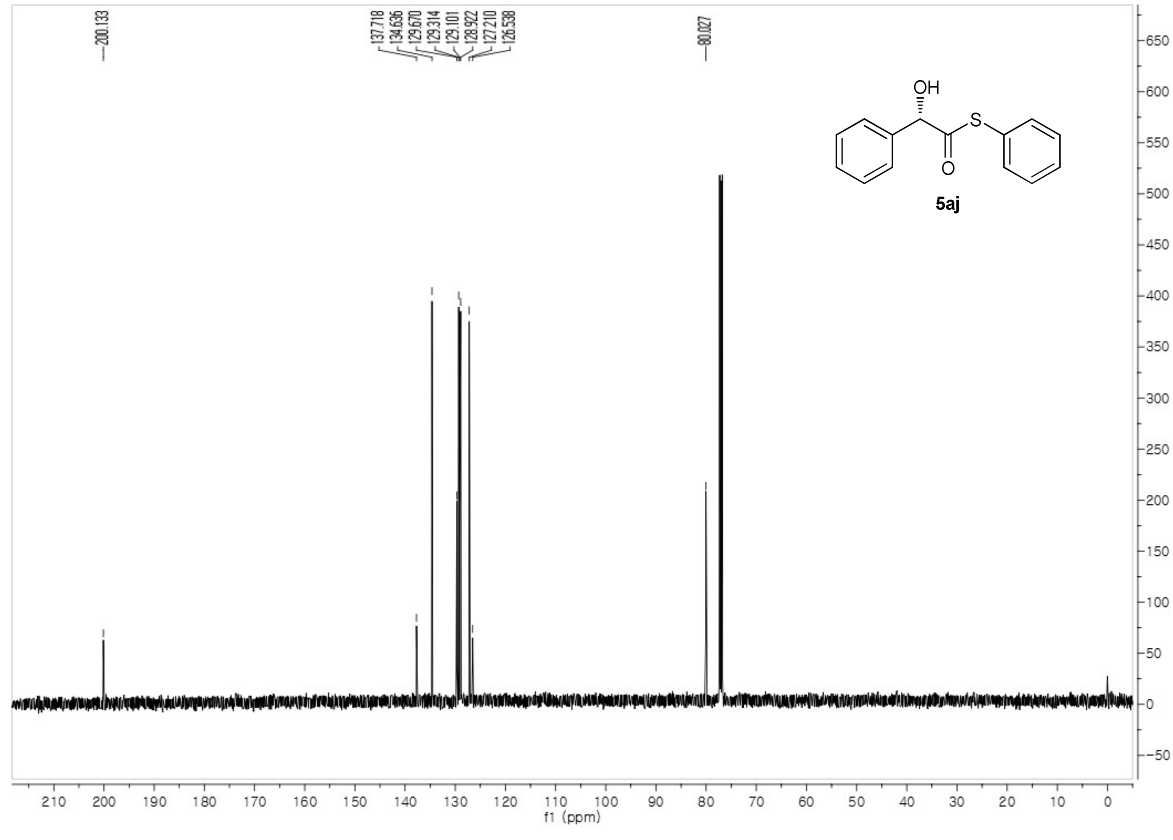

Supplementary Figure 13. ^{1}H and ^{13}C NMR spectra of compound **5ak**.

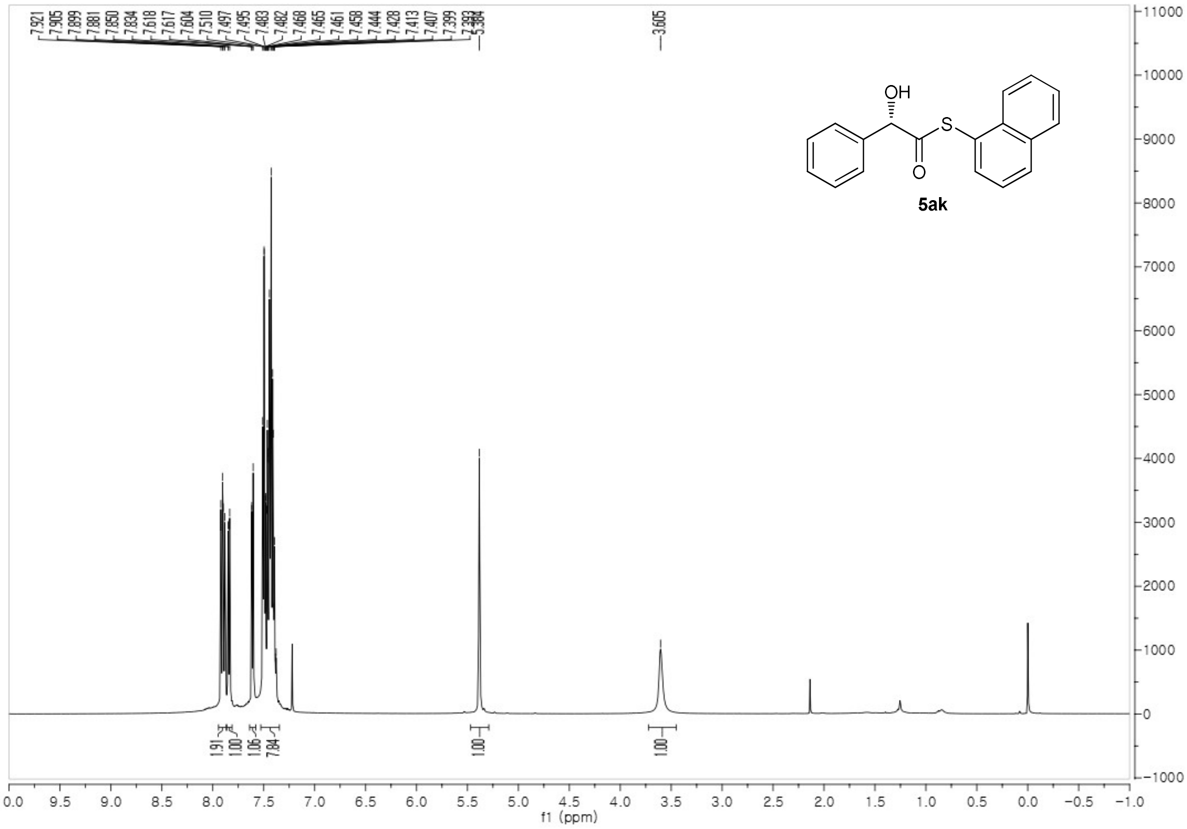

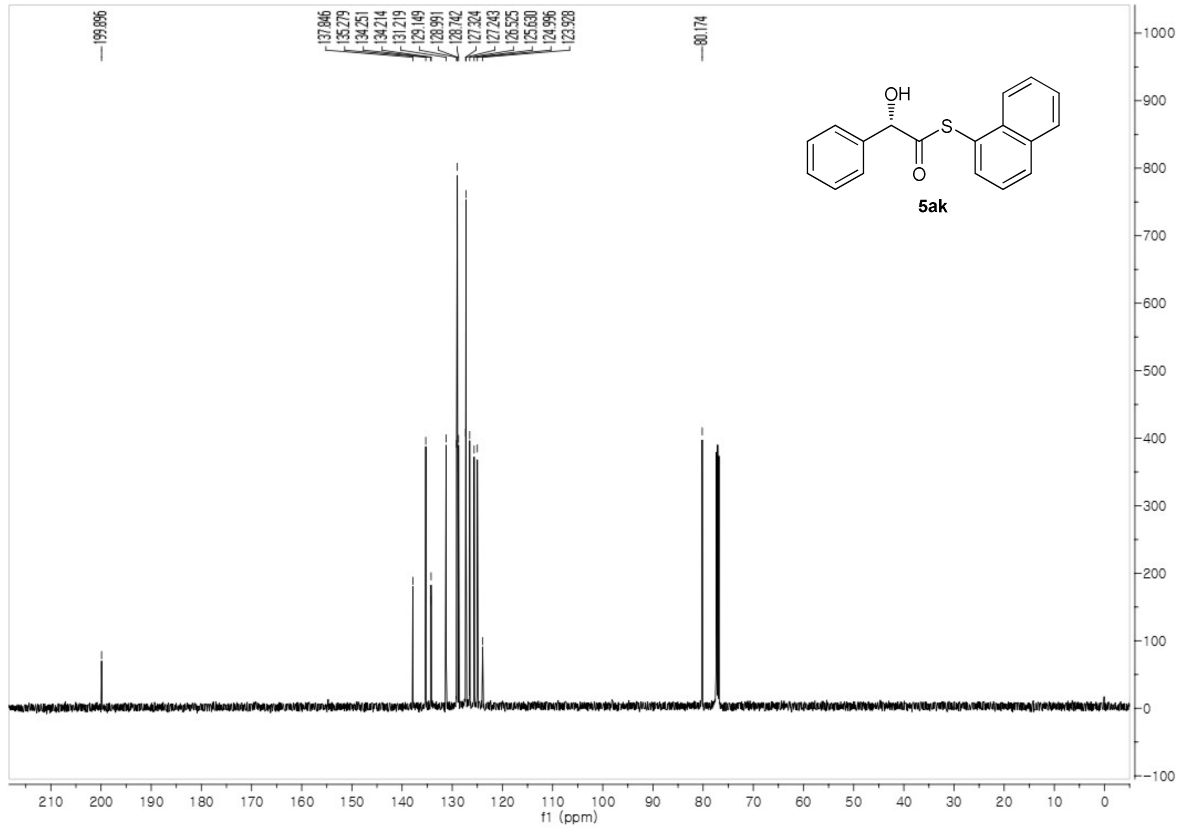

Supplementary Figure 14. ^{1}H and ^{13}C NMR spectra of compound **5al**.

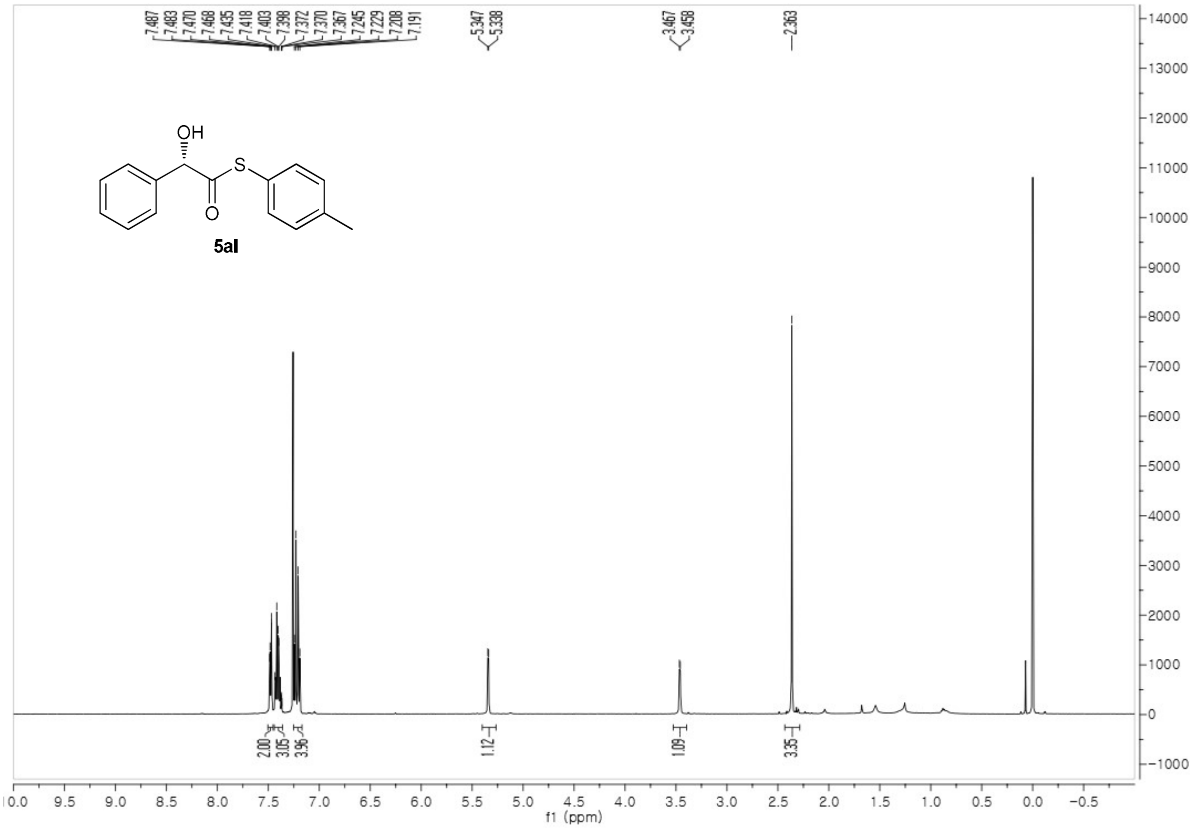

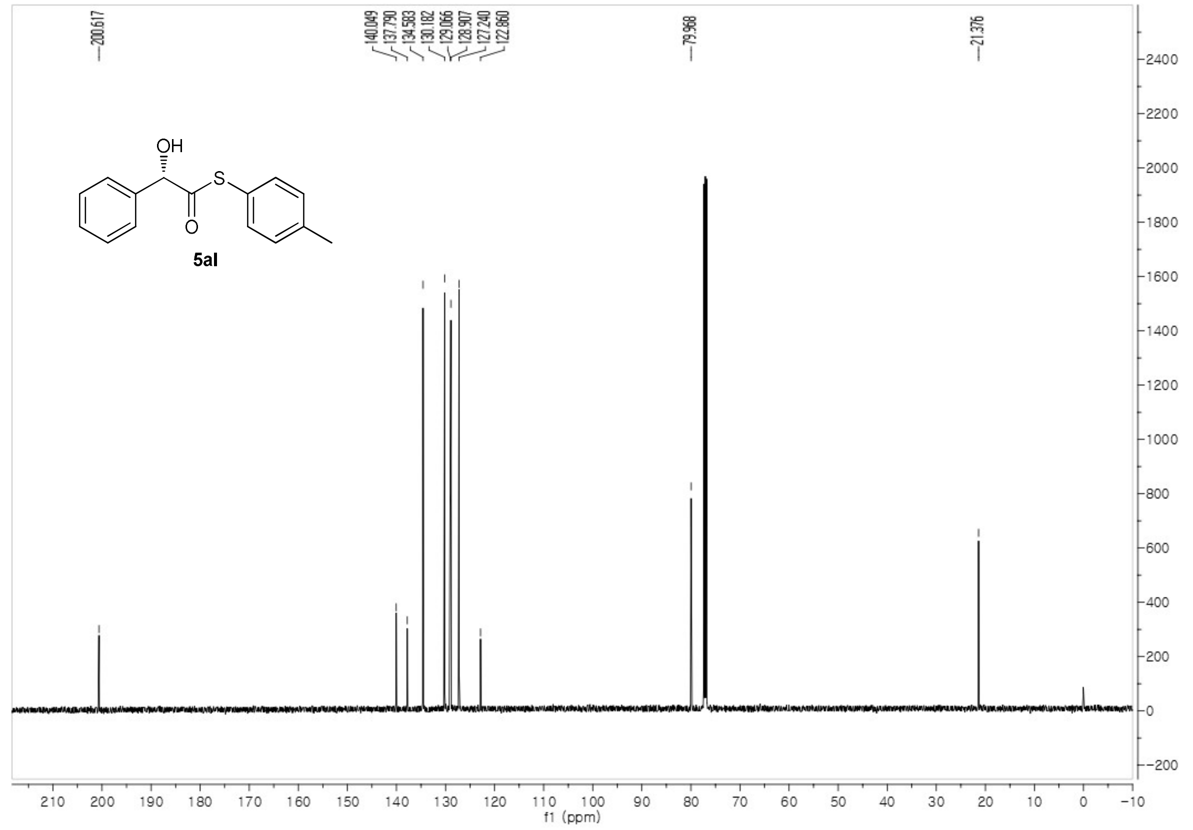

Supplementary Figure 15. ^{1}H and ^{13}C NMR spectra of compound **5am**.

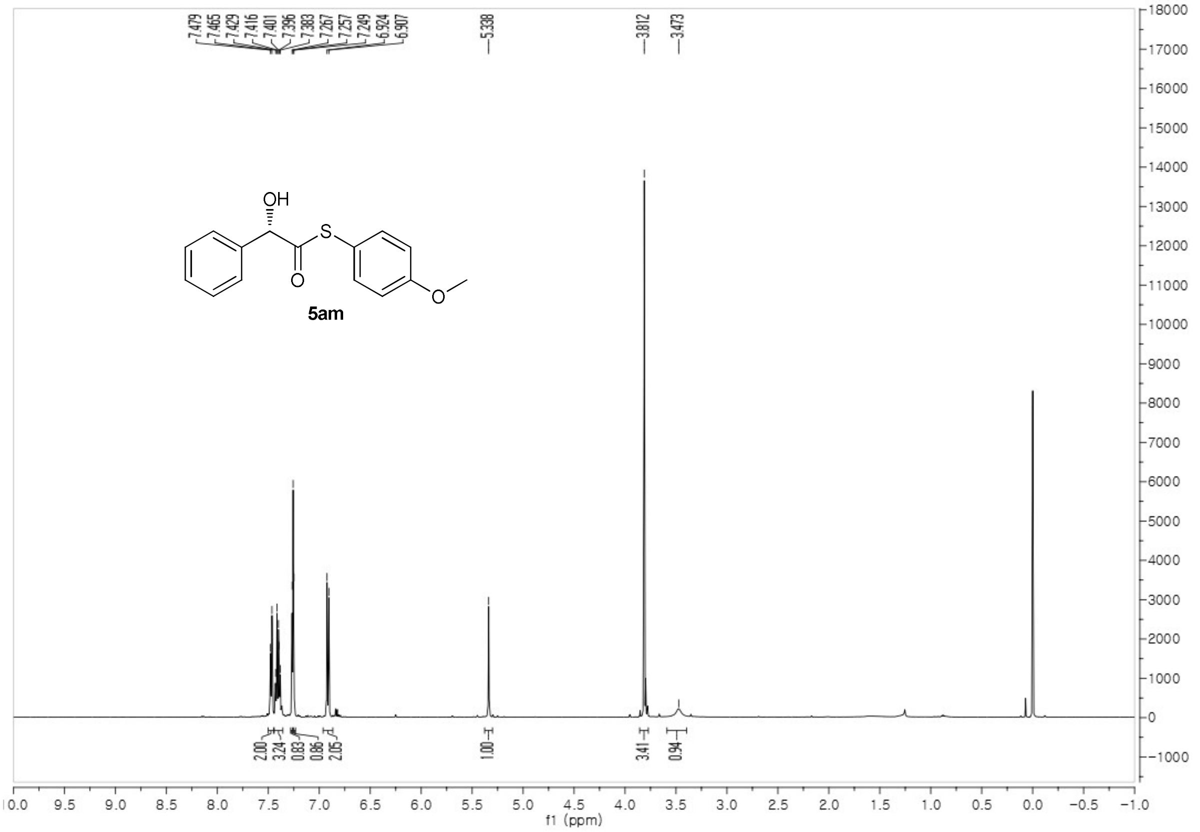

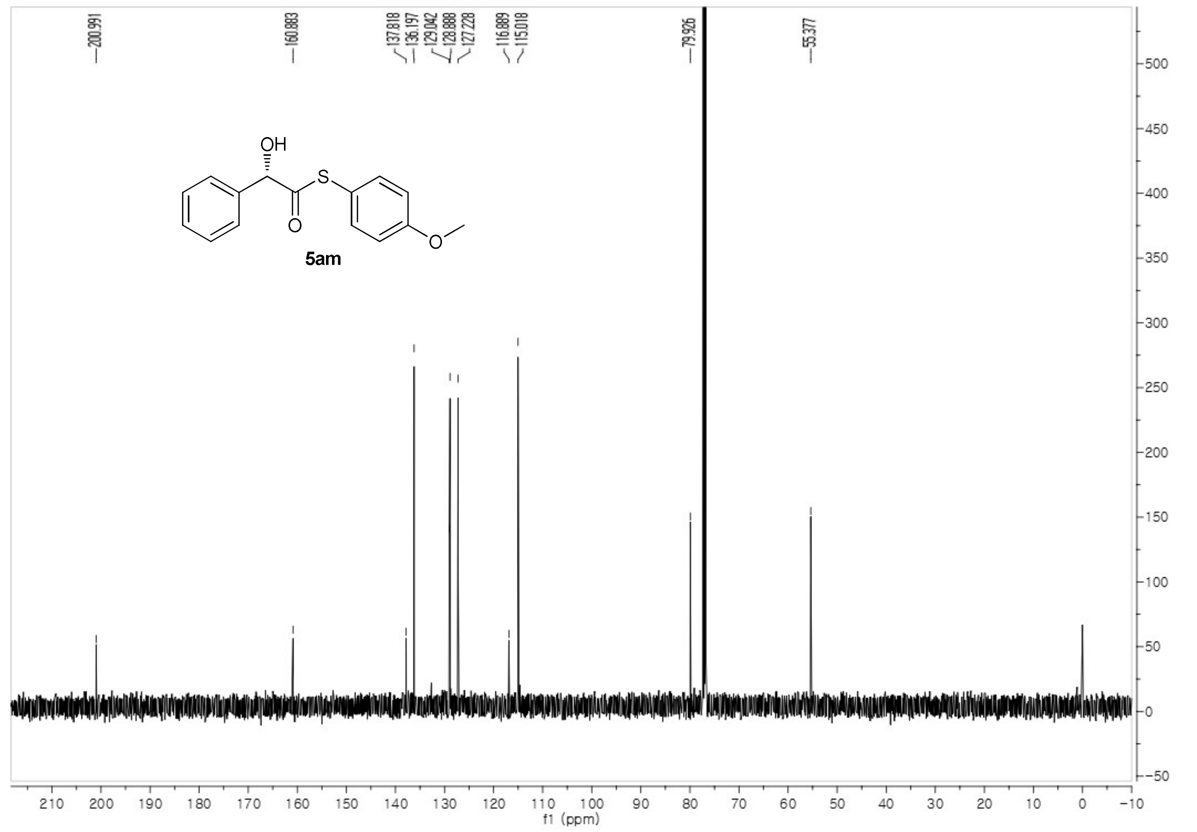

Supplementary Figure 16. ^{1}H and ^{13}C NMR spectra of compound **5an**.

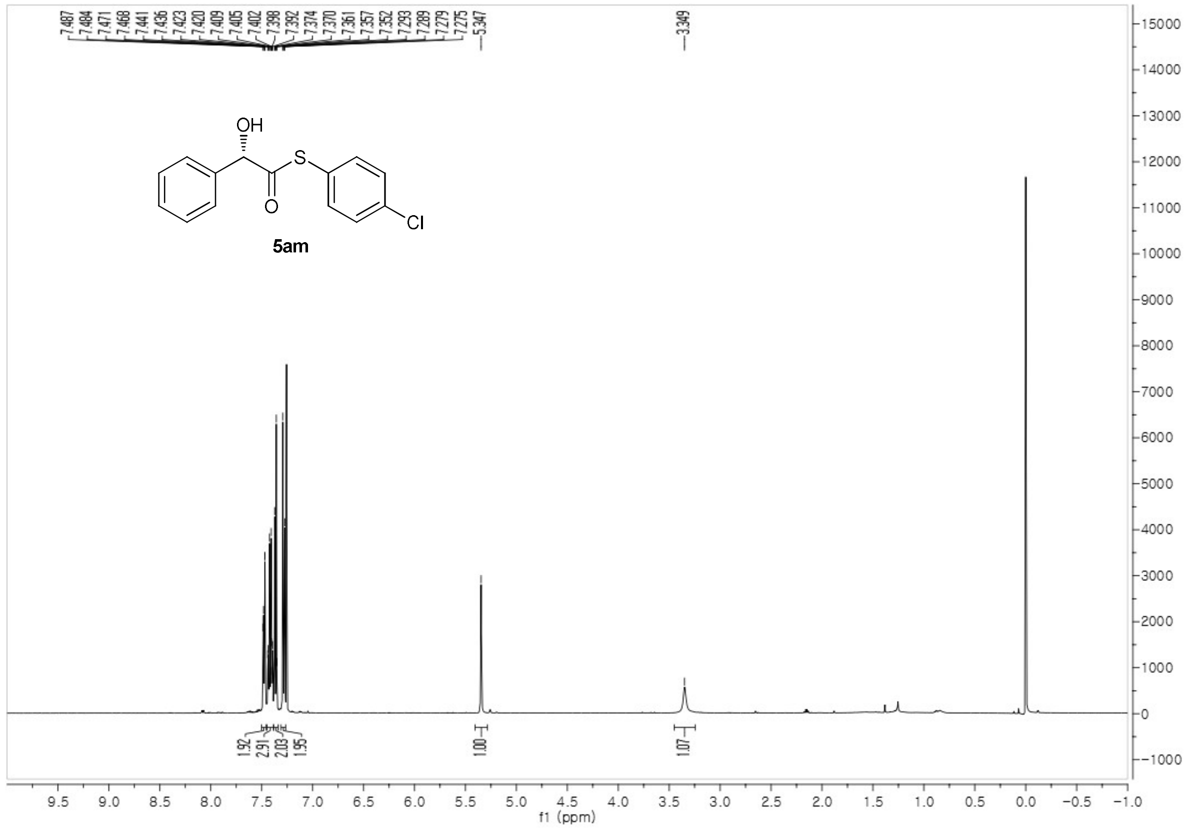

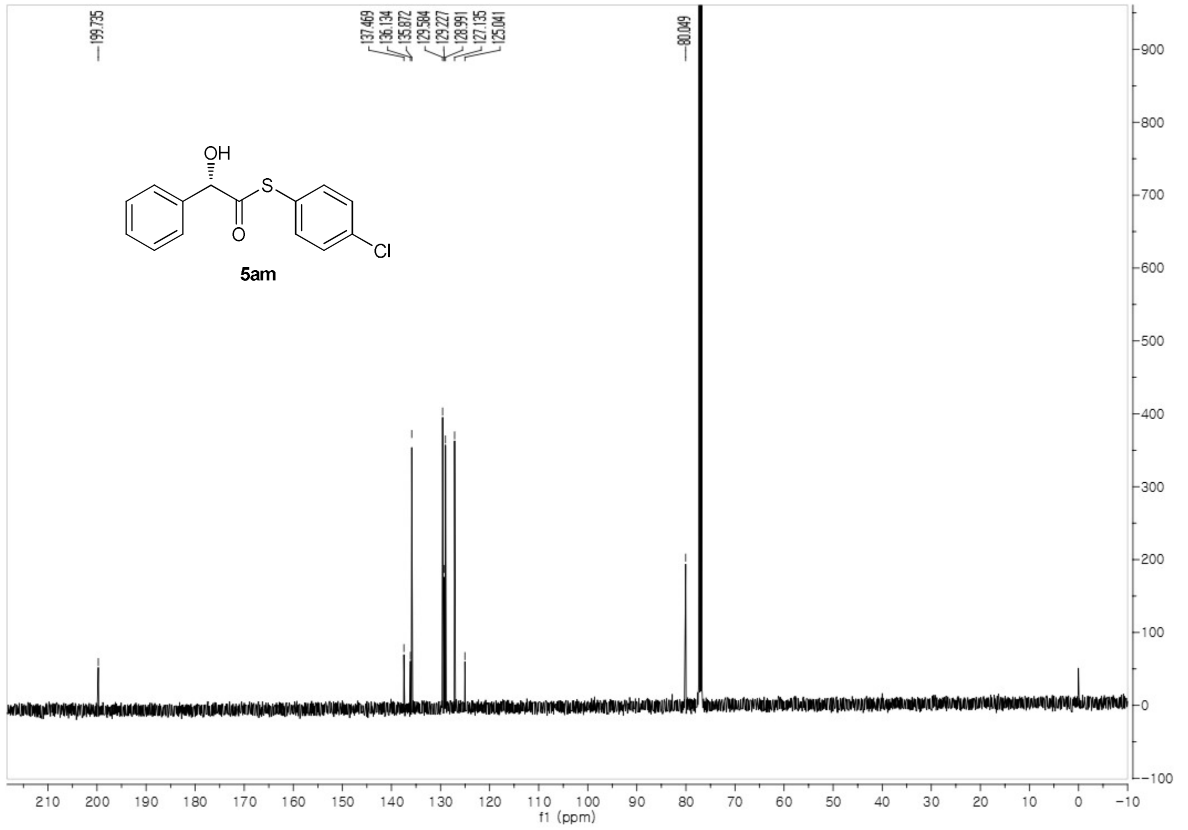

Supplementary Figure 17. ^{1}H and ^{13}C NMR spectra of compound **5ao**.

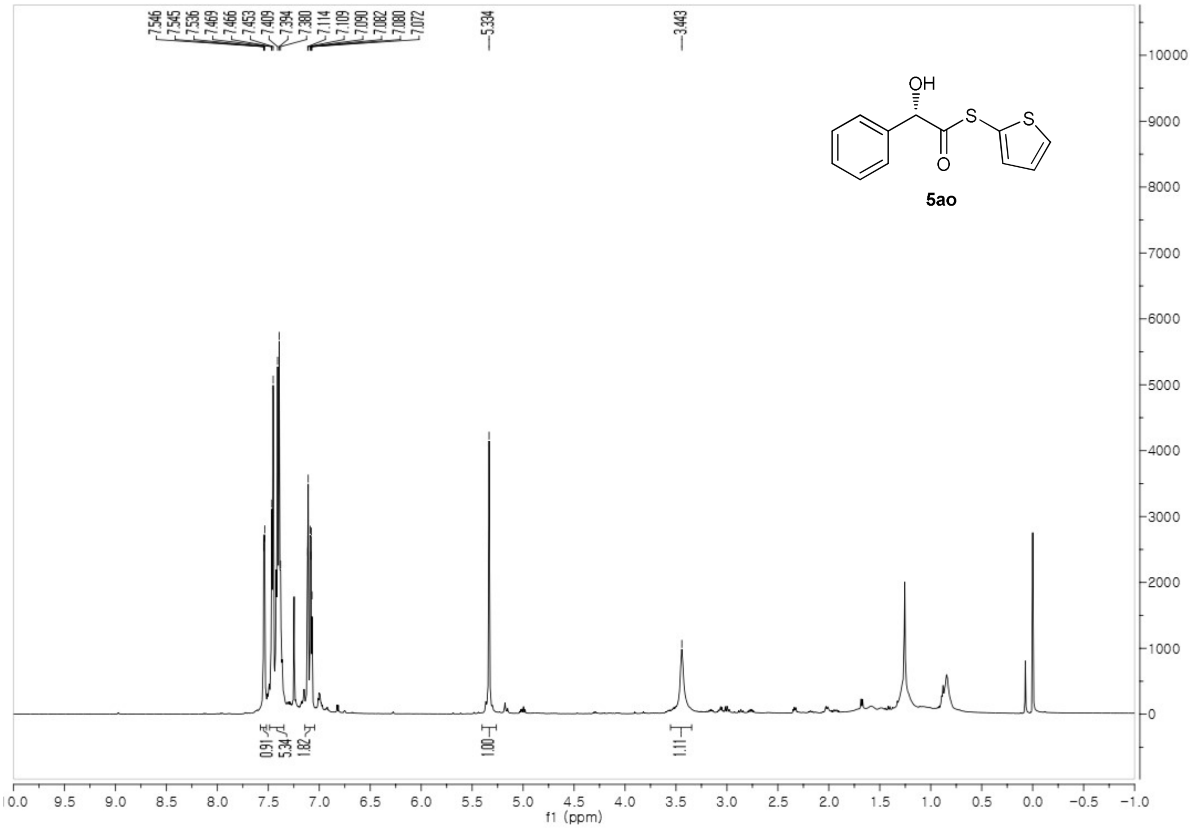

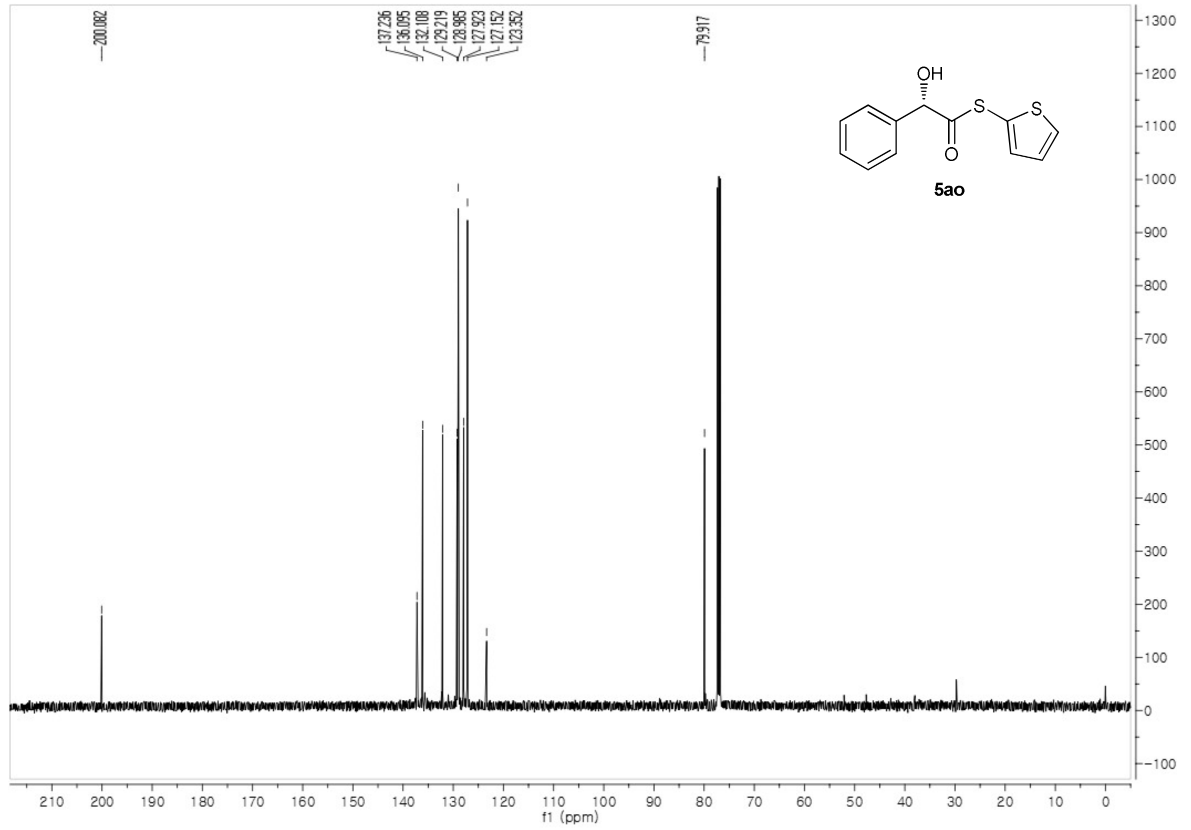

Supplementary Figure 18. ^{1}H and ^{13}C NMR spectra of compound **5b**.

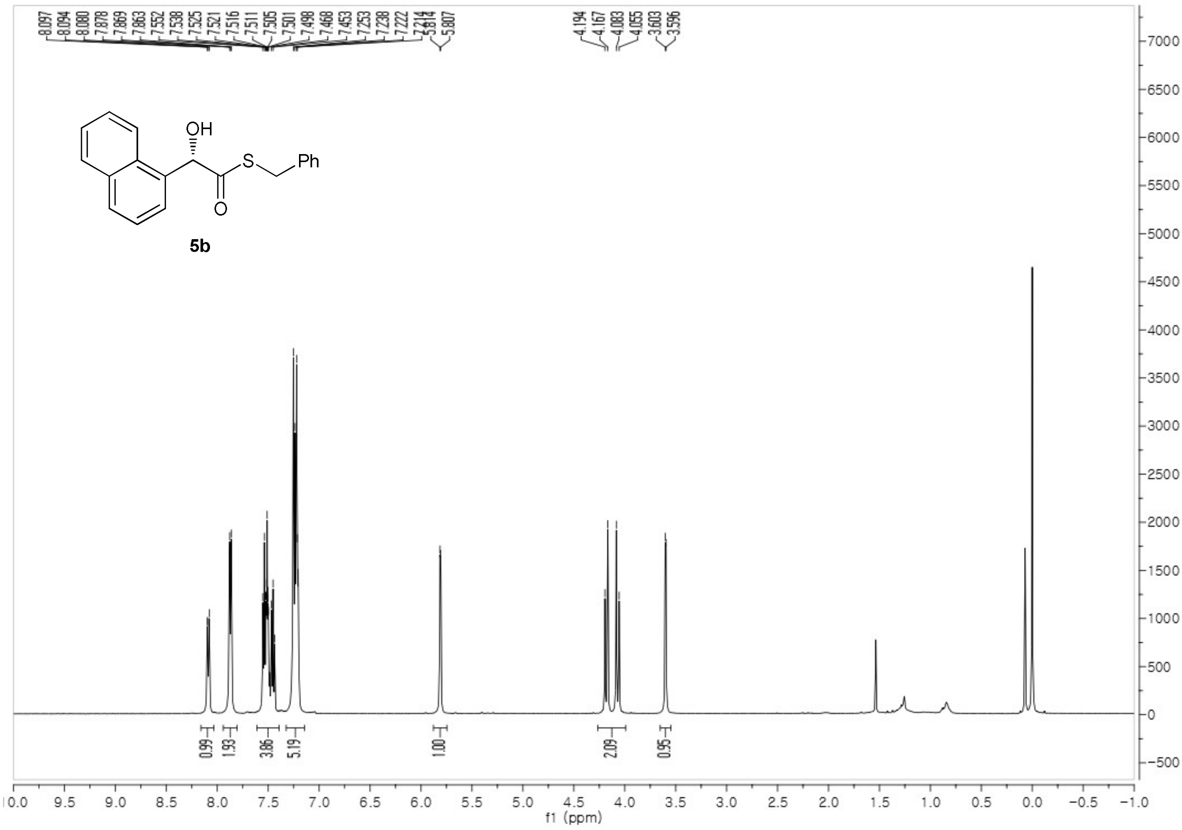

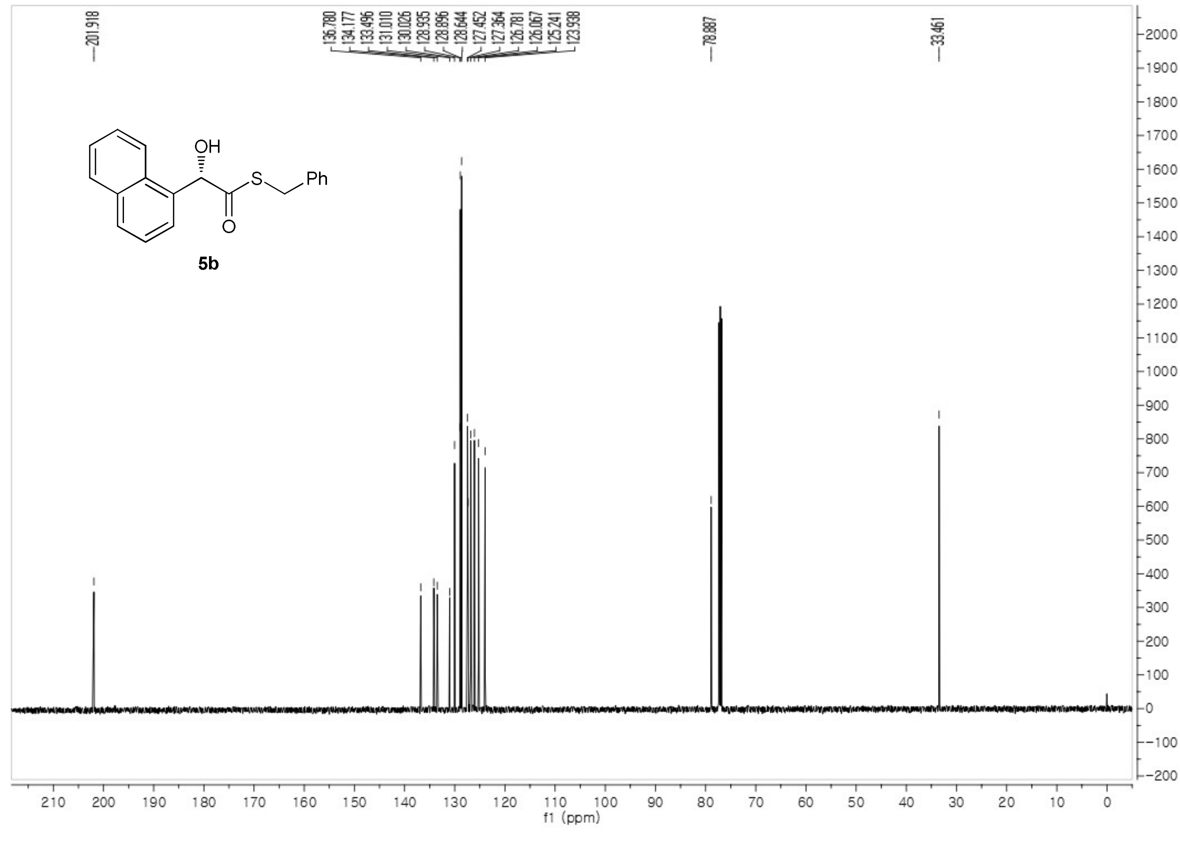

Supplementary Figure 19. ^{1}H and ^{13}C NMR spectra of compound **5c**.

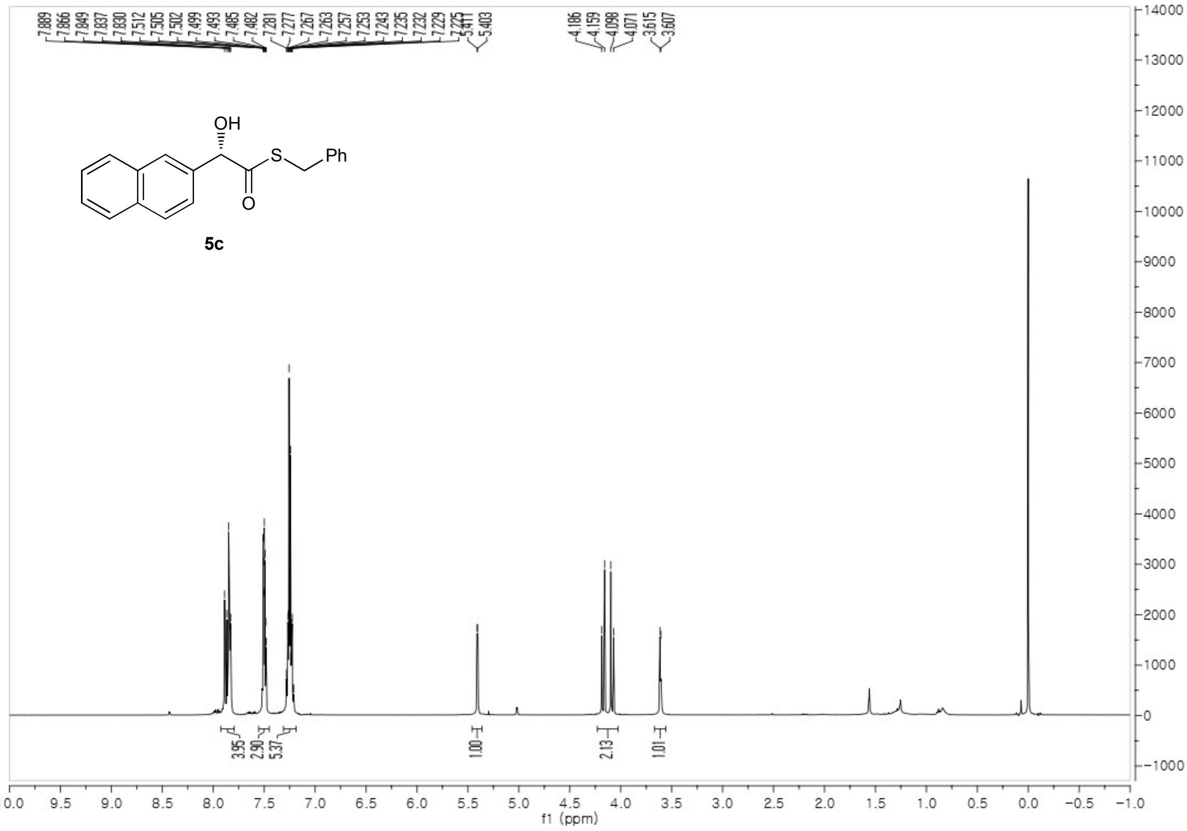

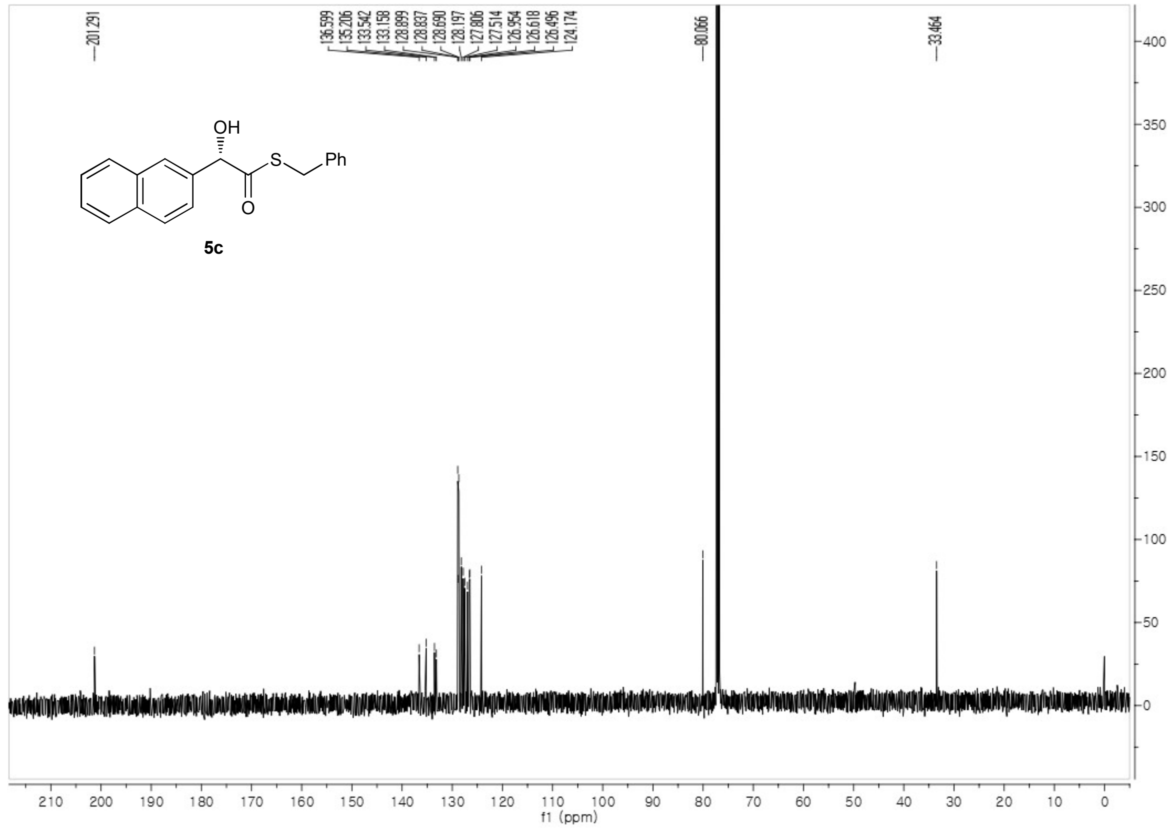

Supplementary Figure 20. ^{1}H and ^{13}C NMR spectra of compound **5d**.

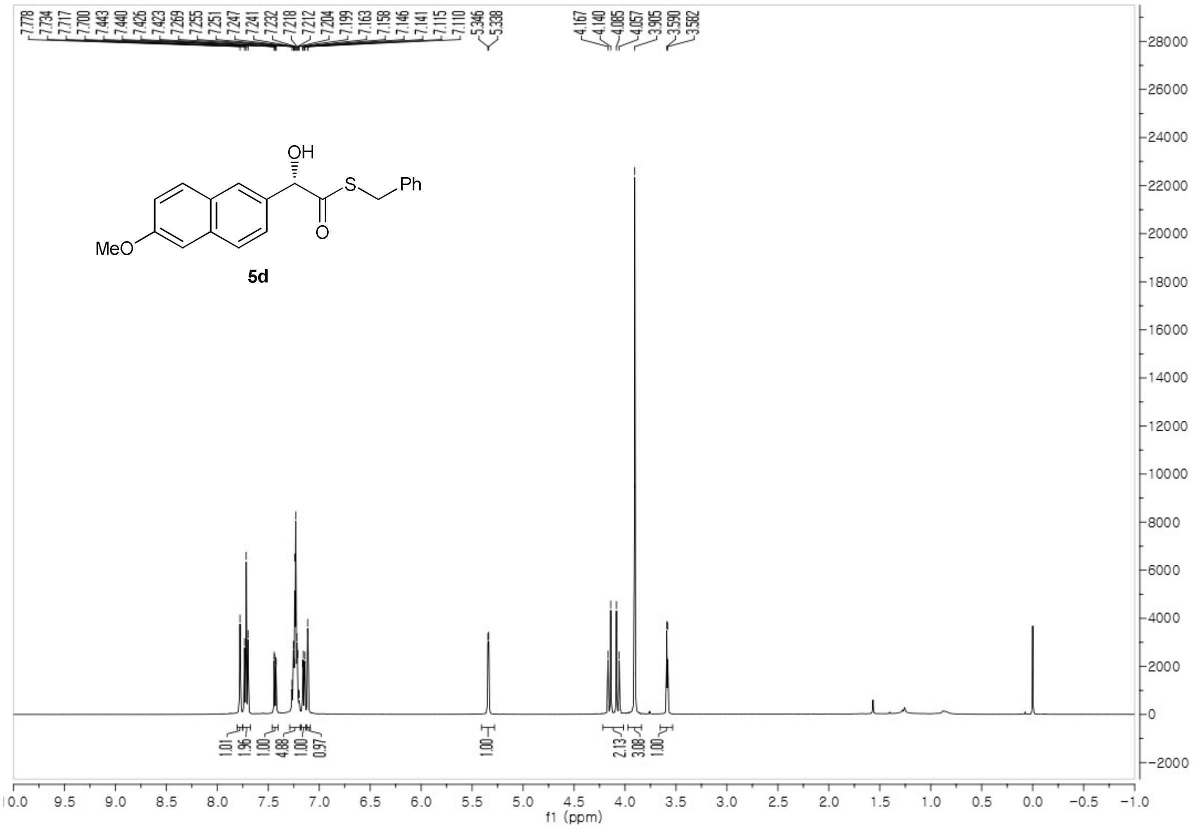

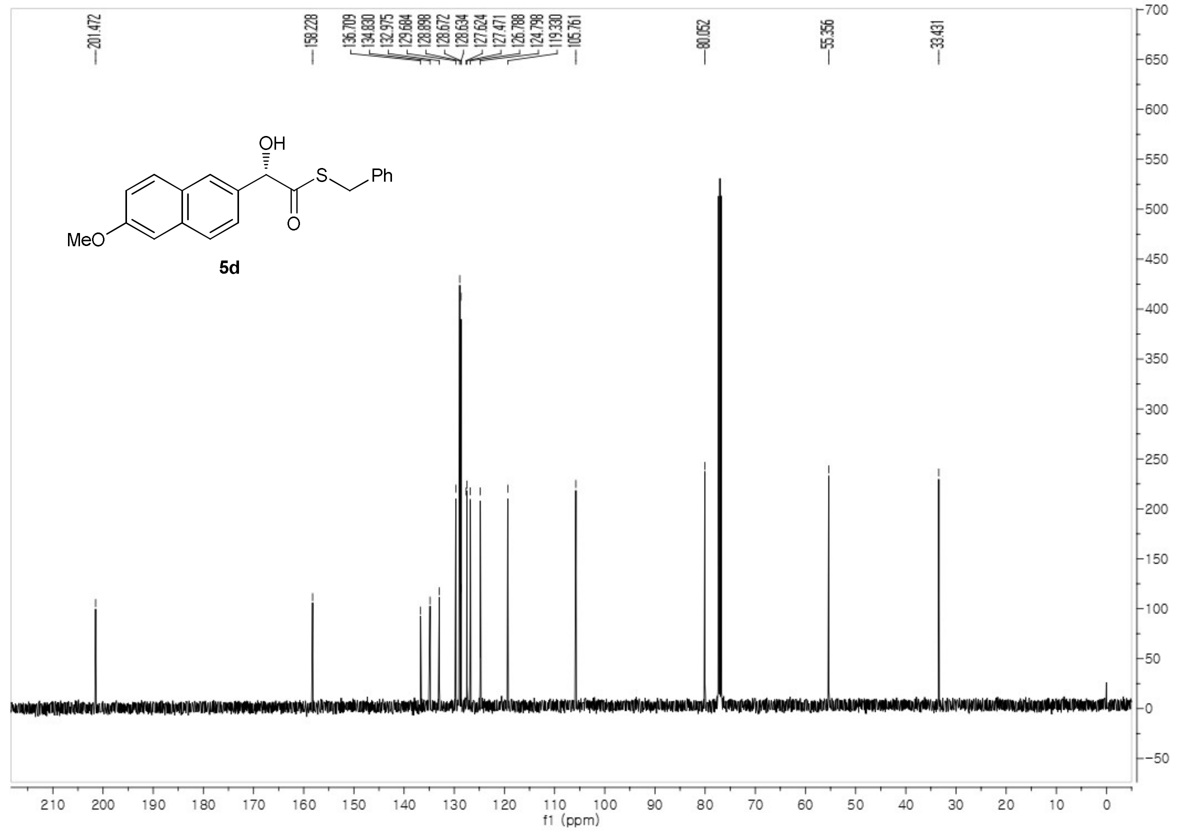

Supplementary Figure 21. ^{1}H and ^{13}C NMR spectra of compound **5e**.

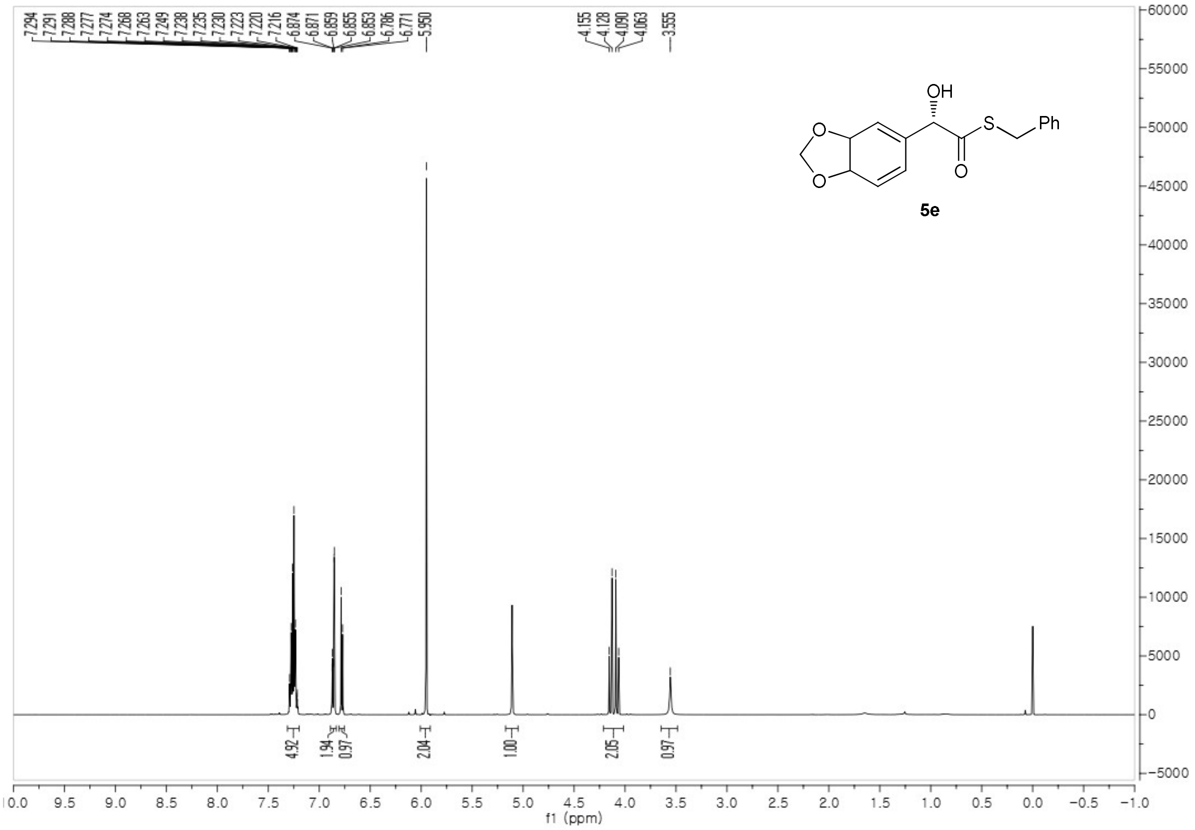

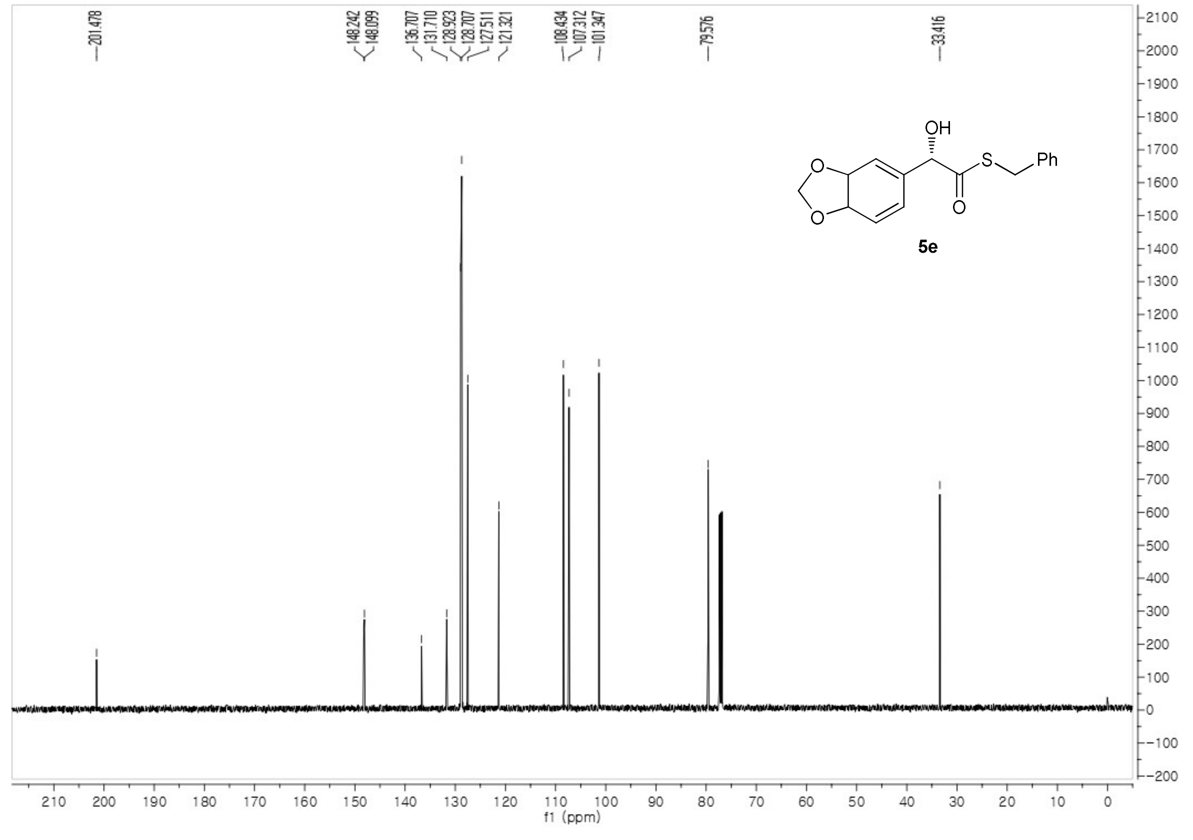

Supplementary Figure 22. ^{1}H and ^{13}C NMR spectra of compound **5f**.

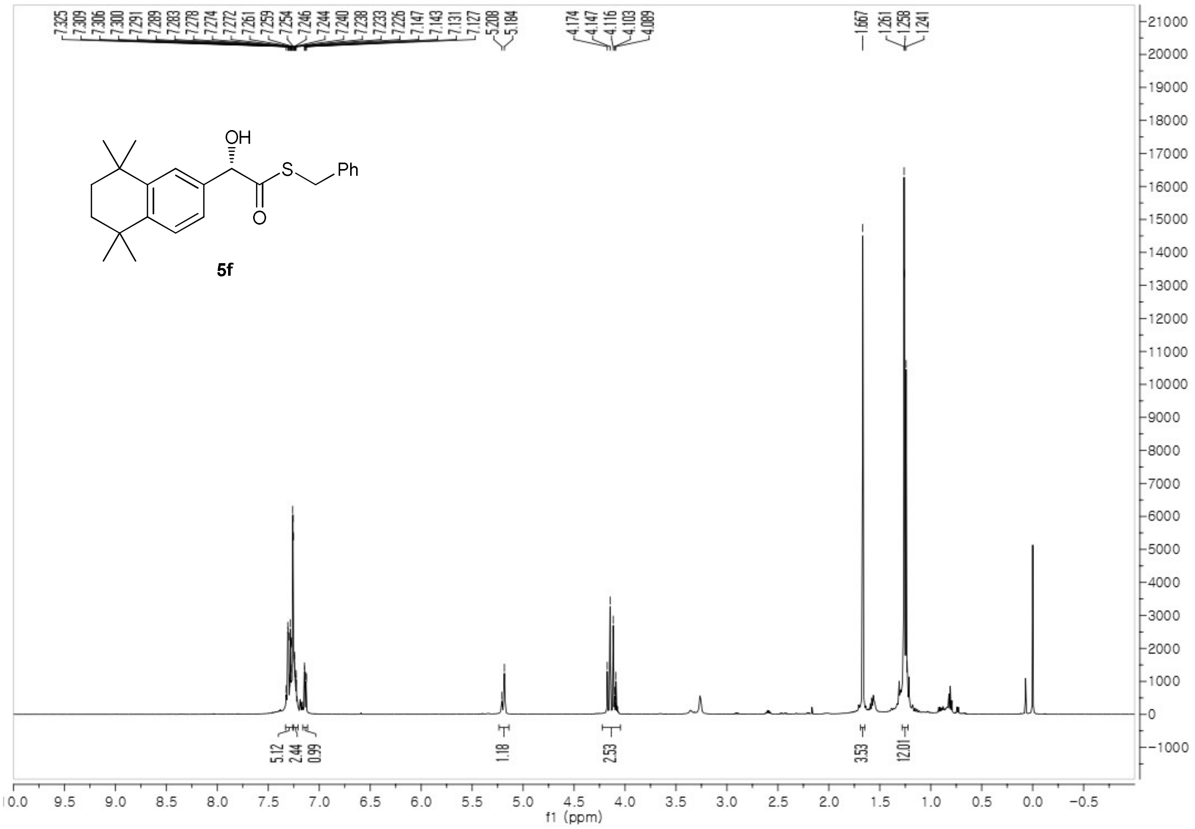

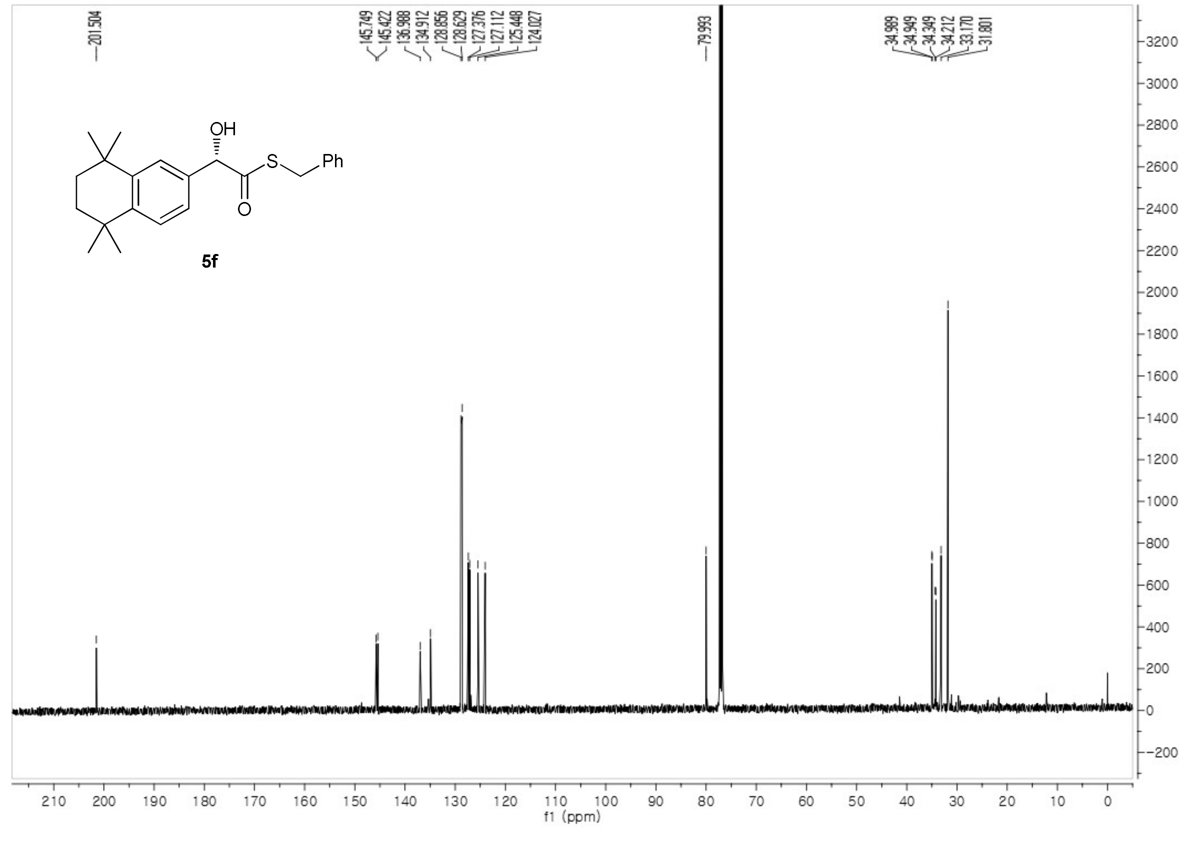

Supplementary Figure 23. ^{1}H and ^{13}C NMR spectra of compound **5g**.

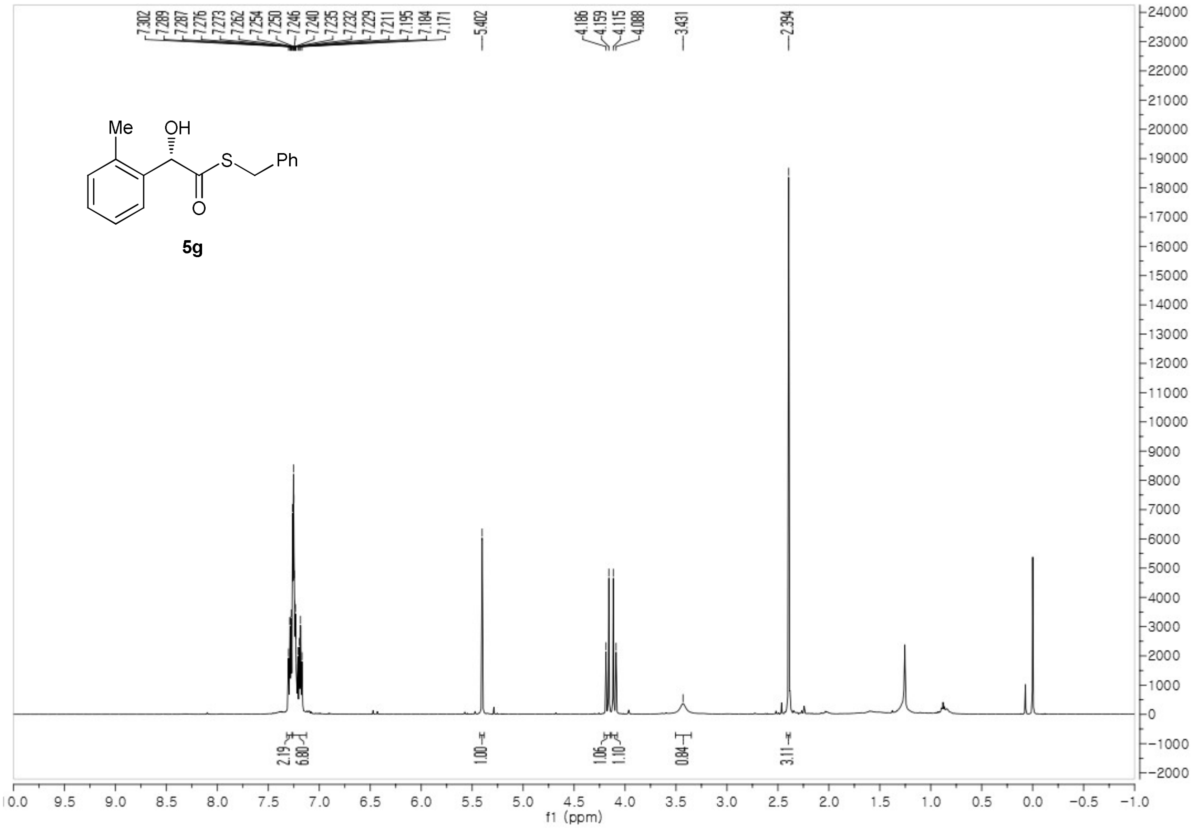

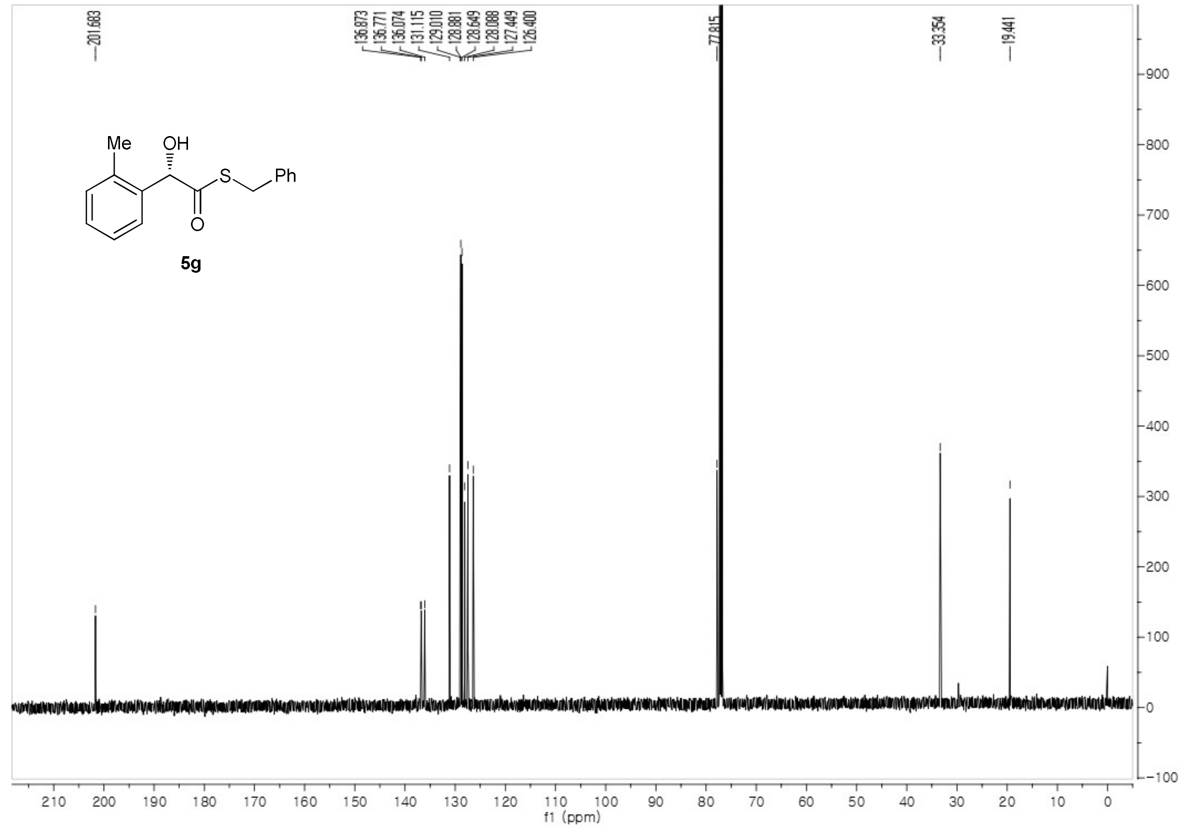

Supplementary Figure 24. ^{1}H and ^{13}C NMR spectra of compound **5h**.

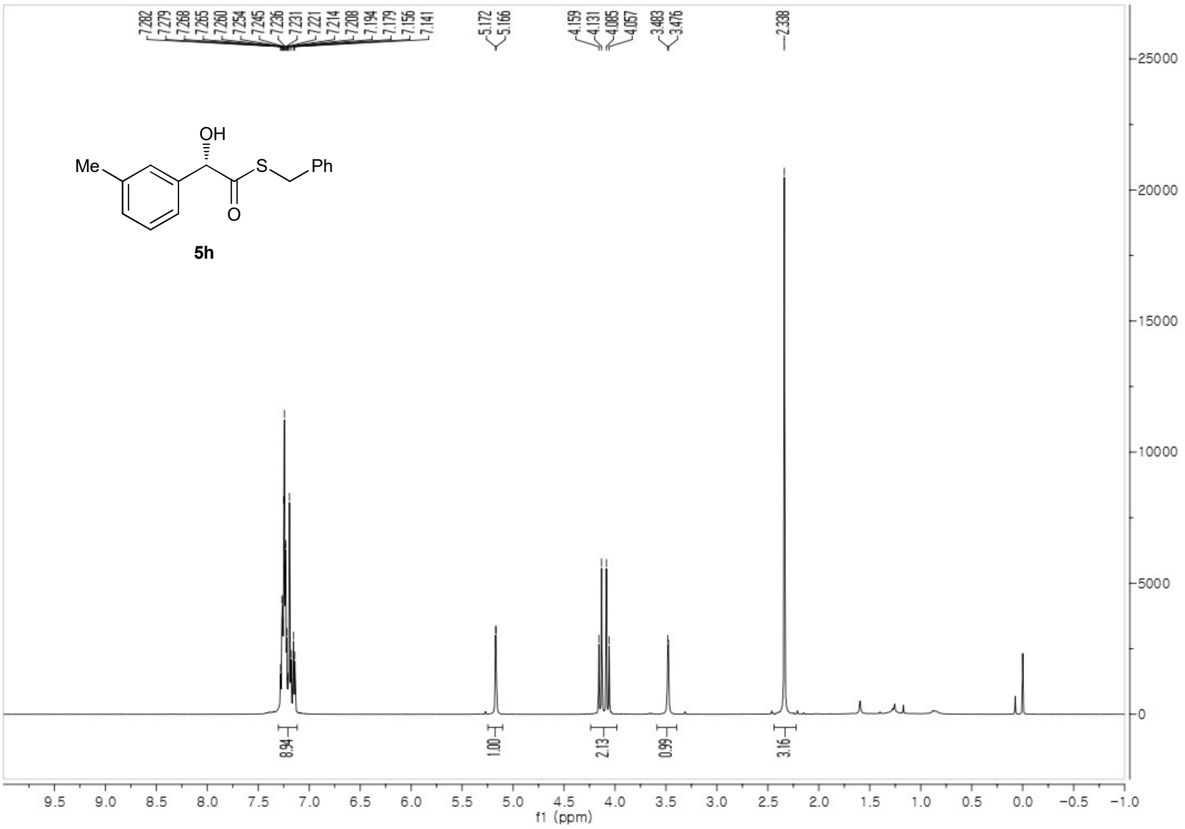

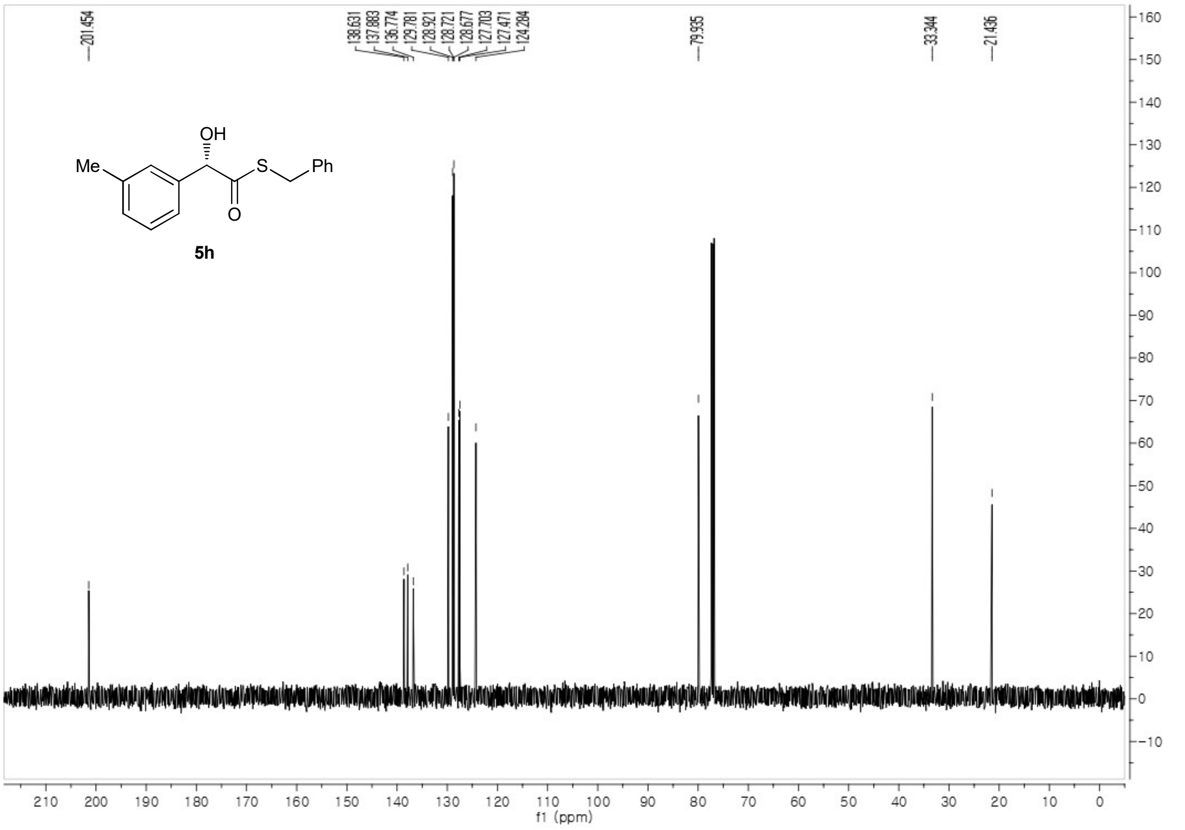

Supplementary Figure 25. ^{1}H and ^{13}C NMR spectra of compound **5i**.

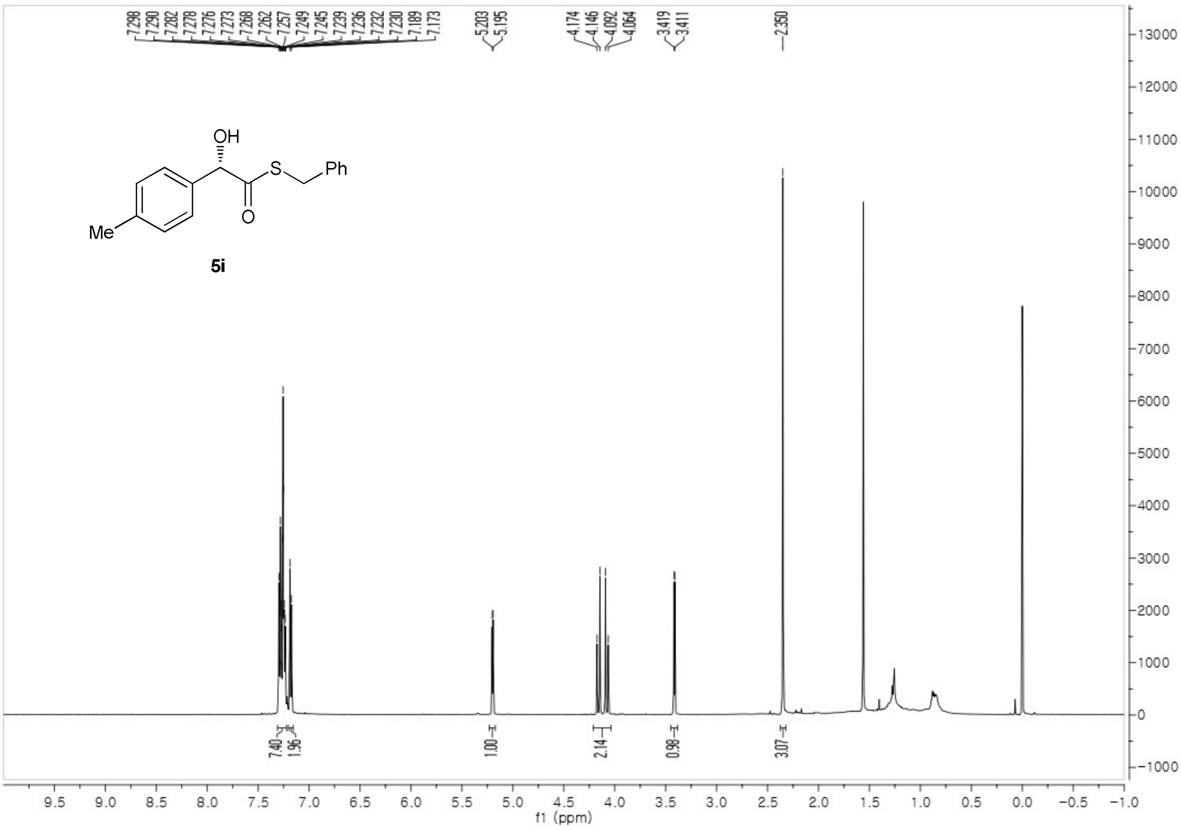

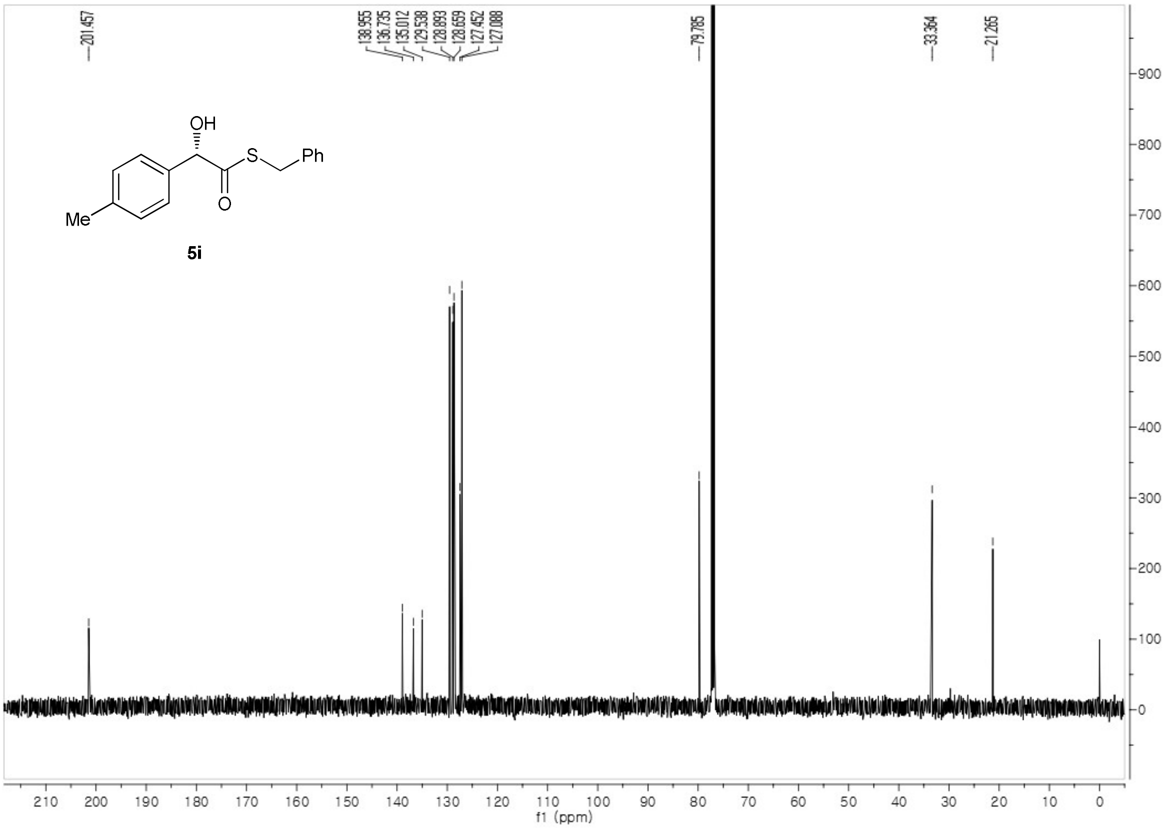

Supplementary Figure 26. ^1H and ^{13}C NMR spectra of compound **5j**.

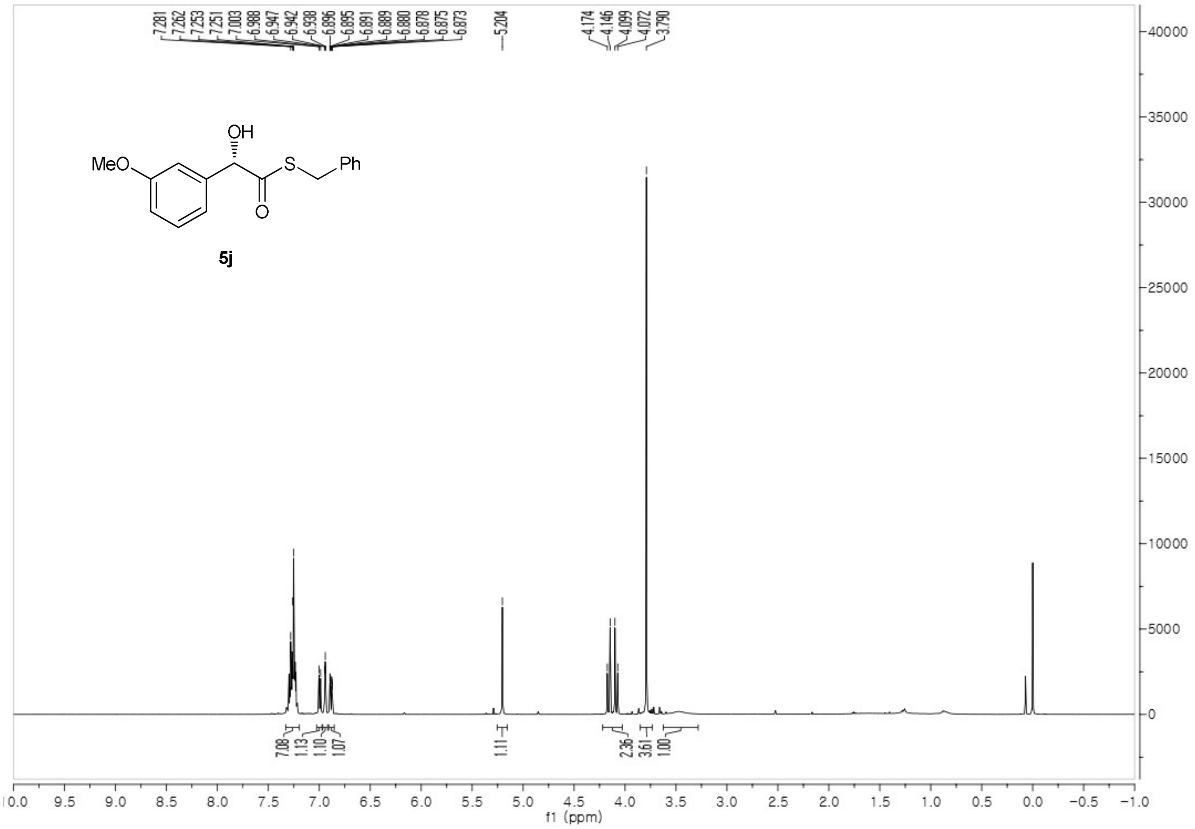

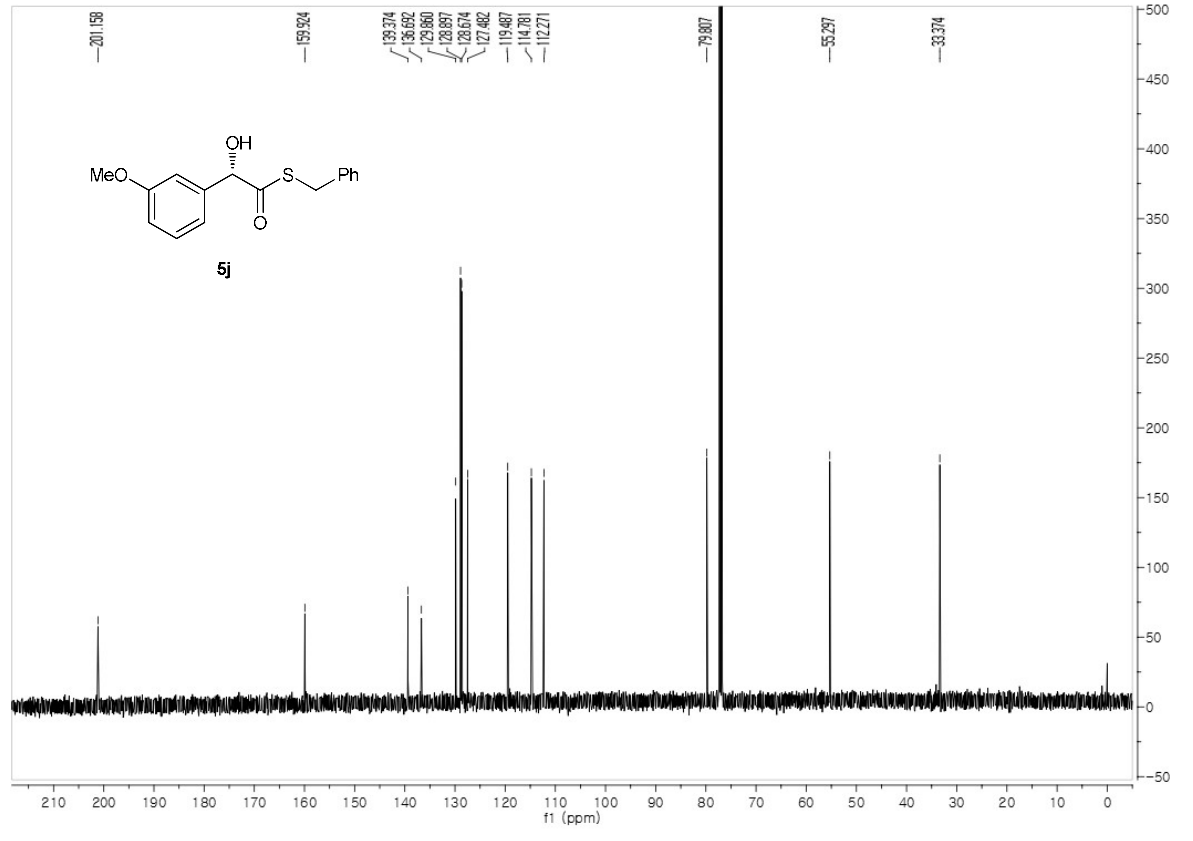

Supplementary Figure 27. ^{1}H and ^{13}C NMR spectra of compound **5k**.

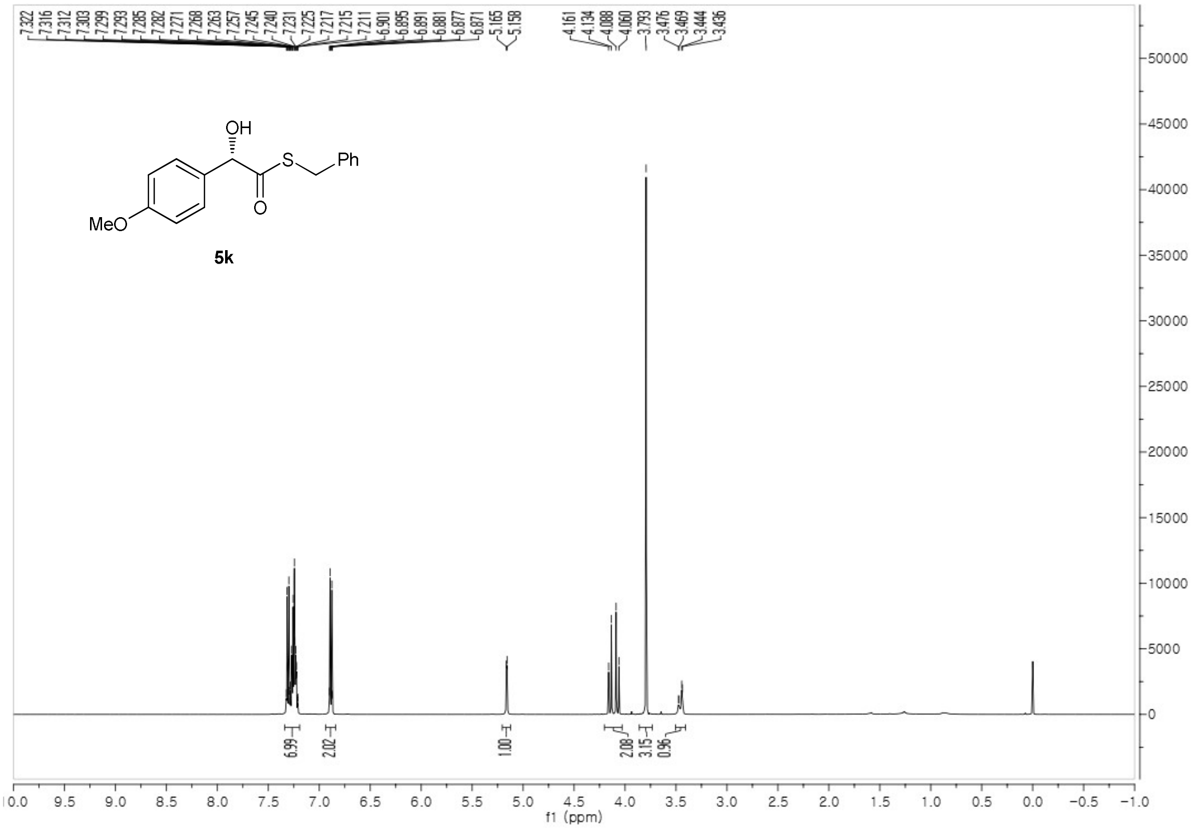

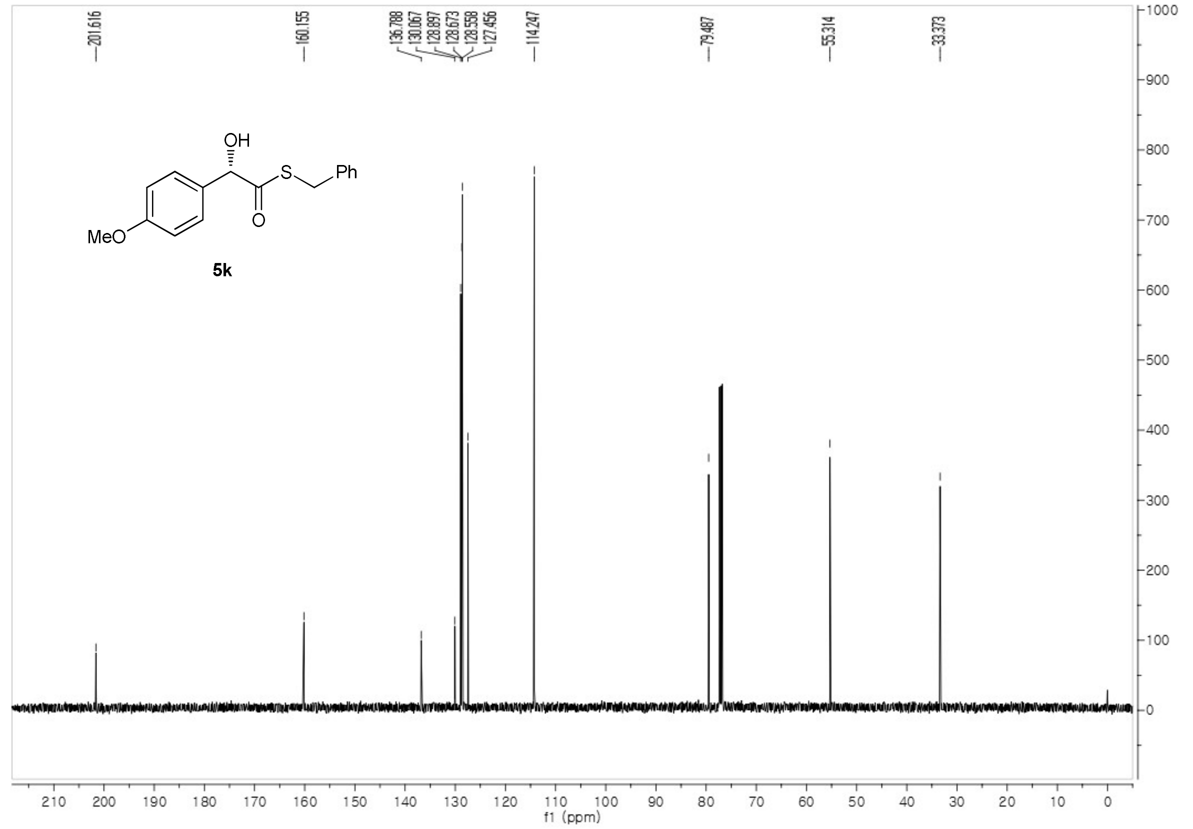

Supplementary Figure 28. ^{1}H and ^{13}C NMR spectra of compound **5l**.

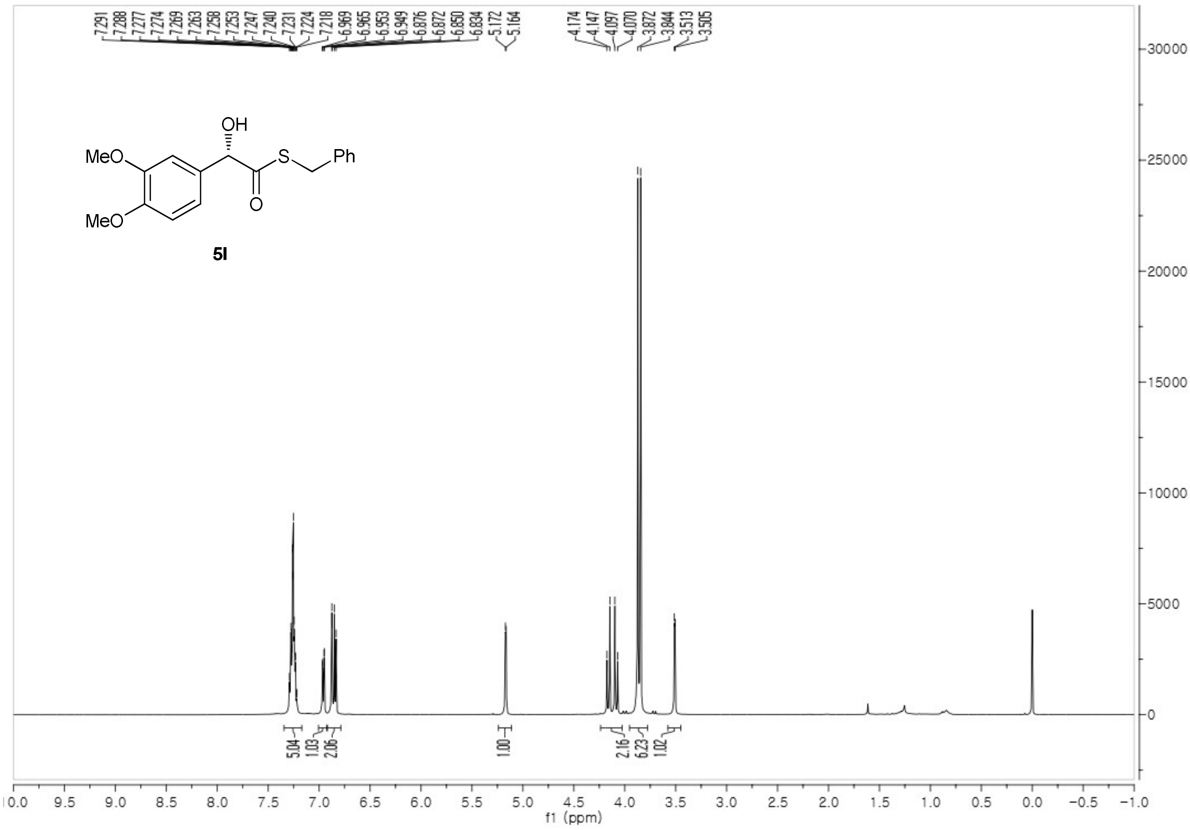

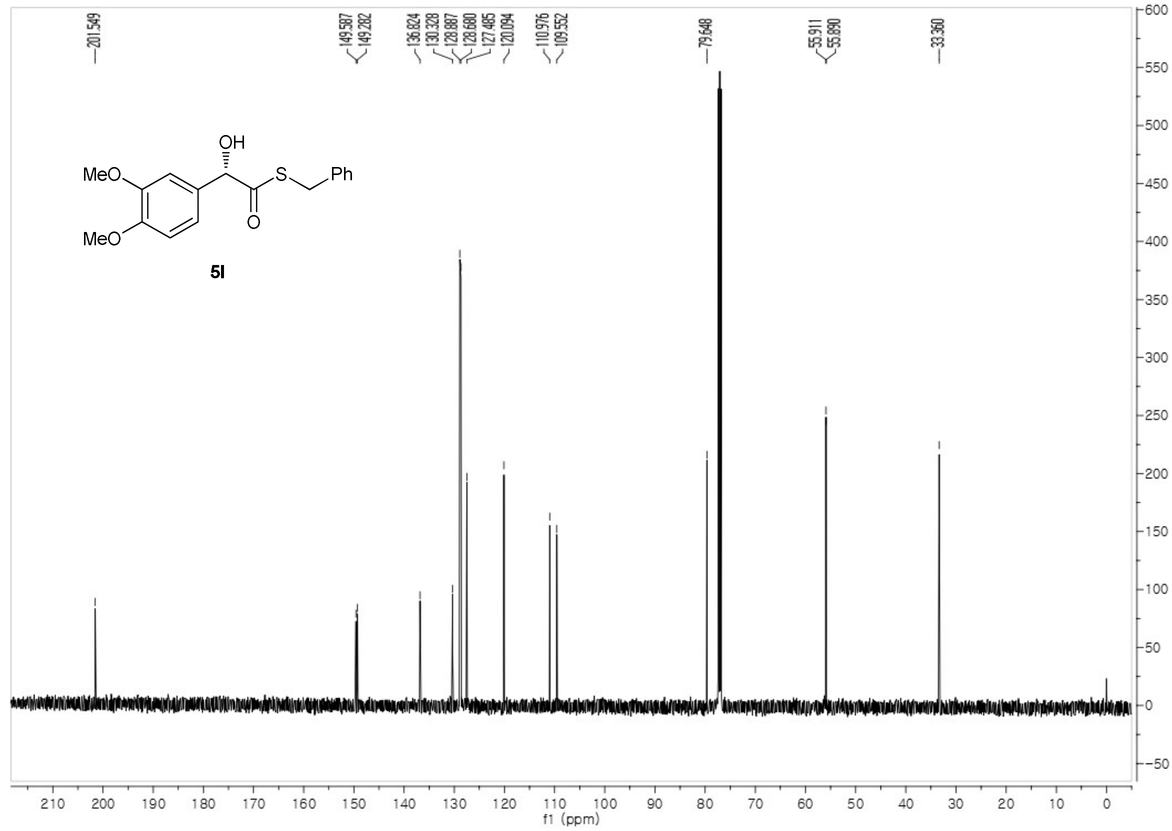

Supplementary Figure 29. ^{1}H and ^{13}C NMR spectra of compound **5m**.

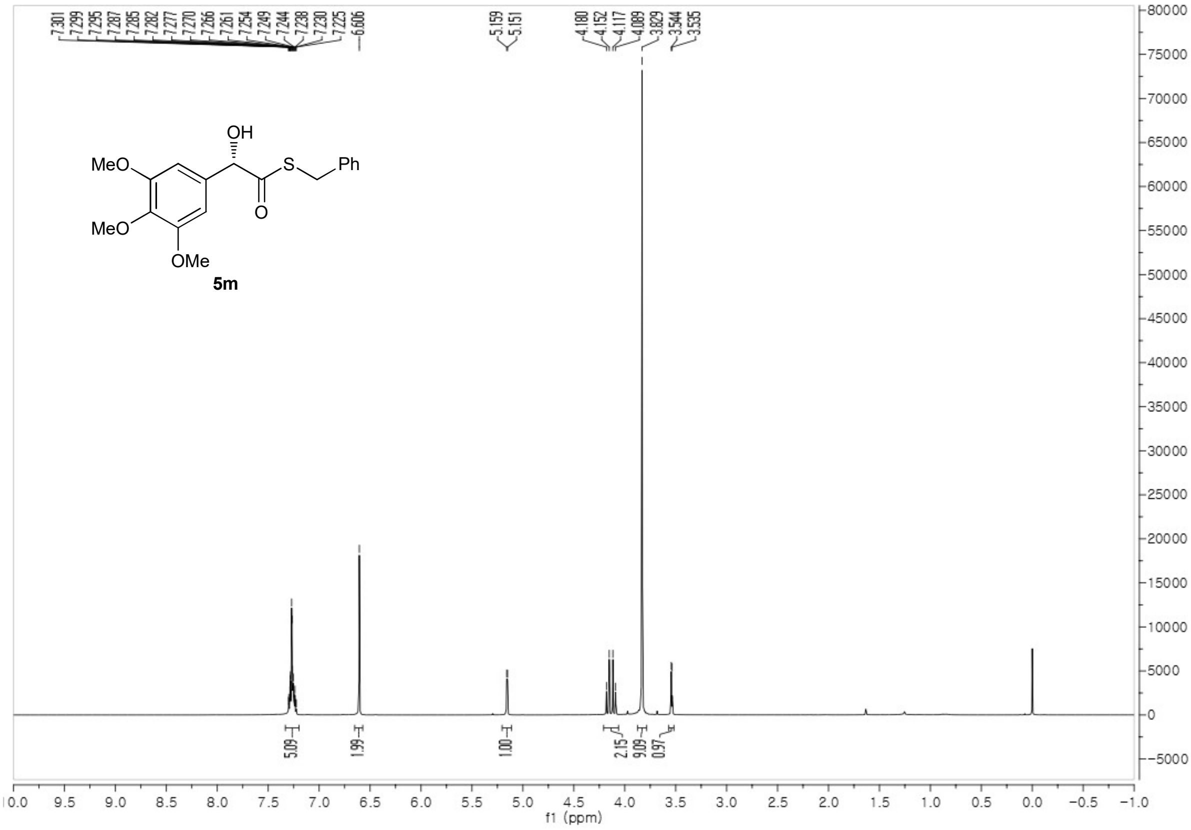

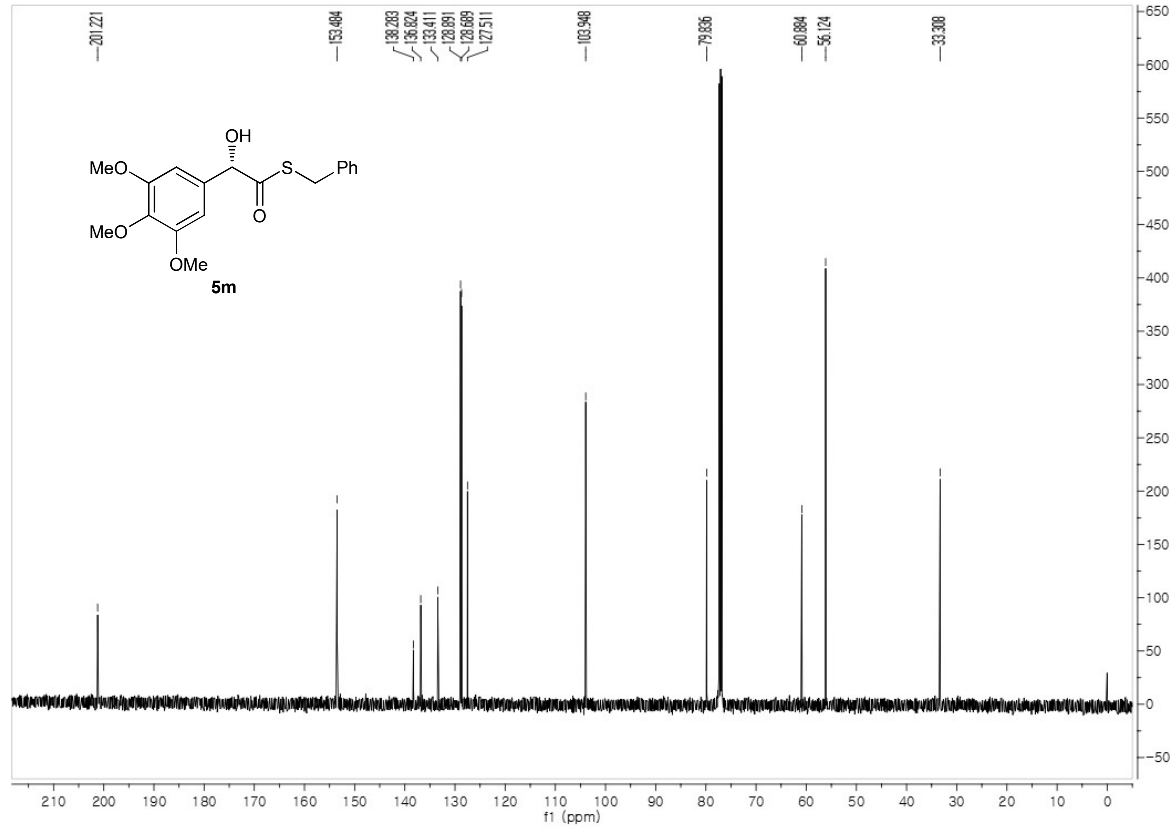

Supplementary Figure 30. ^{1}H, ^{13}C and ^{19}F NMR spectra of compound **5n**.

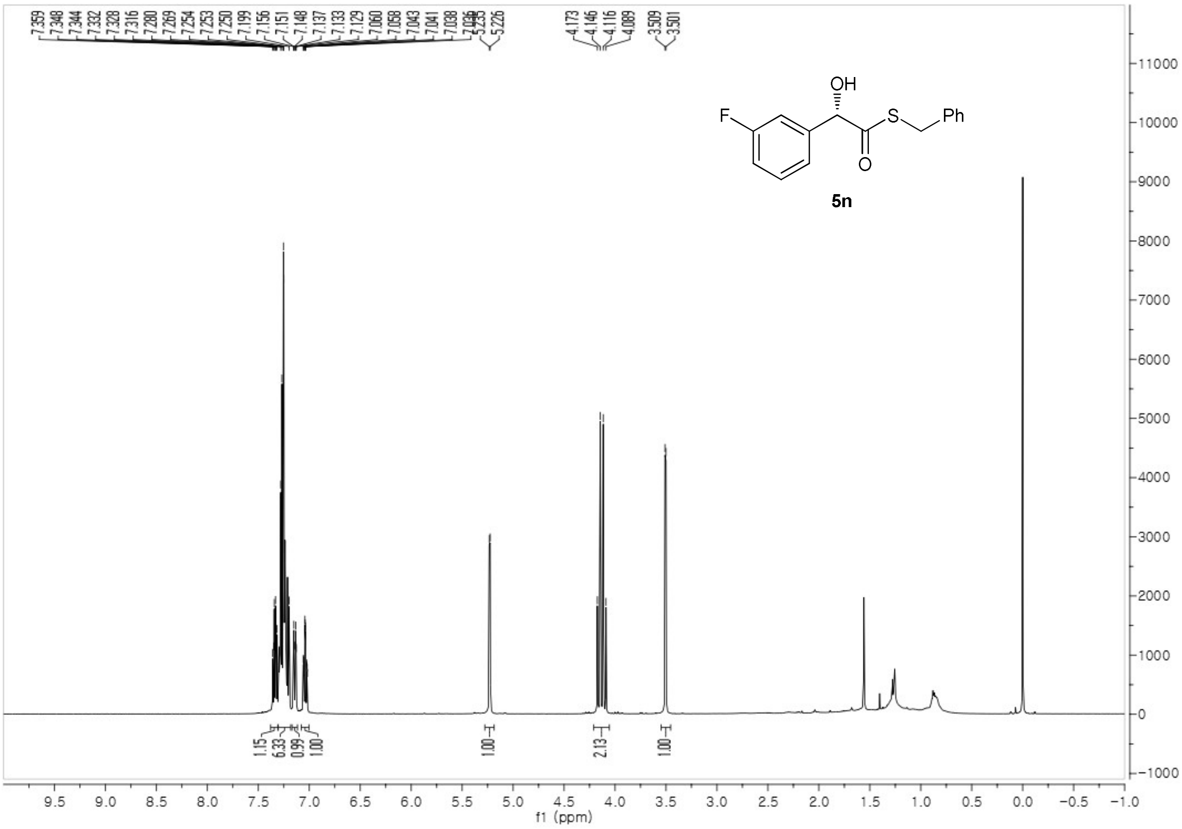

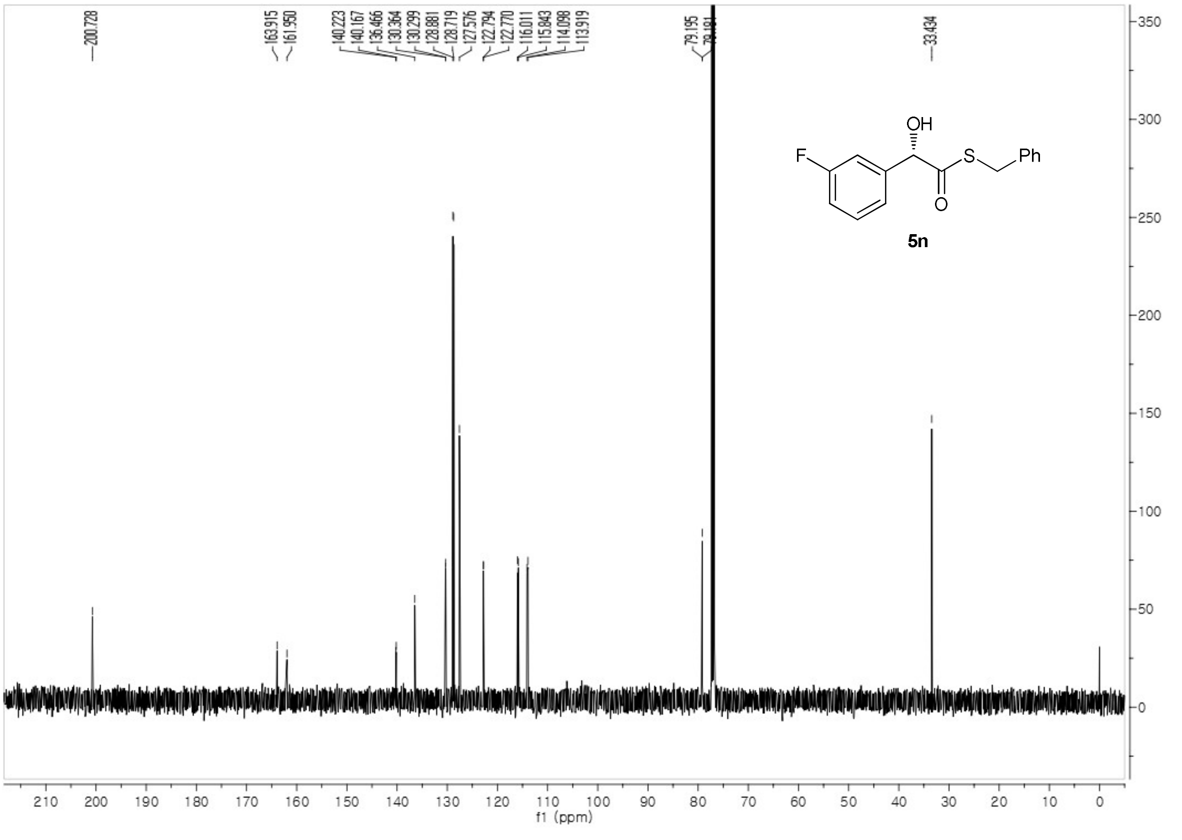

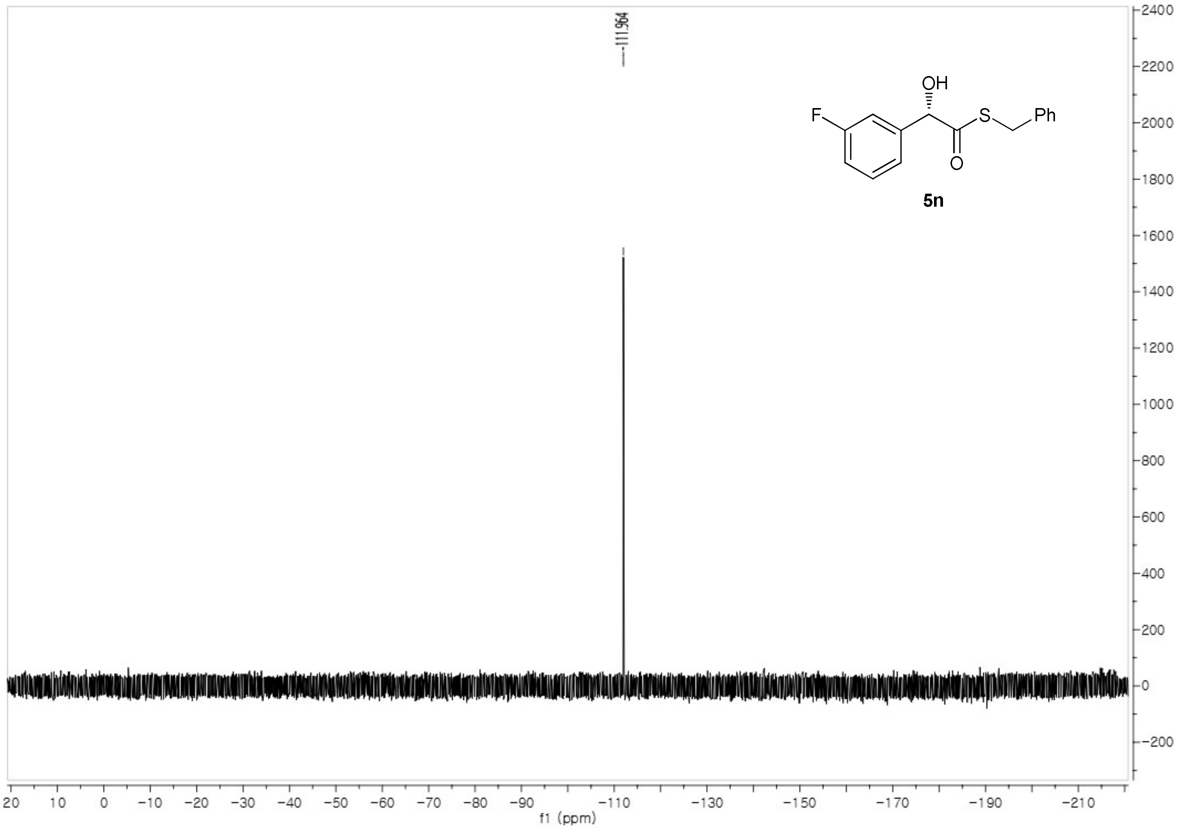

−111.964
OH
F
S
Ph
O
5n
2400
2200
2000
1800
1600
1400
1200
1000
800
600
400
200
0
−200
20
10
0
−10
−20
−30
−40
−50
−60
−70
−80
−90
−110
−130
−150
−170
−190
−210
f1 (ppm)

Supplementary Figure 31. ^{1}H, ^{13}C and ^{19}F NMR spectra of compound **5o**.

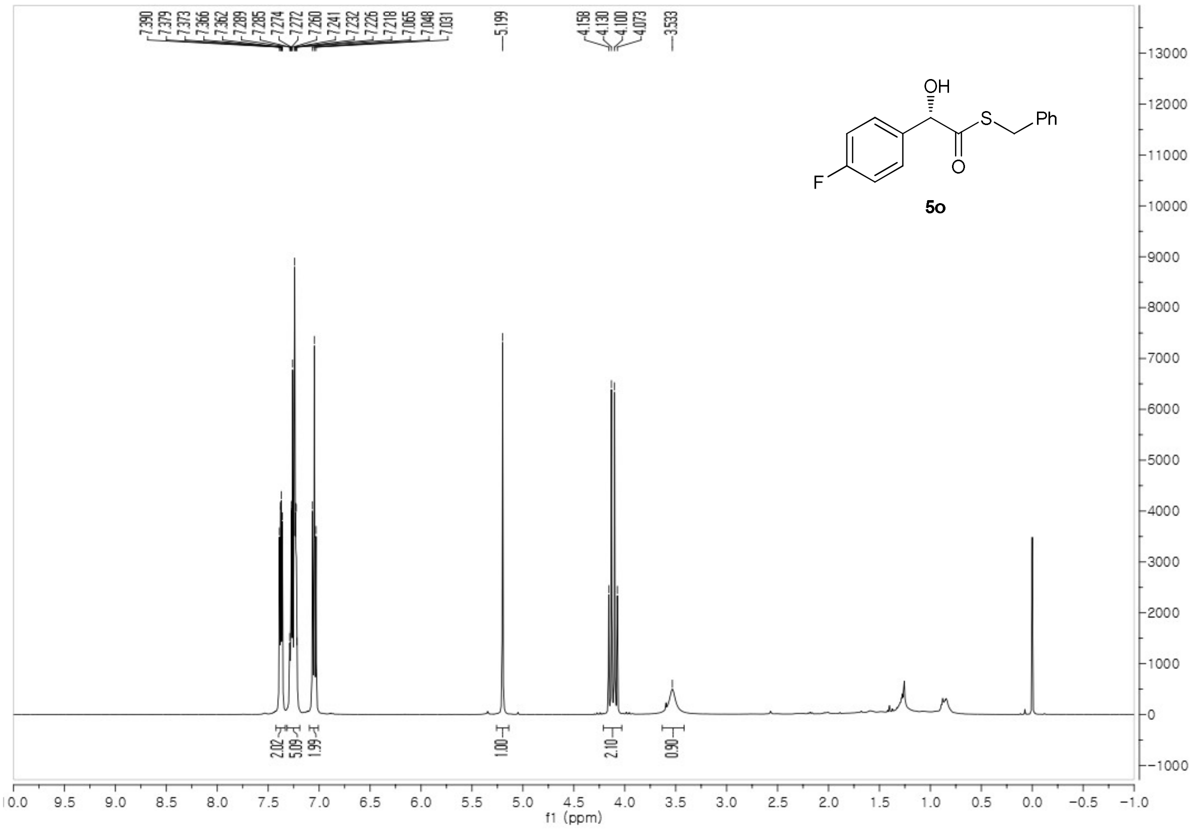

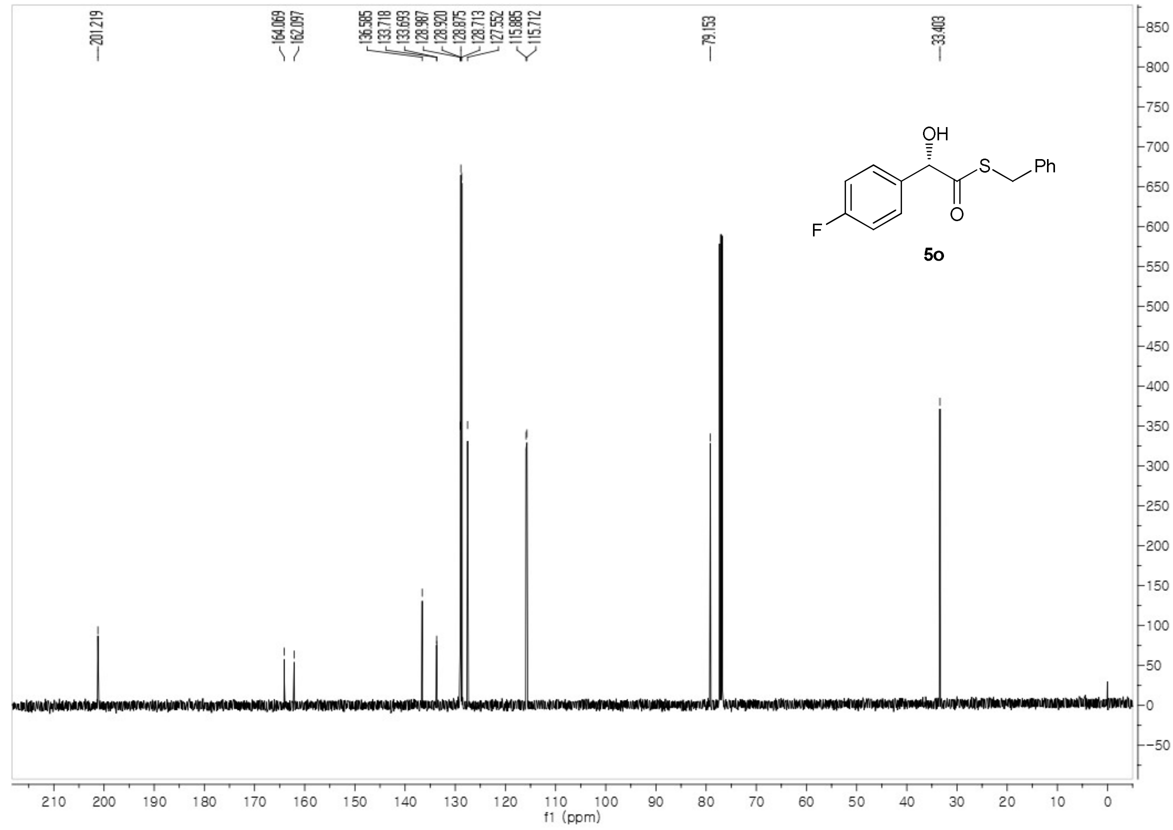

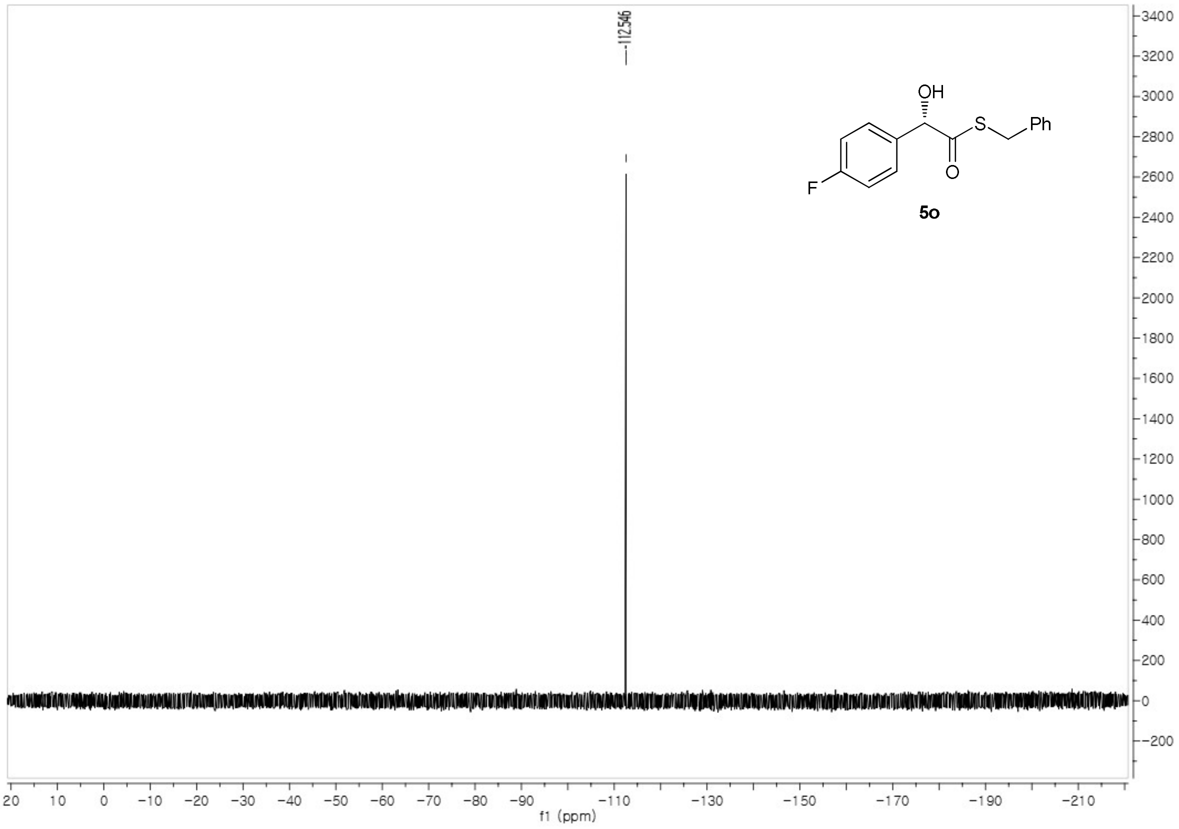
-112.546
OH
S
Ph
F
O
5o
3400
3200
3000
2800
2600
2400
2200
2000
1800
1600
1400
1200
1000
800
600
400
200
0
-200
20 10 0 -10 -20 -30 -40 -50 -60 -70 -80 -90 -110 -130 -150 -170 -190 -210
f1 (ppm)

Supplementary Figure 32. ^{1}H and ^{13}C NMR spectra of compound **5p**.

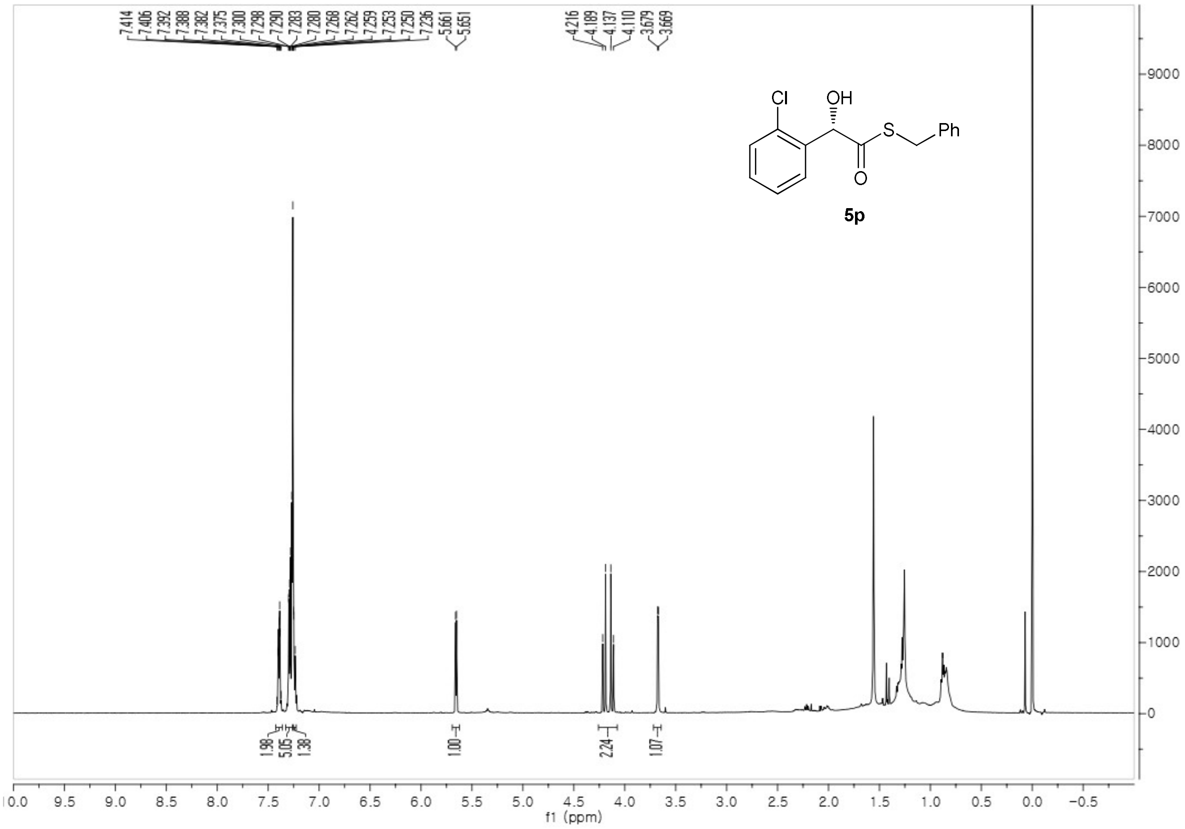

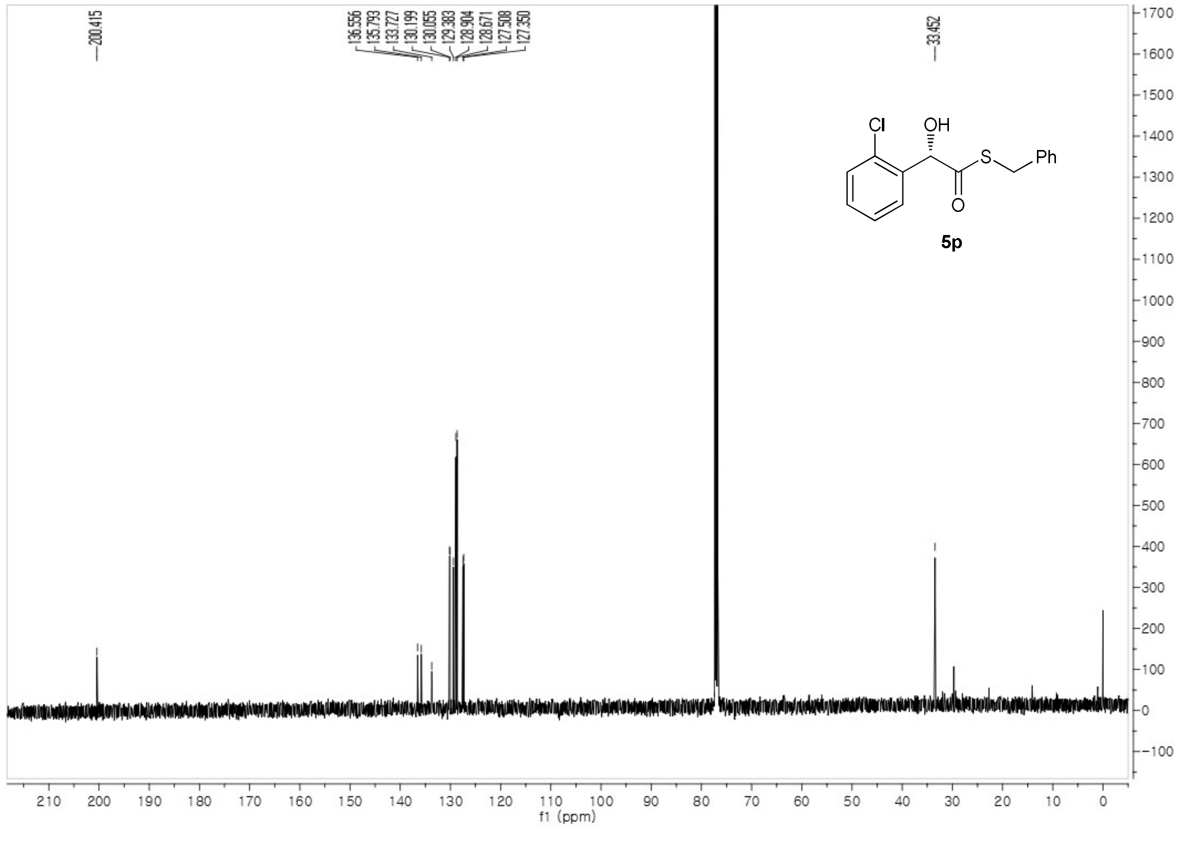

Supplementary Figure 33. ^1H and ^{13}C NMR spectra of compound **5q**.

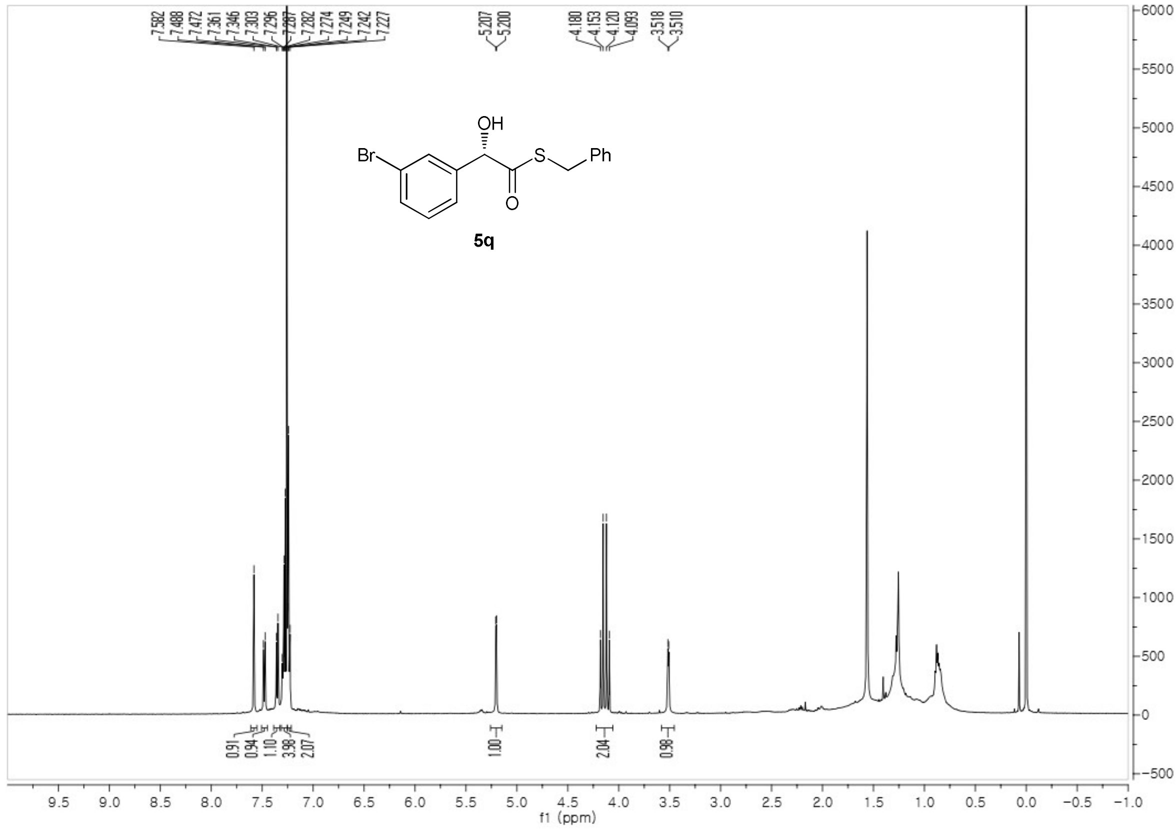

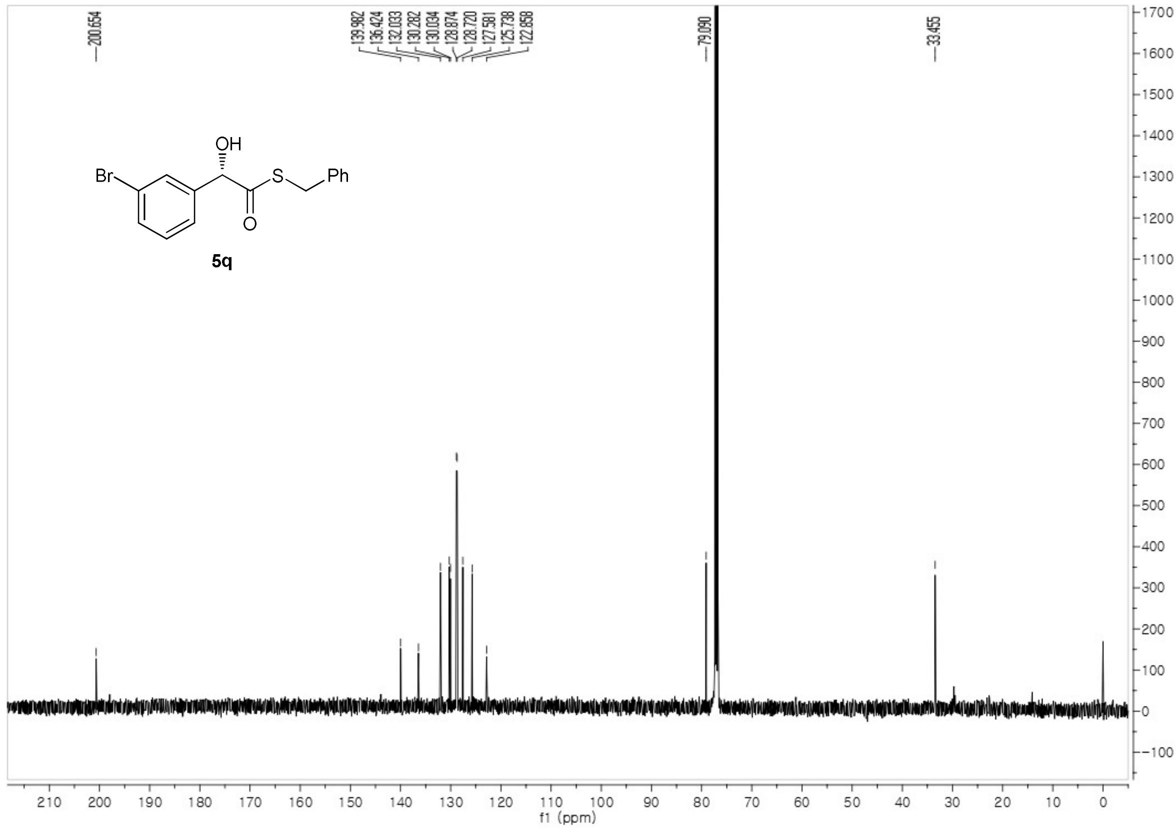

Supplementary Figure 34. ^{1}H, ^{13}C and ^{19}F NMR spectra of compound **5r**.

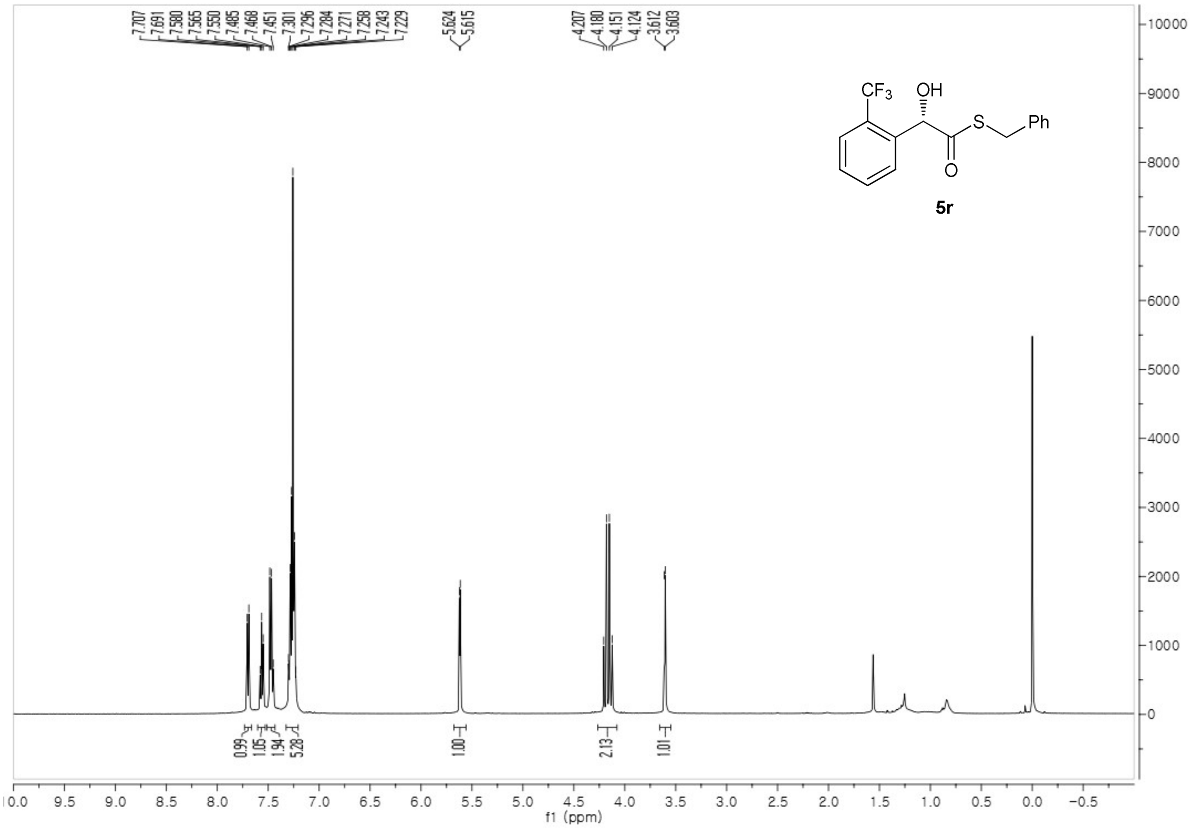

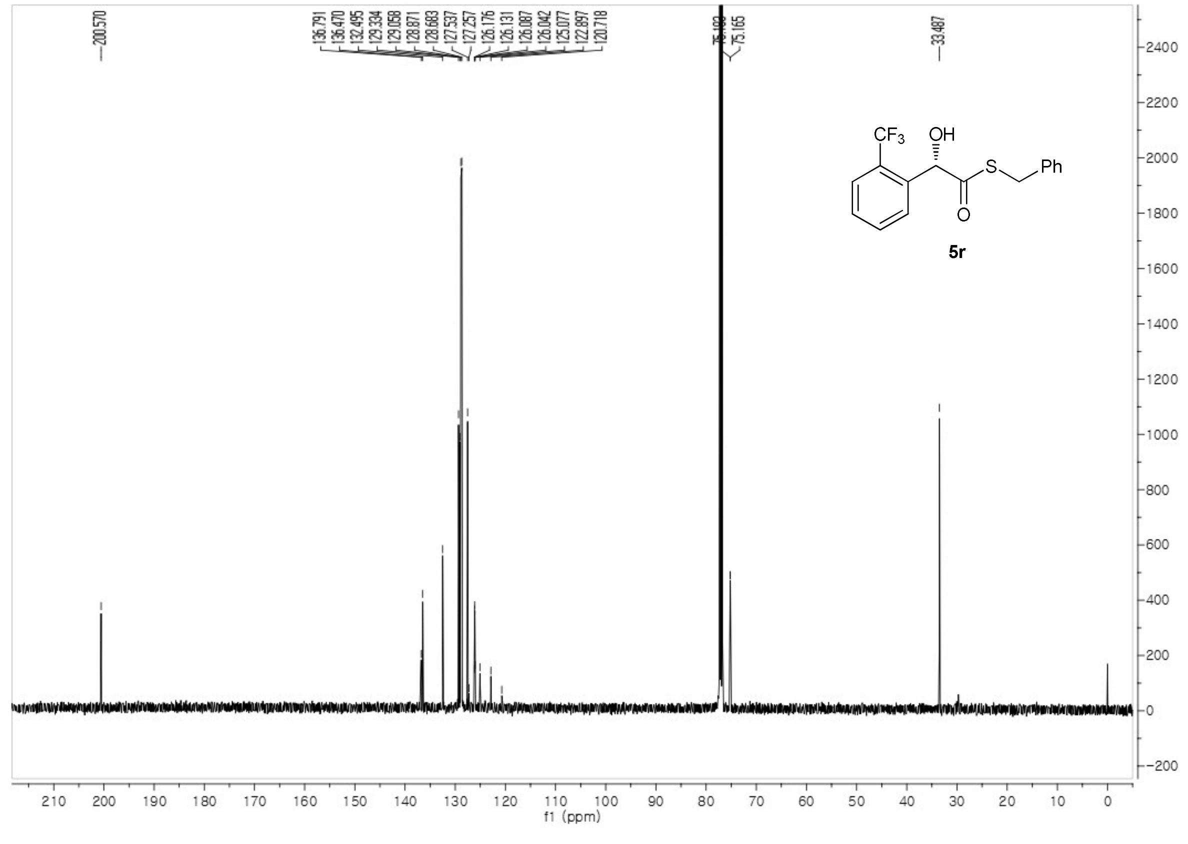

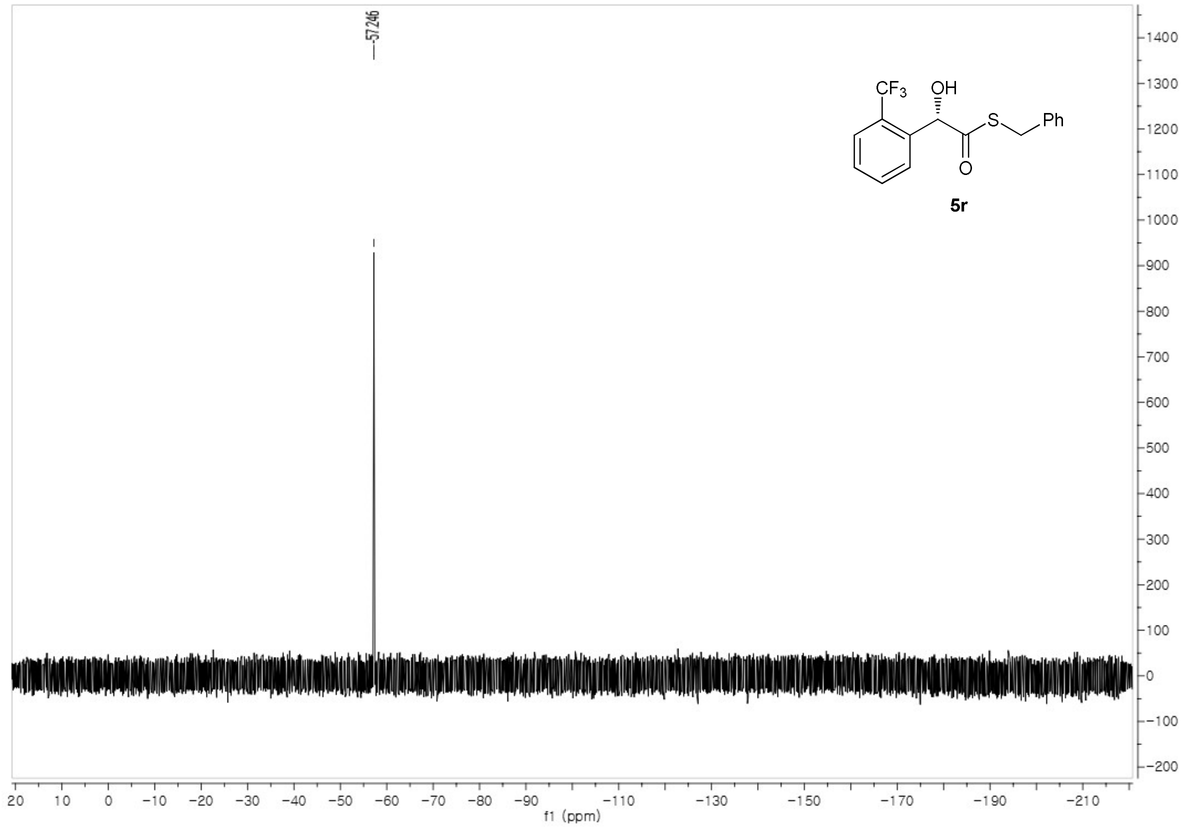

-57.246
CF3
OH
S
Ph
O
5r
f1 (ppm)

Supplementary Figure 35. ^{1}H, ^{13}C and ^{19}F NMR spectra of compound **5s**.

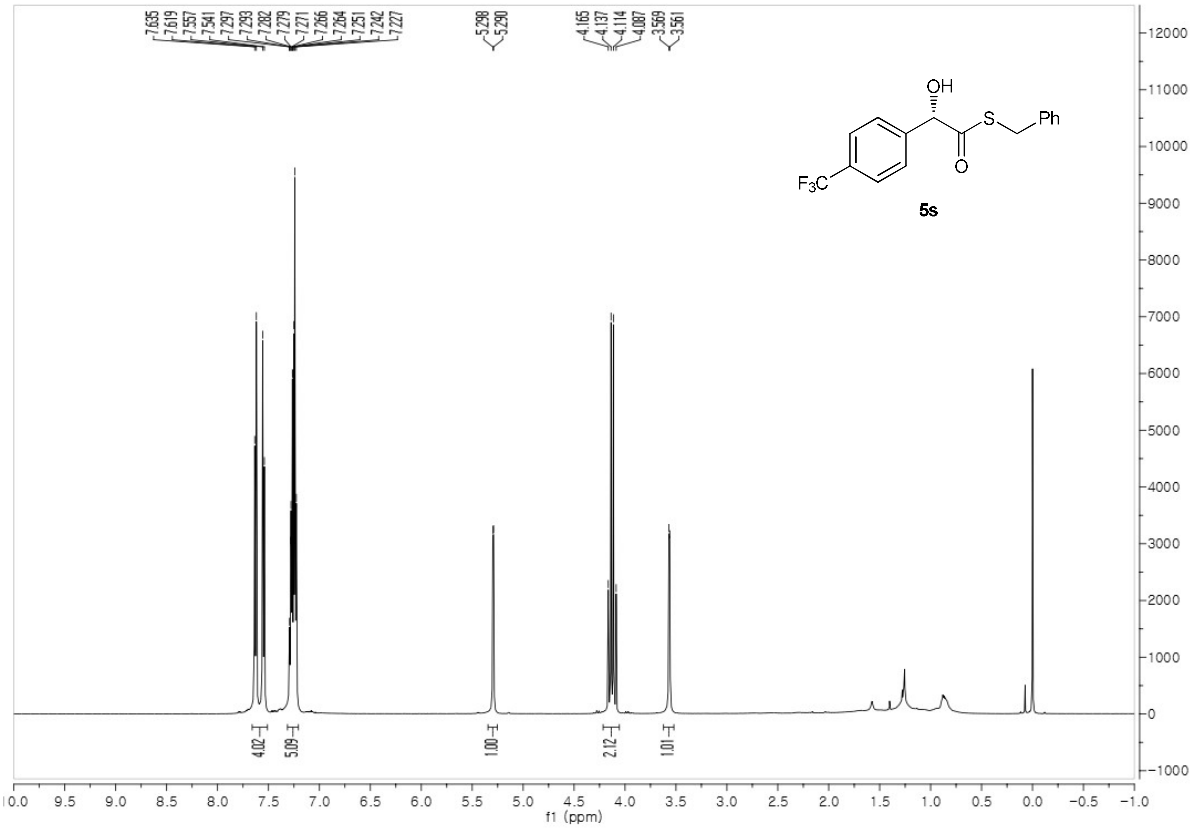

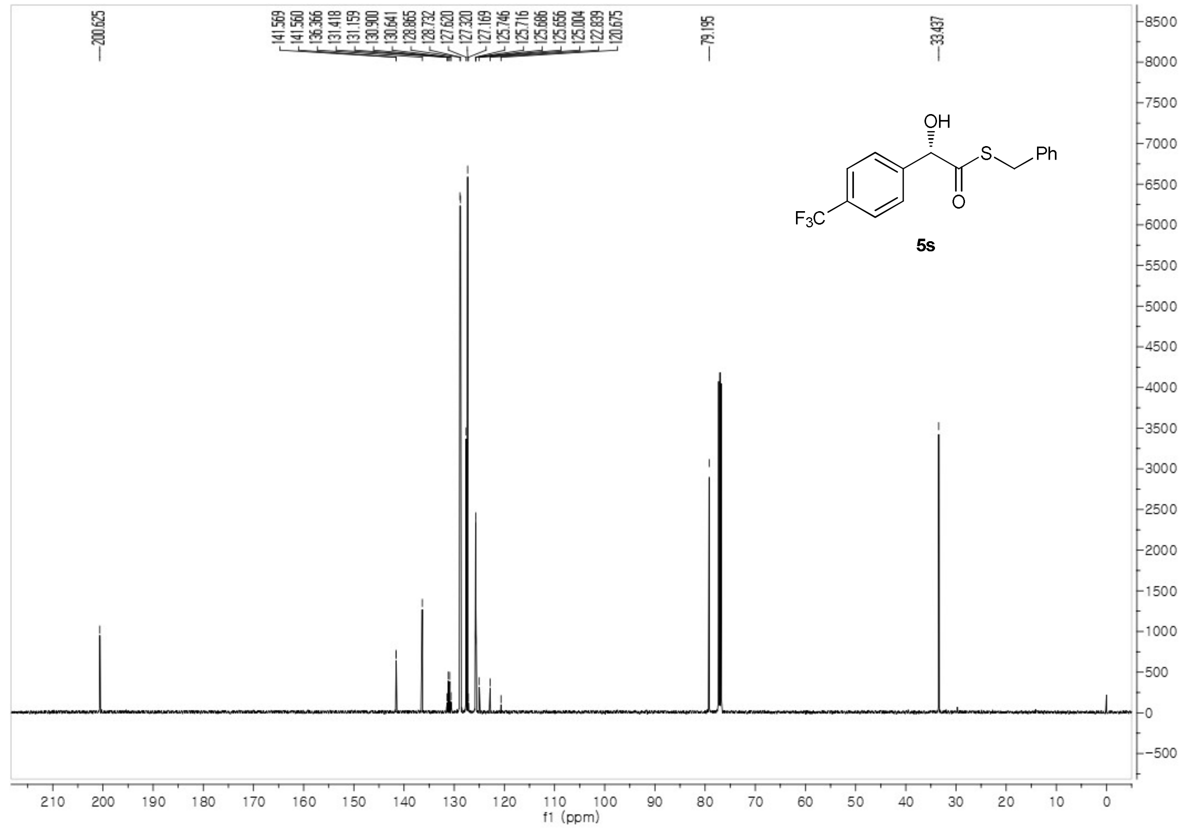

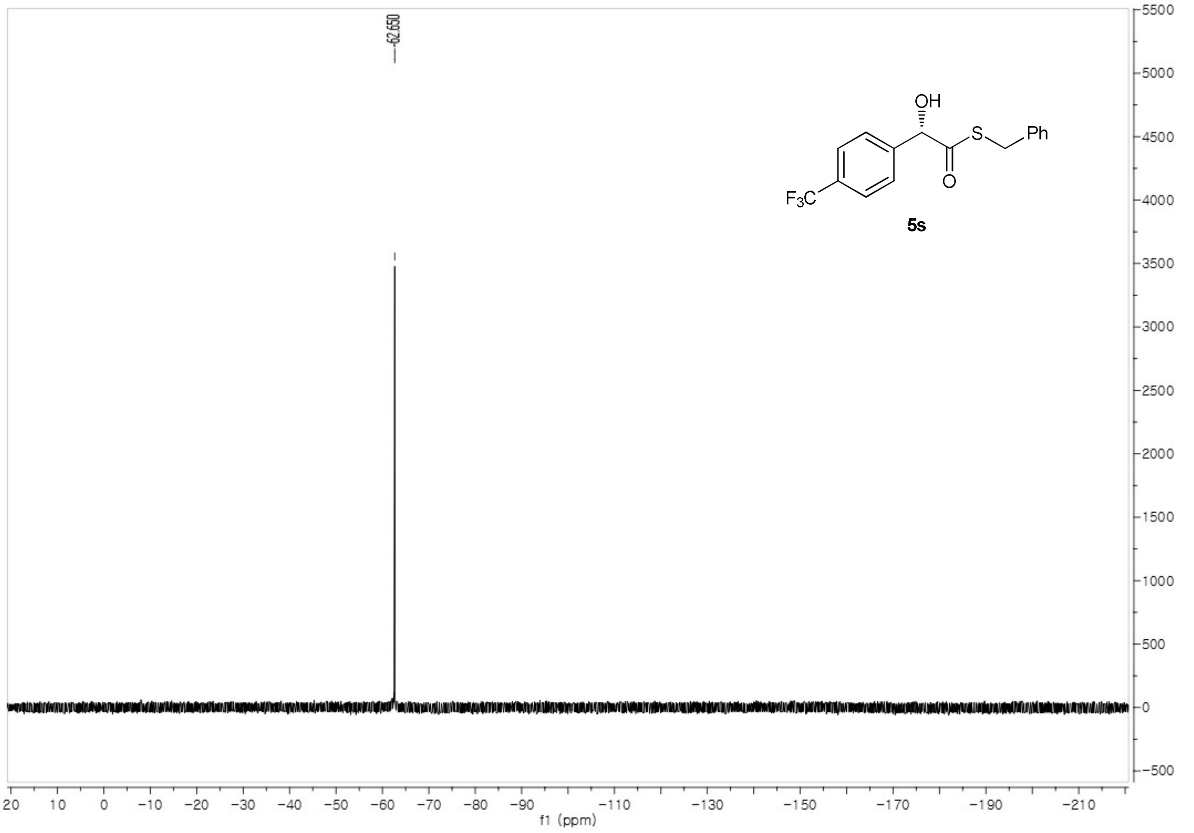

-62.650
OH
S
Ph
O
F3C
5s
f1 (ppm)

Supplementary Figure 36. ^{1}H, ^{13}C and ^{19}F NMR spectra of compound **5t**.

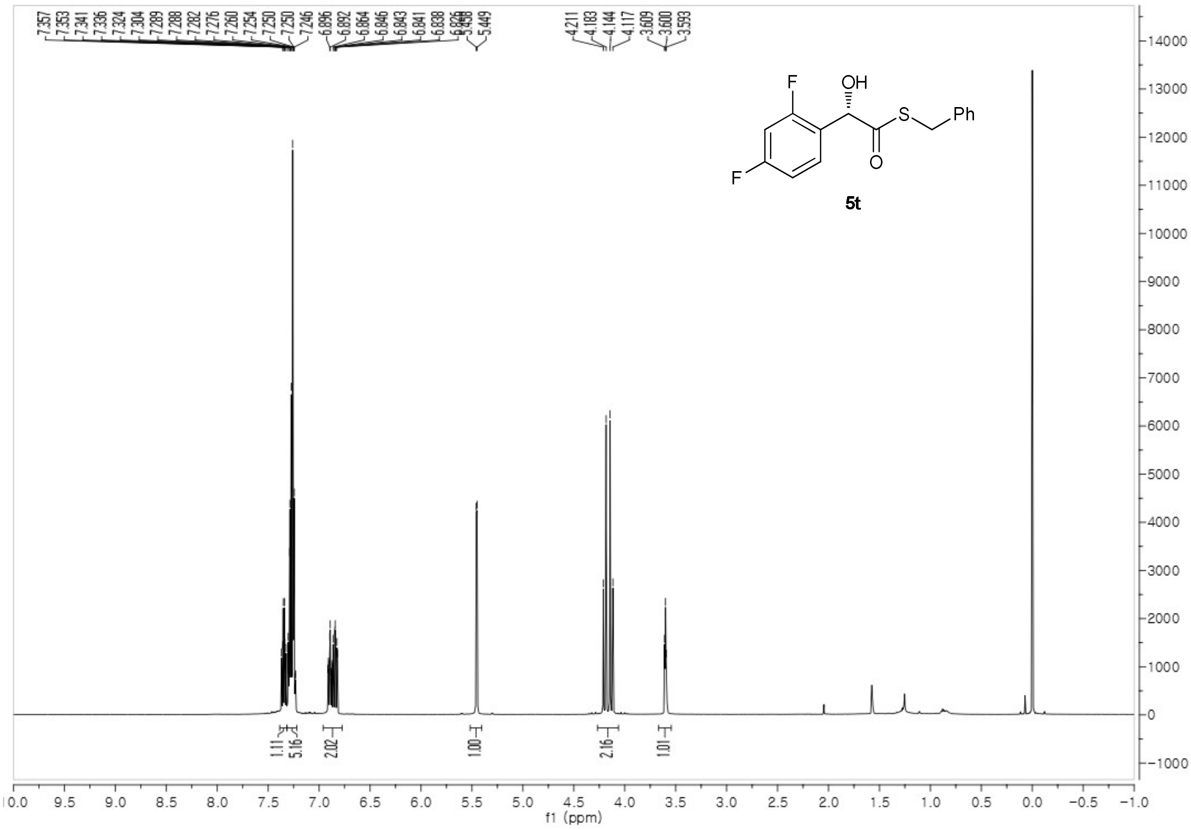

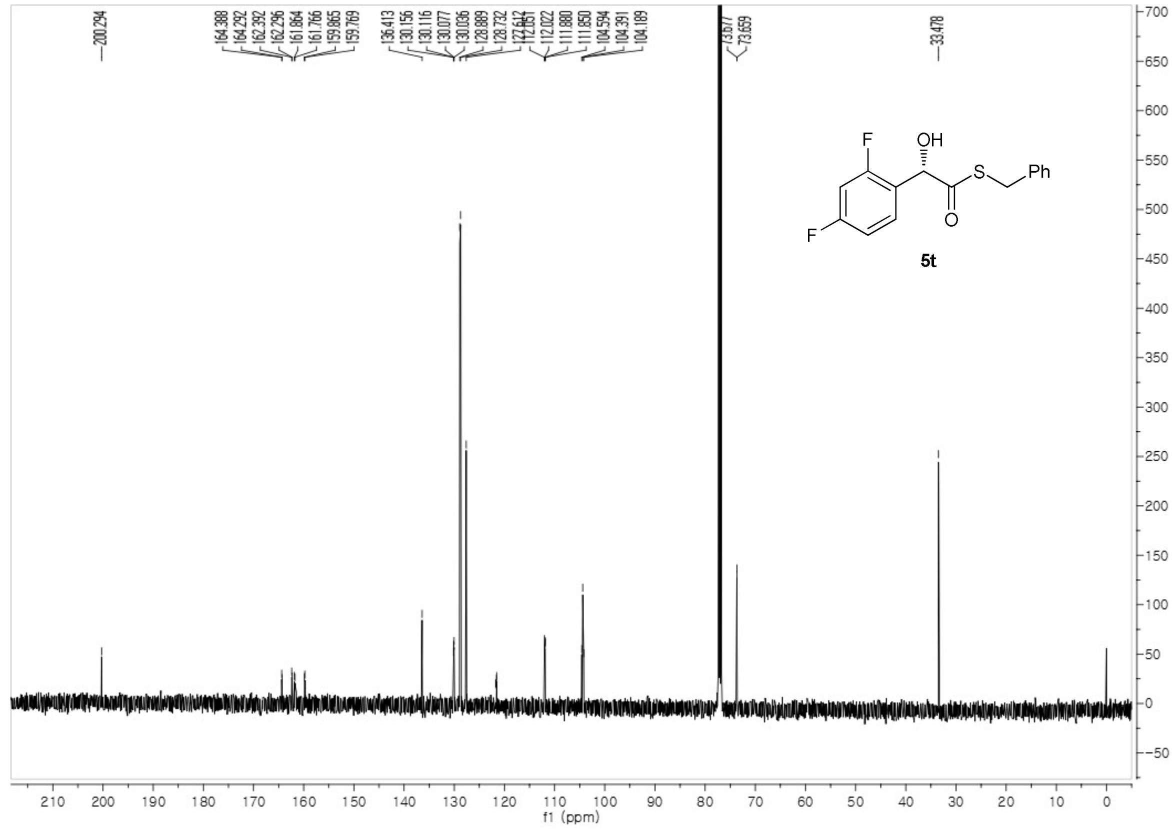

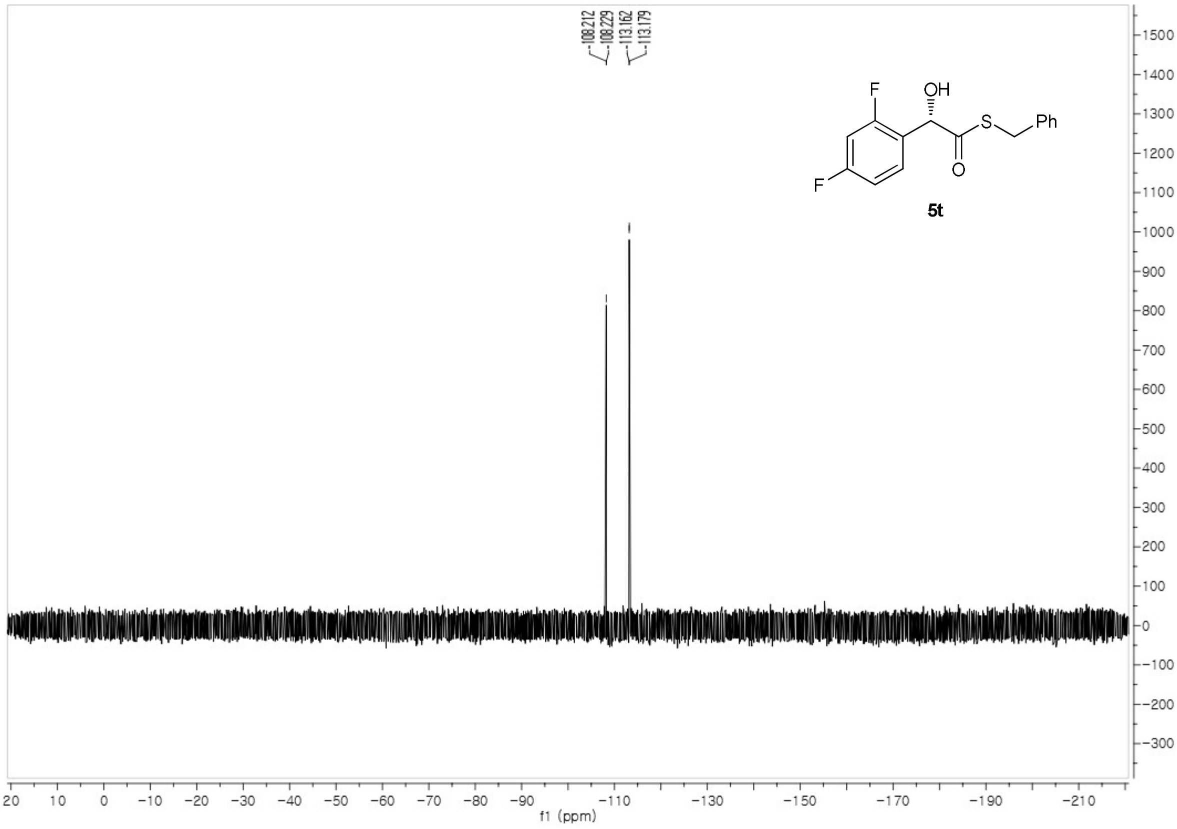
-108.212
-108.229
-113.162
-113.179
F
OH
S
Ph
F
O
5t
f1 (ppm)

Supplementary Figure 37. ^{1}H, ^{13}C and ^{19}F NMR spectra of compound **5u**.

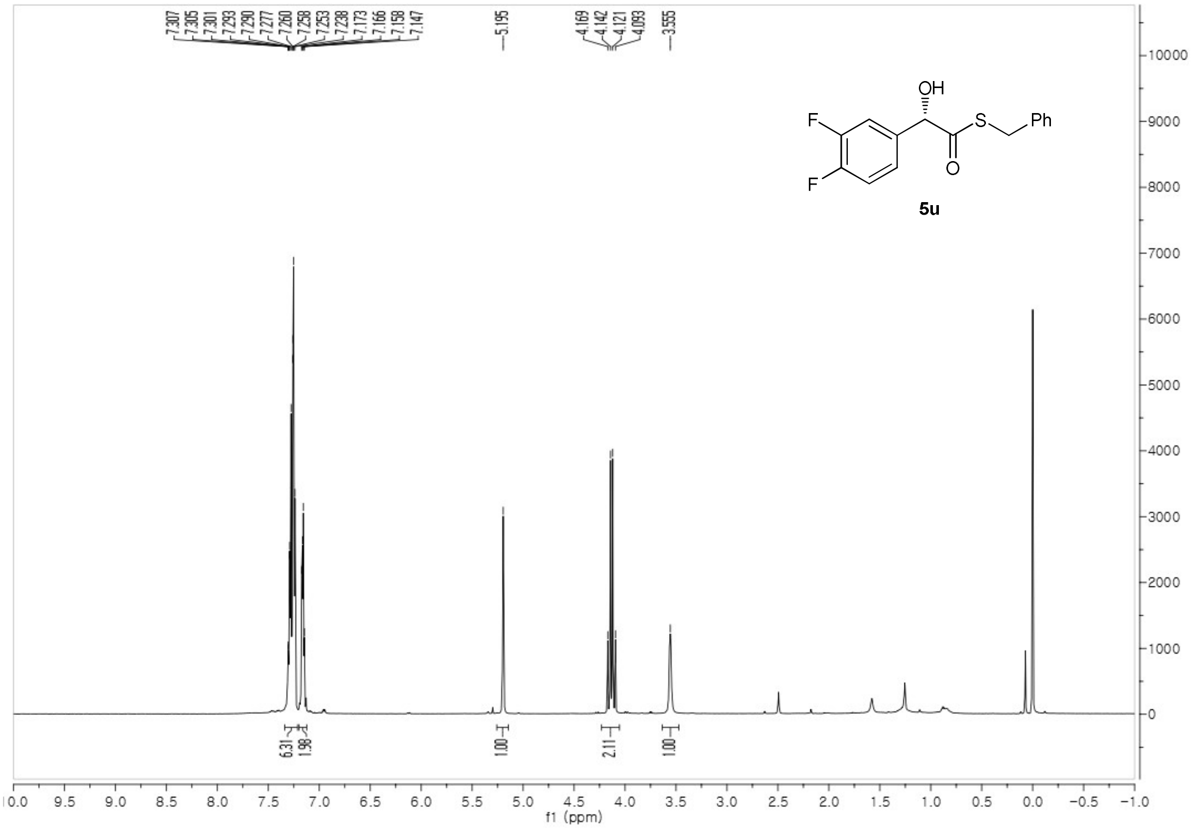

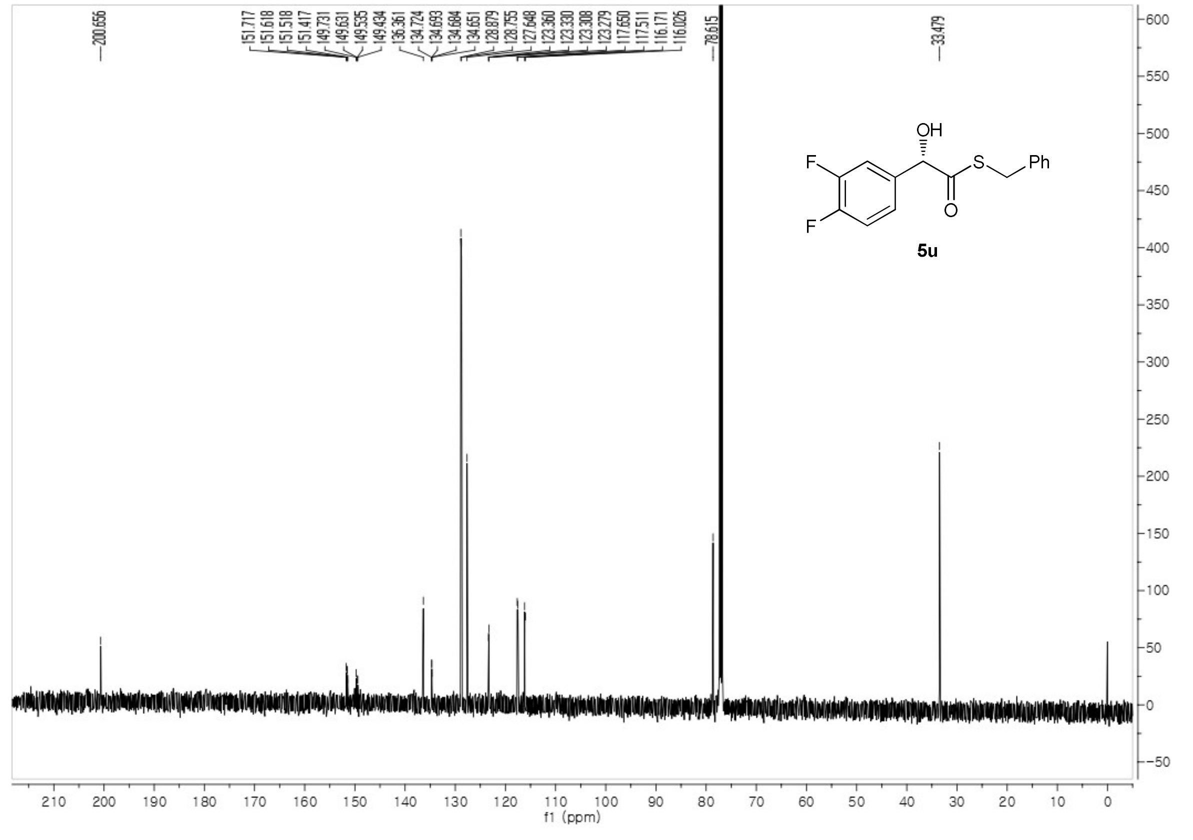

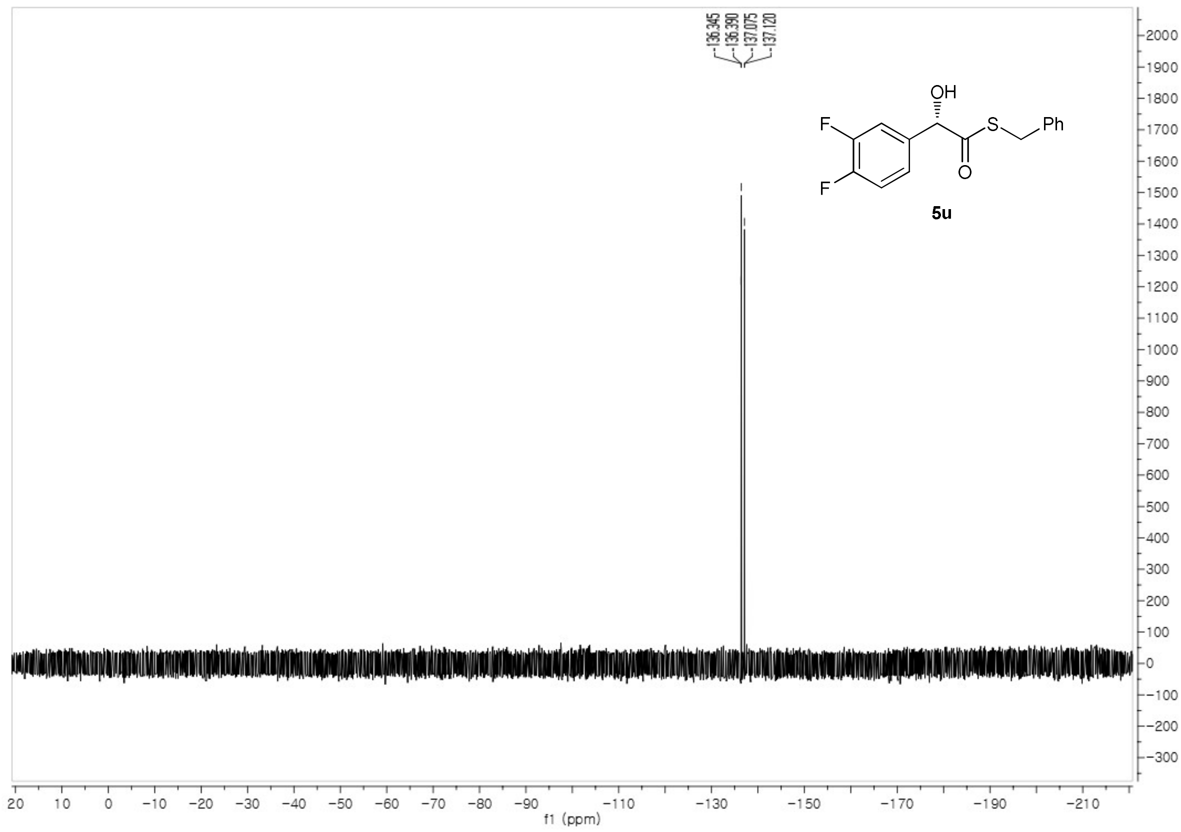

-136.345
-136.390
-137.075
-137.120
OH
F
F
S
Ph
O
5u
f1 (ppm)

Supplementary Figure 38. ^{1}H, ^{13}C and ^{19}F NMR spectra of compound **5v**.

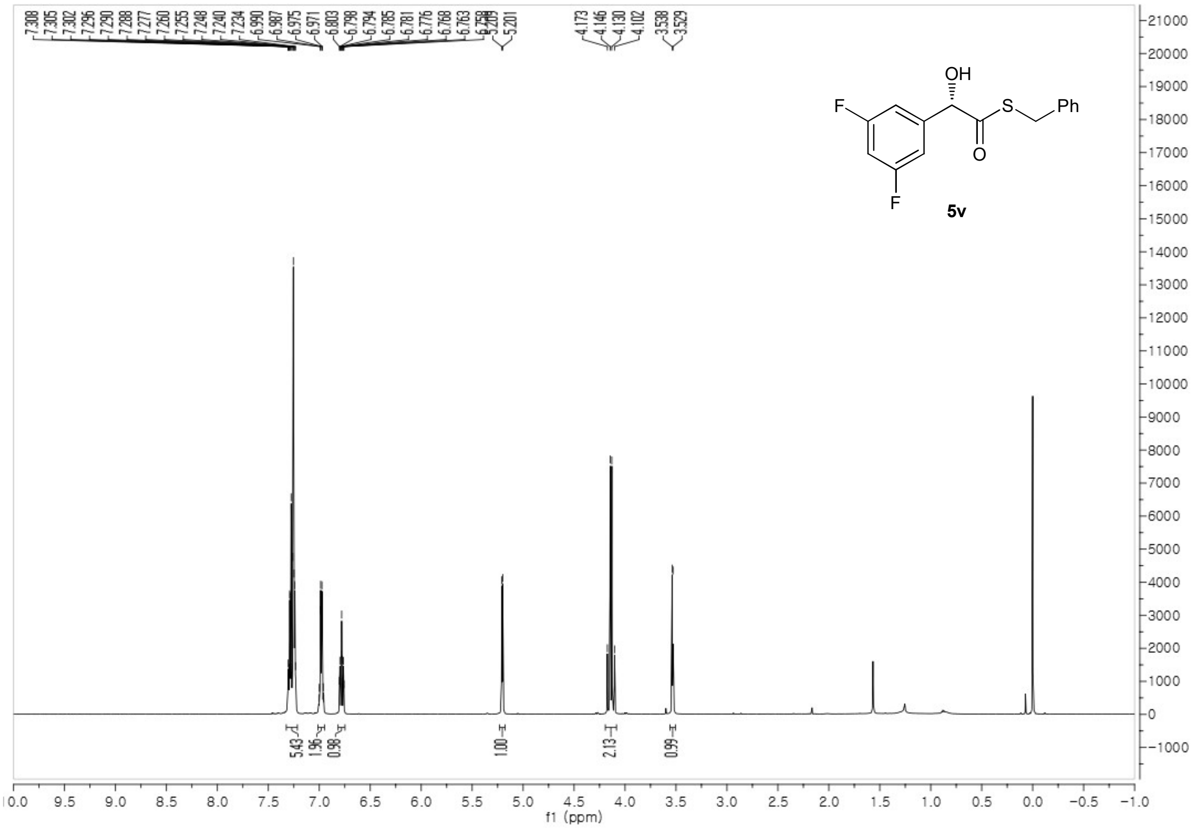

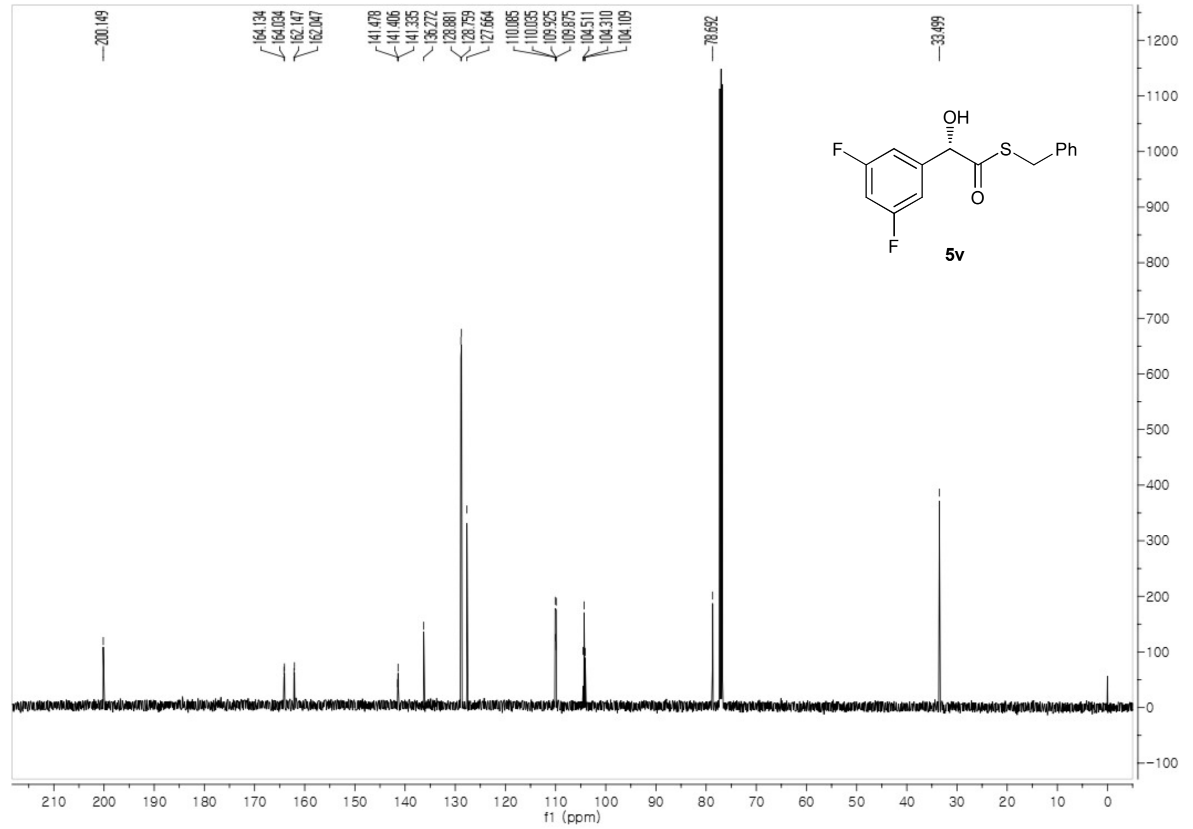

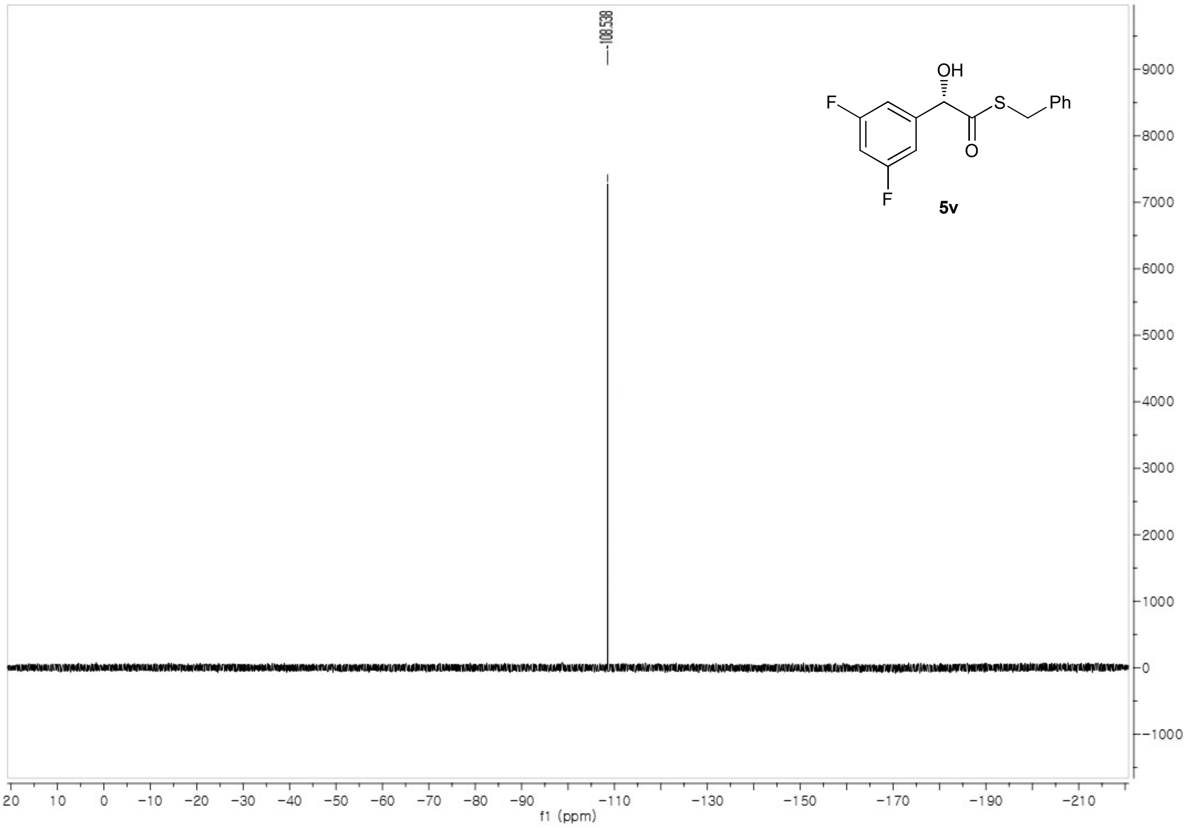
—108.538
OH
F
S
Ph
O
F
5v
9000
8000
7000
6000
5000
4000
3000
2000
1000
0
−1000
20
10
0
−10
−20
−30
−40
−50
−60
−70
−80
−90
−110
−130
−150
−170
−190
−210
f1 (ppm)

Supplementary Figure 39. ^{1}H and ^{13}C NMR spectra of compound **5w**.

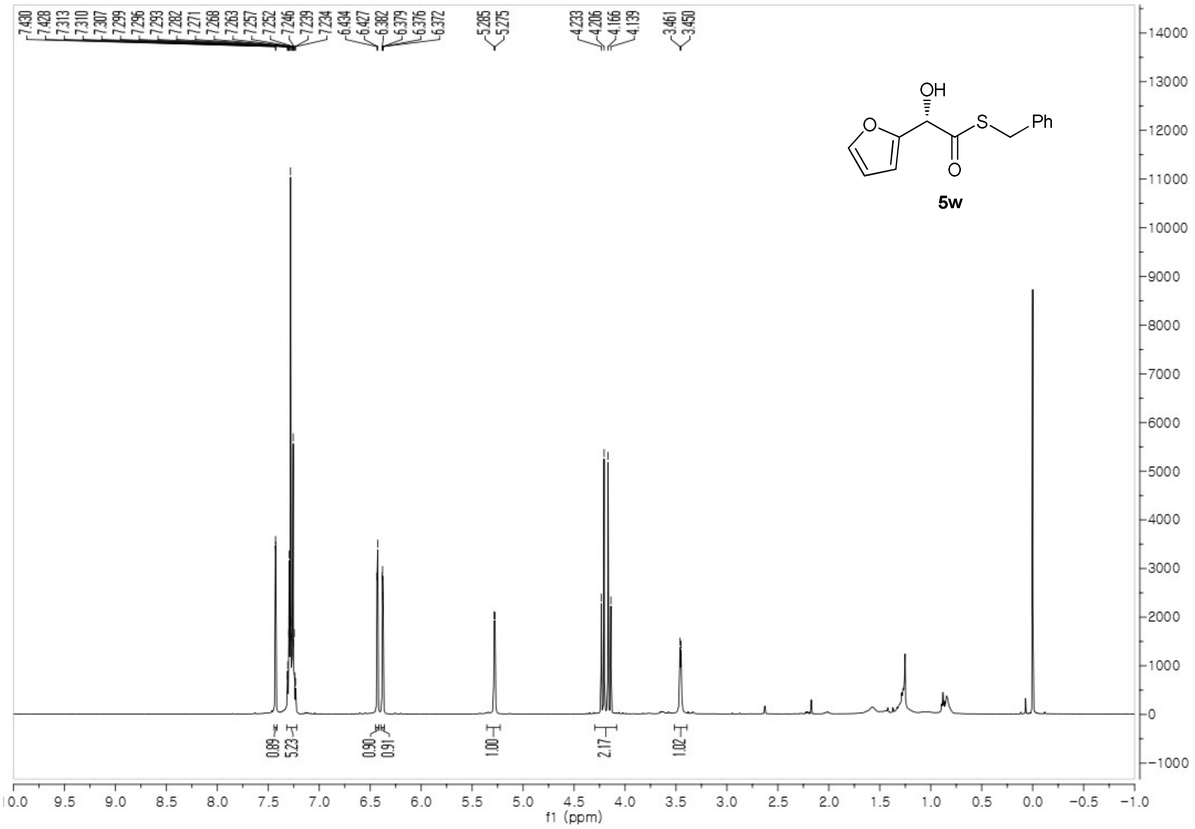

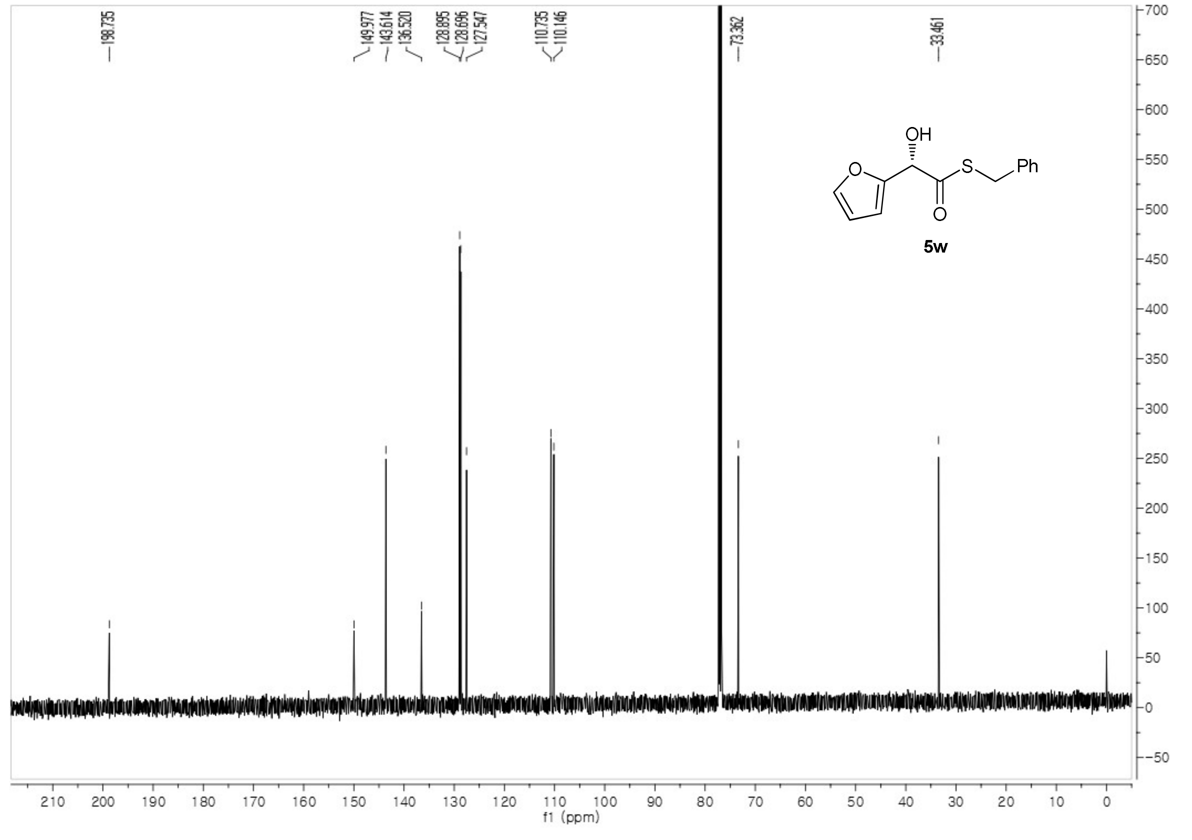

Supplementary Figure 40. ^{1}H and ^{13}C NMR spectra of compound **5x**.

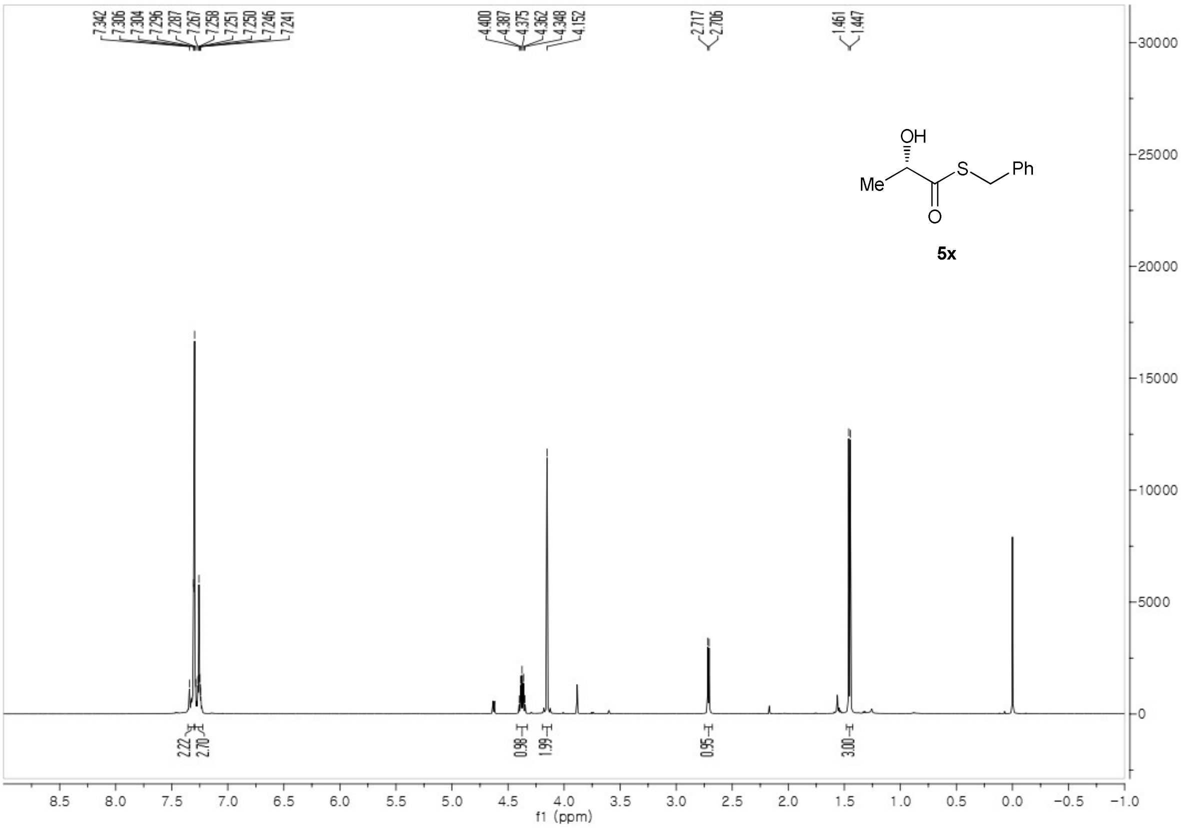

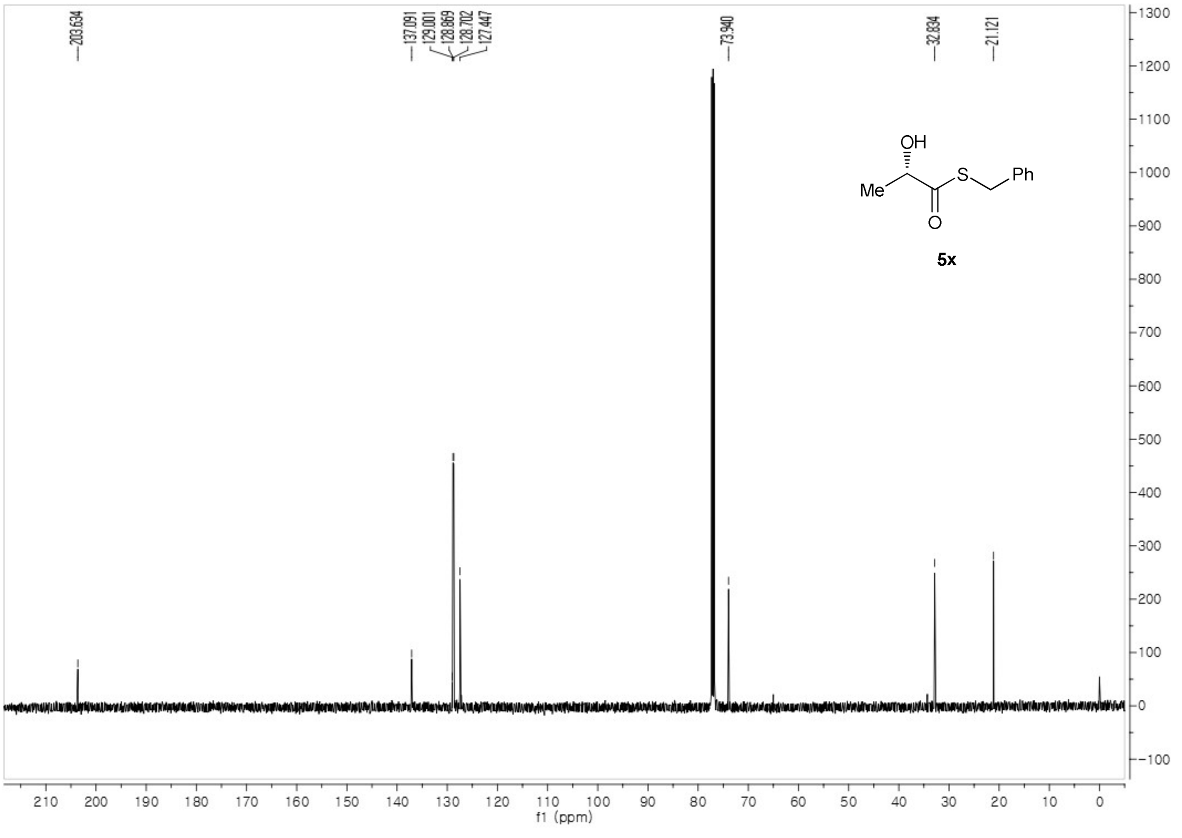

Supplementary Figure 41. ^{1}H and ^{13}C NMR spectra of compound **5y**.

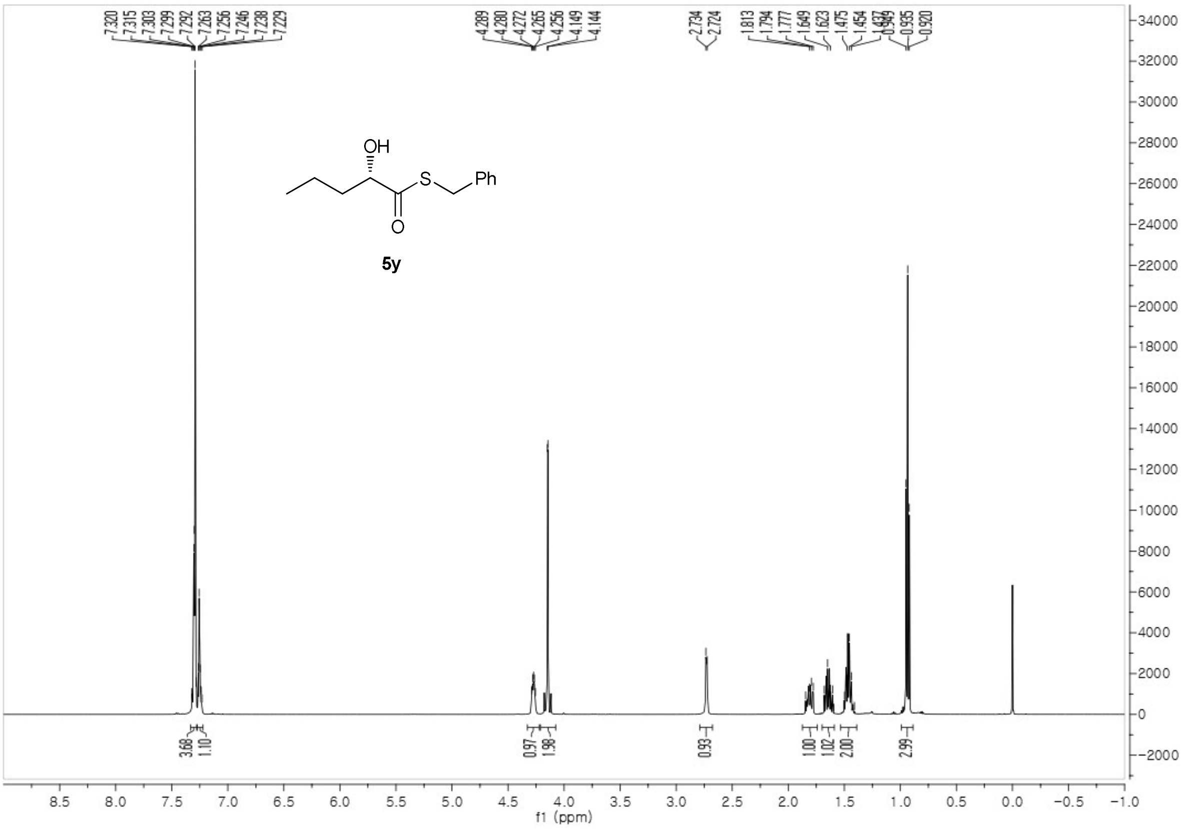

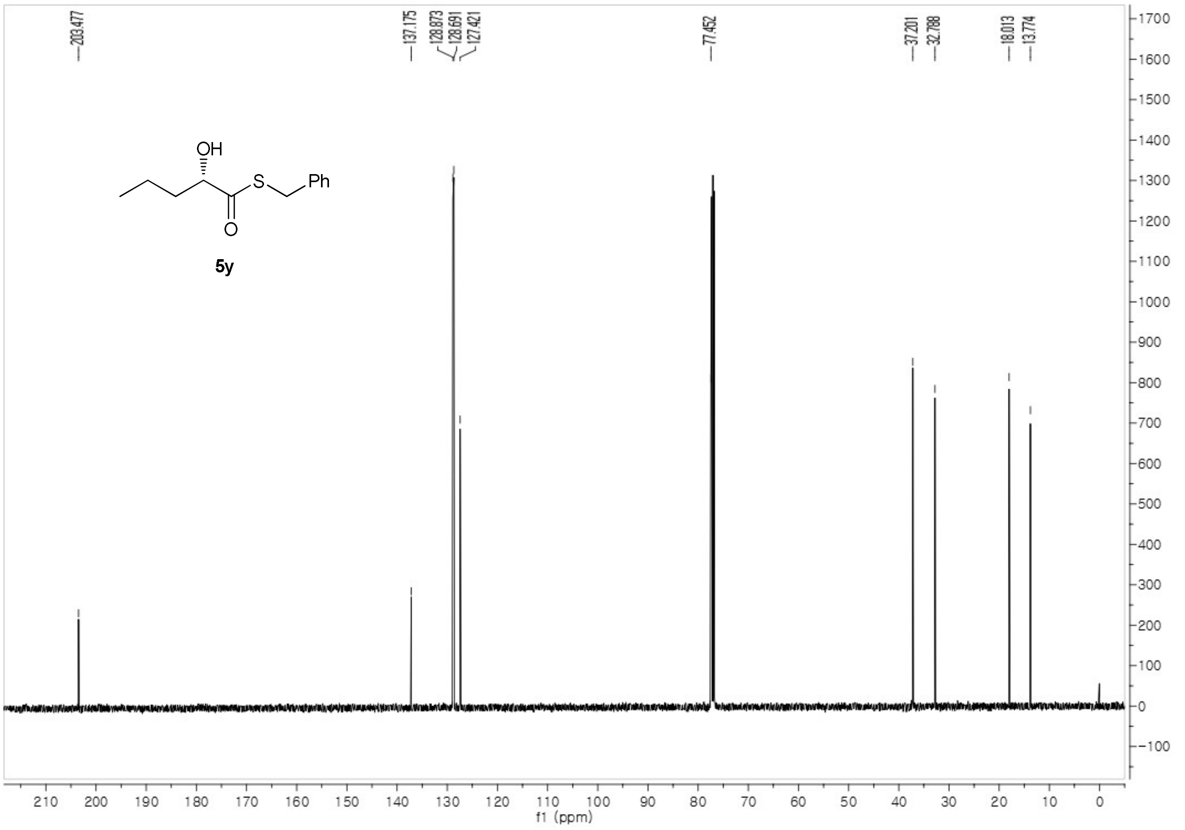

Supplementary Figure 42. ^{1}H and ^{13}C NMR spectra of compound **5z**.

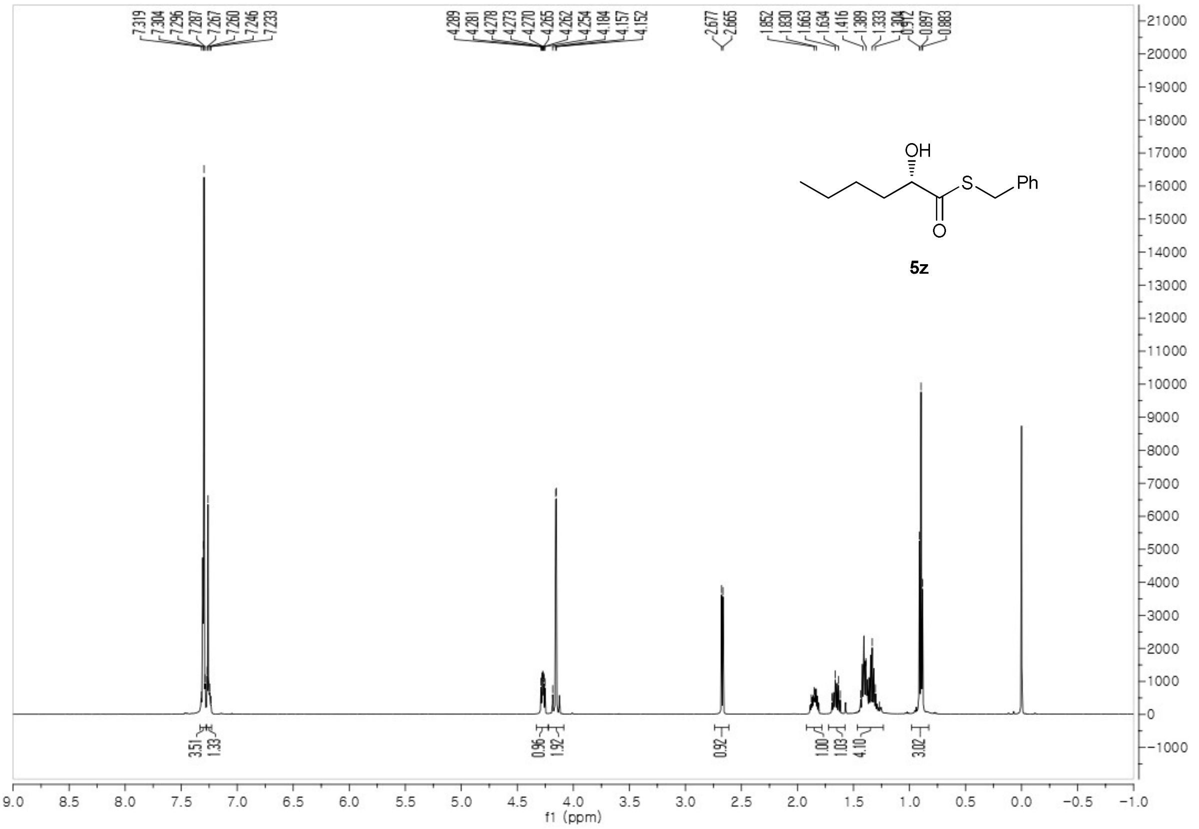

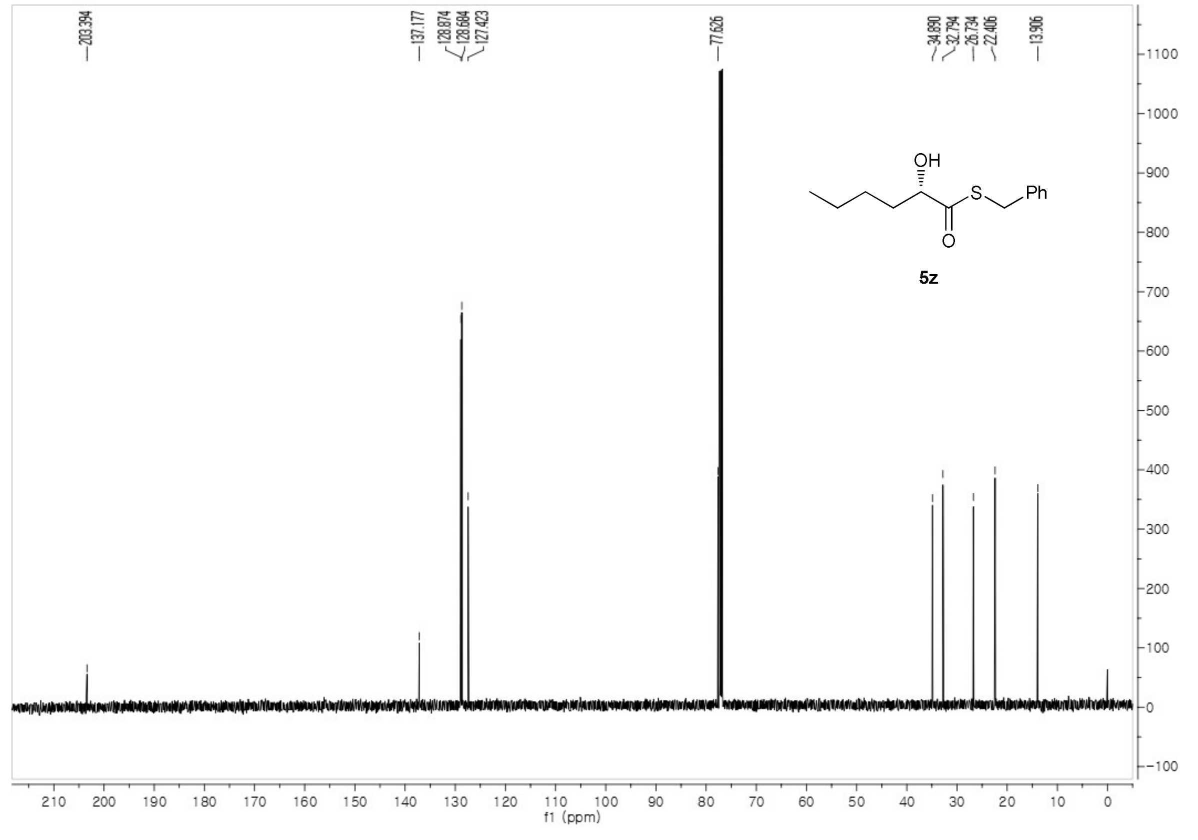

Supplementary Figure 43. ^{1}H, ^{13}C and ^{19}F NMR spectra of compound **7**.

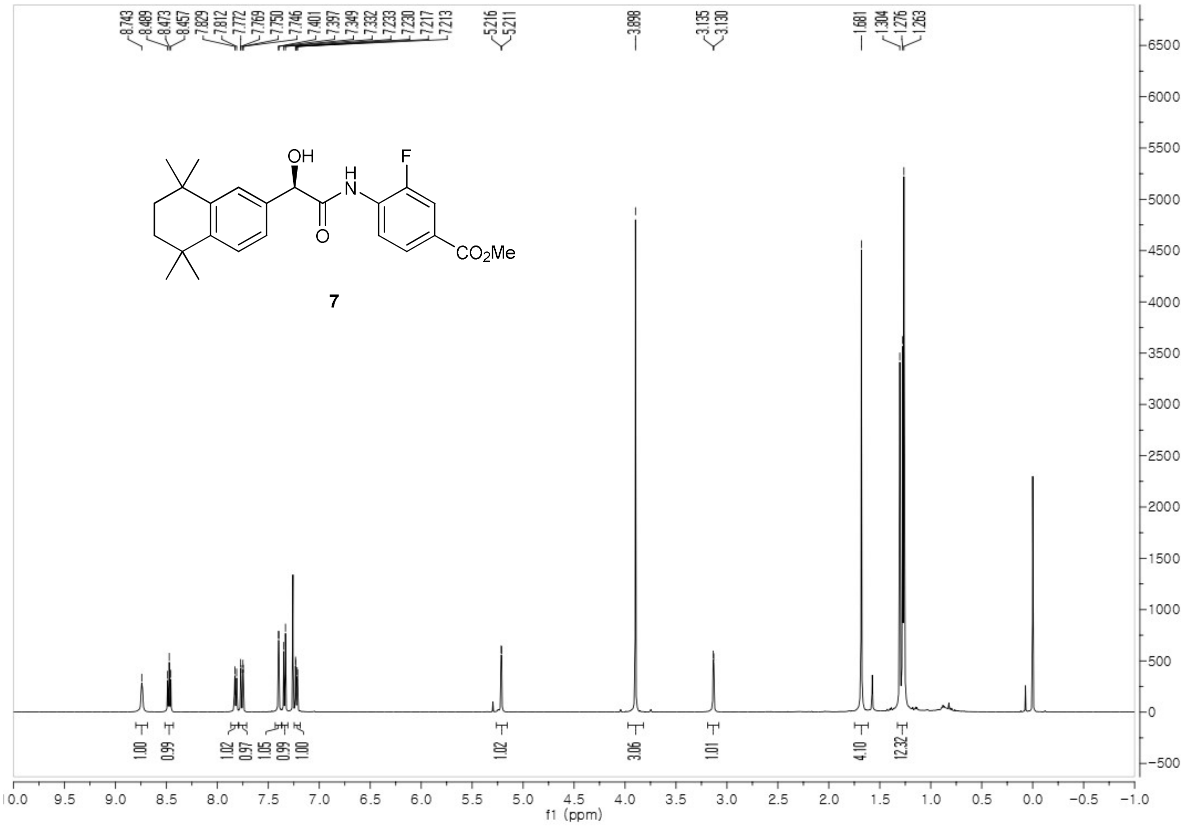

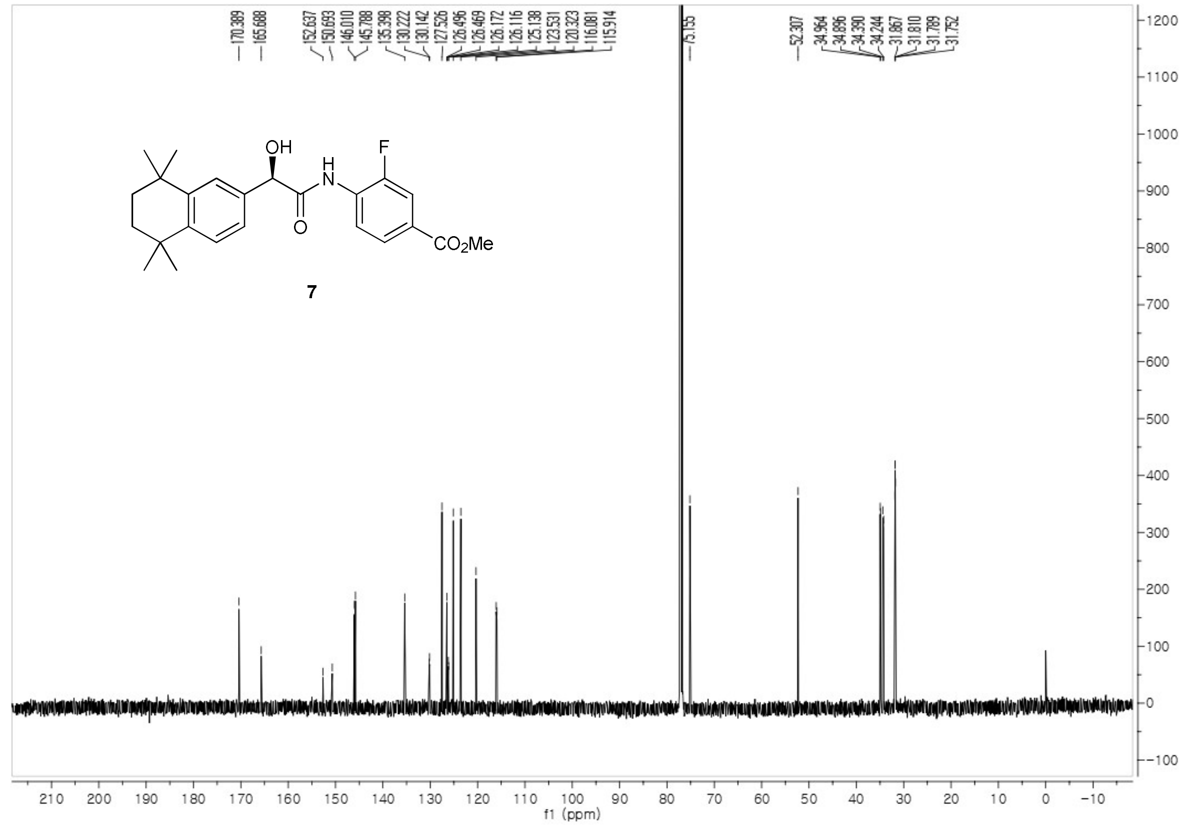

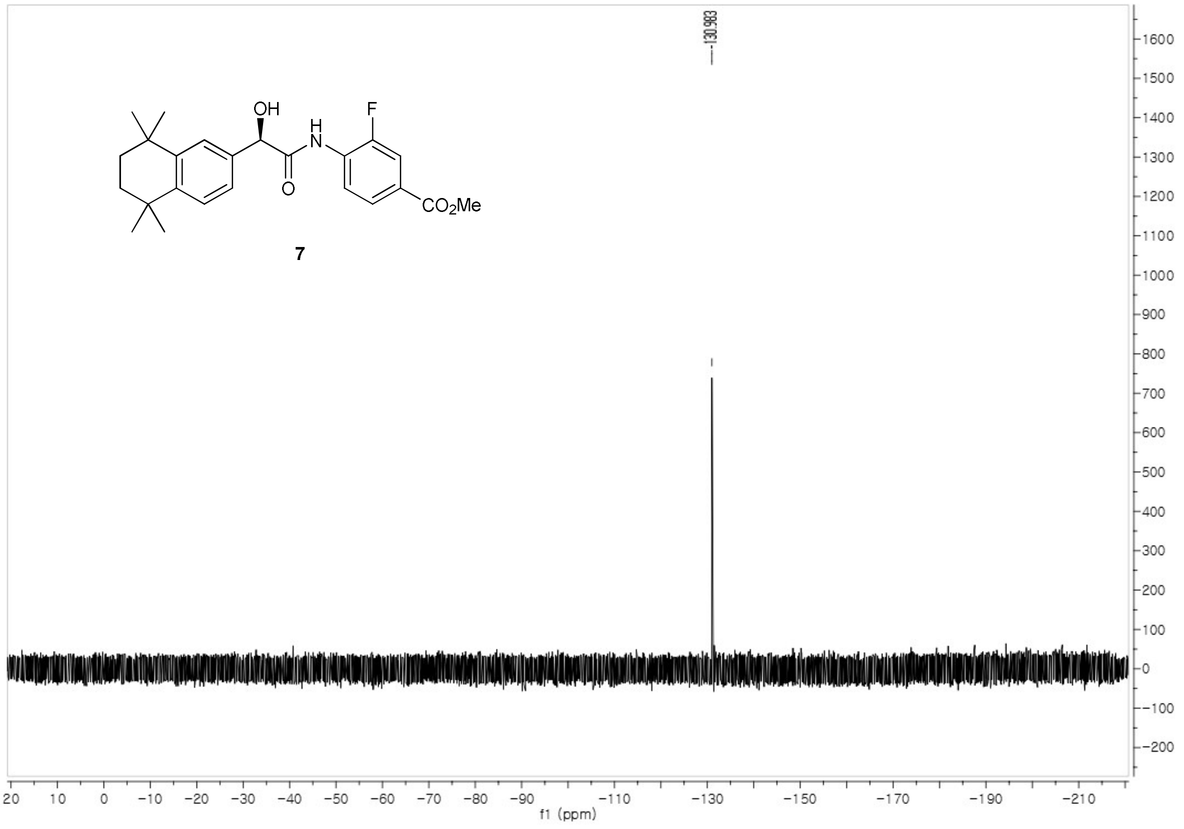
-130.983
OH
H
N
F
O
CO2Me
7
f1 (ppm)

Supplementary Figure 44. ^{1}H, ^{13}C and ^{19}F NMR spectra of compound **9**.

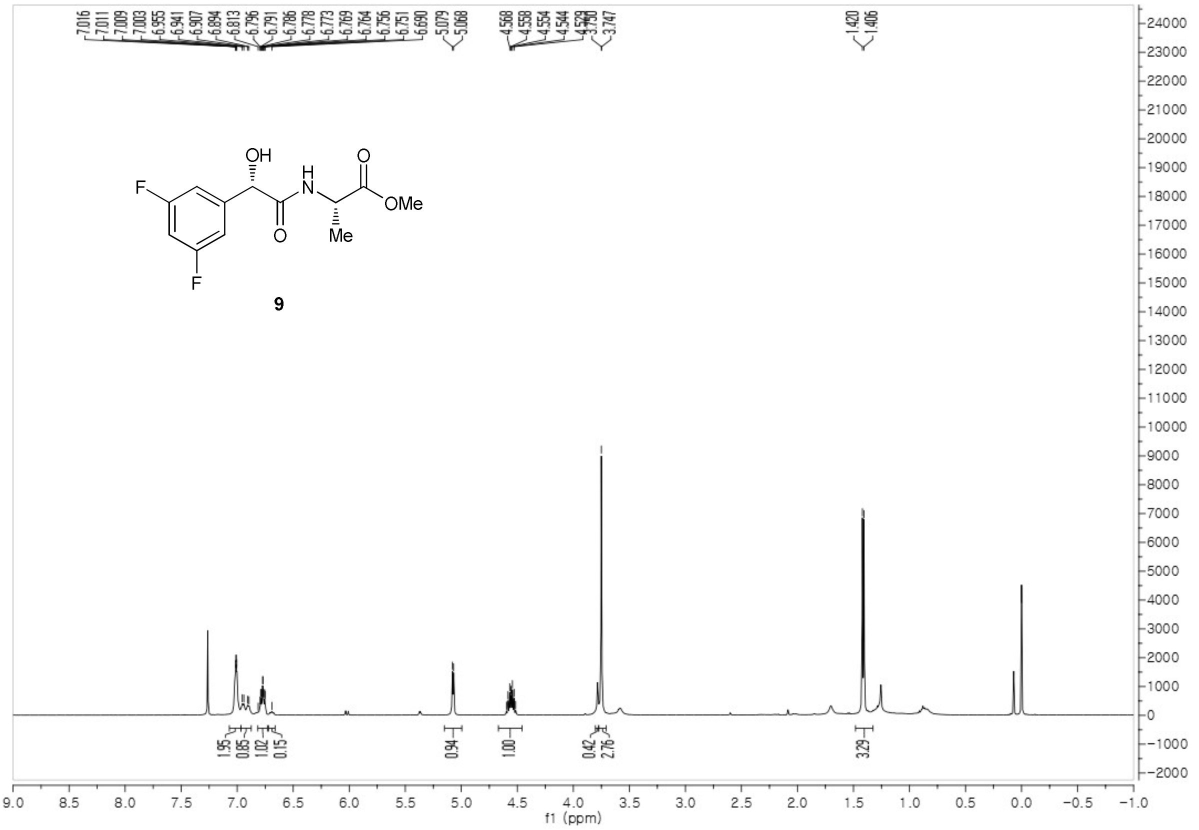

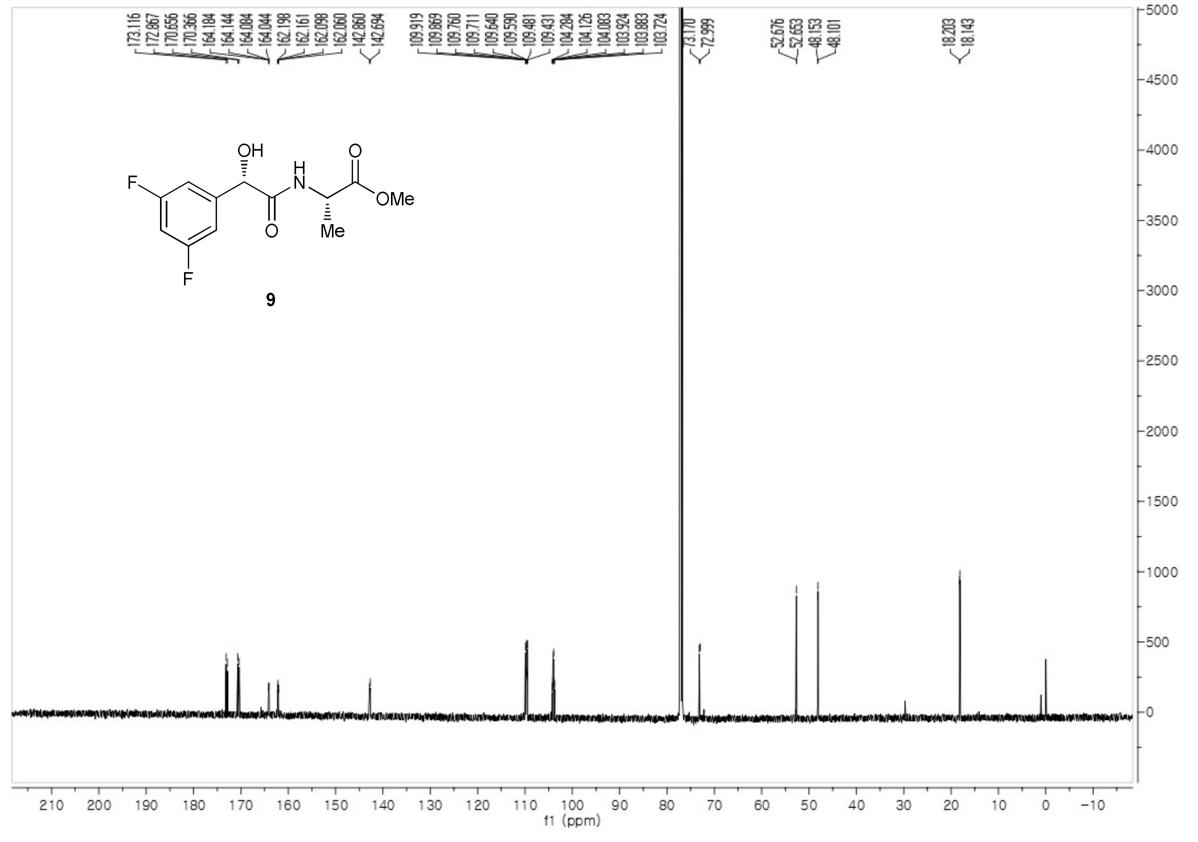

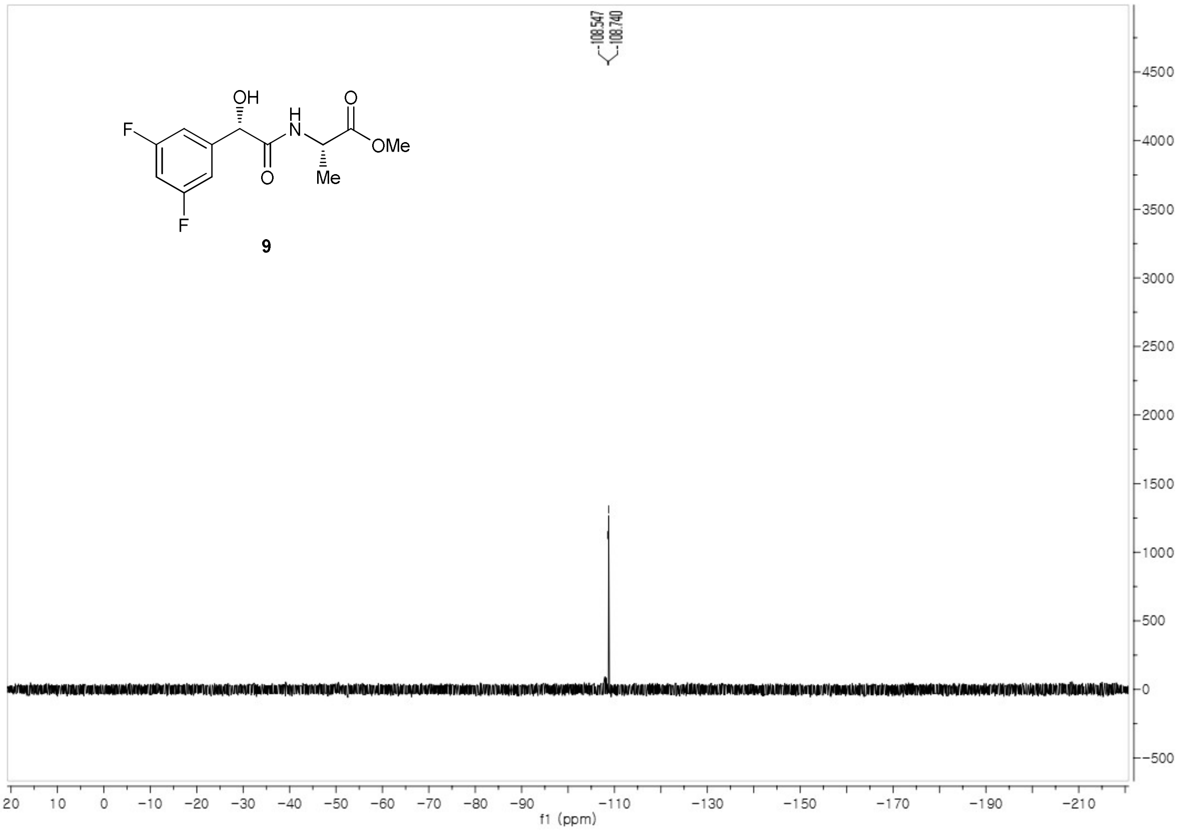

OH
H
N
O
OMe
F
F
O
Me
9
-108.547
-108.740
4500
4000
3500
3000
2500
2000
1500
1000
500
0
-500
20 10 0 -10 -20 -30 -40 -50 -60 -70 -80 -90 -110 -130 -150 -170 -190 -210
f1 (ppm)

Supplementary Figure 45. ^{1}H, ^{13}C and ^{19}F NMR spectra of compound **LY 411575**.

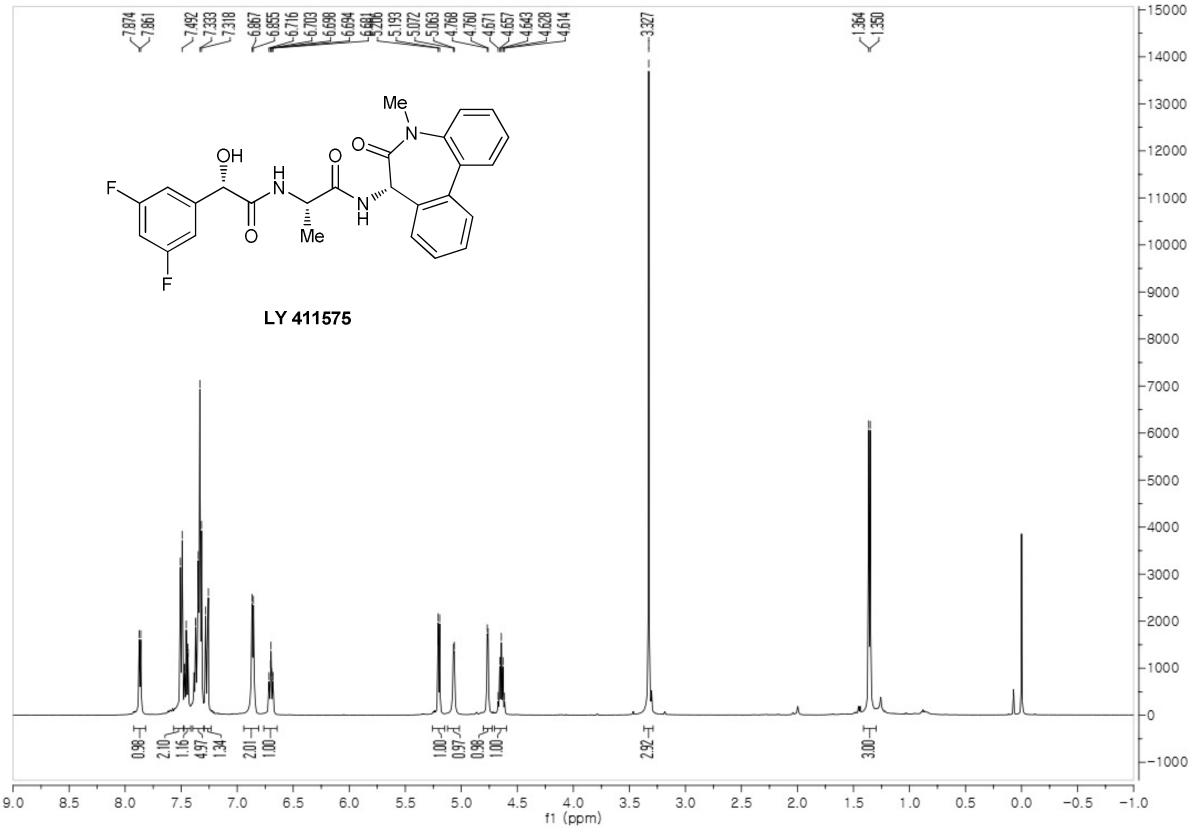

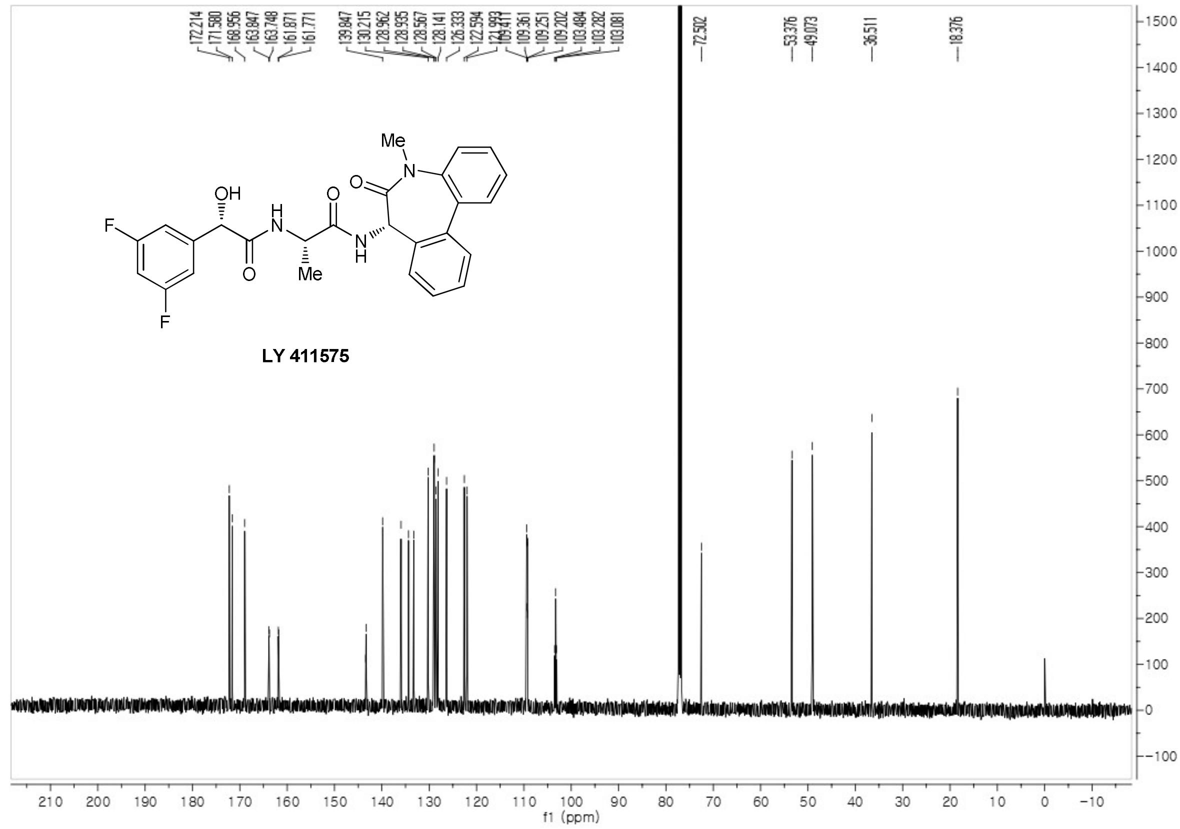

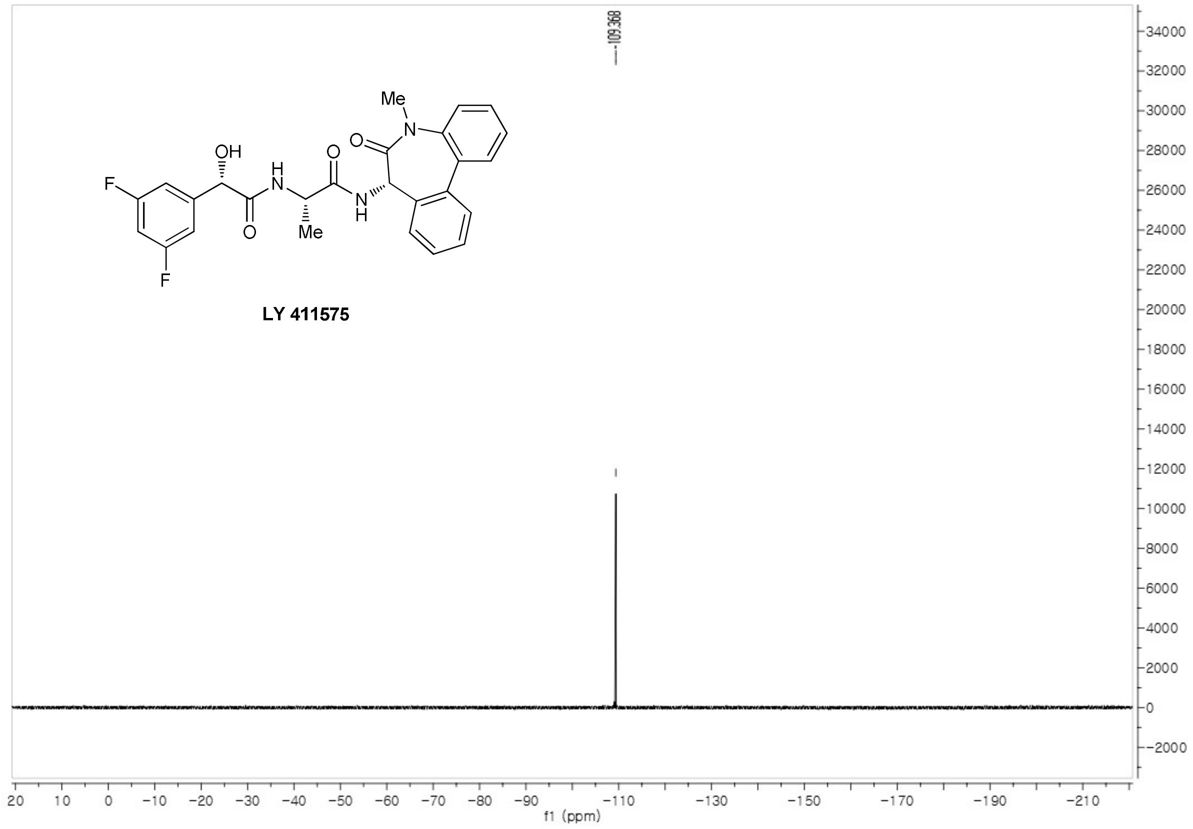

-109.368
Me
N
O
OH
H
N
F
F
O
Me
N
H
LY 411575
34000
32000
30000
28000
26000
24000
22000
20000
18000
16000
14000
12000
10000
8000
6000
4000
2000
0
-2000
20
10
0
-10
-20
-30
-40
-50
-60
-70
-80
-90
-110
-130
-150
-170
-190
-210
f1 (ppm)

Supplementary Figure 46. ^{1}H and ^{13}C NMR spectra of (*S*)-Lactam-*L*-Boc-alanine amide.

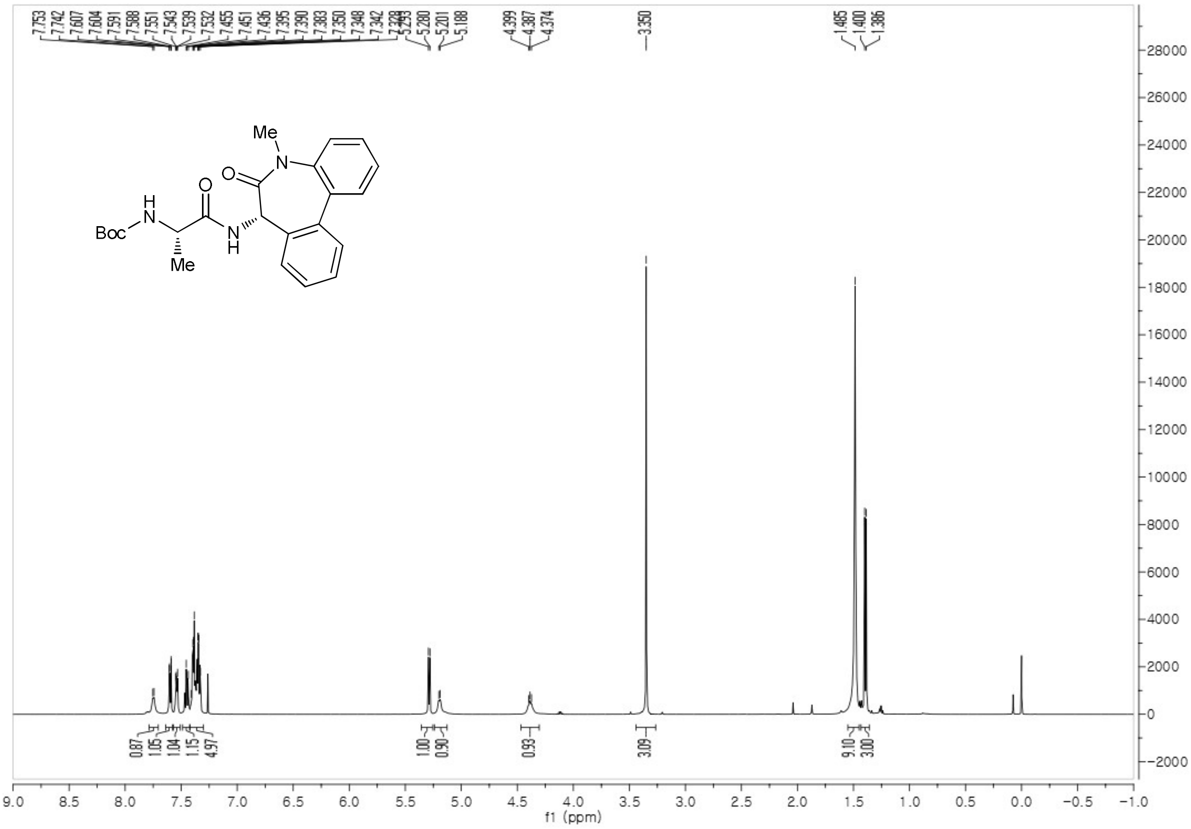

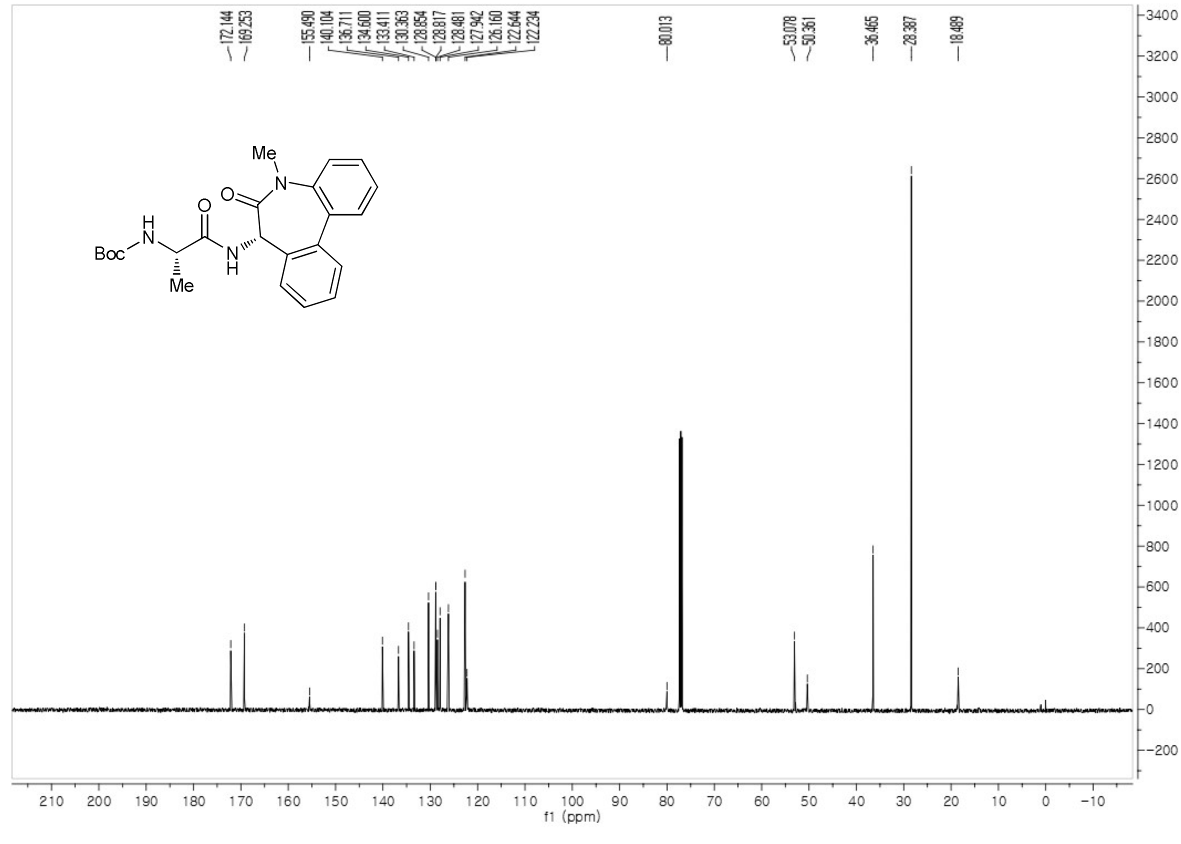

Supplementary Figure 47. HPLC spectra of compound **5a**.

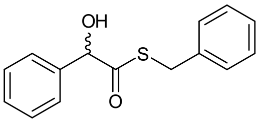

rac-**5a**

Chiralpak AD-H (n-hexane/i-PrOH = 96/4, flow rate: 1.0 mL/min, wave length = 220 nm)

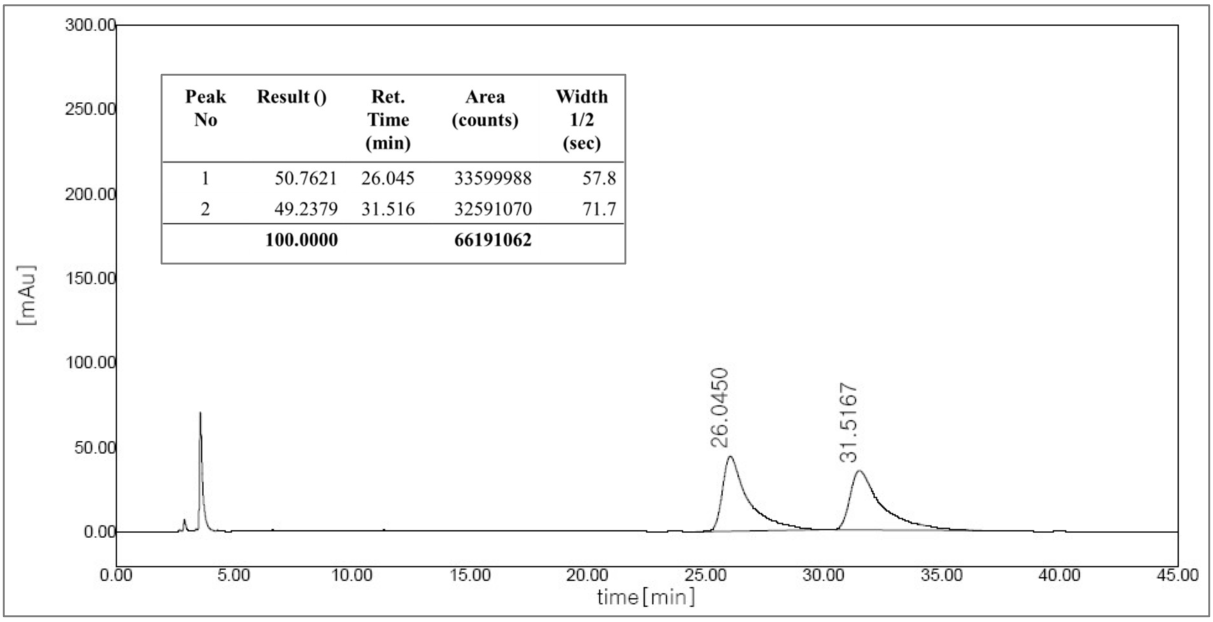

Peak No	Result ()	Ret. Time (min)	Area (counts)	Width 1/2 (sec)
1	50.7621	26.045	33599988	57.8
2	49.2379	31.516	32591070	71.7
	**100.0000**		**66191062**	

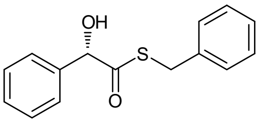

(*S*)-**5a**

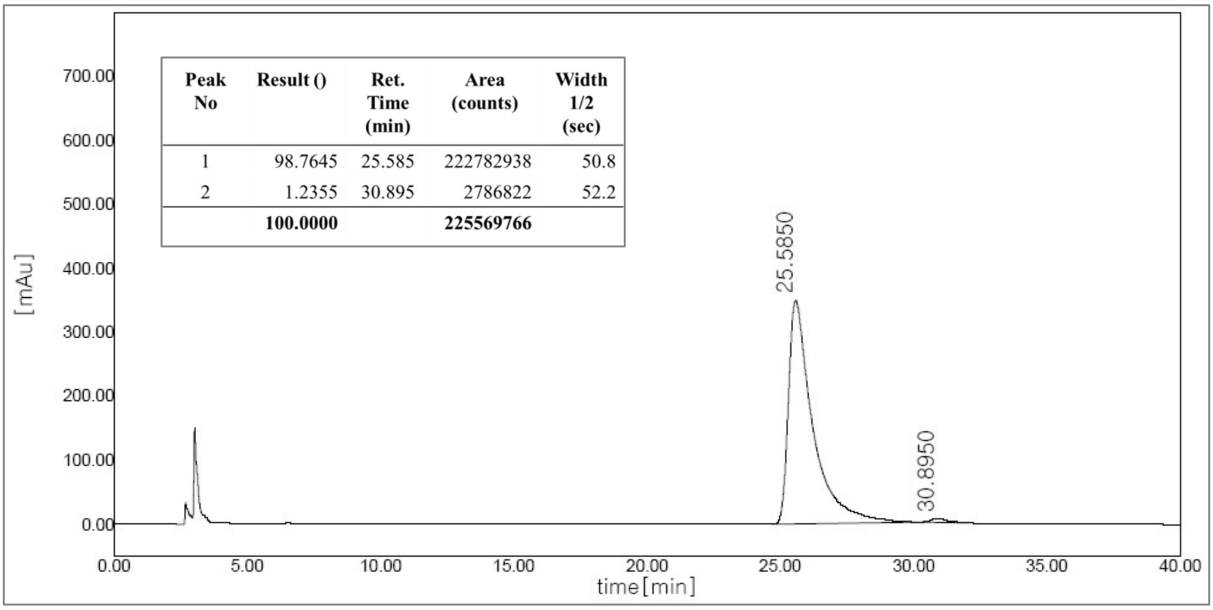

Peak No	Result ()	Ret. Time (min)	Area (counts)	Width 1/2 (sec)
1	98.7645	25.585	222782938	50.8
2	1.2355	30.895	2786822	52.2
	**100.0000**		**225569766**	

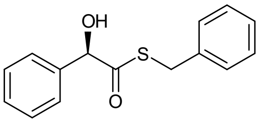

(*R*)-**5a**

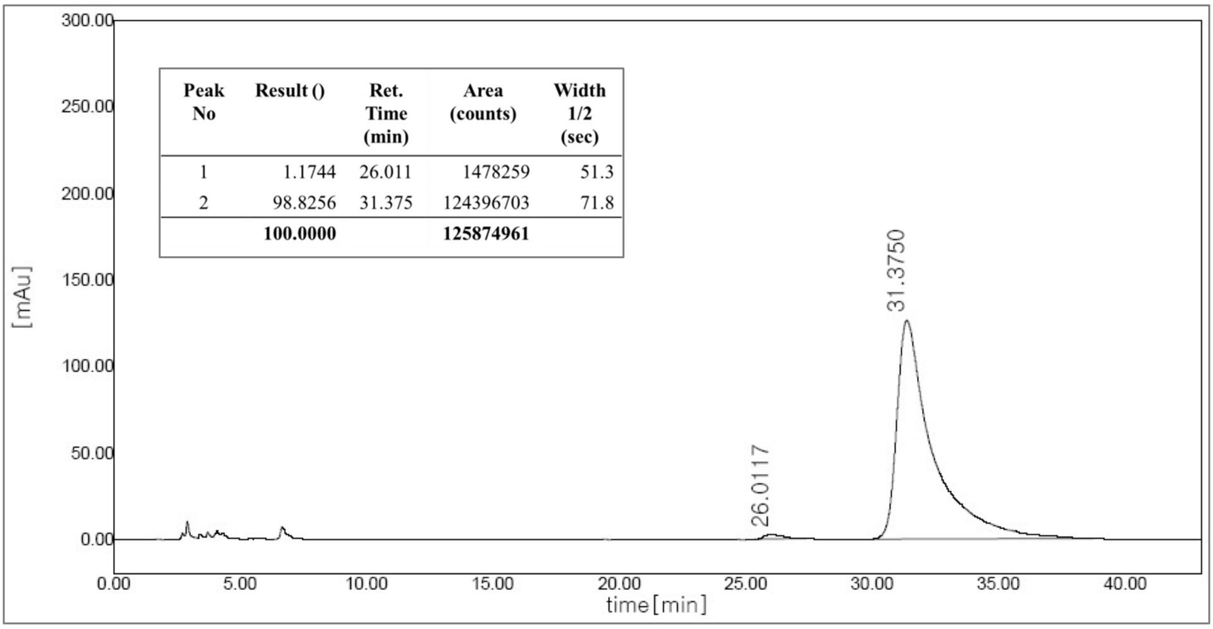

Peak No	Result ()	Ret. Time (min)	Area (counts)	Width 1/2 (sec)
1	1.1744	26.011	1478259	51.3
2	98.8256	31.375	124396703	71.8
	**100.0000**		**125874961**	

Supplementary Figure 48. HPLC spectra of compound **5ab**.

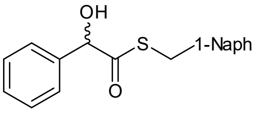

rac-**5ab**

Chiralcel OD-H (n-hexane/i-PrOH = 85/15, flow rate: 1.0 mL/min, wave length = 220 nm)

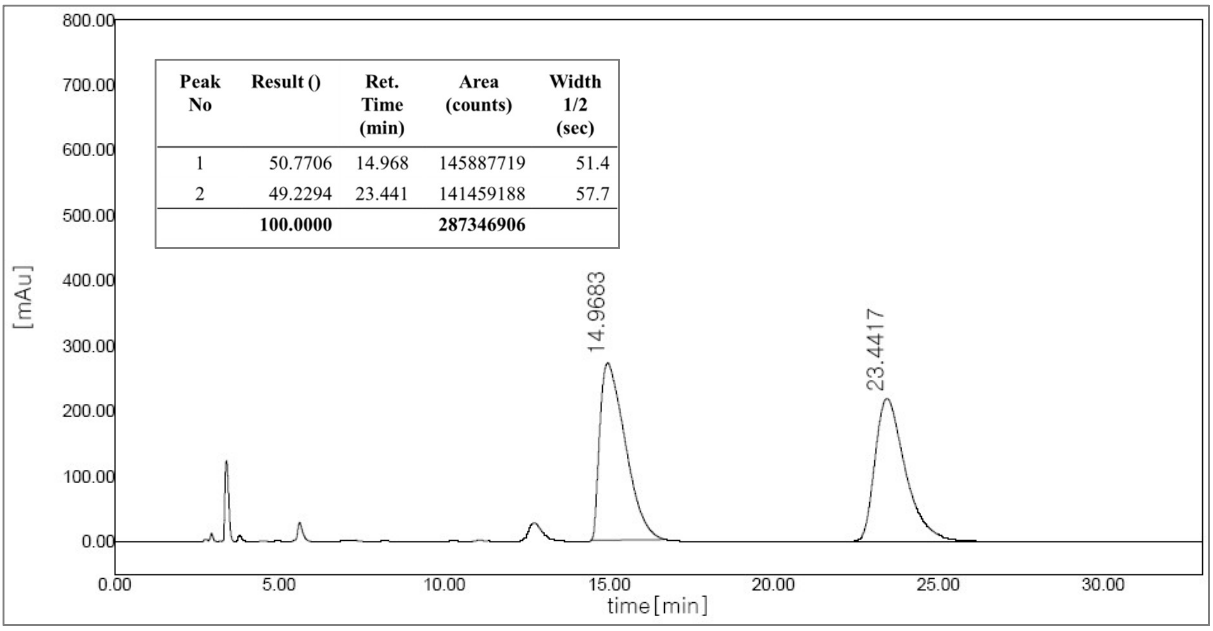

Peak No	Result ()	Ret. Time (min)	Area (counts)	Width 1/2 (sec)
1	50.7706	14.968	145887719	51.4
2	49.2294	23.441	141459188	57.7
	**100.0000**		**287346906**	

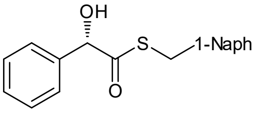

(*S*)-**5ab**

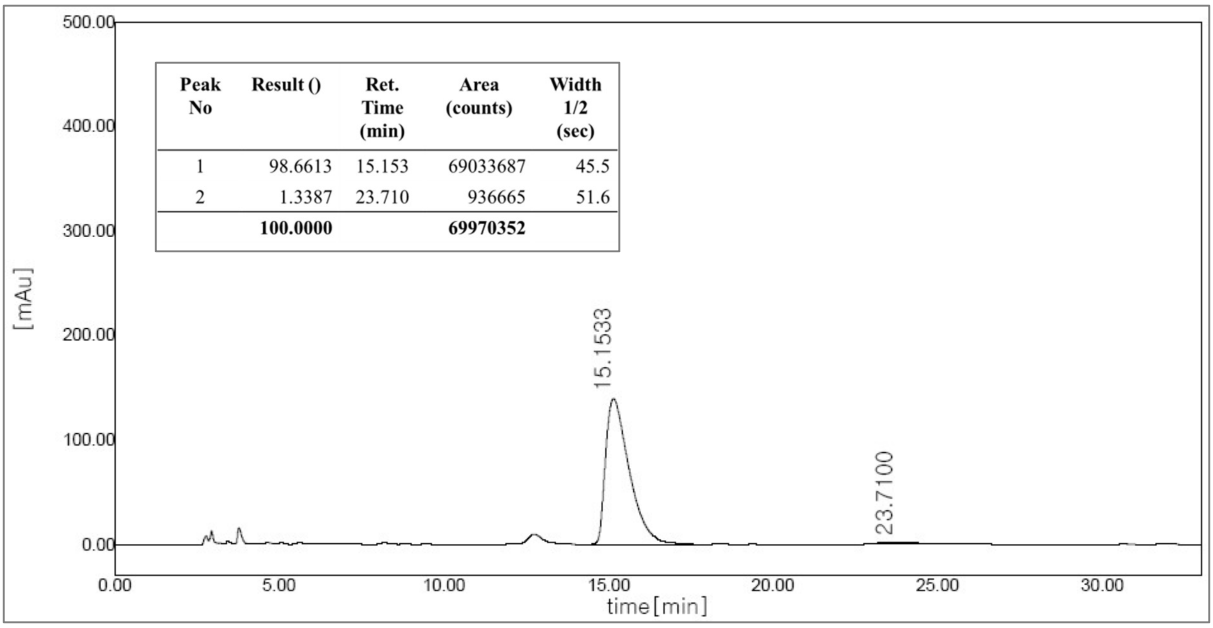

Peak No	Result ()	Ret. Time (min)	Area (counts)	Width 1/2 (sec)
1	98.6613	15.153	69033687	45.5
2	1.3387	23.710	936665	51.6
	**100.0000**		**69970352**	

Supplementary Figure 49. HPLC spectra of compound **5ac**.

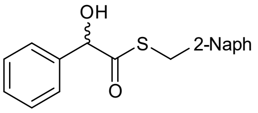

rac-**5ac**

Chiralcel OD-H (n-hexane/i-PrOH = 90/10, flow rate: 1.0 mL/min, wave length = 220 nm)

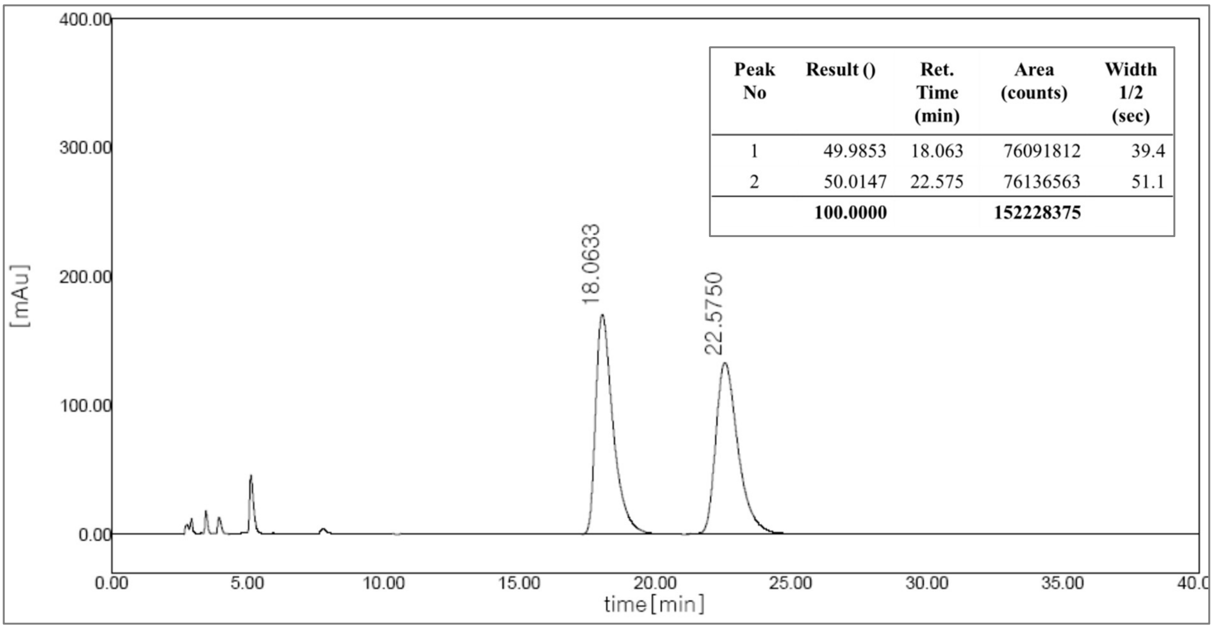

Peak No	Result ()	Ret. Time (min)	Area (counts)	Width 1/2 (sec)
1	49.9853	18.063	76091812	39.4
2	50.0147	22.575	76136563	51.1
	**100.0000**		**152228375**	

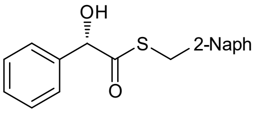

(*S*)-**5ac**

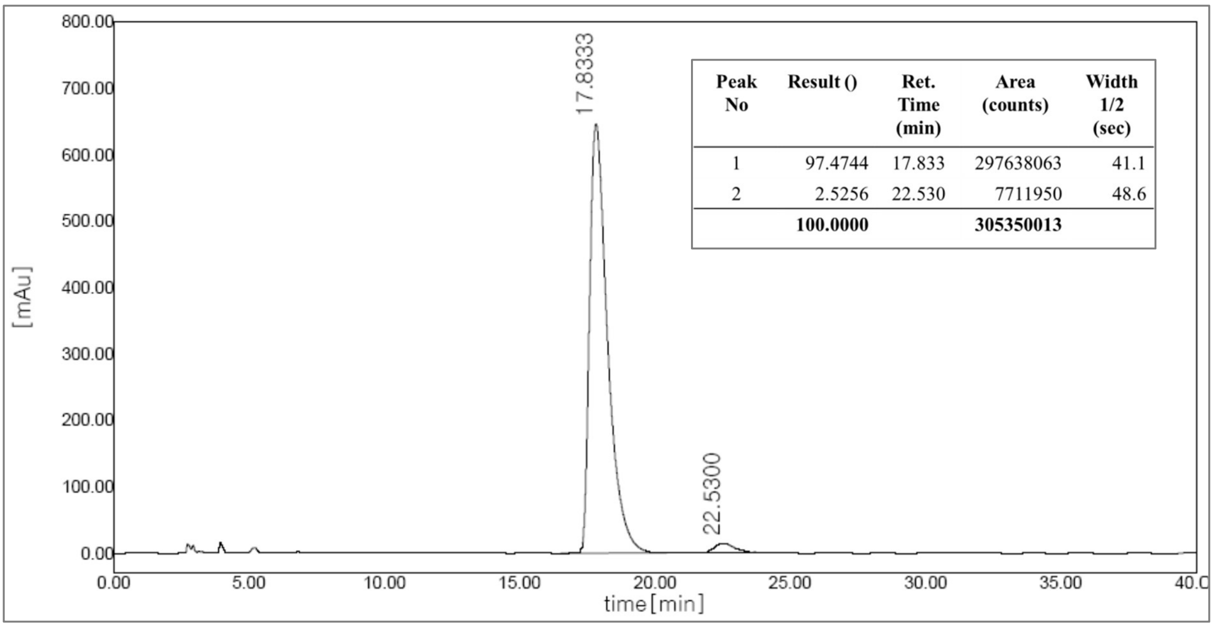

Peak No	Result ()	Ret. Time (min)	Area (counts)	Width 1/2 (sec)
1	97.4744	17.833	297638063	41.1
2	2.5256	22.530	7711950	48.6
	**100.0000**		**305350013**	

Supplementary Figure 50. HPLC spectra of compound **5ad**.

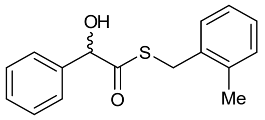

rac-**5ad**

Chiralcel OD-H (n-hexane/i-PrOH = 95/5, flow rate: 1.0 mL/min, wave length = 220 nm)

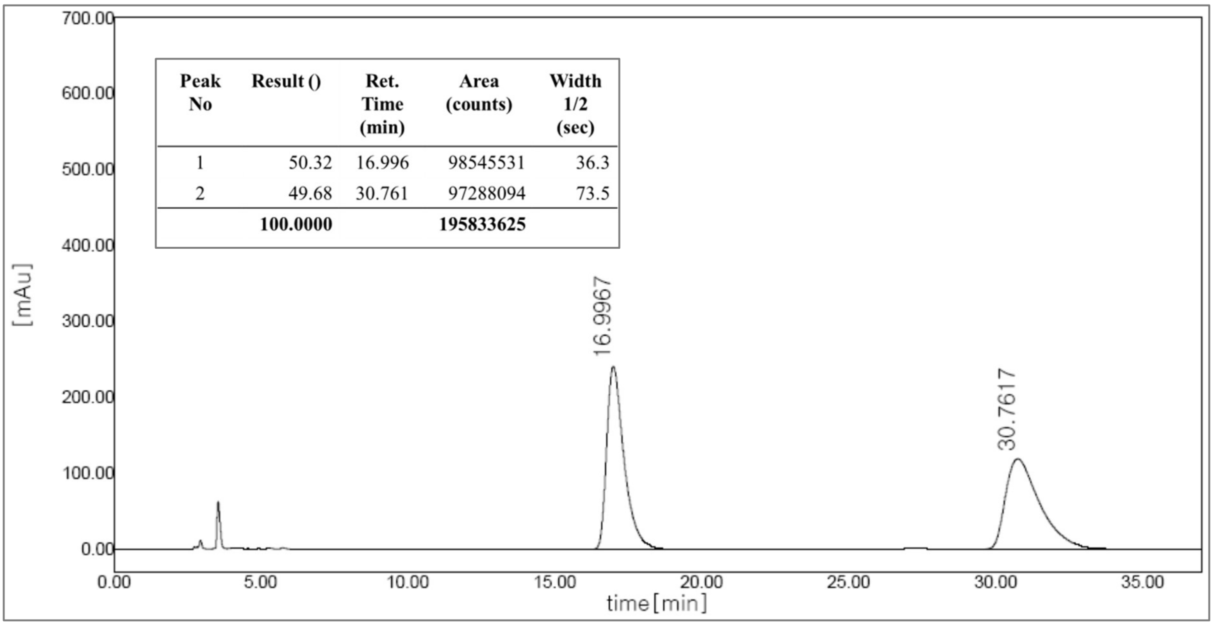

Peak No	Result ()	Ret. Time (min)	Area (counts)	Width 1/2 (sec)
1	50.32	16.996	98545531	36.3
2	49.68	30.761	97288094	73.5
	**100.0000**		**195833625**	

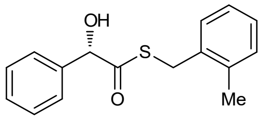

(*S*)-**5ad**

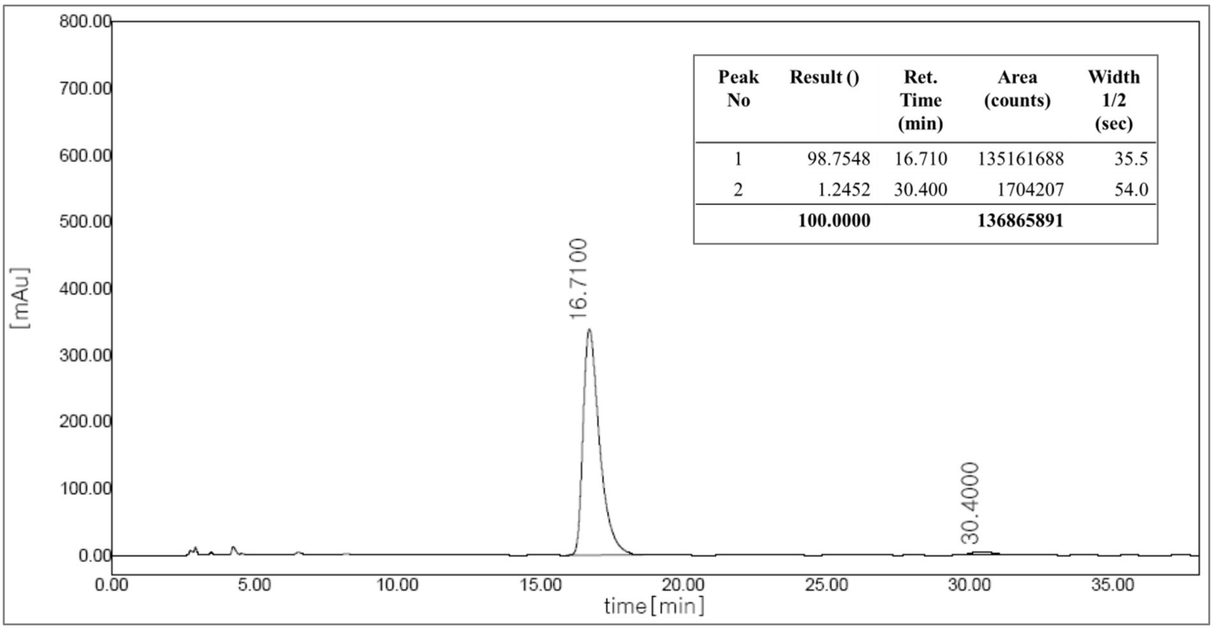

Peak No	Result ()	Ret. Time (min)	Area (counts)	Width 1/2 (sec)
1	98.7548	16.710	135161688	35.5
2	1.2452	30.400	1704207	54.0
	**100.0000**		**136865891**	

Supplementary Figure 51. HPLC spectra of compound **5ae**.

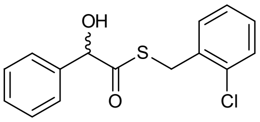

rac-**5ae**

Chiralcel OD-H (n-hexane/i-PrOH = 95/5, flow rate: 1.0 mL/min, wave length = 220 nm)

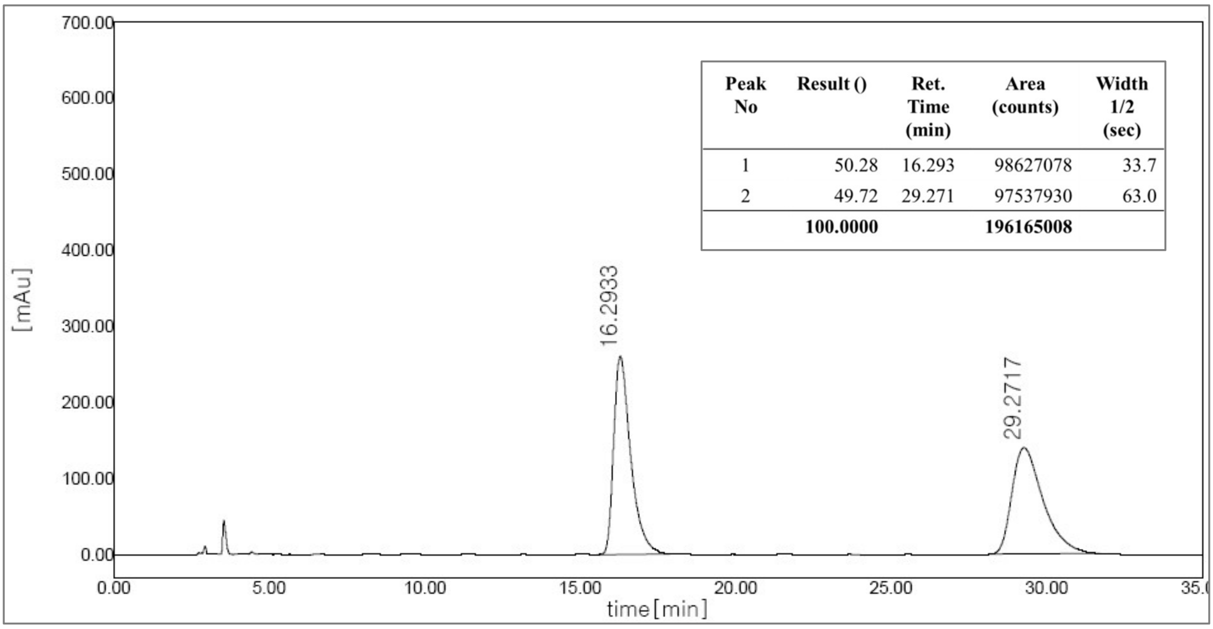

Peak No	Result ()	Ret. Time (min)	Area (counts)	Width 1/2 (sec)
1	50.28	16.293	98627078	33.7
2	49.72	29.271	97537930	63.0
	**100.0000**		**196165008**	

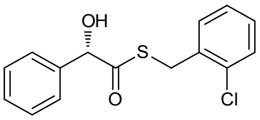

(*S*)-**5ae**

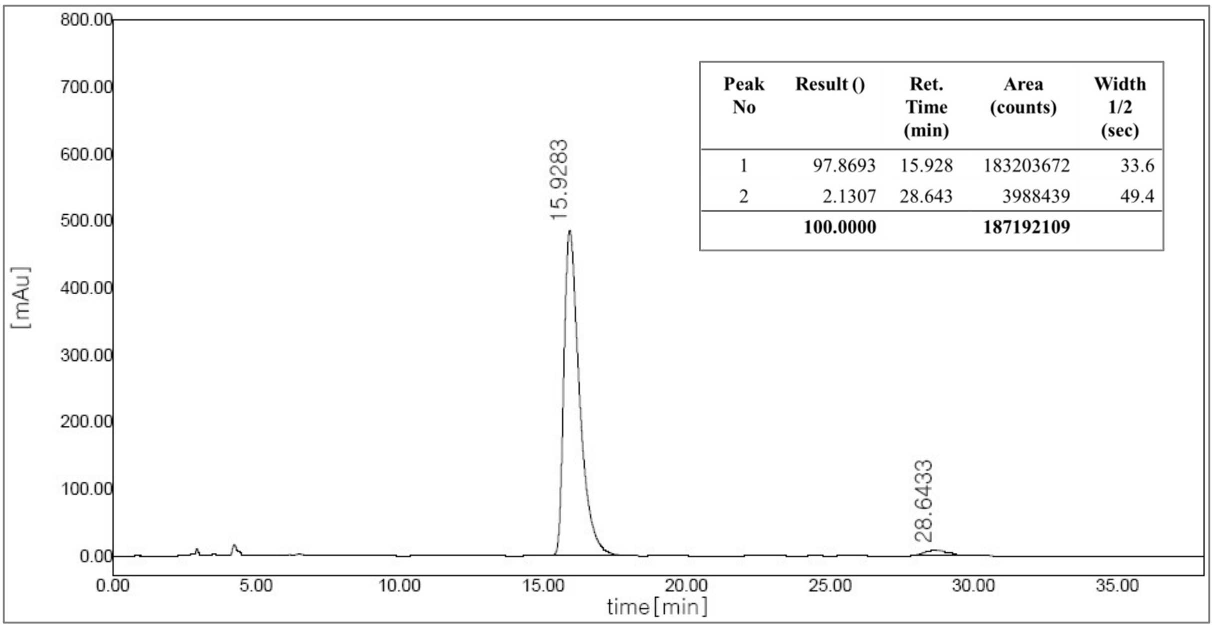

Peak No	Result ()	Ret. Time (min)	Area (counts)	Width 1/2 (sec)
1	97.8693	15.928	183203672	33.6
2	2.1307	28.643	3988439	49.4
	**100.0000**		**187192109**	

Supplementary Figure 52. HPLC spectra of compound **5af**.

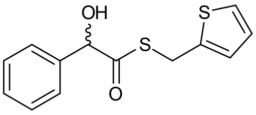

rac-**5af**

Chiralcel OD-H (n-hexane/i-PrOH = 95/5, flow rate: 1.0 mL/min, wave length = 220 nm)

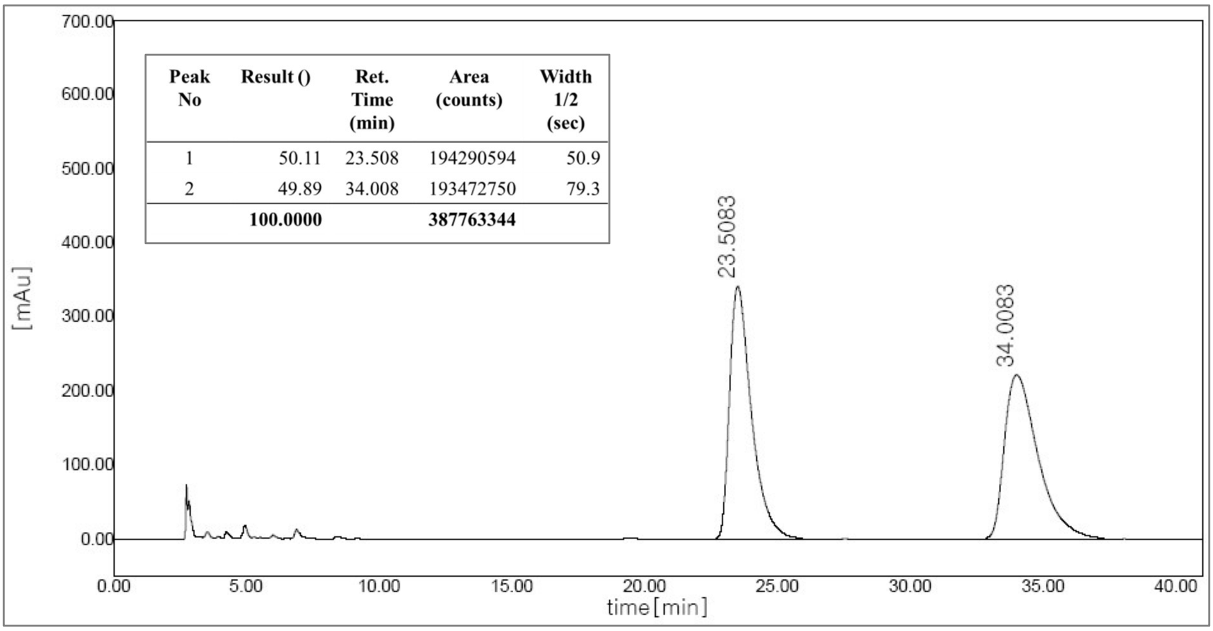

Peak No	Result ()	Ret. Time (min)	Area (counts)	Width 1/2 (sec)
1	50.11	23.508	194290594	50.9
2	49.89	34.008	193472750	79.3
	**100.0000**		**387763344**	

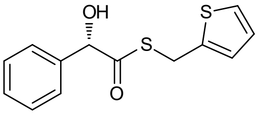

(*S*)-**5af**

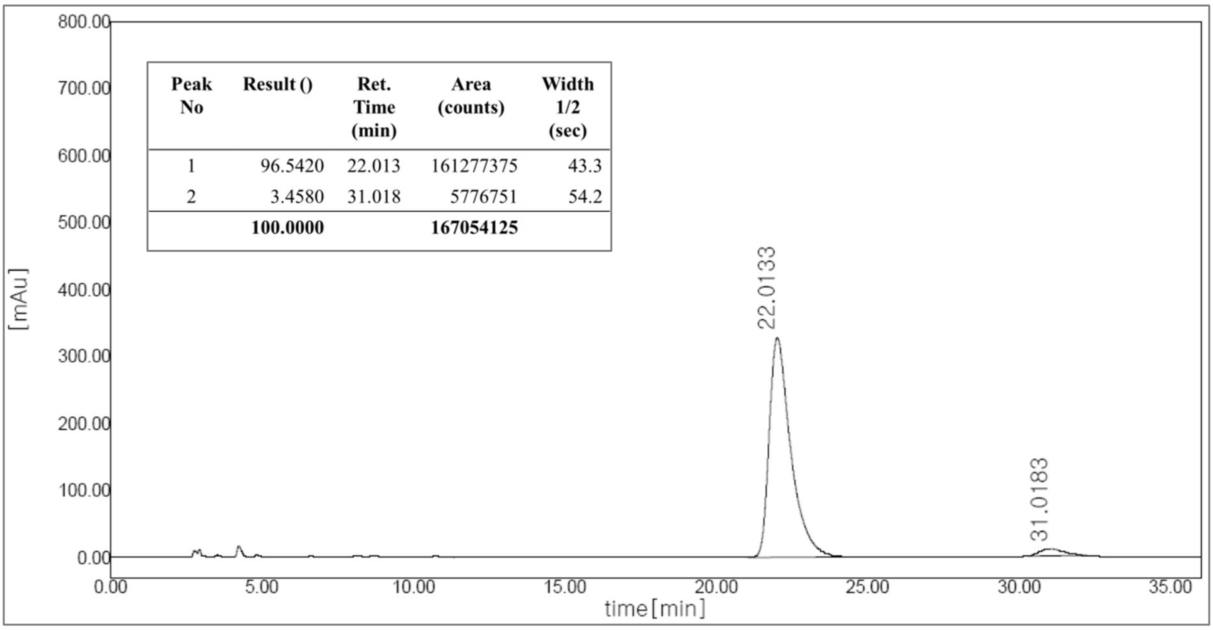

Peak No	Result ()	Ret. Time (min)	Area (counts)	Width 1/2 (sec)
1	96.5420	22.013	161277375	43.3
2	3.4580	31.018	5776751	54.2
	**100.0000**		**167054125**	

Supplementary Figure 53. HPLC spectra of compound **5ag**.

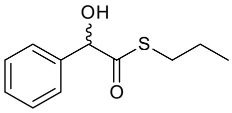

rac-**5ag**

Chiralcel OD-H (n-hexane/i-PrOH = 95/5, flow rate: 1.0 mL/min, wave length = 220 nm)

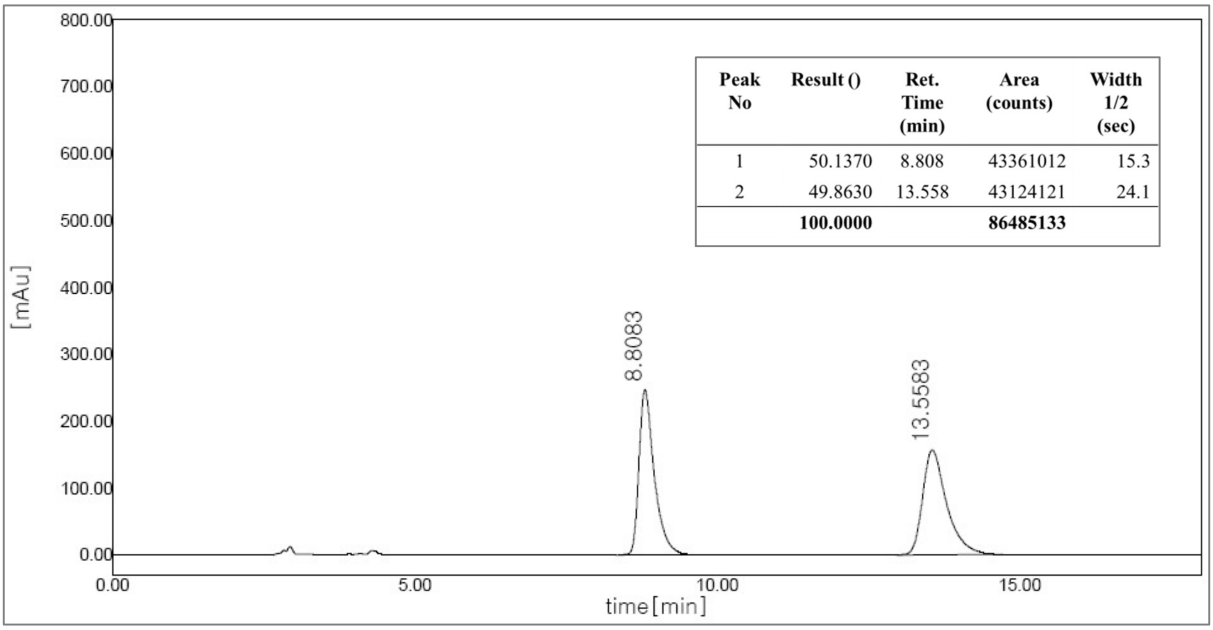

Peak No	Result ()	Ret. Time (min)	Area (counts)	Width 1/2 (sec)
1	50.1370	8.808	43361012	15.3
2	49.8630	13.558	43124121	24.1
	**100.0000**		**86485133**	

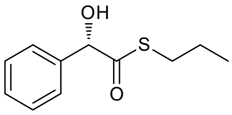

(*S*)-**5ag**

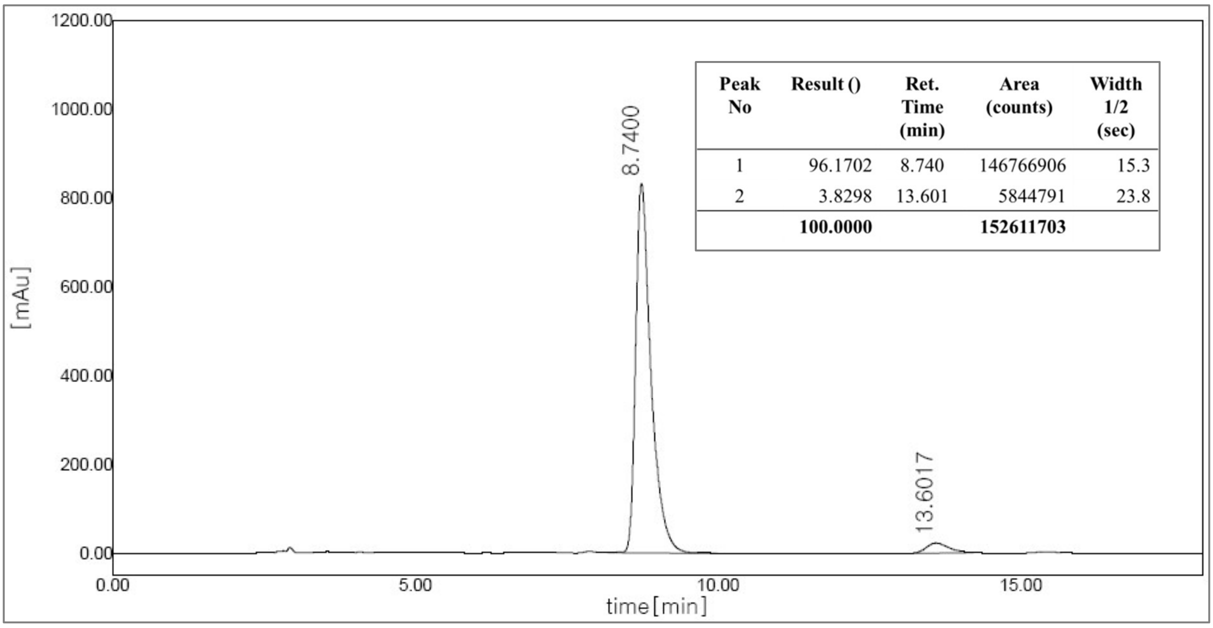

Peak No	Result ()	Ret. Time (min)	Area (counts)	Width 1/2 (sec)
1	96.1702	8.740	146766906	15.3
2	3.8298	13.601	5844791	23.8
	**100.0000**		**152611703**	

Supplementary Figure 54. HPLC spectra of compound **5ah**.

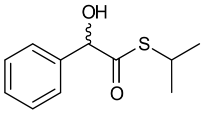

rac-**5ah**

Chiralcel OD-H (n-hexane/i-PrOH = 95/5, flow rate: 1.0 mL/min, wave length = 220 nm)

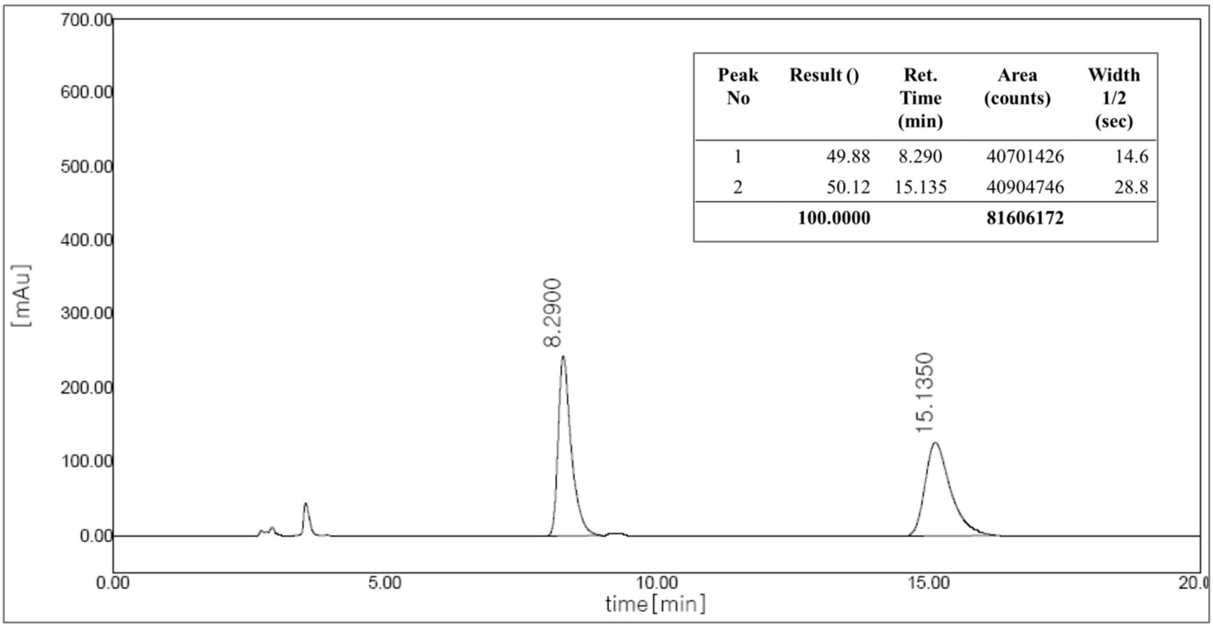

Peak No	Result ()	Ret. Time (min)	Area (counts)	Width 1/2 (sec)
1	49.88	8.290	40701426	14.6
2	50.12	15.135	40904746	28.8
	**100.0000**		**81606172**	

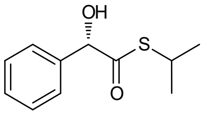

(*S*)-**5ah**

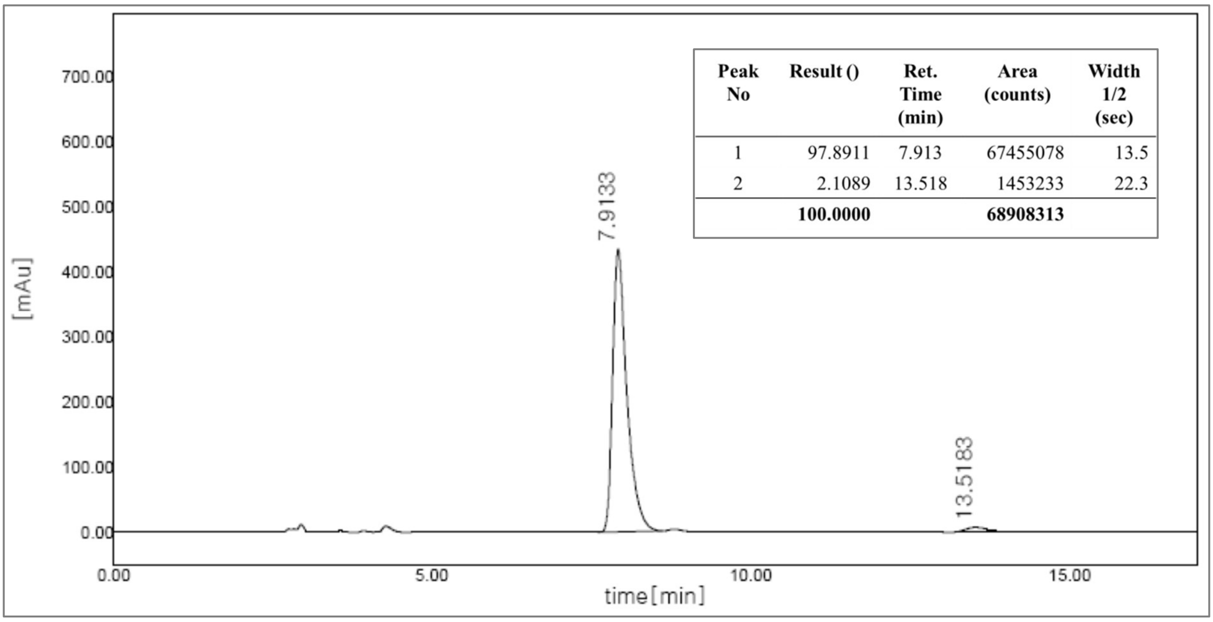

Peak No	Result ()	Ret. Time (min)	Area (counts)	Width 1/2 (sec)
1	97.8911	7.913	67455078	13.5
2	2.1089	13.518	1453233	22.3
	**100.0000**		**68908313**	

Supplementary Figure 55. HPLC spectra of compound **5ai**.

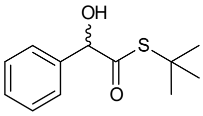

rac-**5ai**

Chiralcel OD-H (n-hexane/i-PrOH = 95/5, flow rate: 1.0 mL/min, wave length = 220 nm)

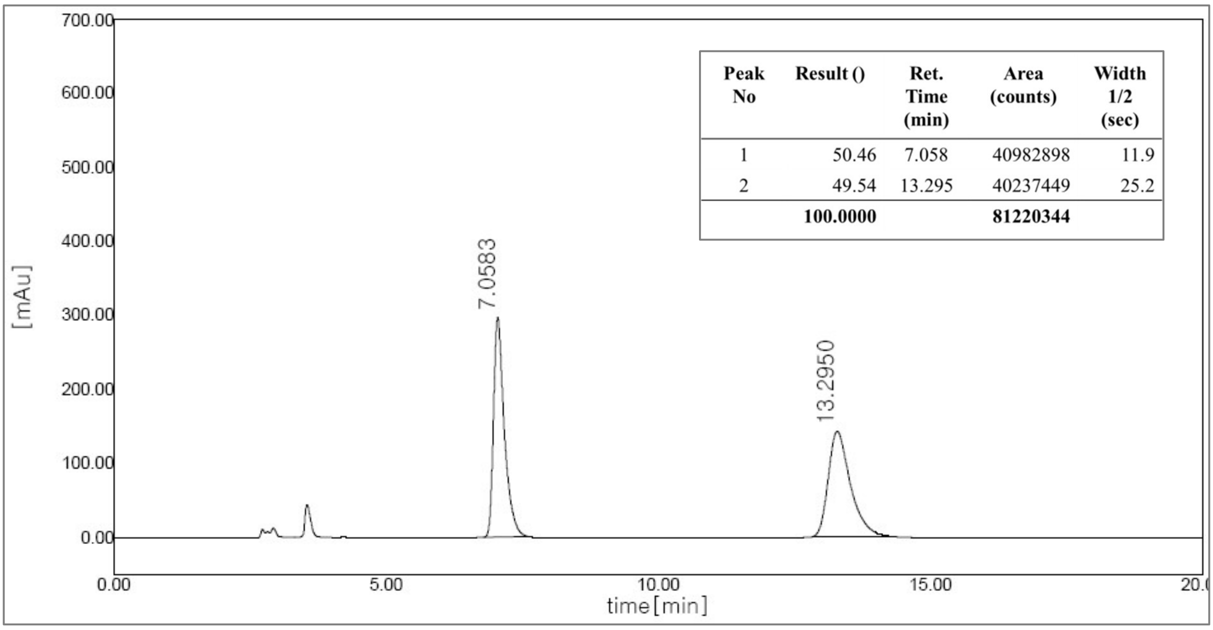

Peak No	Result ()	Ret. Time (min)	Area (counts)	Width 1/2 (sec)
1	50.46	7.058	40982898	11.9
2	49.54	13.295	40237449	25.2
	**100.0000**		**81220344**	

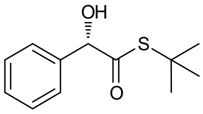

(*S*)-**5ai**

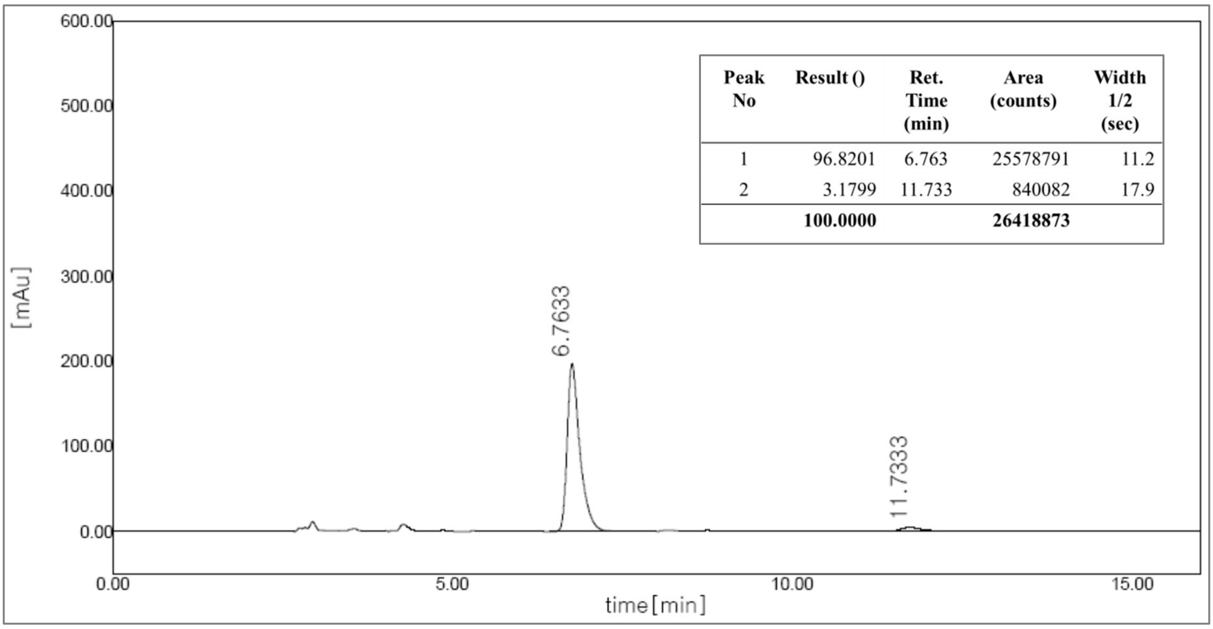

Peak No	Result ()	Ret. Time (min)	Area (counts)	Width 1/2 (sec)
1	96.8201	6.763	25578791	11.2
2	3.1799	11.733	840082	17.9
	**100.0000**		**26418873**	

Supplementary Figure 56. HPLC spectra of compound **5aj**.

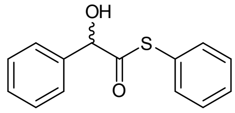

rac-**5aj**

Chiralcel OD-H (n-hexane/i-PrOH = 95/5, flow rate: 1.0 mL/min, wave length = 220 nm)

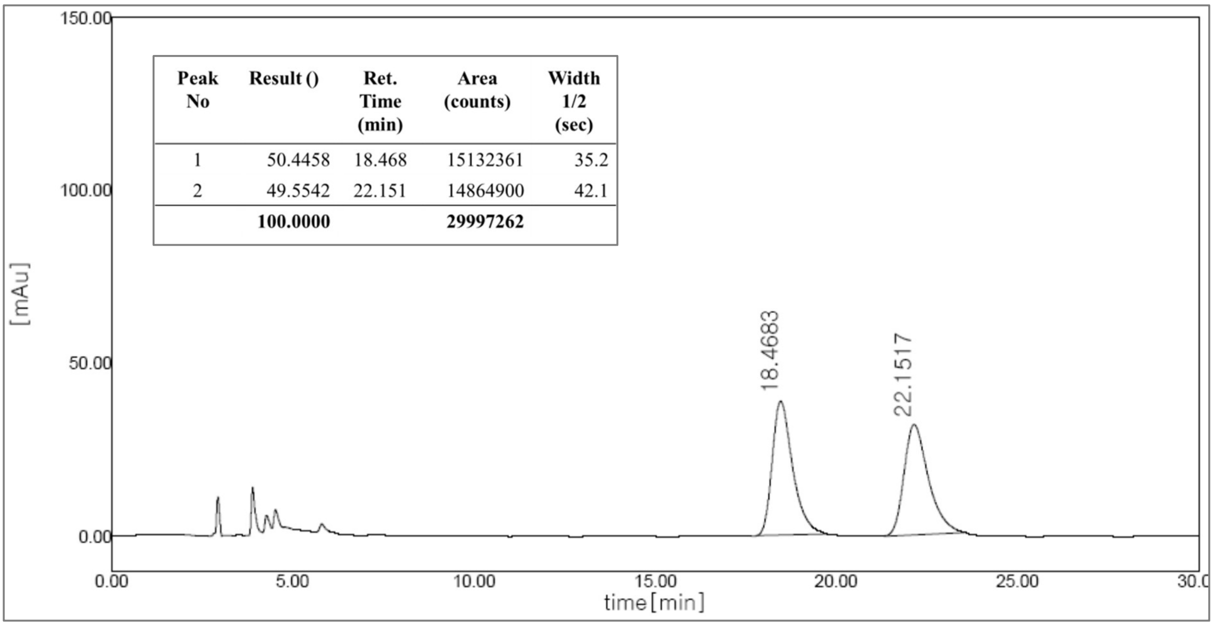

Peak No	Result ()	Ret. Time (min)	Area (counts)	Width 1/2 (sec)
1	50.4458	18.468	15132361	35.2
2	49.5542	22.151	14864900	42.1
	**100.0000**		**29997262**	

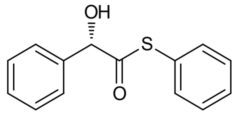

(*S*)-**5aj**

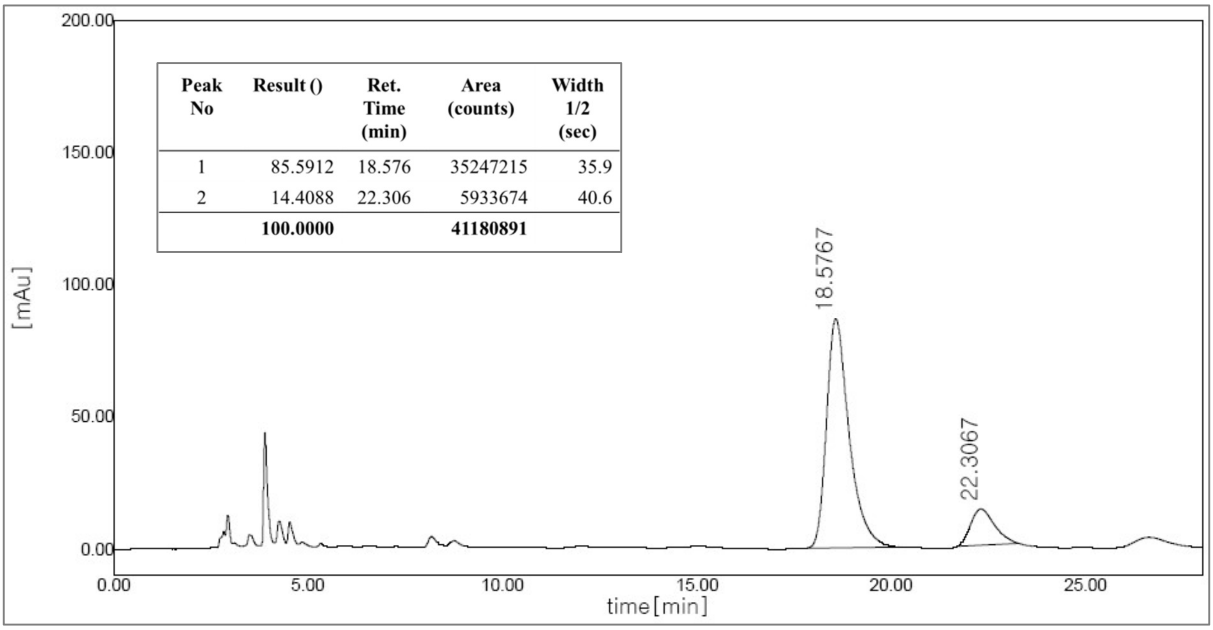

Peak No	Result ()	Ret. Time (min)	Area (counts)	Width 1/2 (sec)
1	85.5912	18.576	35247215	35.9
2	14.4088	22.306	5933674	40.6
	**100.0000**		**41180891**	

Supplementary Figure 57. HPLC spectra of compound **5ak**.

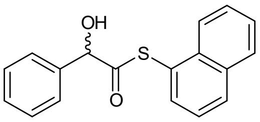

rac-**5ak**

Chiralcel OD-H (n-hexane/i-PrOH = 90/10, flow rate: 1.0 mL/min, wave length = 220 nm)

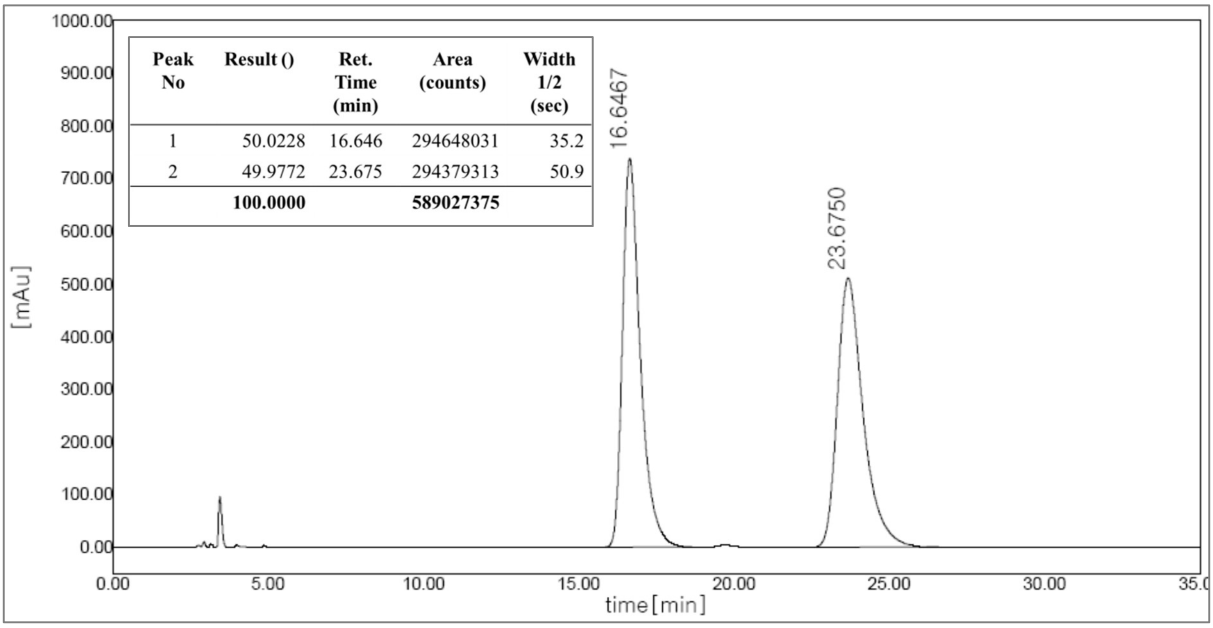

Peak No	Result ()	Ret. Time (min)	Area (counts)	Width 1/2 (sec)
1	50.0228	16.646	294648031	35.2
2	49.9772	23.675	294379313	50.9
	**100.0000**		**589027375**	

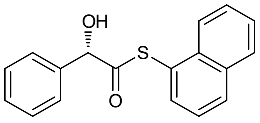

(*S*)-**5ak**

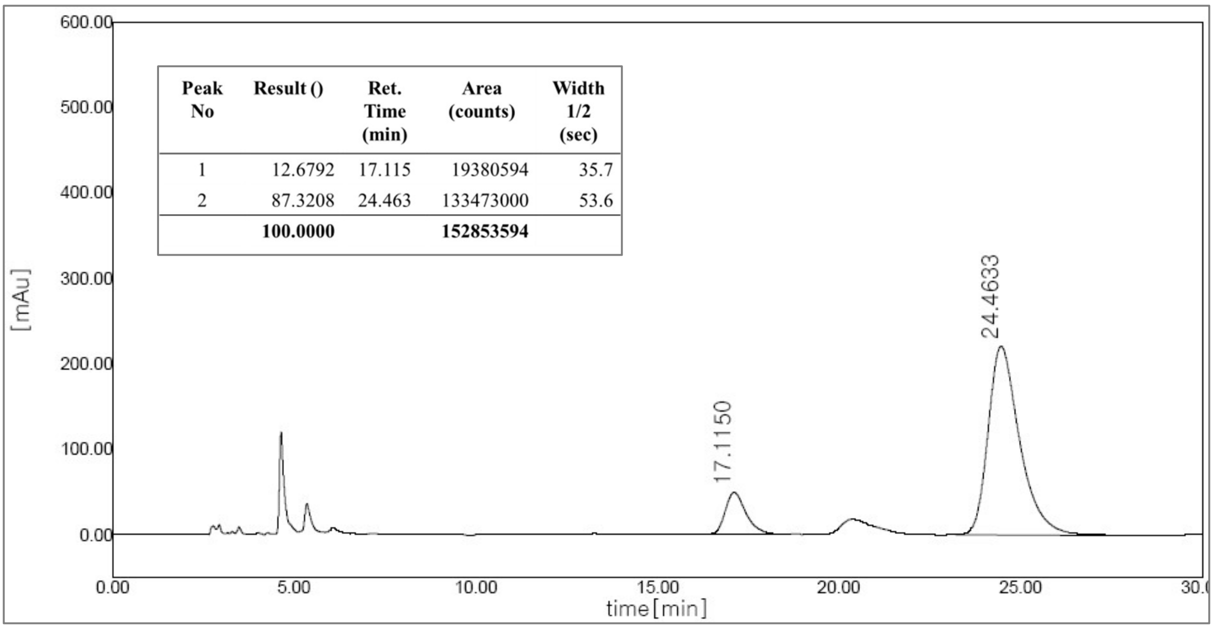

Peak No	Result ()	Ret. Time (min)	Area (counts)	Width 1/2 (sec)
1	12.6792	17.115	19380594	35.7
2	87.3208	24.463	133473000	53.6
	**100.0000**		**152853594**	

Supplementary Figure 58. HPLC spectra of compound **5al**.

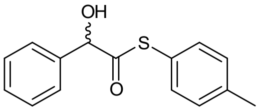

rac-**5al**

Chiralcel OD-H (n-hexane/i-PrOH = 95/5, flow rate: 1.0 mL/min, wave length = 220 nm)

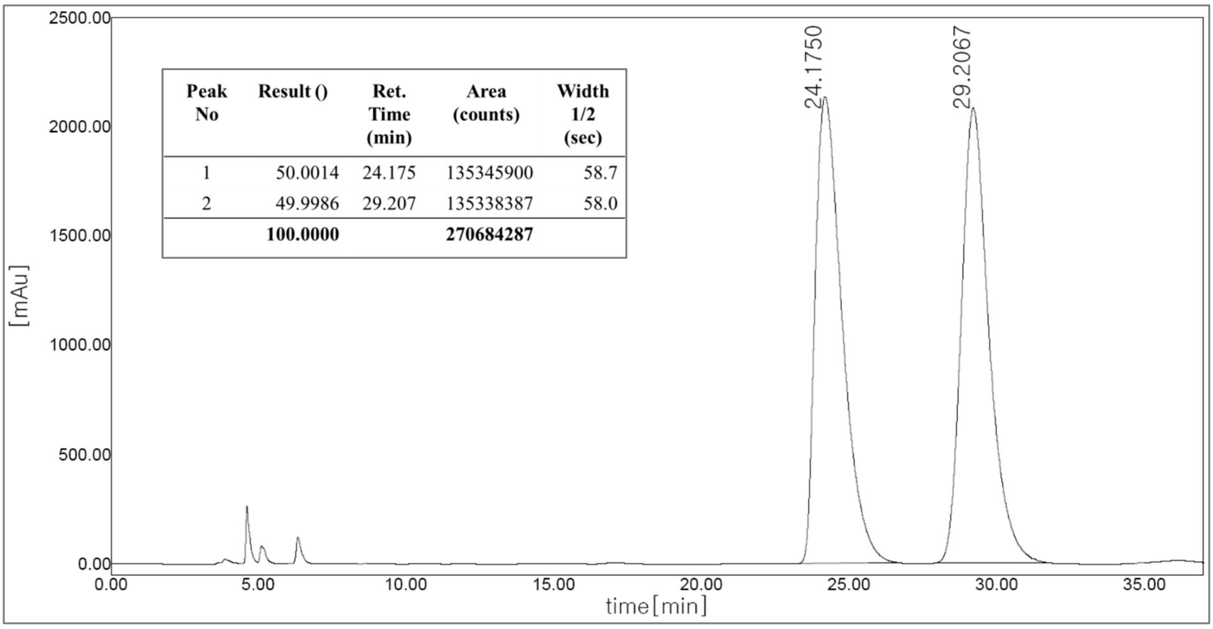

Peak No	Result ()	Ret. Time (min)	Area (counts)	Width 1/2 (sec)
1	50.0014	24.175	135345900	58.7
2	49.9986	29.207	135338387	58.0
	**100.0000**		**270684287**	

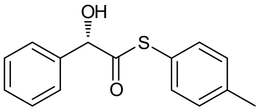

(*S*)-**5al**

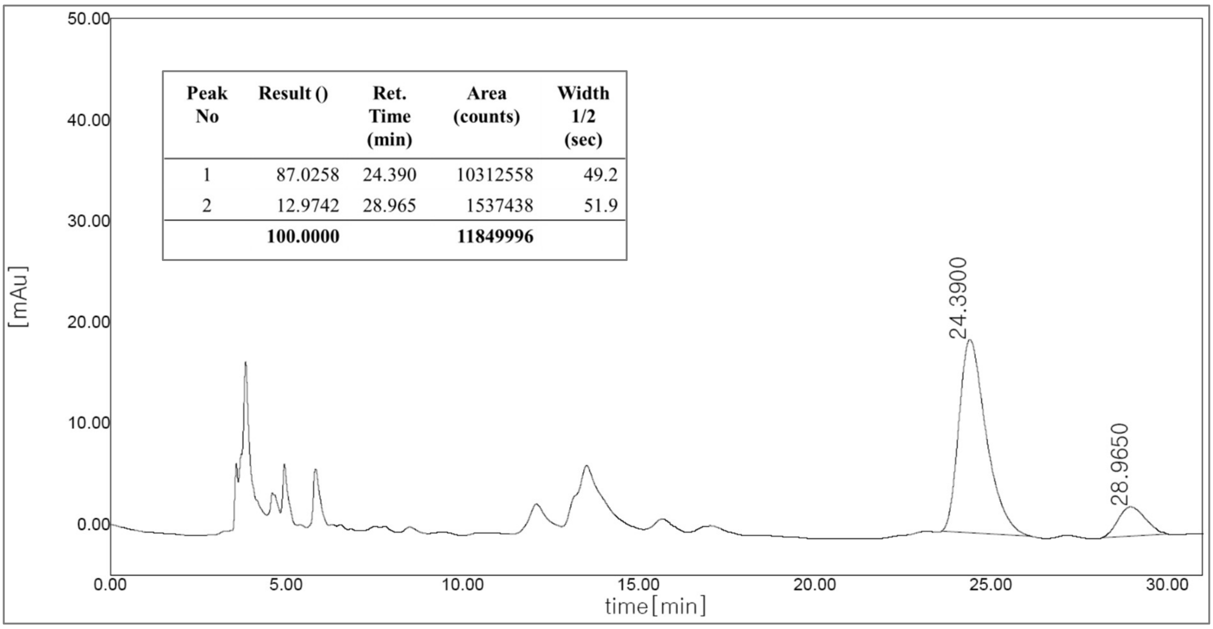

Peak No	Result ()	Ret. Time (min)	Area (counts)	Width 1/2 (sec)
1	87.0258	24.390	10312558	49.2
2	12.9742	28.965	1537438	51.9
	**100.0000**		**11849996**	

Supplementary Figure 59. HPLC spectra of compound **5am**.

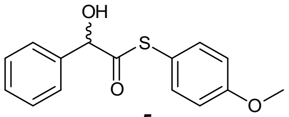

rac-**5am**

Regis (*R*,*R*) Whelk-O1

(n-hexane/i-PrOH = 90/10, flow rate: 1.0 mL/min, wave length = 220 nm)

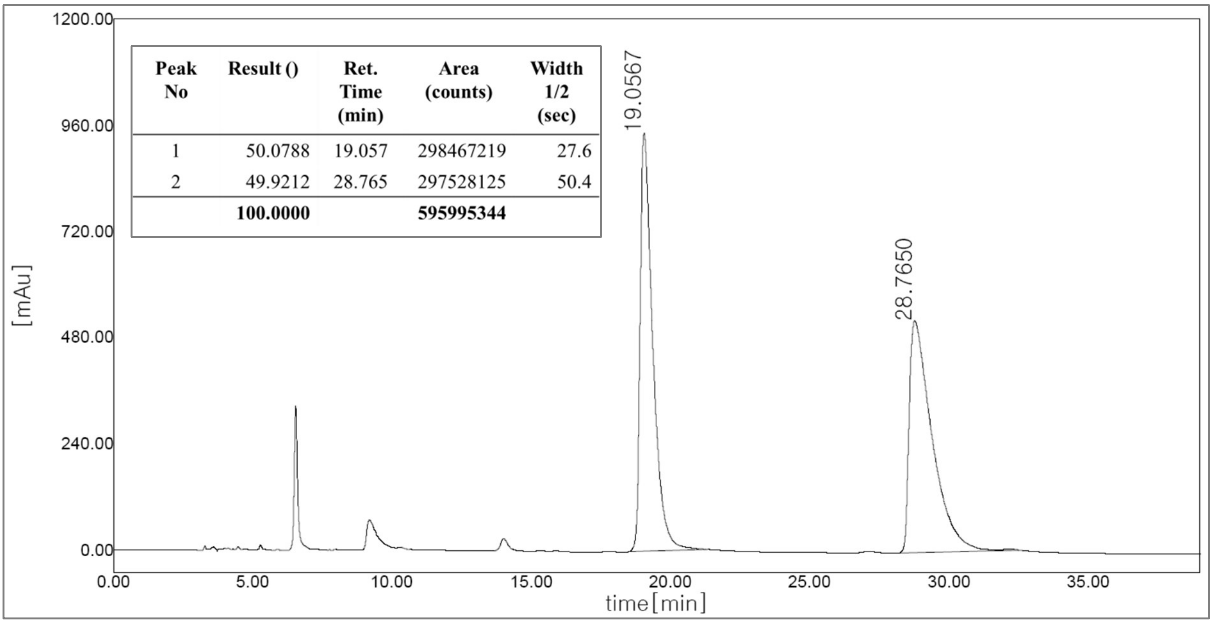

Peak No	Result ()	Ret. Time (min)	Area (counts)	Width 1/2 (sec)
1	50.0788	19.057	298467219	27.6
2	49.9212	28.765	297528125	50.4
	**100.0000**		**595995344**	

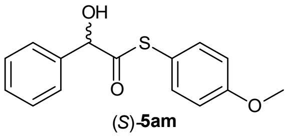

(*S*)-**5am**

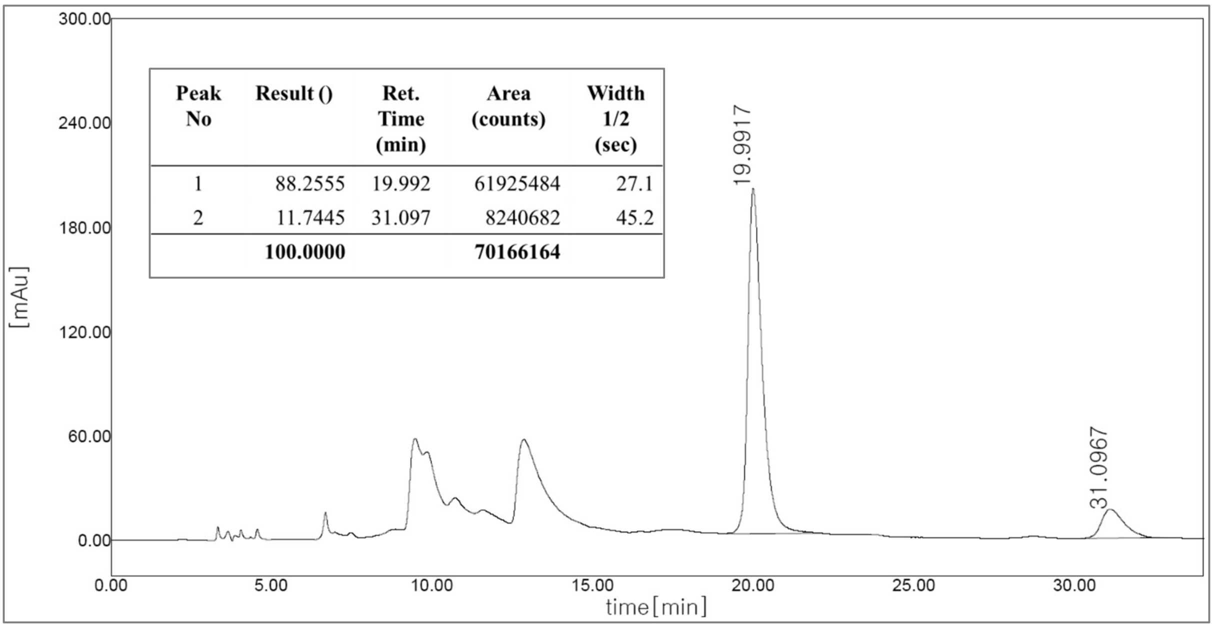

Peak No	Result ()	Ret. Time (min)	Area (counts)	Width 1/2 (sec)
1	88.2555	19.992	61925484	27.1
2	11.7445	31.097	8240682	45.2
	**100.0000**		**70166164**	

Supplementary Figure 60. HPLC spectra of compound **5an**.

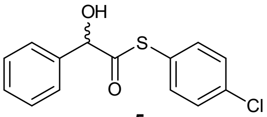

rac-**5an**

Chiralcel OD-H (n-hexane/i-PrOH = 95/5, flow rate: 1.0 mL/min, wave length = 220 nm)

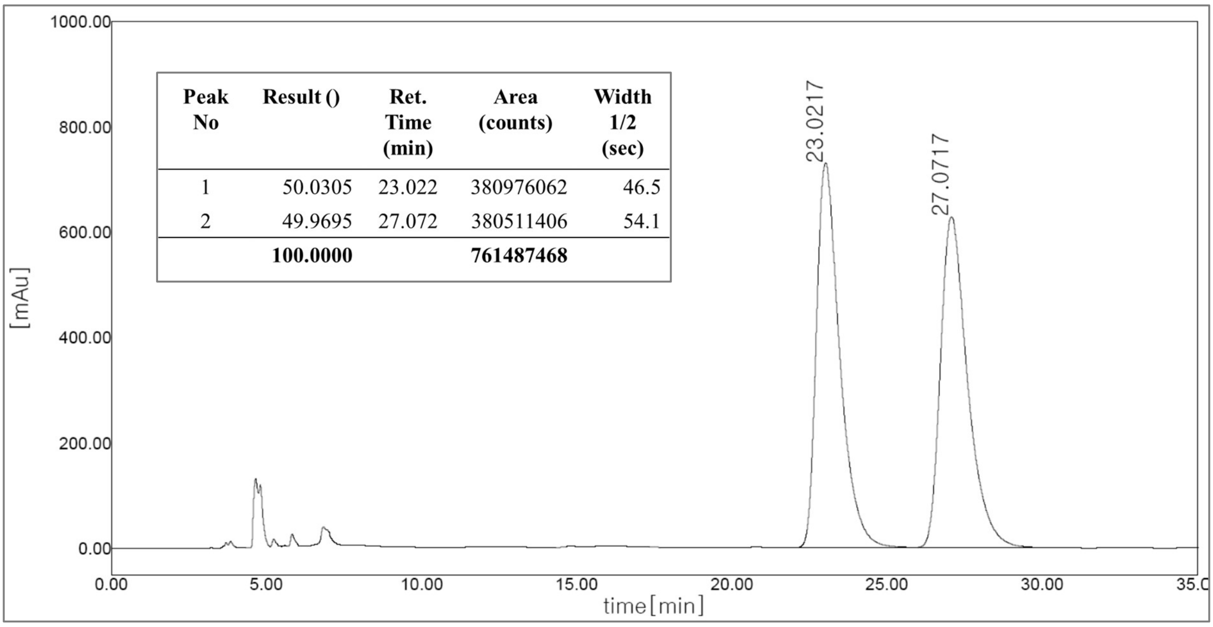

Peak No	Result ()	Ret. Time (min)	Area (counts)	Width 1/2 (sec)
1	50.0305	23.022	380976062	46.5
2	49.9695	27.072	380511406	54.1
	**100.0000**		**761487468**	

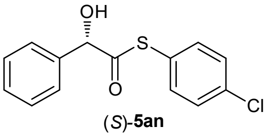

(*S*)-**5an**

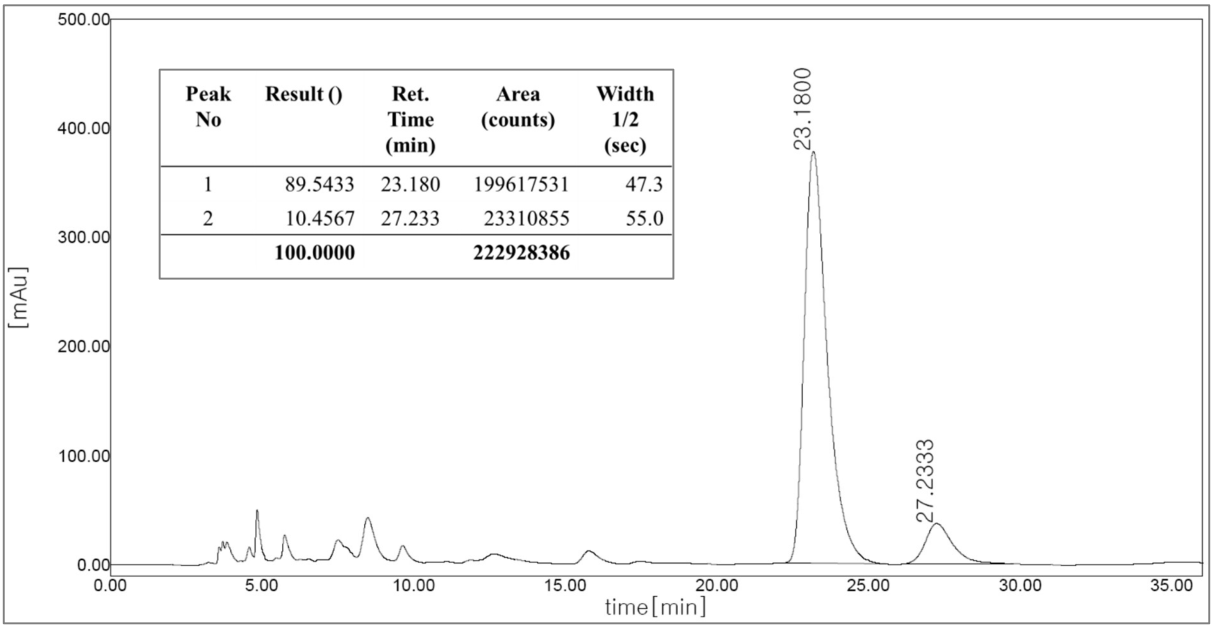

Peak No	Result ()	Ret. Time (min)	Area (counts)	Width 1/2 (sec)
1	89.5433	23.180	199617531	47.3
2	10.4567	27.233	23310855	55.0
	**100.0000**		**222928386**	

Supplementary Figure 61. HPLC spectra of compound **5ao**.

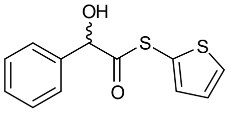

rac-**5al**

Chiralcel OD-H (n-hexane/i-PrOH = 95/5, flow rate: 1.0 mL/min, wave length = 220 nm)

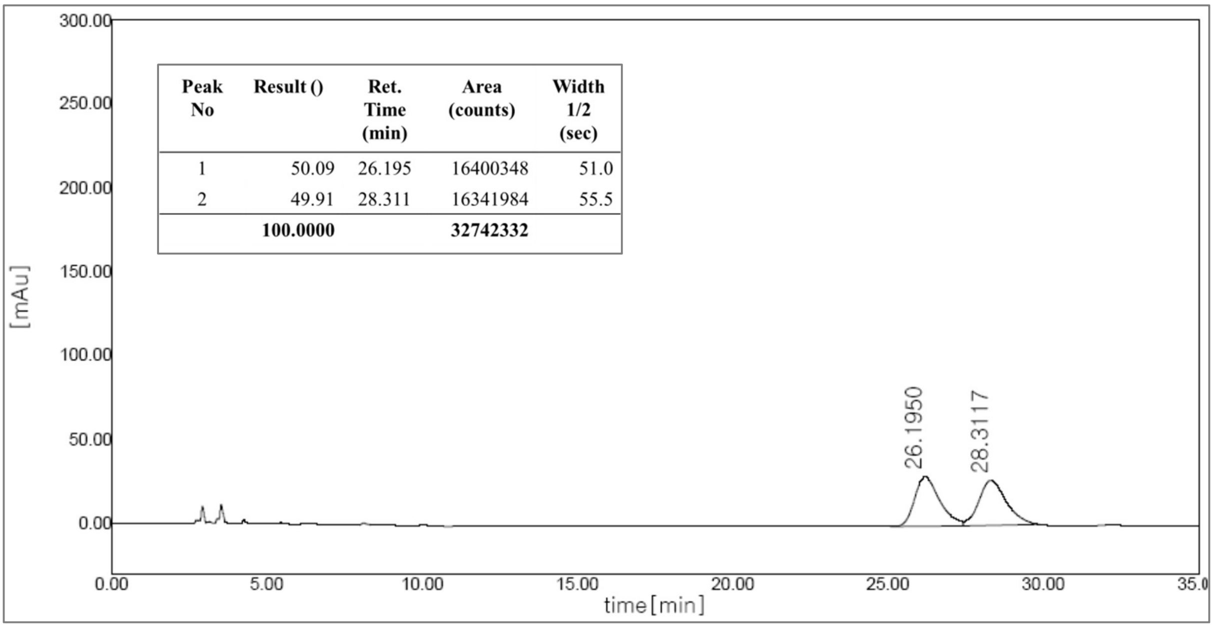

Peak No	Result ()	Ret. Time (min)	Area (counts)	Width 1/2 (sec)
1	50.09	26.195	16400348	51.0
2	49.91	28.311	16341984	55.5
	**100.0000**		**32742332**	

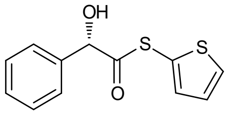

(*S*)-**5al**

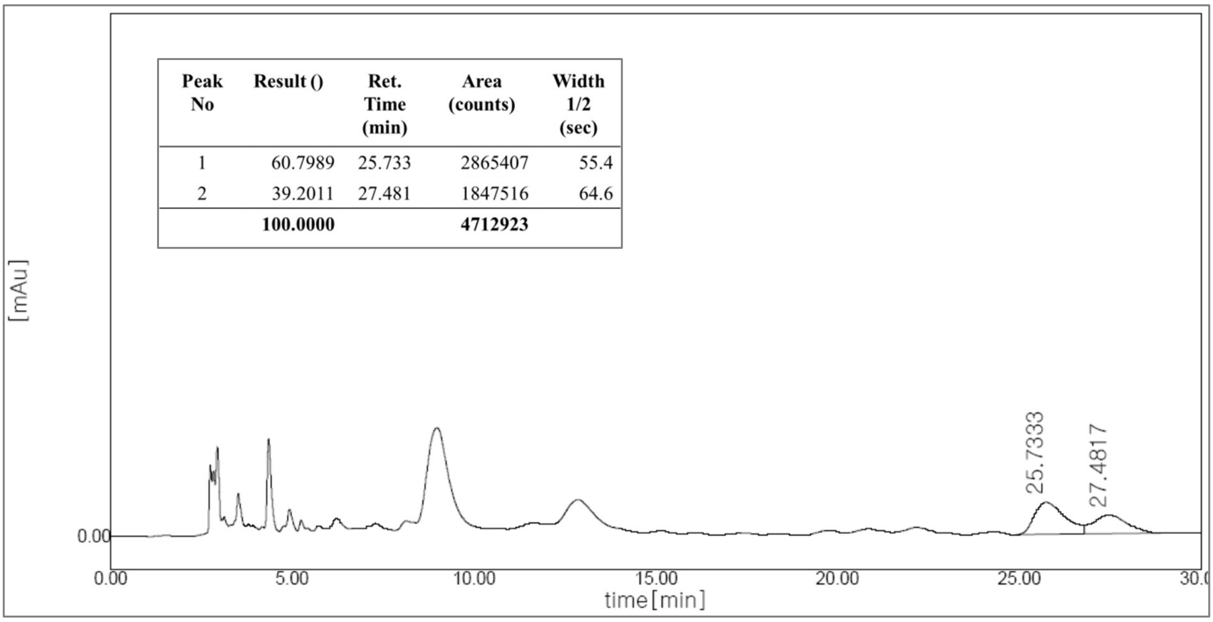

Peak No	Result ()	Ret. Time (min)	Area (counts)	Width 1/2 (sec)
1	60.7989	25.733	2865407	55.4
2	39.2011	27.481	1847516	64.6
	**100.0000**		**4712923**	

Supplementary Figure 62. HPLC spectra of compound **5b**.

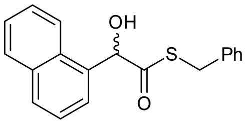

rac-**5b**

Chiralcel OD-H (n-hexane/i-PrOH = 90/10, flow rate: 1.0 mL/min, wave length = 220 nm)

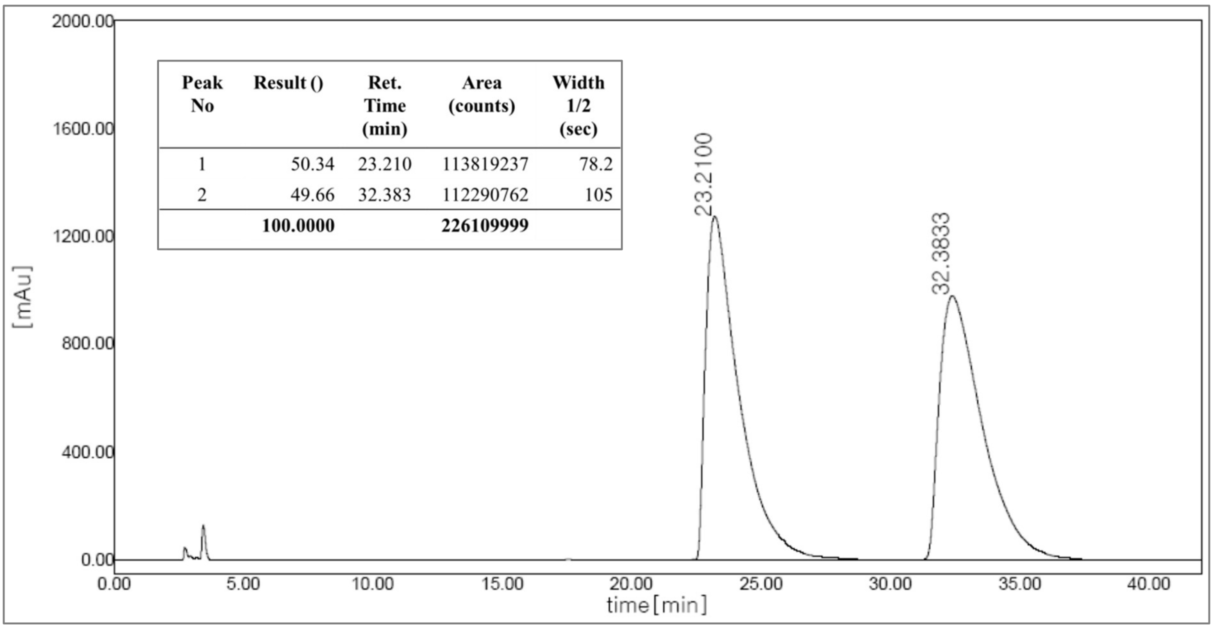

Peak No	Result ()	Ret. Time (min)	Area (counts)	Width 1/2 (sec)
1	50.34	23.210	113819237	78.2
2	49.66	32.383	112290762	105
	**100.0000**		**226109999**	

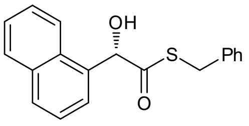

(*S*)-**5b**

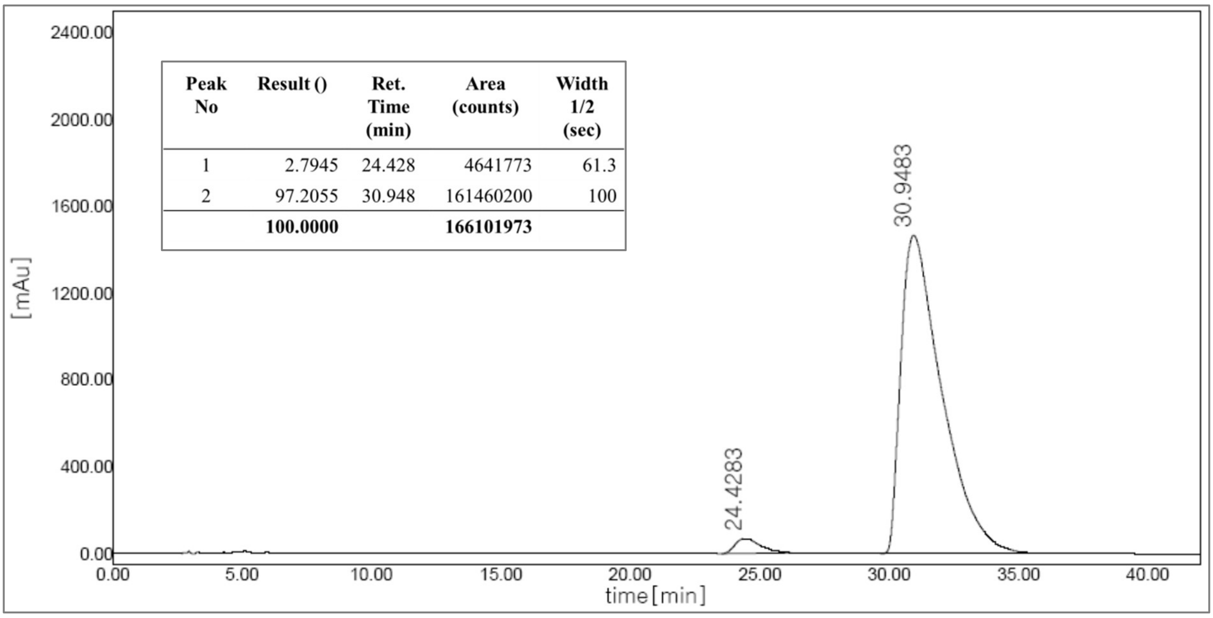

Peak No	Result ()	Ret. Time (min)	Area (counts)	Width 1/2 (sec)
1	2.7945	24.428	4641773	61.3
2	97.2055	30.948	161460200	100
	**100.0000**		**166101973**	

Supplementary Figure 63. HPLC spectra of compound **5c**.

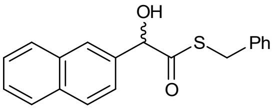

rac-**5c**

Chiralpak AD-H (n-hexane/i-PrOH = 96/4, flow rate: 1.0 mL/min, wave length = 220 nm)

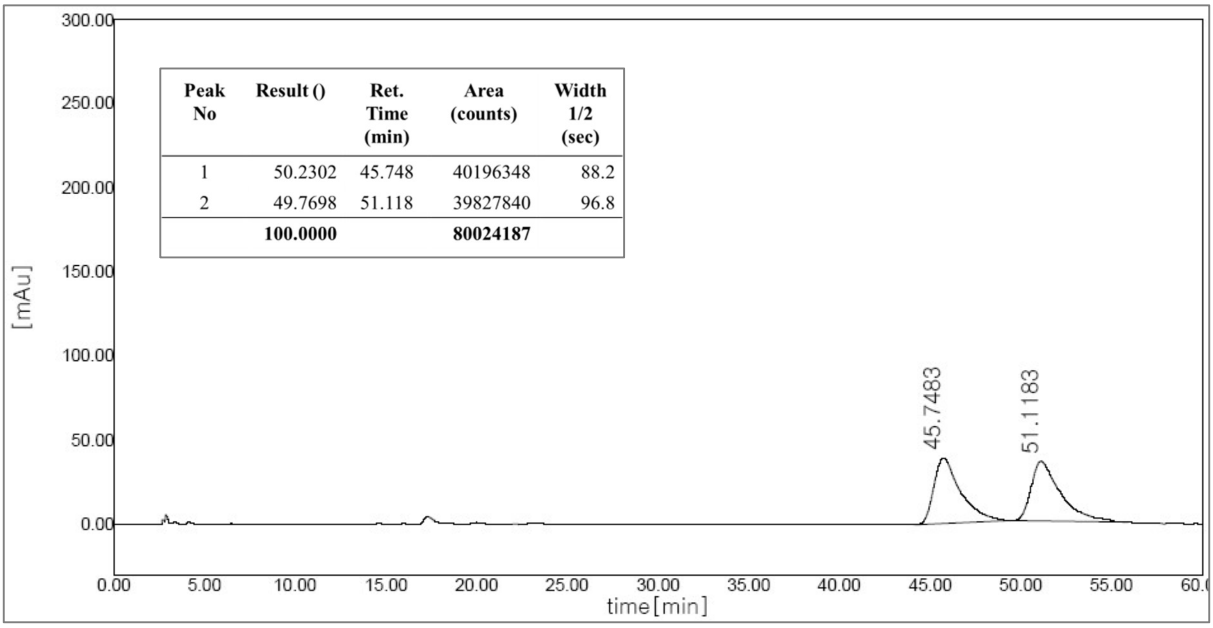

Peak No	Result ()	Ret. Time (min)	Area (counts)	Width 1/2 (sec)
1	50.2302	45.748	40196348	88.2
2	49.7698	51.118	39827840	96.8
	**100.0000**		**80024187**	

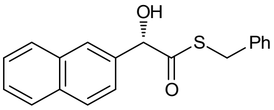

(*S*)-**5c**

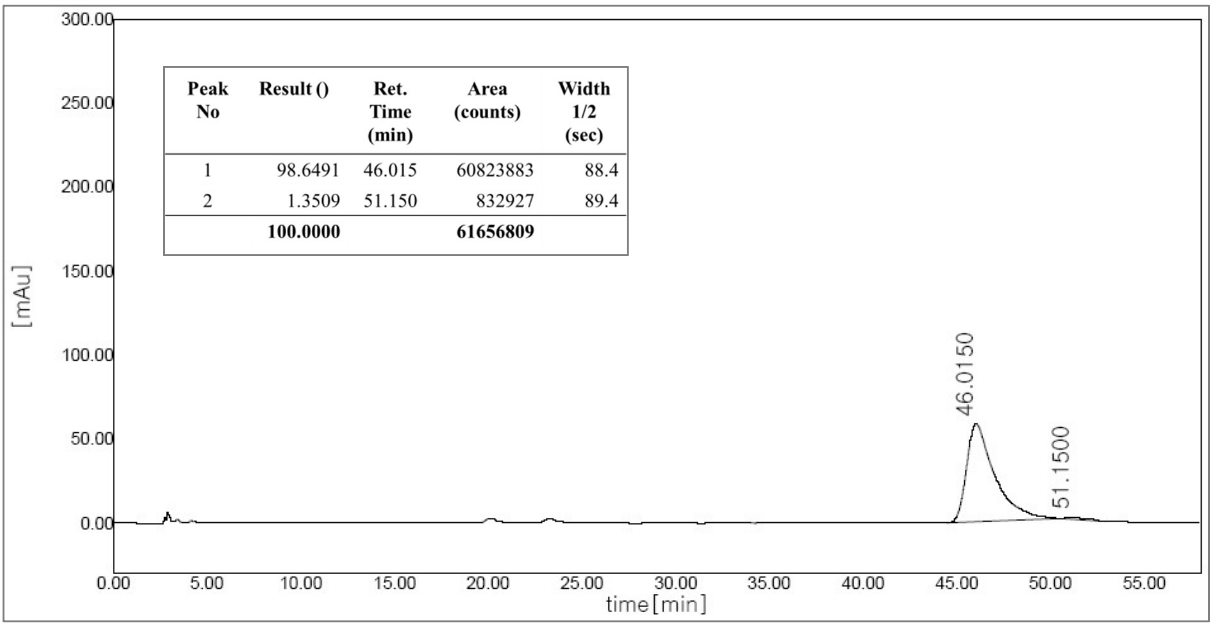

Peak No	Result ()	Ret. Time (min)	Area (counts)	Width 1/2 (sec)
1	98.6491	46.015	60823883	88.4
2	1.3509	51.150	832927	89.4
	**100.0000**		**61656809**	

Supplementary Figure 64. HPLC spectra of compound **5d**.

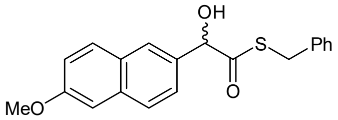

rac-**5d**

Chiralcel OD-H (n-hexane/i-PrOH = 90/10, flow rate: 1.0 mL/min, wave length = 220 nm)

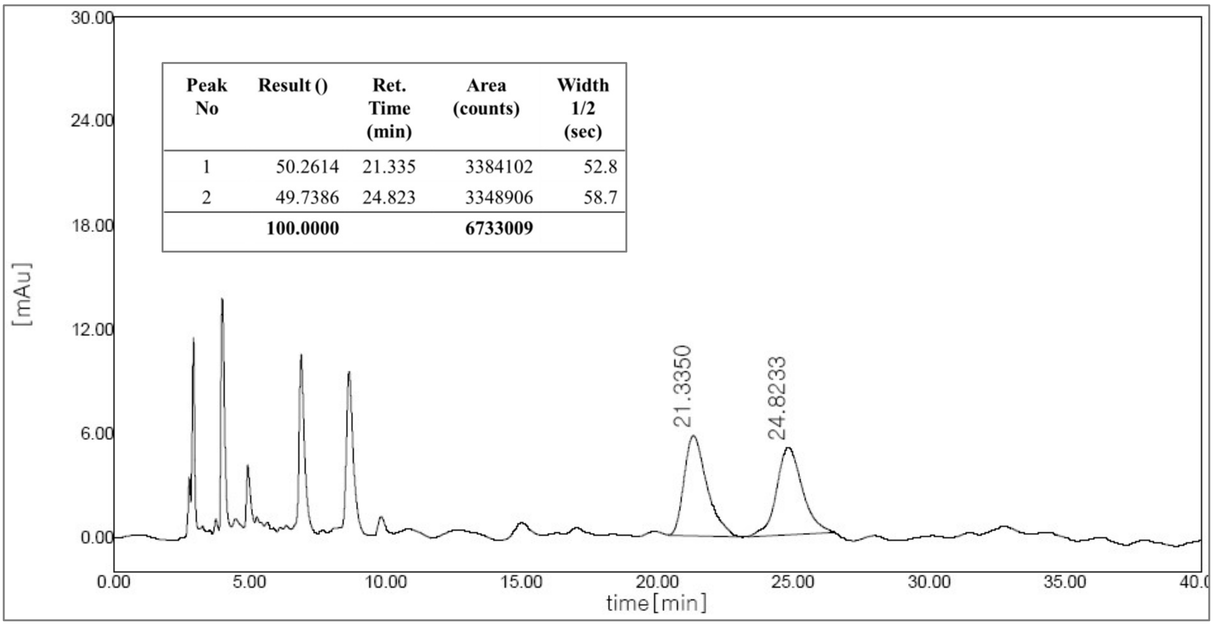

Peak No	Result ()	Ret. Time (min)	Area (counts)	Width 1/2 (sec)
1	50.2614	21.335	3384102	52.8
2	49.7386	24.823	3348906	58.7
	**100.0000**		**6733009**	

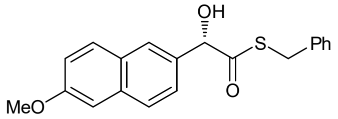

(*S*)-**5d**

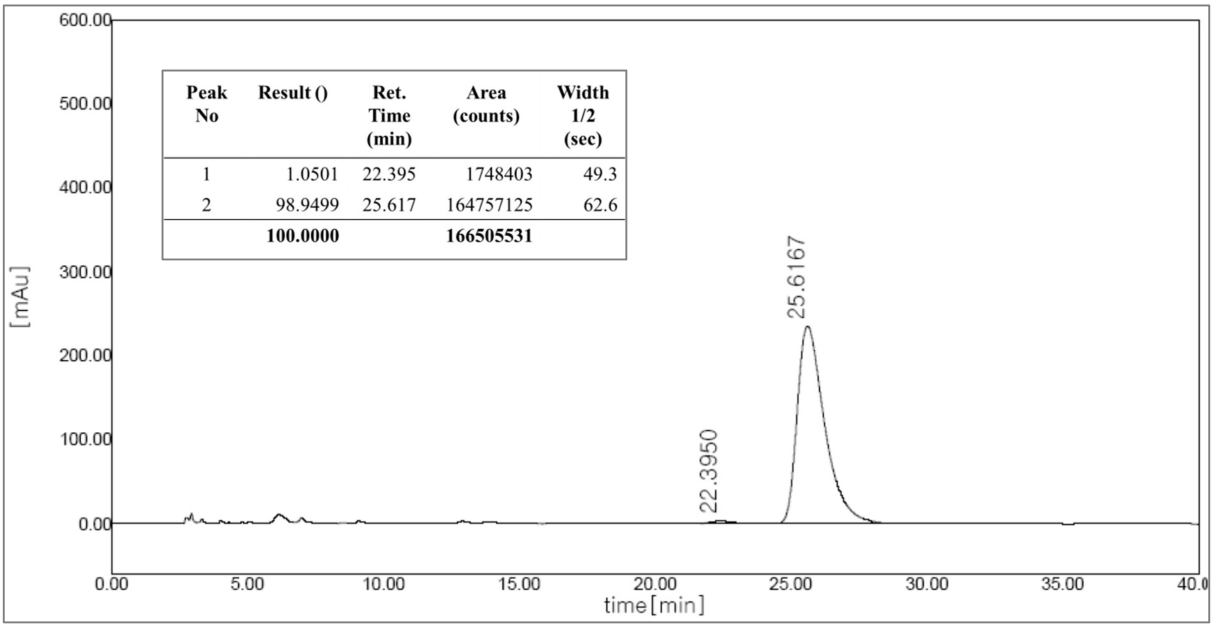

Peak No	Result ()	Ret. Time (min)	Area (counts)	Width 1/2 (sec)
1	1.0501	22.395	1748403	49.3
2	98.9499	25.617	164757125	62.6
	**100.0000**		**166505531**	

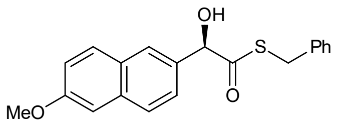

(*R*)-**5d**

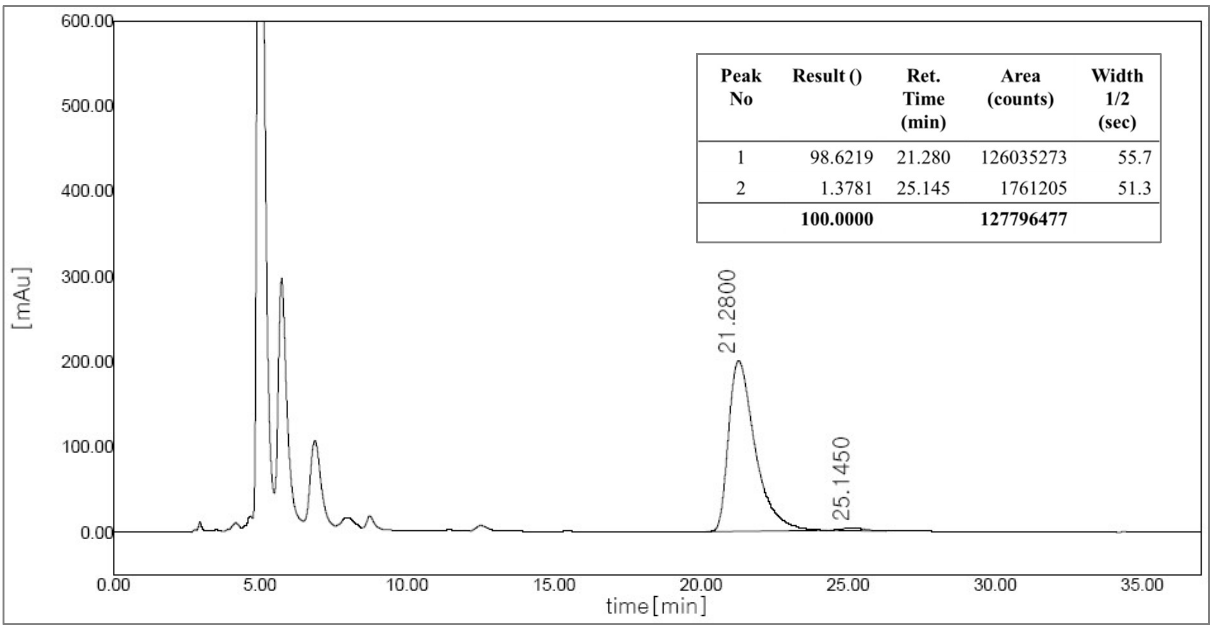

Peak No	Result ()	Ret. Time (min)	Area (counts)	Width 1/2 (sec)
1	98.6219	21.280	126035273	55.7
2	1.3781	25.145	1761205	51.3
	**100.0000**		**127796477**	

Supplementary Figure 65. HPLC spectra of compound **5e**.

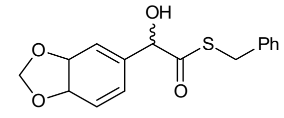

rac-**5e**

Chiralcel OD-H (n-hexane/i-PrOH = 95/5, flow rate: 1.0 mL/min, wave length = 220 nm)

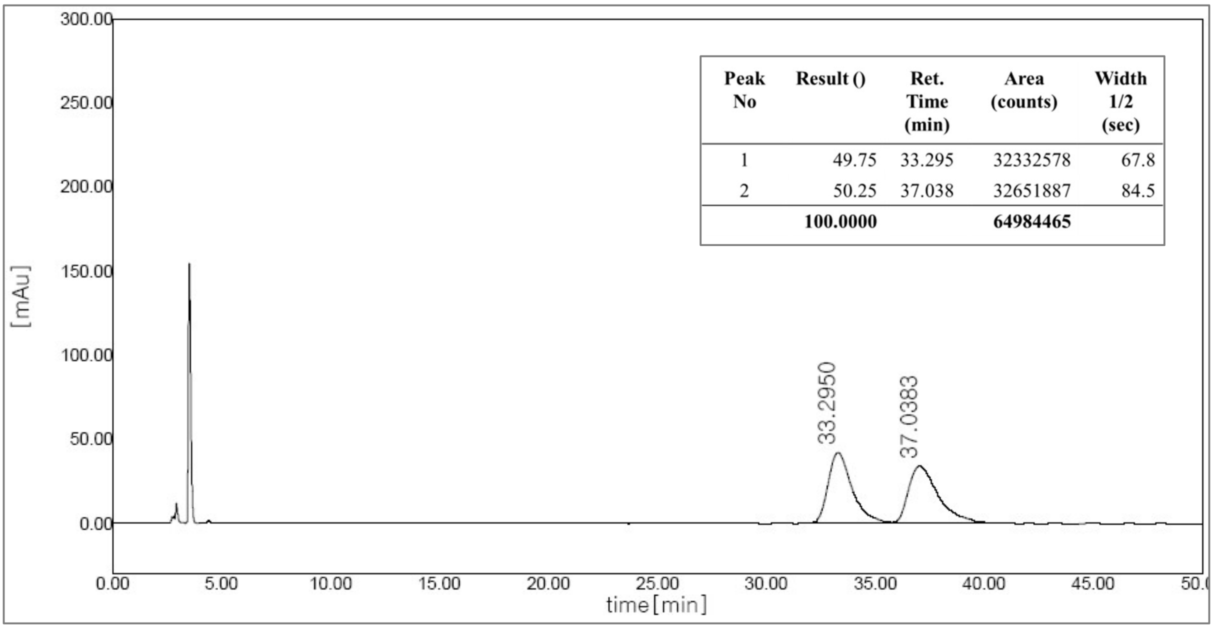

Peak No	Result ()	Ret. Time (min)	Area (counts)	Width 1/2 (sec)
1	49.75	33.295	32332578	67.8
2	50.25	37.038	32651887	84.5
	**100.0000**		**64984465**	

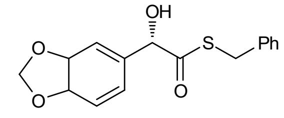

(*S*)-**5e**

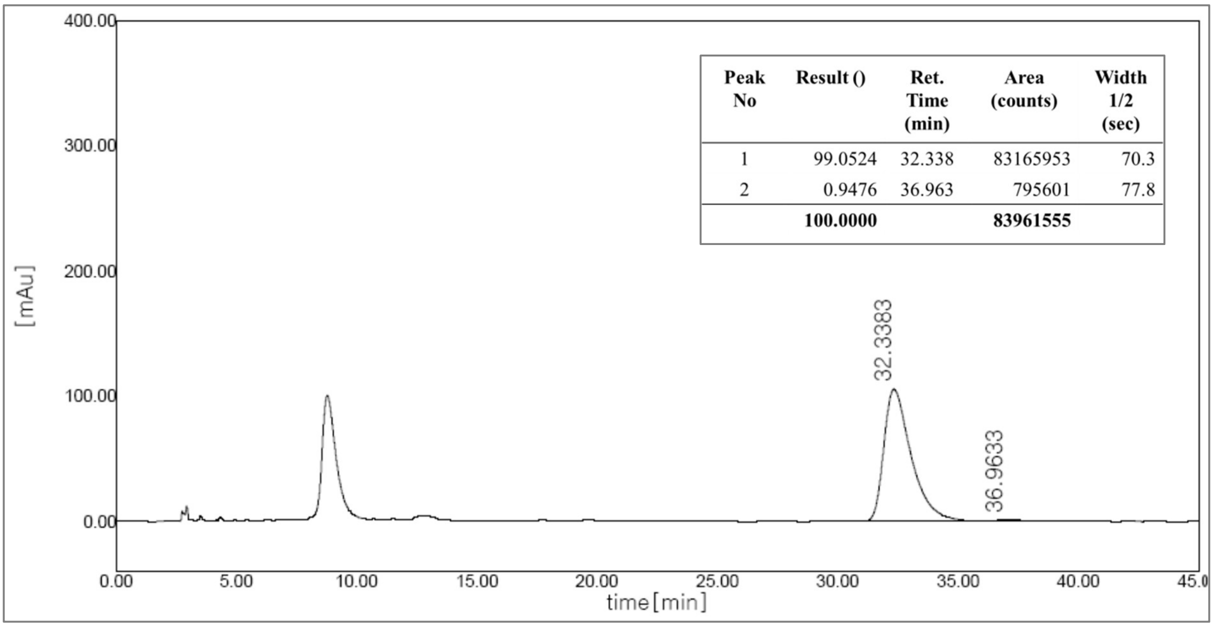

Peak No	Result ()	Ret. Time (min)	Area (counts)	Width 1/2 (sec)
1	99.0524	32.338	83165953	70.3
2	0.9476	36.963	795601	77.8
	**100.0000**		**83961555**	

Supplementary Figure 66. HPLC spectra of compound **5f**.

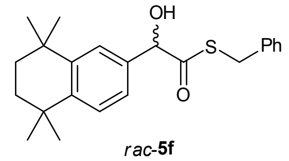

rac-**5f**

Chiralcel OD-H (n-hexane/i-PrOH = 99/1, flow rate: 1.0 mL/min, wave length = 220 nm)

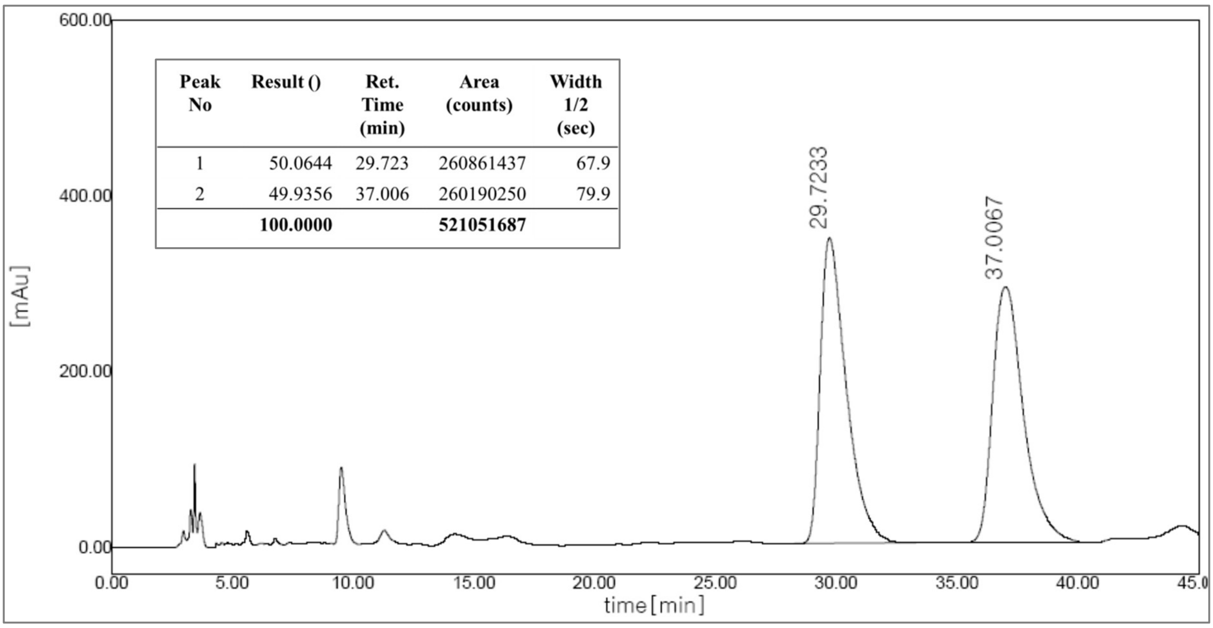

Peak No	Result ()	Ret. Time (min)	Area (counts)	Width 1/2 (sec)
1	50.0644	29.723	260861437	67.9
2	49.9356	37.006	260190250	79.9
	**100.0000**		**521051687**	

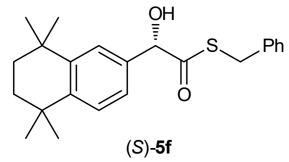

(*S*)-**5f**

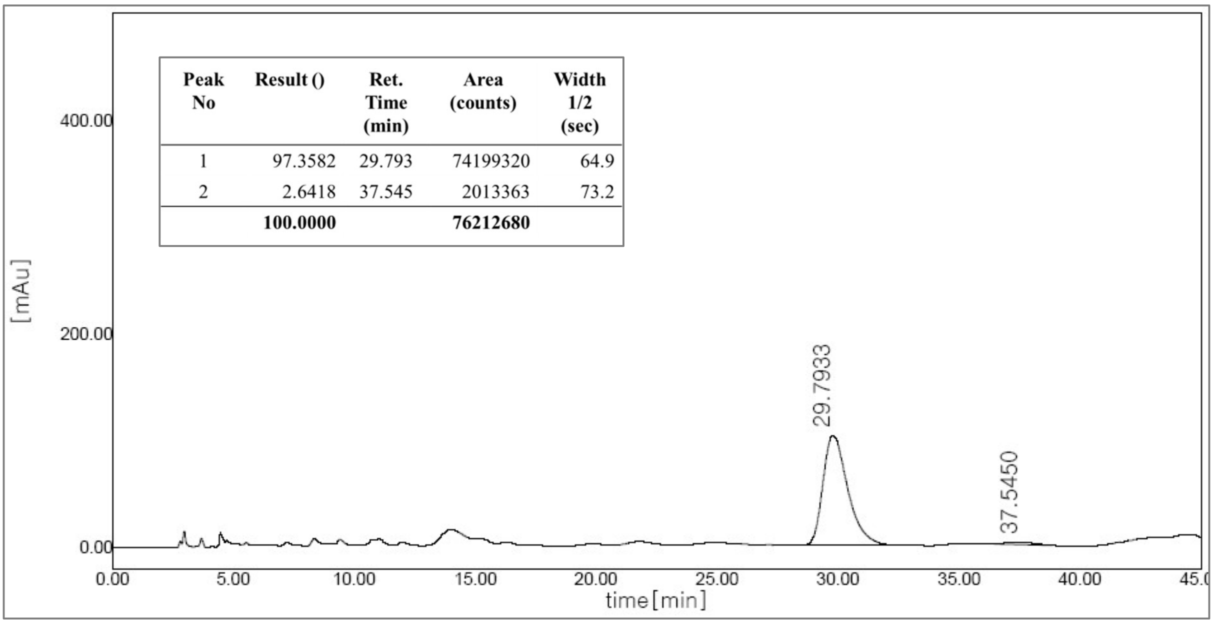

Peak No	Result ()	Ret. Time (min)	Area (counts)	Width 1/2 (sec)
1	97.3582	29.793	74199320	64.9
2	2.6418	37.545	2013363	73.2
	**100.0000**		**76212680**	

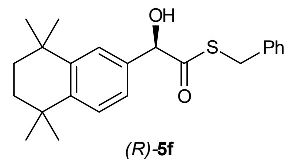

(R)-**5f**

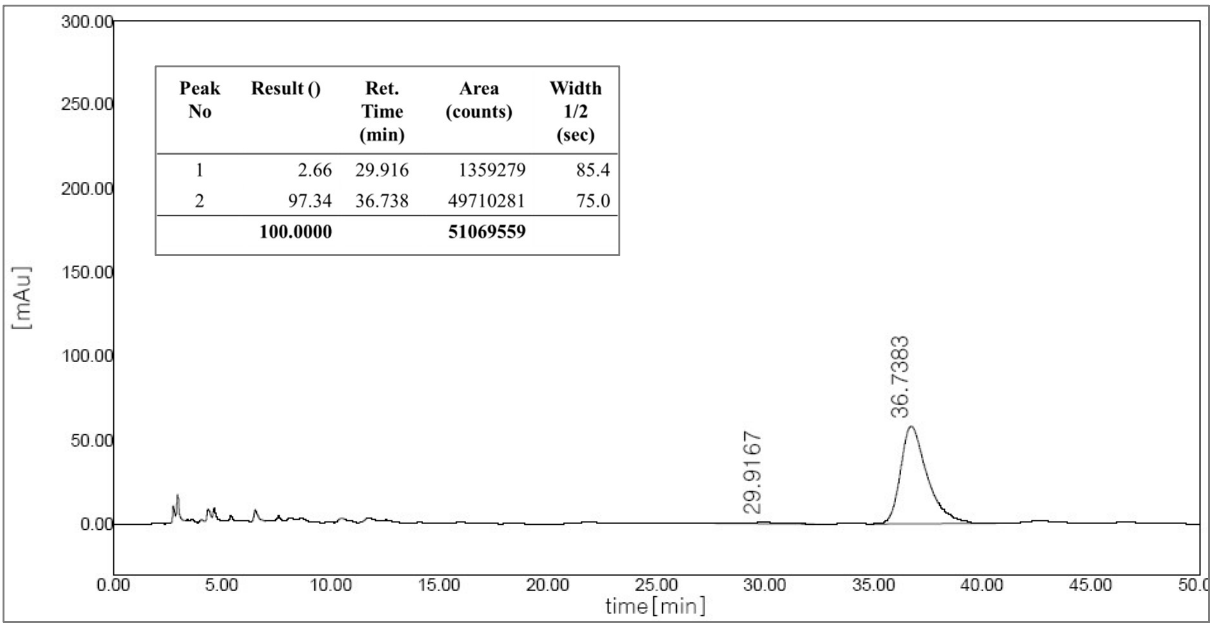

Peak No	Result ()	Ret. Time (min)	Area (counts)	Width 1/2 (sec)
1	2.66	29.916	1359279	85.4
2	97.34	36.738	49710281	75.0
	**100.0000**		**51069559**	

Supplementary Figure 67. HPLC spectra of compound **5g**.

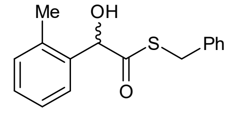

rac-**5g**

Chiralpak AD-H (n-hexane/i-PrOH = 96/4, flow rate: 1.0 mL/min, wave length = 220 nm)

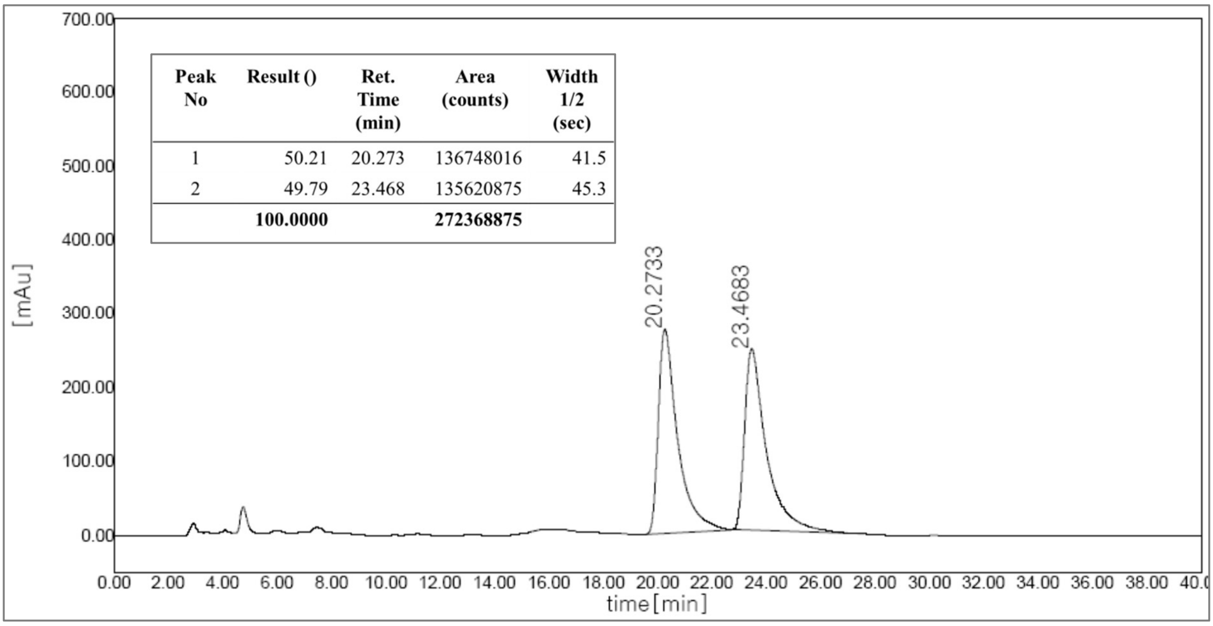

Peak No	Result ()	Ret. Time (min)	Area (counts)	Width 1/2 (sec)
1	50.21	20.273	136748016	41.5
2	49.79	23.468	135620875	45.3
	**100.0000**		**272368875**	

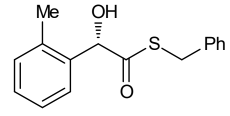

(*S*)-**5g**

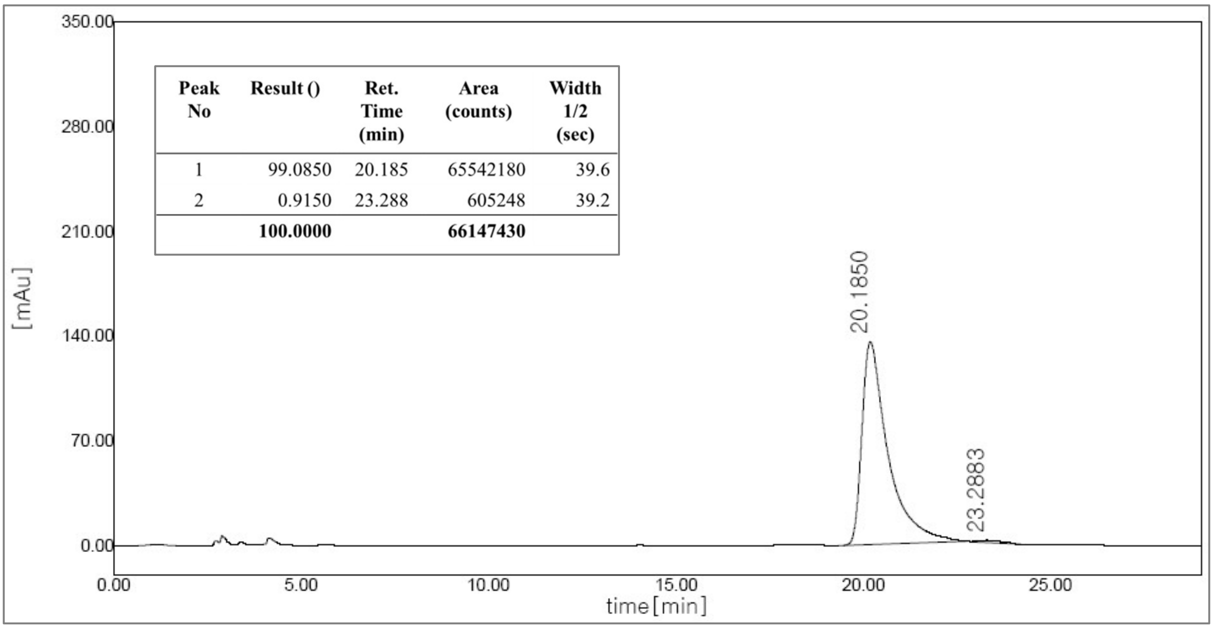

Peak No	Result ()	Ret. Time (min)	Area (counts)	Width 1/2 (sec)
1	99.0850	20.185	65542180	39.6
2	0.9150	23.288	605248	39.2
	**100.0000**		**66147430**	

Supplementary Figure 68. HPLC spectra of compound **5h**.

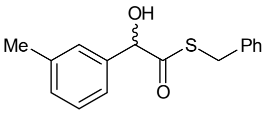

rac-**5h**

Chiralpak AD-H (n-hexane/i-PrOH = 96/4, flow rate: 1.0 mL/min, wave length = 220 nm)

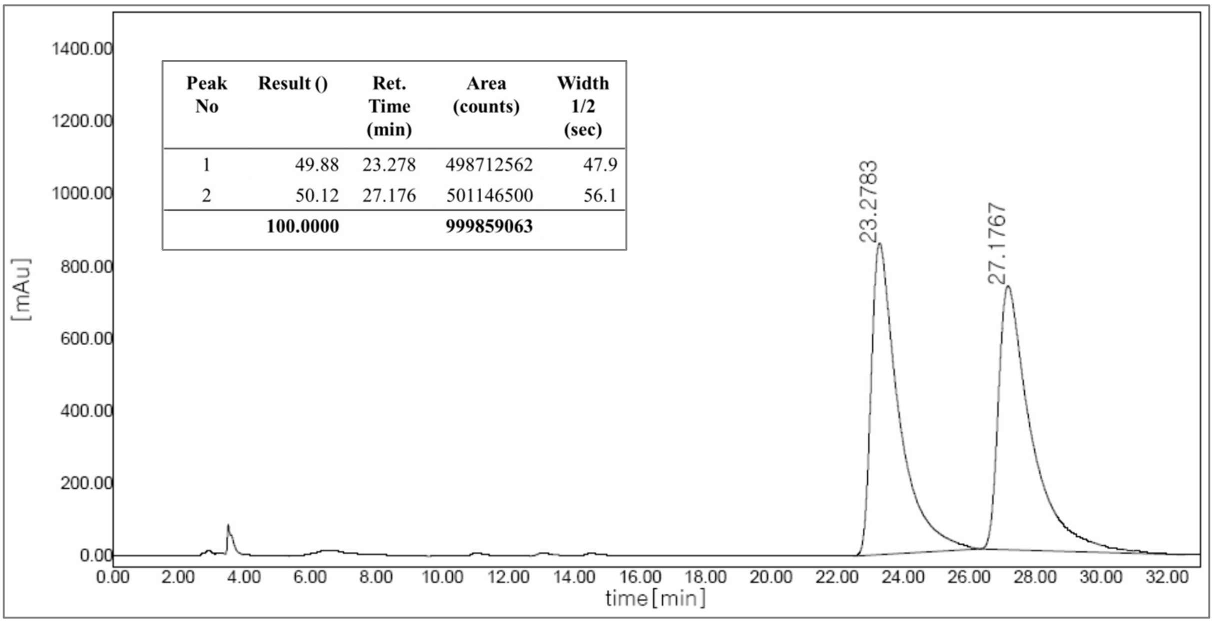

Peak No	Result ()	Ret. Time (min)	Area (counts)	Width 1/2 (sec)
1	49.88	23.278	498712562	47.9
2	50.12	27.176	501146500	56.1
	**100.0000**		**999859063**	

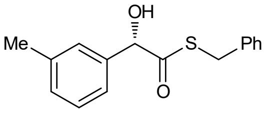

(*S*)-**5h**

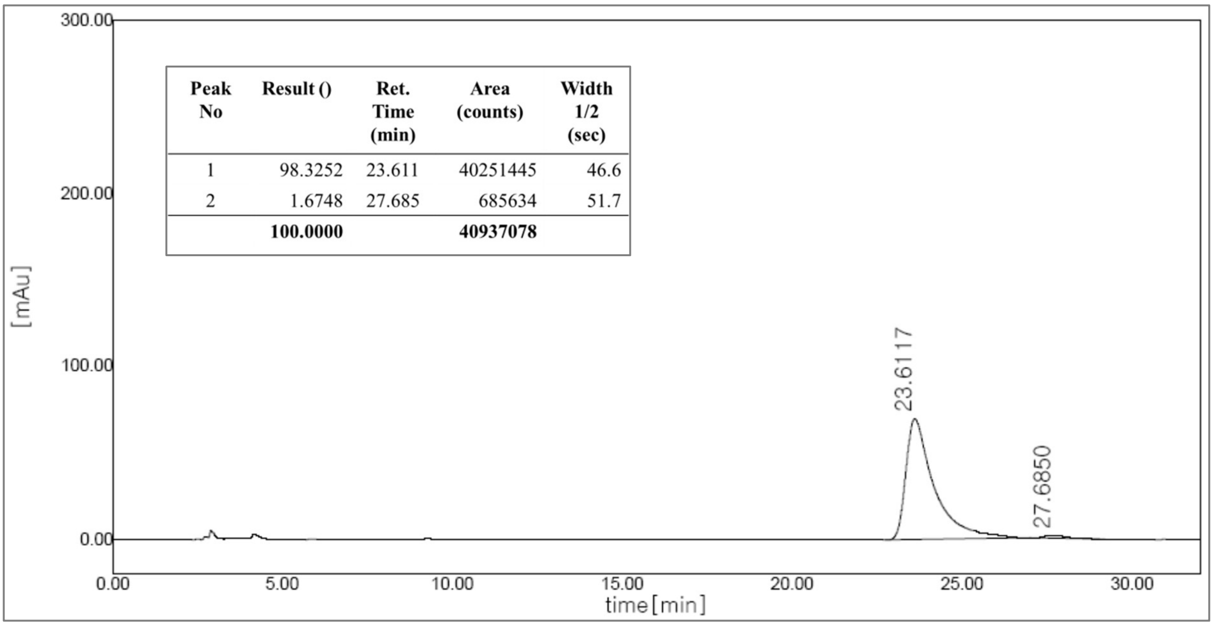

Peak No	Result ()	Ret. Time (min)	Area (counts)	Width 1/2 (sec)
1	98.3252	23.611	40251445	46.6
2	1.6748	27.685	685634	51.7
	**100.0000**		**40937078**	

Supplementary Figure 69. HPLC spectra of compound **5i**.

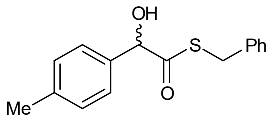

rac-**5i**

Chiralpak AD-H (n-hexane/i-PrOH = 96/4, flow rate: 1.0 mL/min, wave length = 220 nm)

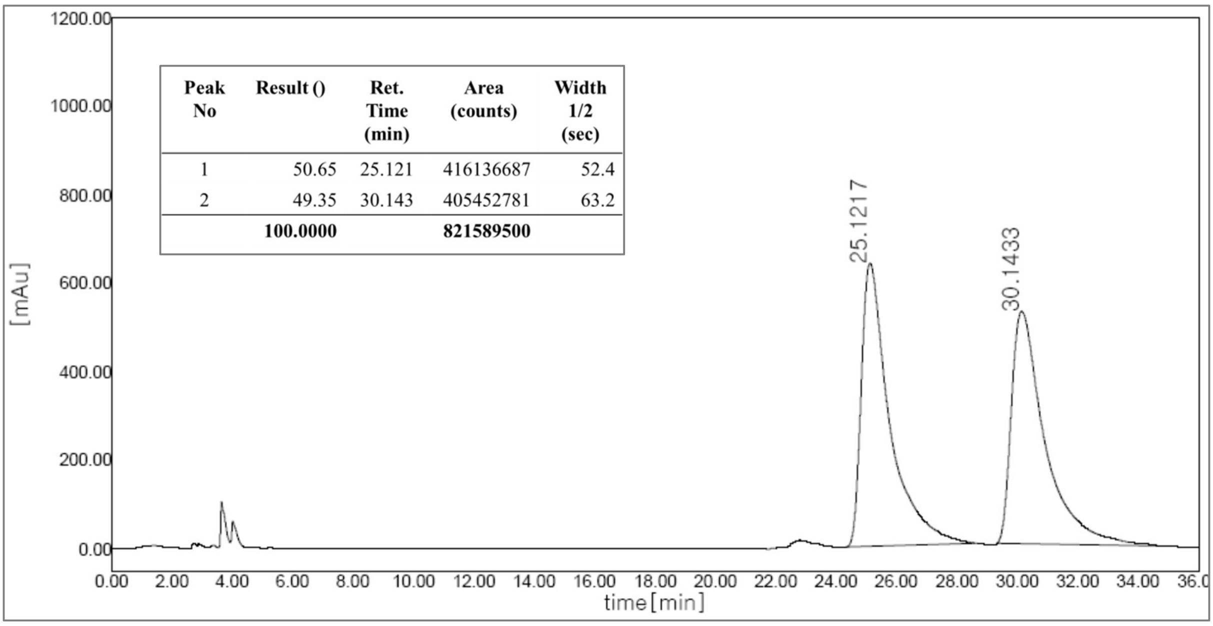

Peak No	Result ()	Ret. Time (min)	Area (counts)	Width 1/2 (sec)
1	50.65	25.121	416136687	52.4
2	49.35	30.143	405452781	63.2
	**100.0000**		**821589500**	

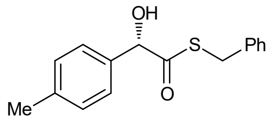

(*S*)-**5i**

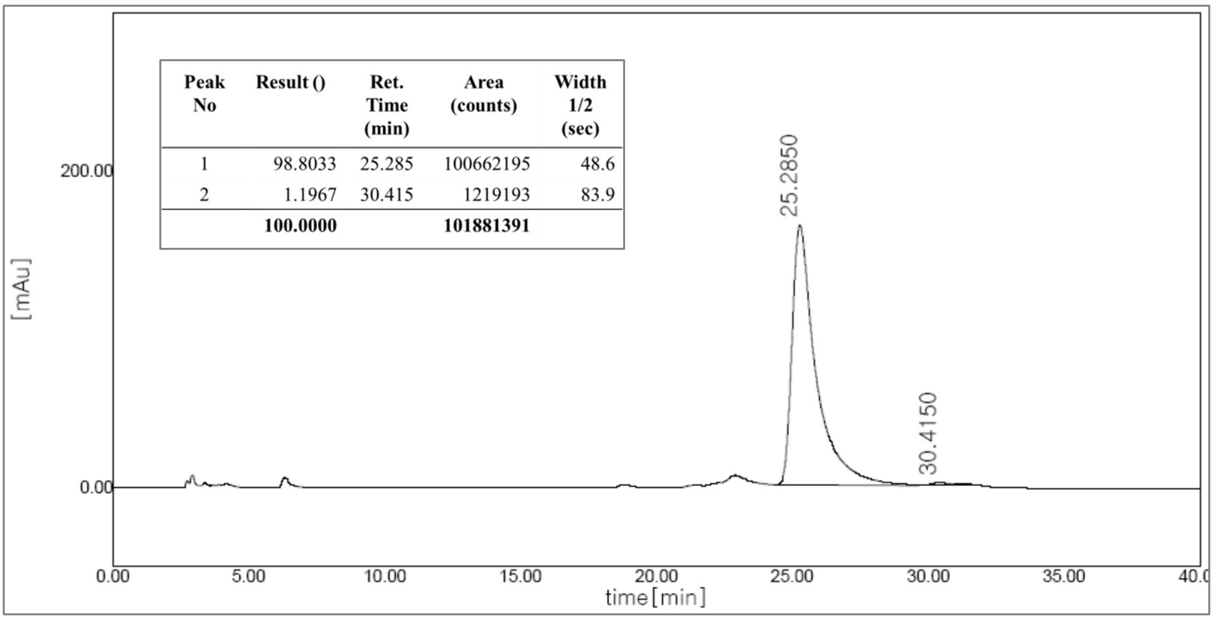

Peak No	Result ()	Ret. Time (min)	Area (counts)	Width 1/2 (sec)
1	98.8033	25.285	100662195	48.6
2	1.1967	30.415	1219193	83.9
	**100.0000**		**101881391**	

Supplementary Figure 70. HPLC spectra of compound **5j**.

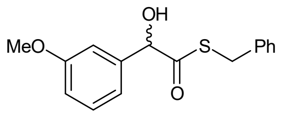

rac-**5j**

Chiralpak AD-H (n-hexane/i-PrOH = 96/4, flow rate: 1.0 mL/min, wave length = 220 nm)

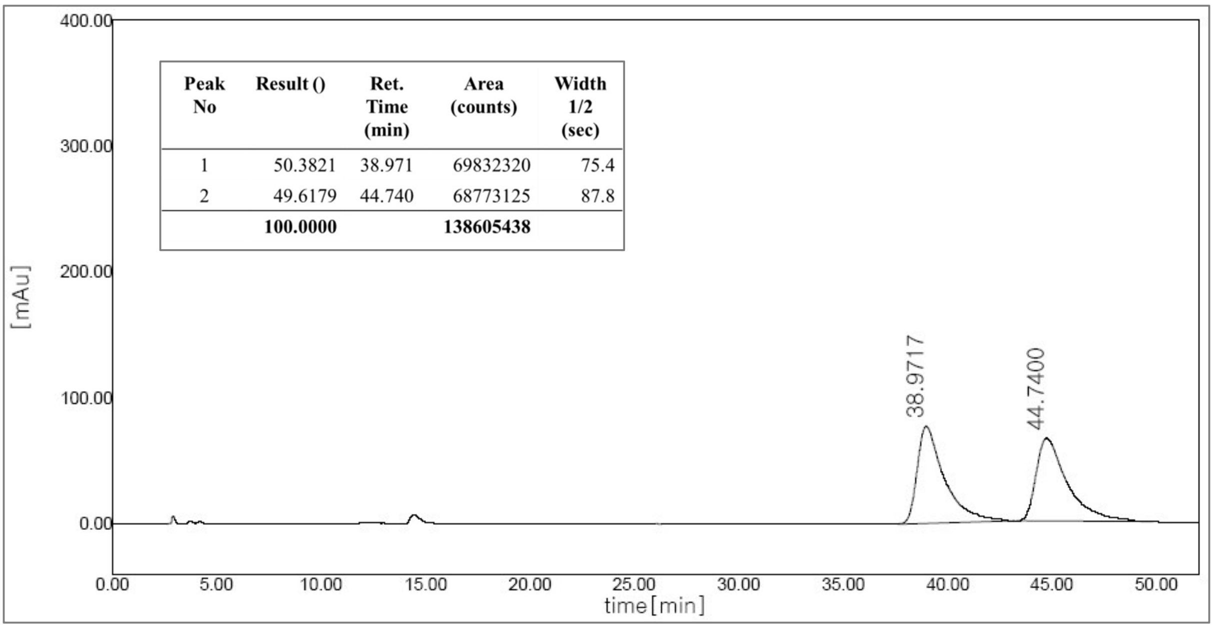

Peak No	Result ()	Ret. Time (min)	Area (counts)	Width 1/2 (sec)
1	50.3821	38.971	69832320	75.4
2	49.6179	44.740	68773125	87.8
	**100.0000**		**138605438**	

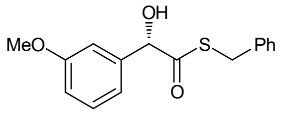

(*S*)-**5j**

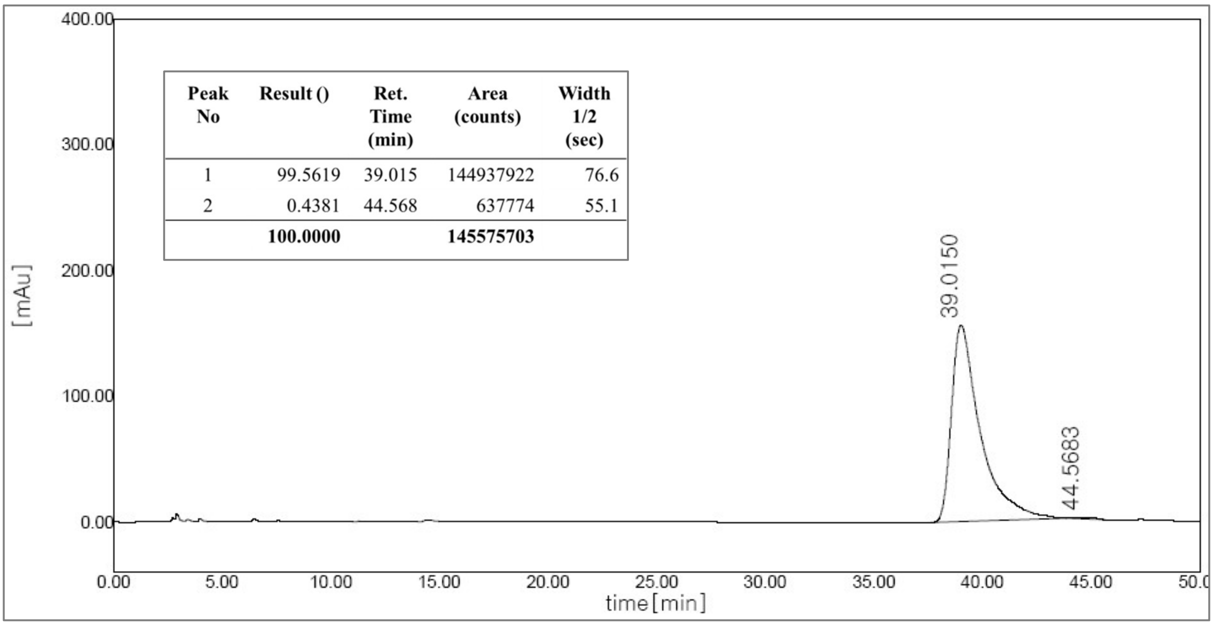

Peak No	Result ()	Ret. Time (min)	Area (counts)	Width 1/2 (sec)
1	99.5619	39.015	144937922	76.6
2	0.4381	44.568	637774	55.1
	**100.0000**		**145575703**	

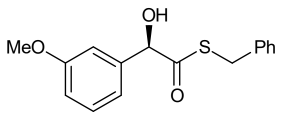

(*R*)-**5j**

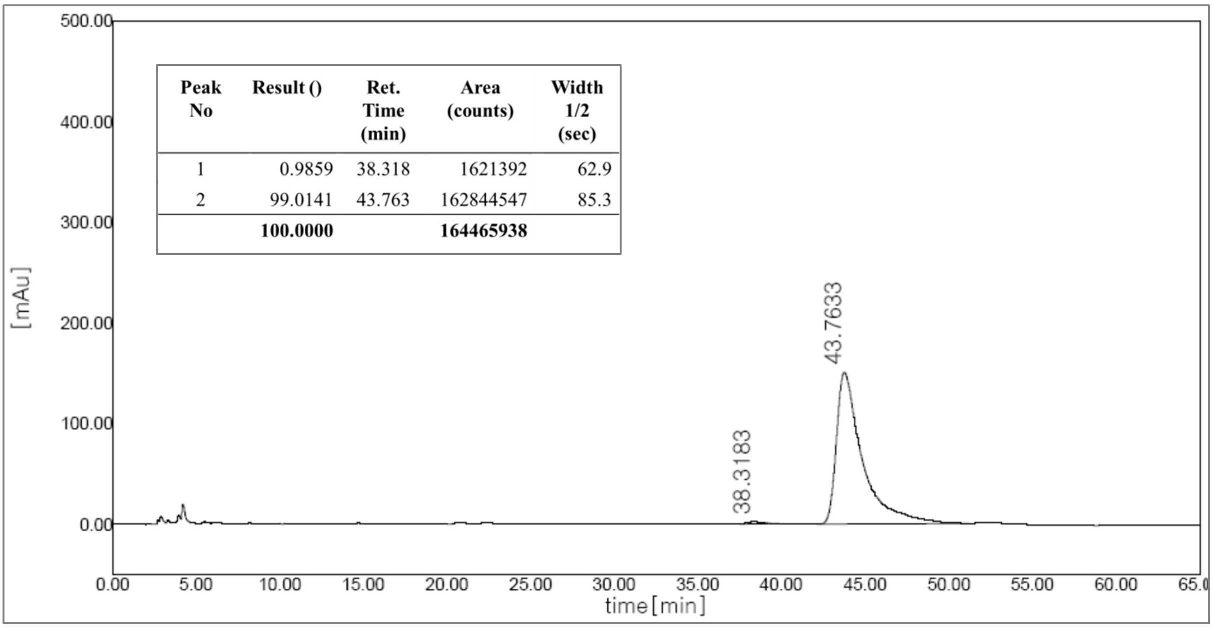

Peak No	Result ()	Ret. Time (min)	Area (counts)	Width 1/2 (sec)
1	0.9859	38.318	1621392	62.9
2	99.0141	43.763	162844547	85.3
	**100.0000**		**164465938**	

Supplementary Figure 71. HPLC spectra of compound **5k**.

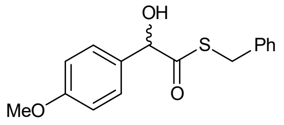

rac-**5k**

Chiralcel OD-H (n-hexane/i-PrOH = 95/5, flow rate: 1.0 mL/min, wave length = 220 nm)

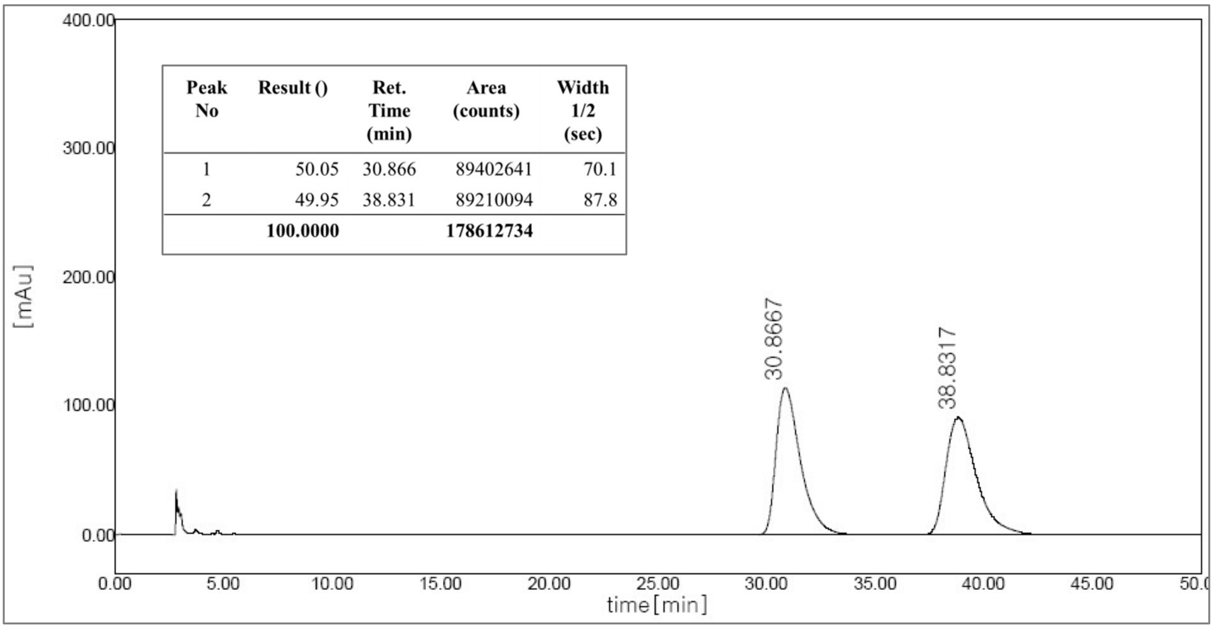

Peak No	Result ()	Ret. Time (min)	Area (counts)	Width 1/2 (sec)
1	50.05	30.866	89402641	70.1
2	49.95	38.831	89210094	87.8
	**100.0000**		**178612734**	

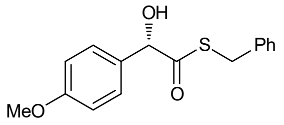

(*S*)-**5k**

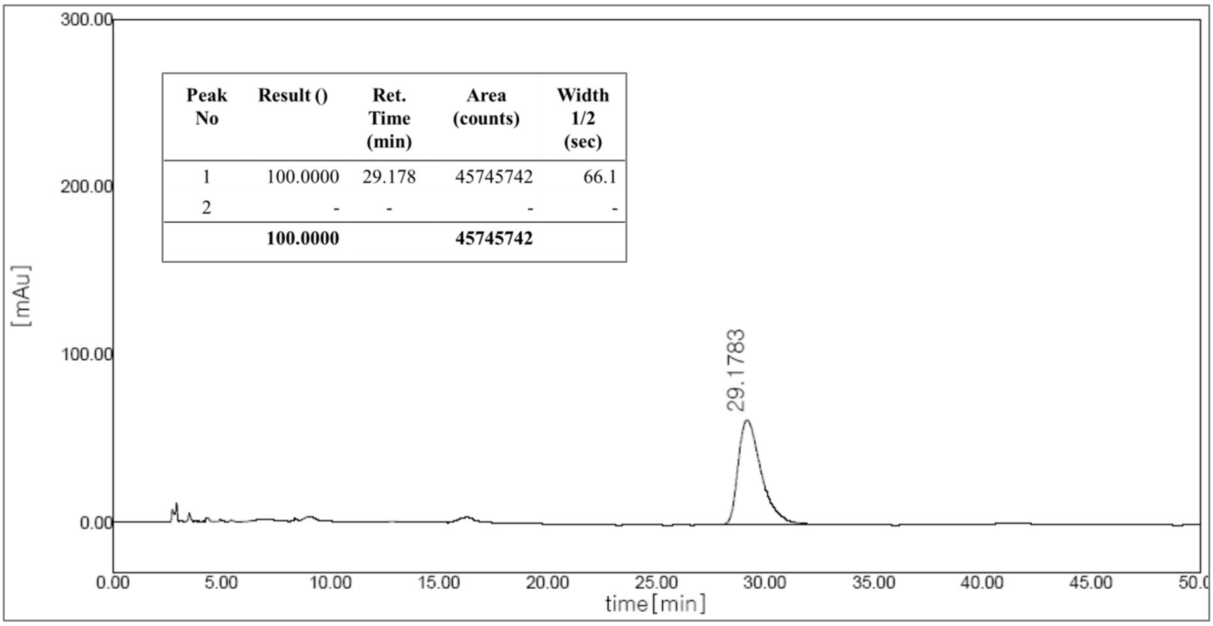

Peak No	Result ()	Ret. Time (min)	Area (counts)	Width 1/2 (sec)
1	100.0000	29.178	45745742	66.1
2	-	-	-	-
	**100.0000**		**45745742**	

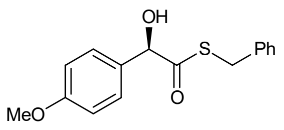

(*R*)-**5k**

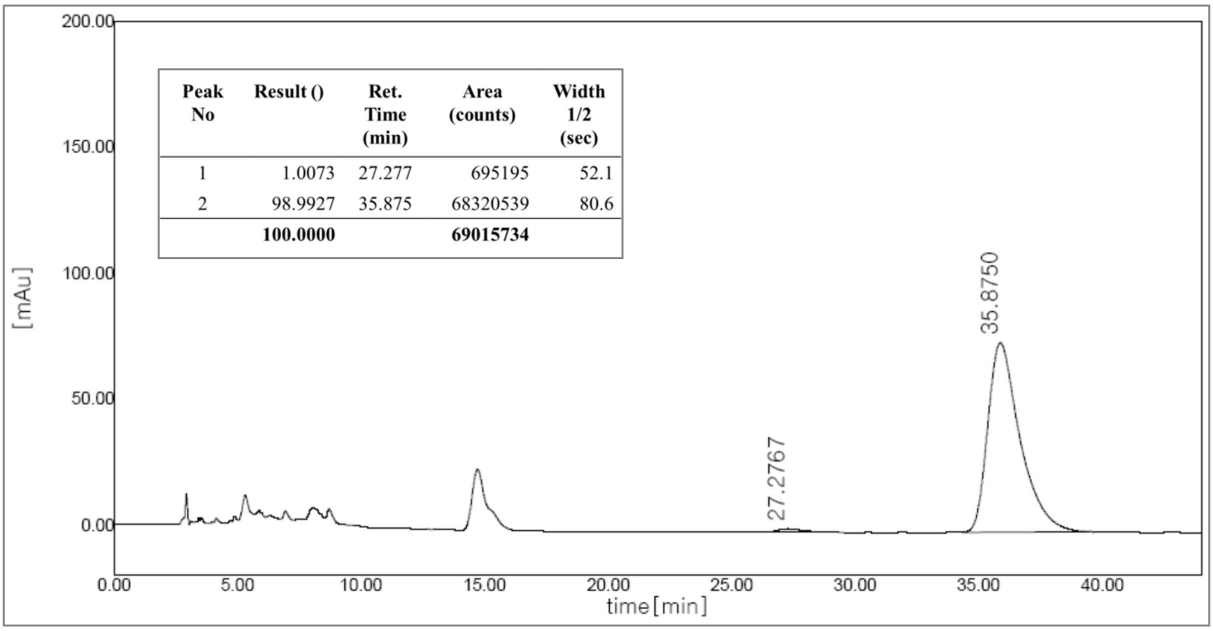

Peak No	Result ()	Ret. Time (min)	Area (counts)	Width 1/2 (sec)
1	1.0073	27.277	695195	52.1
2	98.9927	35.875	68320539	80.6
	**100.0000**		**69015734**	

Supplementary Figure 72. HPLC spectra of compound **5l**.

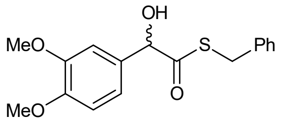

rac-**5l**

Chiralpak AD-H (n-hexane/i-PrOH = 85/15, flow rate: 1.0 mL/min, wave length = 220 nm)

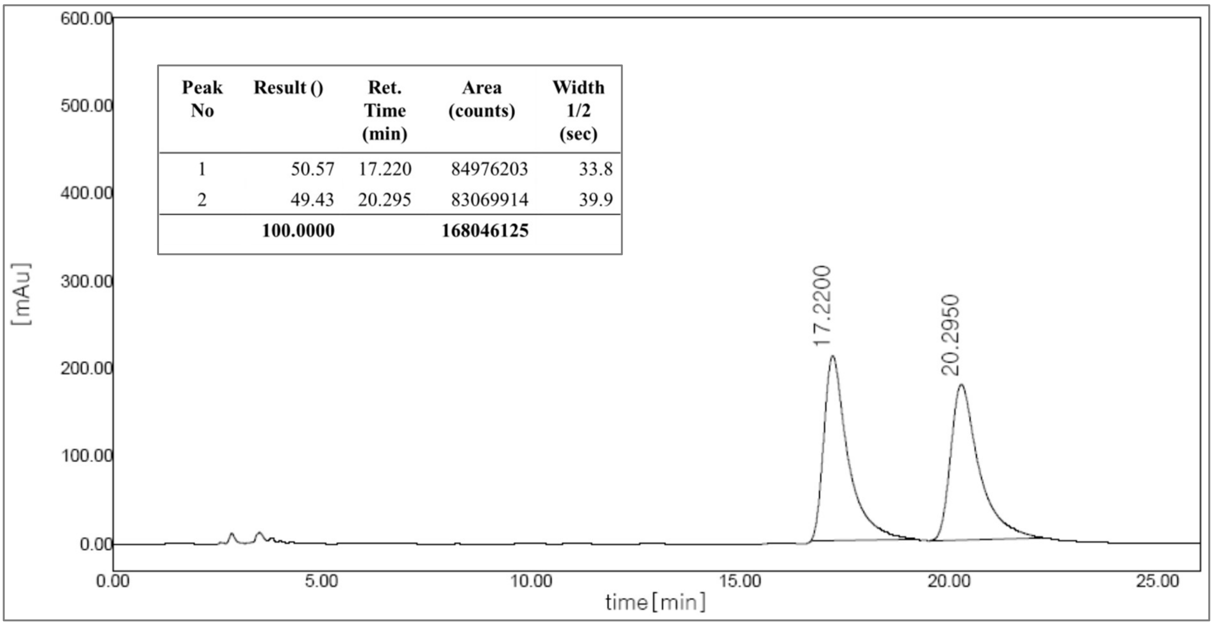

Peak No	Result ()	Ret. Time (min)	Area (counts)	Width 1/2 (sec)
1	50.57	17.220	84976203	33.8
2	49.43	20.295	83069914	39.9
	**100.0000**		**168046125**	

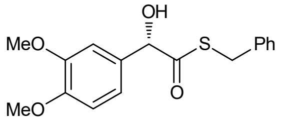

(*S*)-**5l**

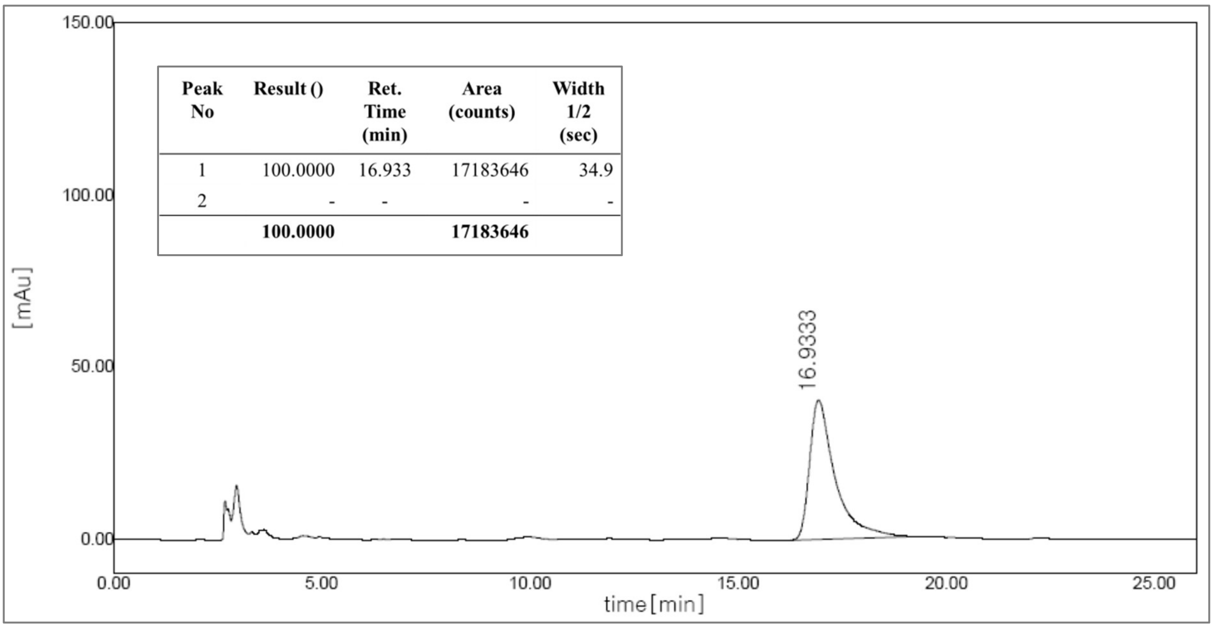

Peak No	Result ()	Ret. Time (min)	Area (counts)	Width 1/2 (sec)
1	100.0000	16.933	17183646	34.9
2	-	-	-	-
	**100.0000**		**17183646**	

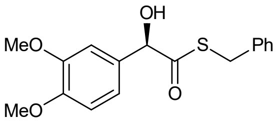

(*R*)-**5l**

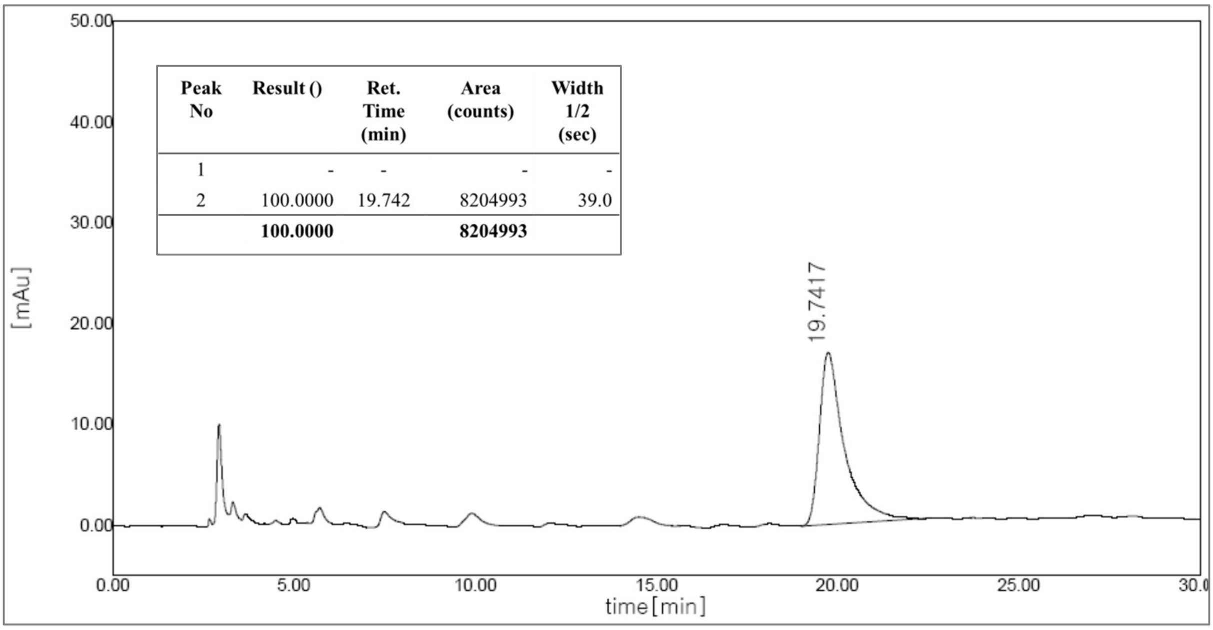

Peak No	Result ()	Ret. Time (min)	Area (counts)	Width 1/2 (sec)
1	-	-	-	-
2	100.0000	19.742	8204993	39.0
	**100.0000**		**8204993**	

Supplementary Figure 73. HPLC spectra of compound **5m**.

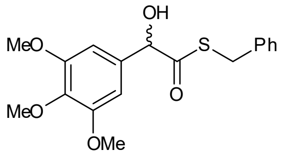

rac-**5m**

Chiralpak AD-H (n-hexane/i-PrOH = 85/15, flow rate: 1.0 mL/min, wave length = 220 nm)

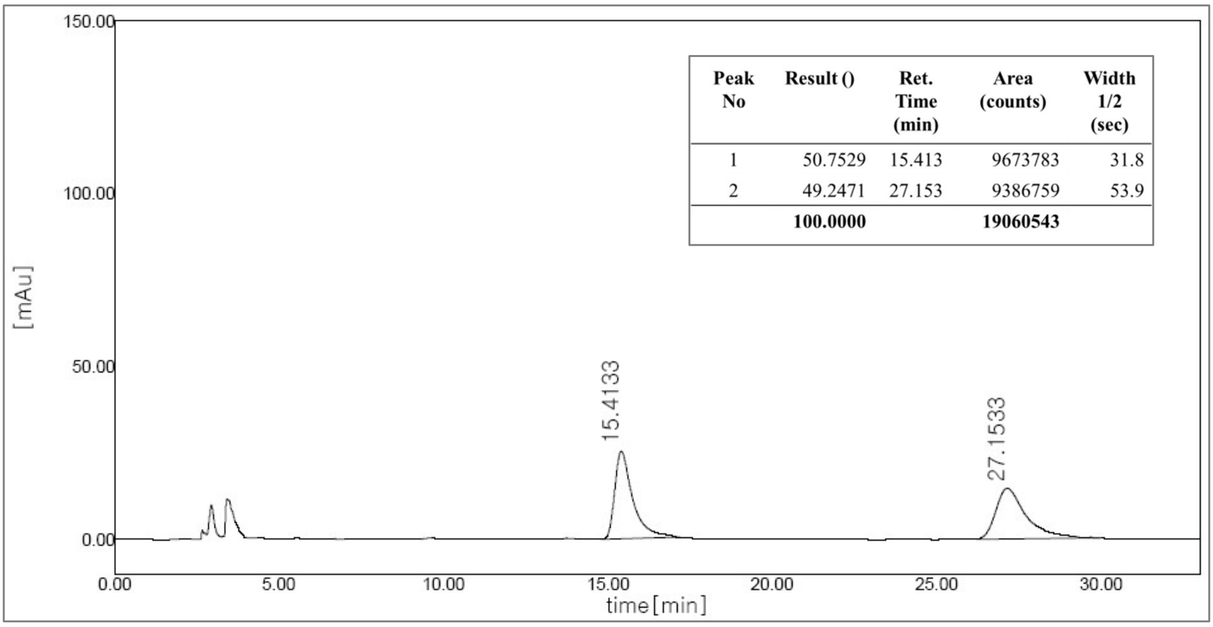

Peak No	Result ()	Ret. Time (min)	Area (counts)	Width 1/2 (sec)
1	50.7529	15.413	9673783	31.8
2	49.2471	27.153	9386759	53.9
	**100.0000**		**19060543**	

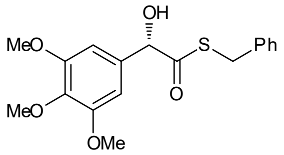

(*S*)-**5m**

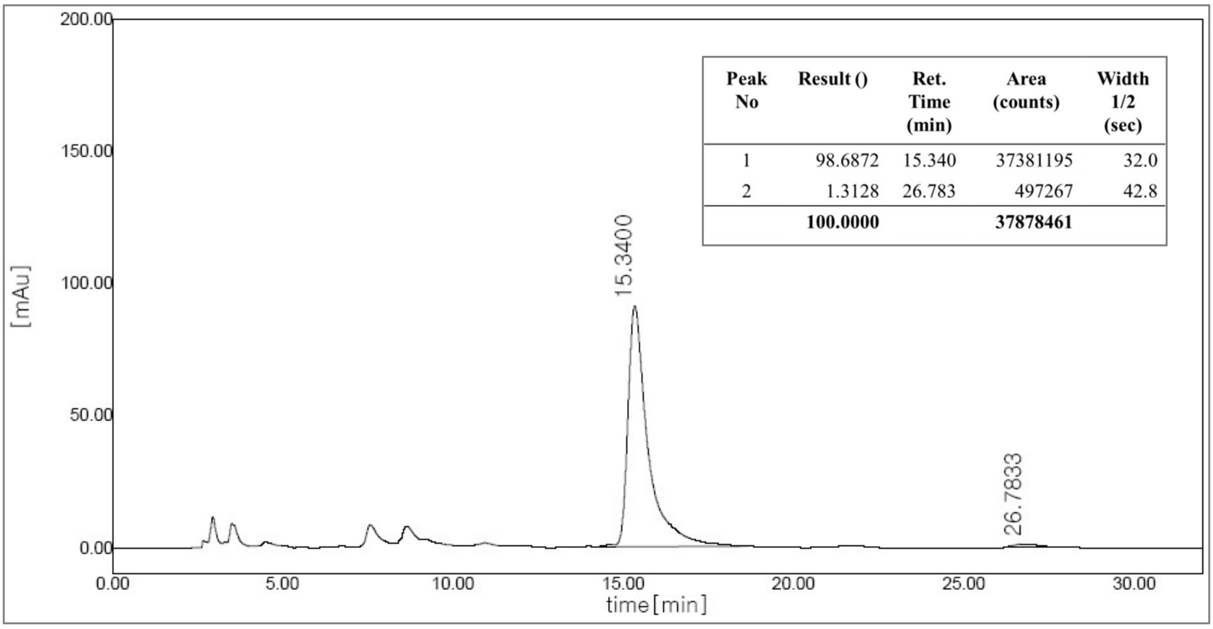

Peak No	Result ()	Ret. Time (min)	Area (counts)	Width 1/2 (sec)
1	98.6872	15.340	37381195	32.0
2	1.3128	26.783	497267	42.8
	**100.0000**		**37878461**	

Supplementary Figure 74. HPLC spectra of compound **5n**.

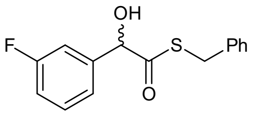

rac-**5n**

Chiralcel OD-H (n-hexane/i-PrOH = 95/5, flow rate: 1.0 mL/min, wave length = 220 nm)

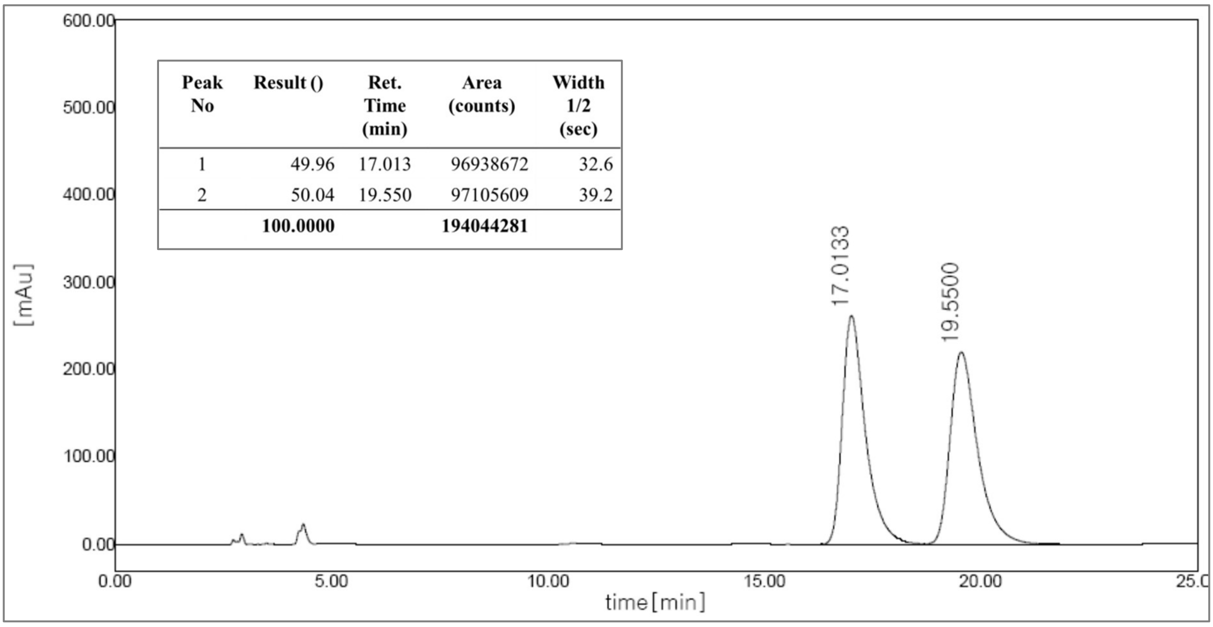

Peak No	Result ()	Ret. Time (min)	Area (counts)	Width 1/2 (sec)
1	49.96	17.013	96938672	32.6
2	50.04	19.550	97105609	39.2
	**100.0000**		**194044281**	

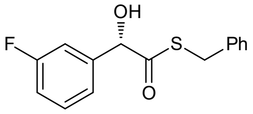

(*S*)-**5n**

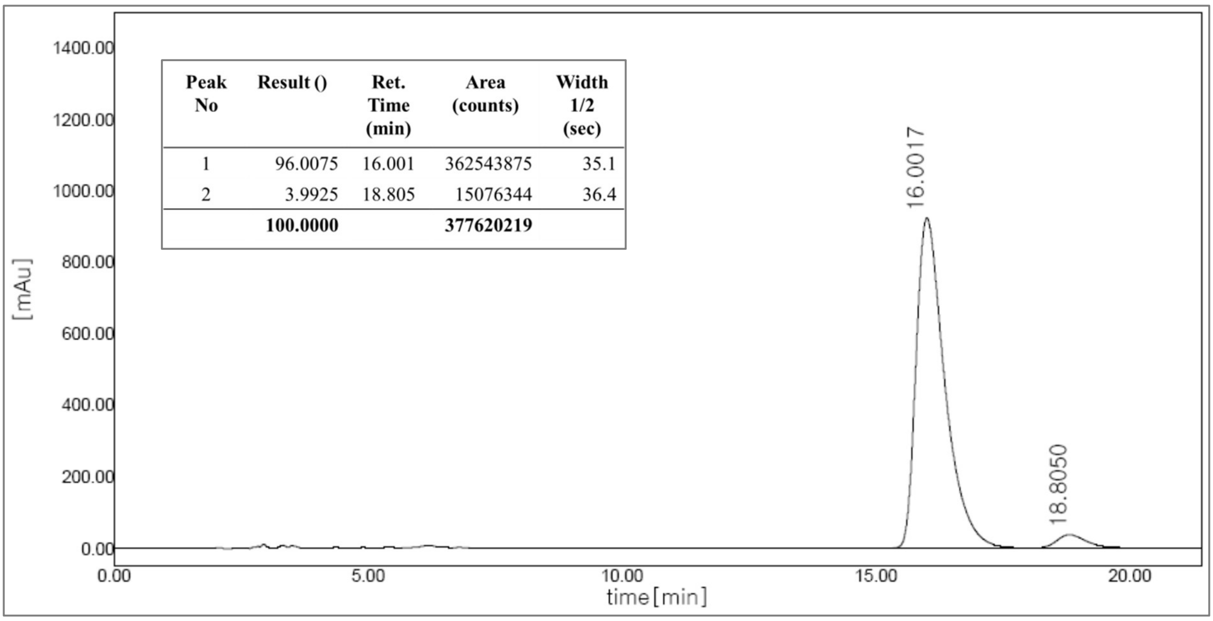

Peak No	Result ()	Ret. Time (min)	Area (counts)	Width 1/2 (sec)
1	96.0075	16.001	362543875	35.1
2	3.9925	18.805	15076344	36.4
	**100.0000**		**377620219**	

Supplementary Figure 75. HPLC spectra of compound **5o**.

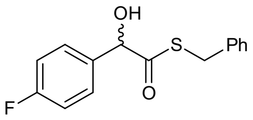

rac-**5o**

Chiralpak AD-H (n-hexane/i-PrOH = 96/4, flow rate: 1.0 mL/min, wave length = 220 nm)

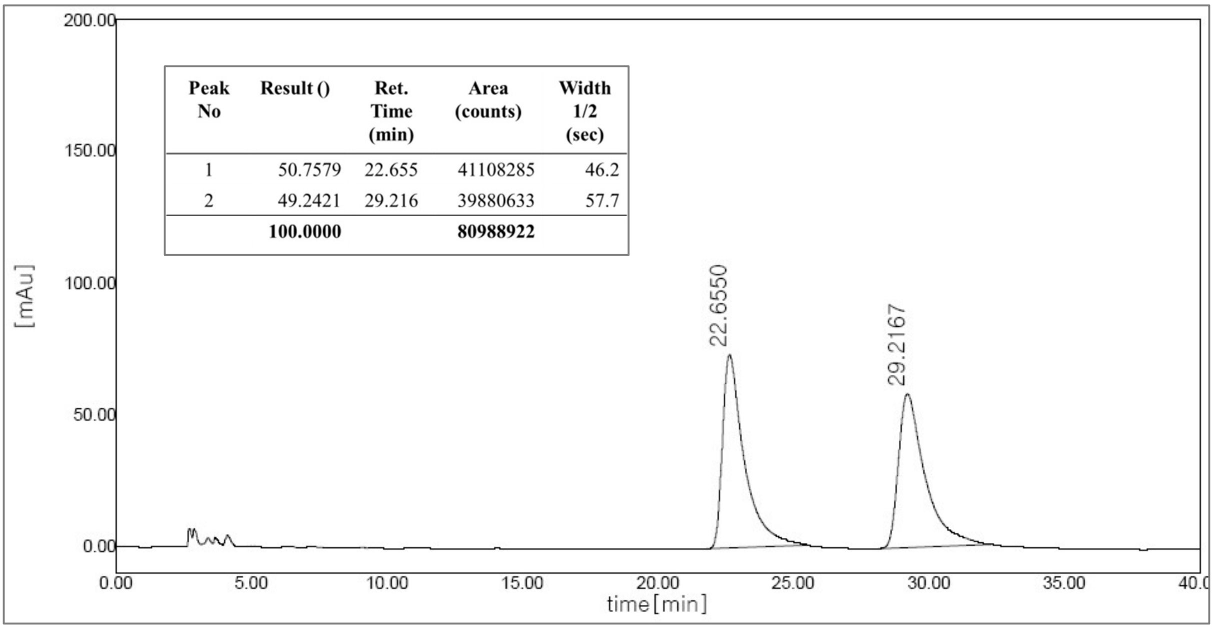

Peak No	Result ()	Ret. Time (min)	Area (counts)	Width 1/2 (sec)
1	50.7579	22.655	41108285	46.2
2	49.2421	29.216	39880633	57.7
	**100.0000**		**80988922**	

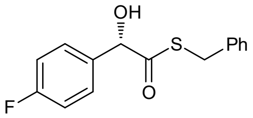

(*S*)-**5o**

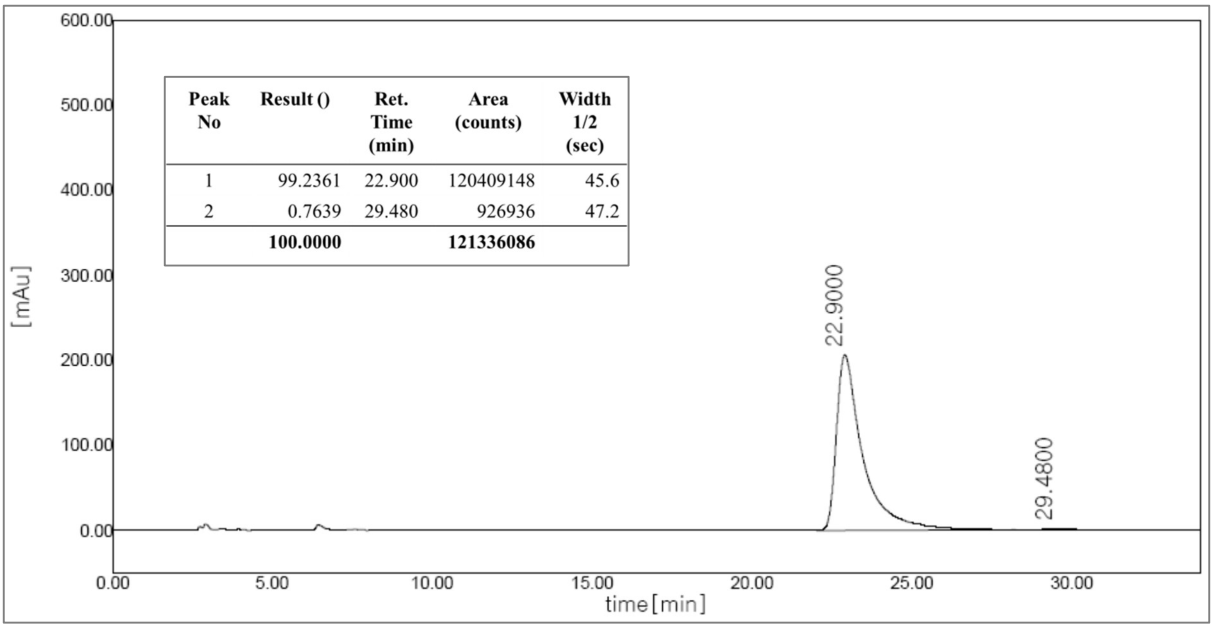

Peak No	Result ()	Ret. Time (min)	Area (counts)	Width 1/2 (sec)
1	99.2361	22.900	120409148	45.6
2	0.7639	29.480	926936	47.2
	**100.0000**		**121336086**	

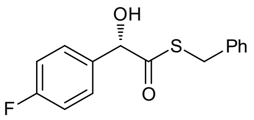

(*S*)-**5o**

using 5 mol% of cat **1d**

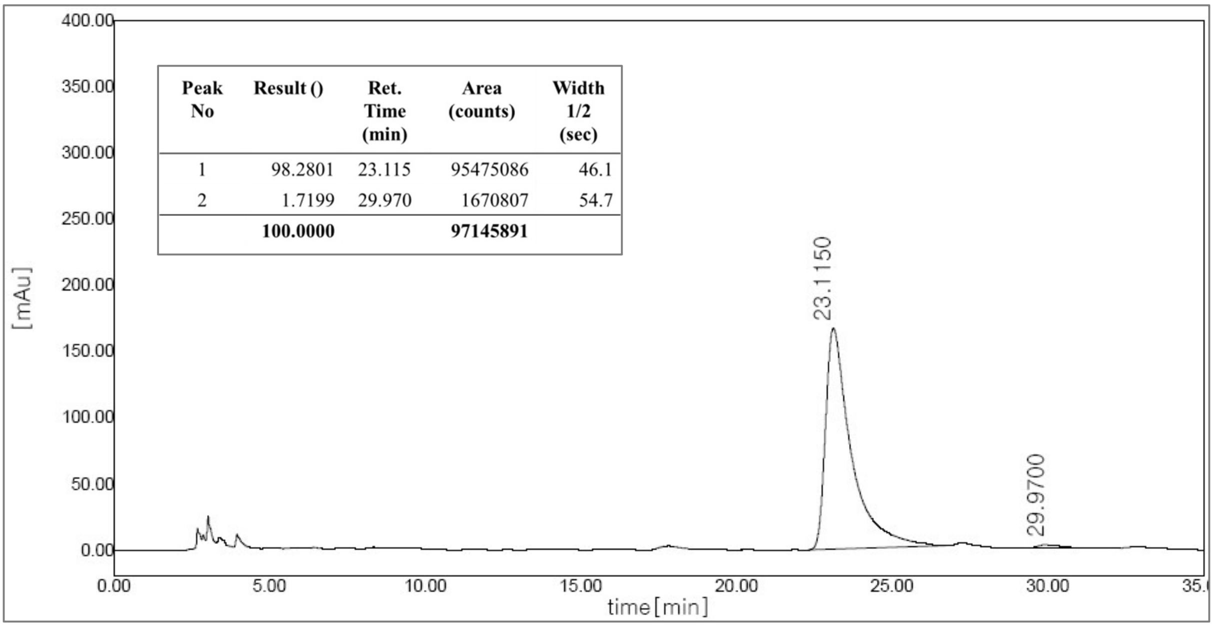

Peak No	Result ()	Ret. Time (min)	Area (counts)	Width 1/2 (sec)
1	98.2801	23.115	95475086	46.1
2	1.7199	29.970	1670807	54.7
	**100.0000**		**97145891**	

Supplementary Figure 76. HPLC spectra of compound **5p**.

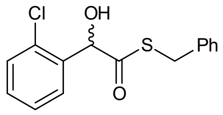

rac-**5p**

Chiralpak AD-H (n-hexane/i-PrOH = 96/4, flow rate: 1.0 mL/min, wave length = 220 nm)

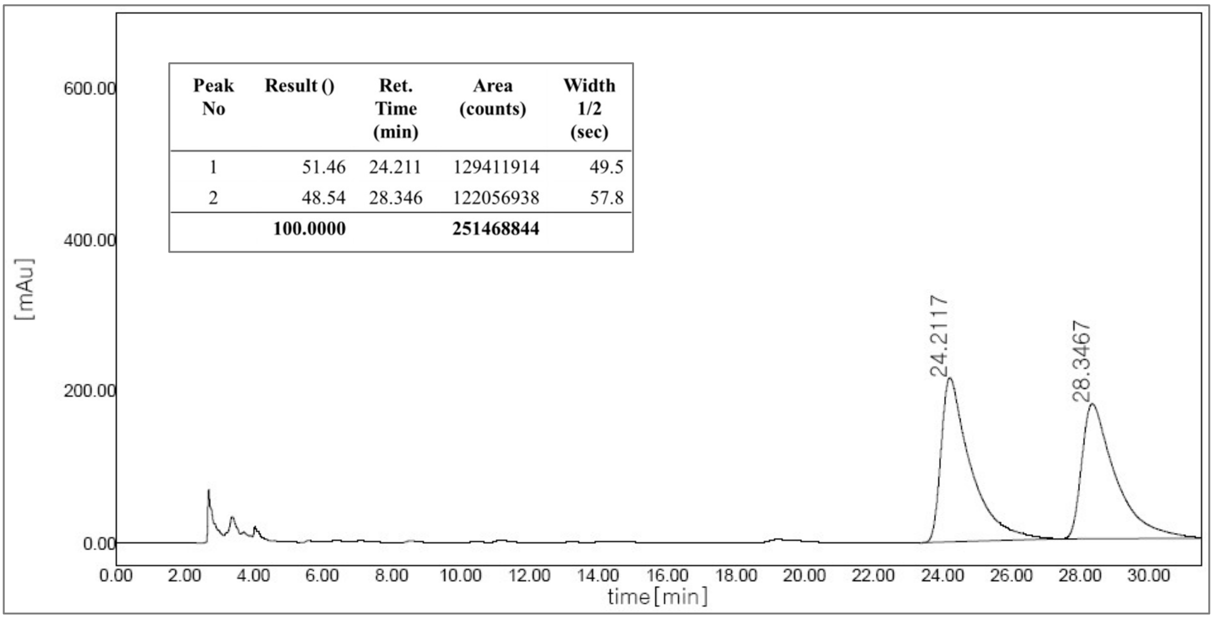

Peak No	Result ()	Ret. Time (min)	Area (counts)	Width 1/2 (sec)
1	51.46	24.211	129411914	49.5
2	48.54	28.346	122056938	57.8
	**100.0000**		**251468844**	

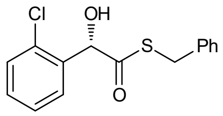

(*S*)-**5p**

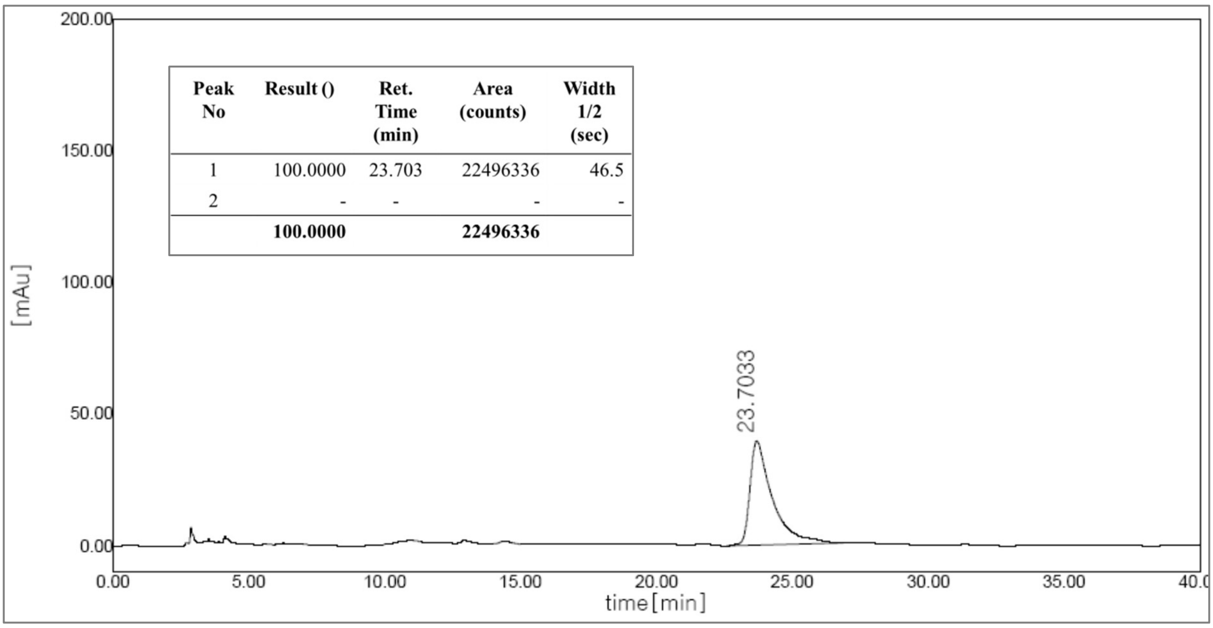

Peak No	Result ()	Ret. Time (min)	Area (counts)	Width 1/2 (sec)
1	100.0000	23.703	22496336	46.5
2	-	-	-	-
	**100.0000**		**22496336**	

Supplementary Figure 77. HPLC spectra of compound **5q**.

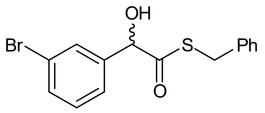

rac-**5q**

Chiralcel OZ-H (n-hexane/i-PrOH = 98/2, flow rate: 1.0 mL/min, wave length = 220 nm)

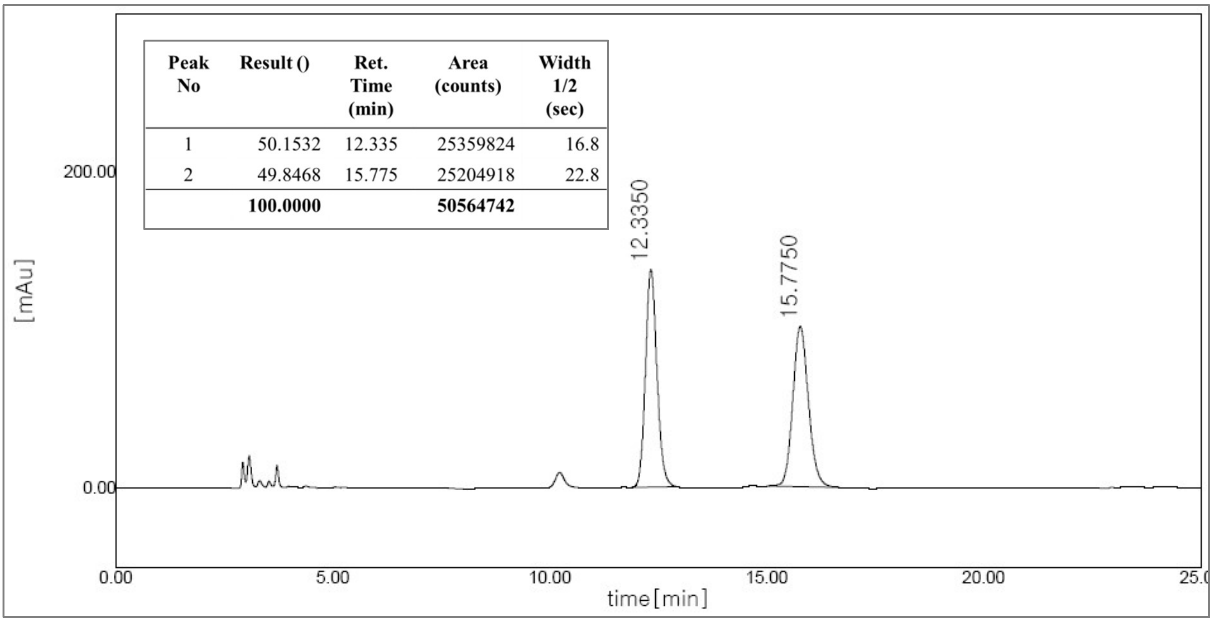

Peak No	Result ()	Ret. Time (min)	Area (counts)	Width 1/2 (sec)
1	50.1532	12.335	25359824	16.8
2	49.8468	15.775	25204918	22.8
	**100.0000**		**50564742**	

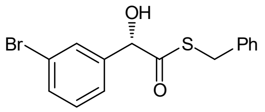

(*S*)-**5q**

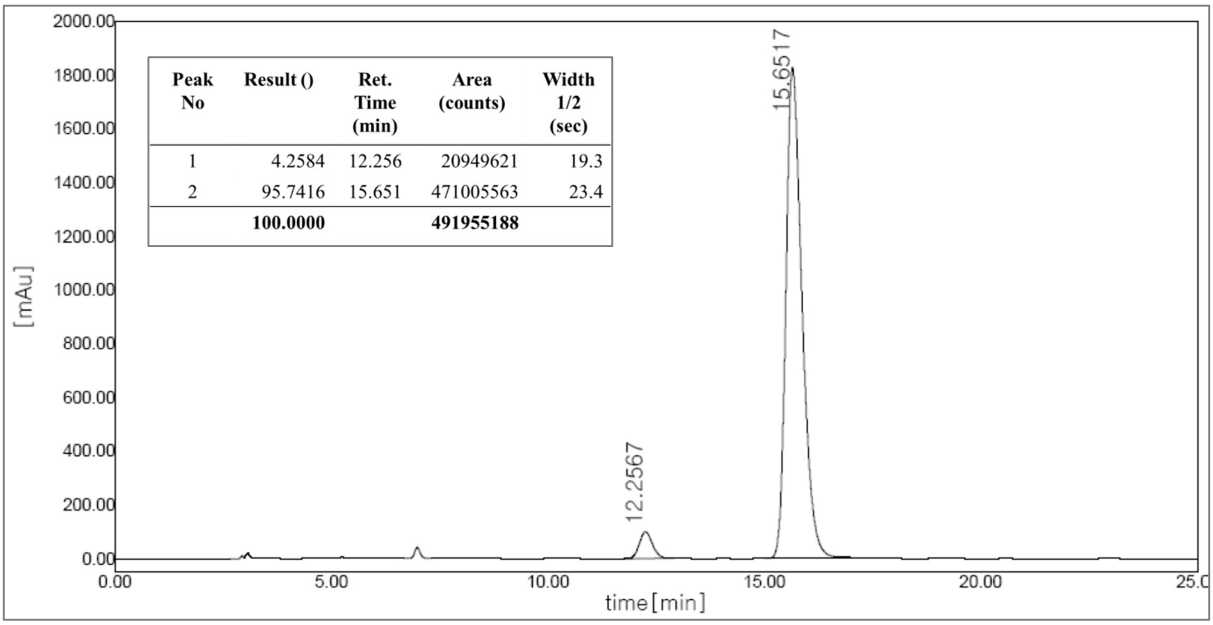

Peak No	Result ()	Ret. Time (min)	Area (counts)	Width 1/2 (sec)
1	4.2584	12.256	20949621	19.3
2	95.7416	15.651	471005563	23.4
	**100.0000**		**491955188**	

Supplementary Figure 78. HPLC spectra of compound **5r**.

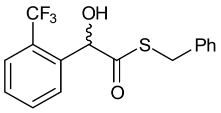

rac-**5r**

Chiralpak AD-H (n-hexane/i-PrOH = 96/4, flow rate: 1.0 mL/min, wave length = 220 nm)

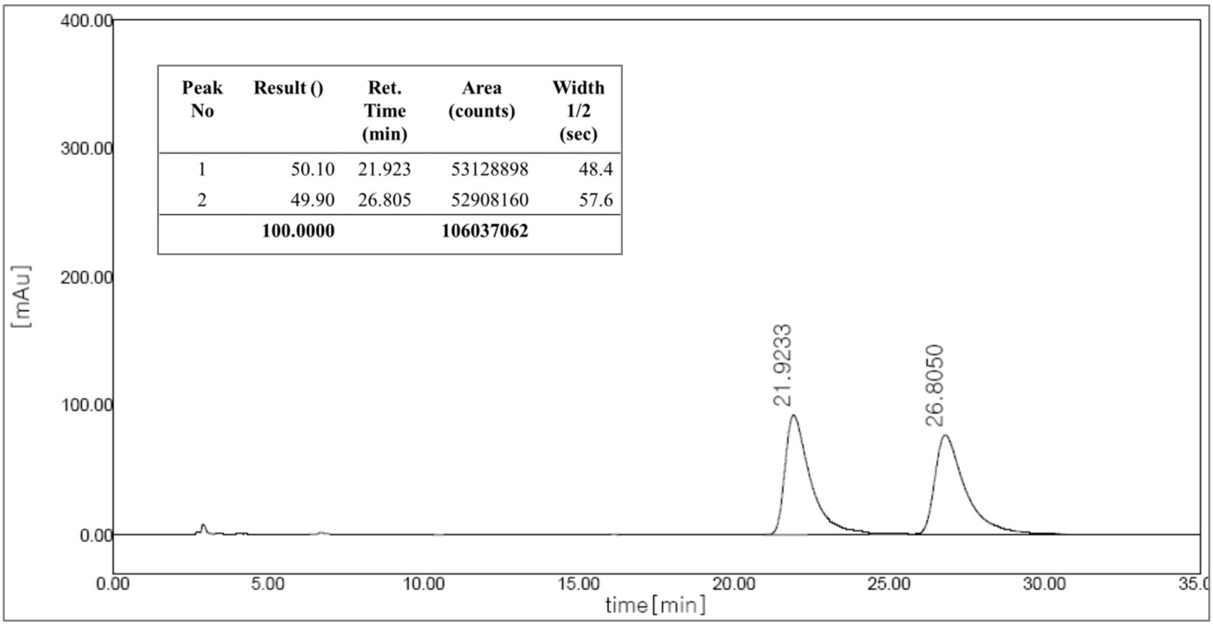

Peak No	Result ()	Ret. Time (min)	Area (counts)	Width 1/2 (sec)
1	50.10	21.923	53128898	48.4
2	49.90	26.805	52908160	57.6
	**100.0000**		**106037062**	

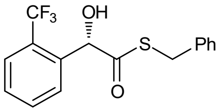

(*S*)-**5r**

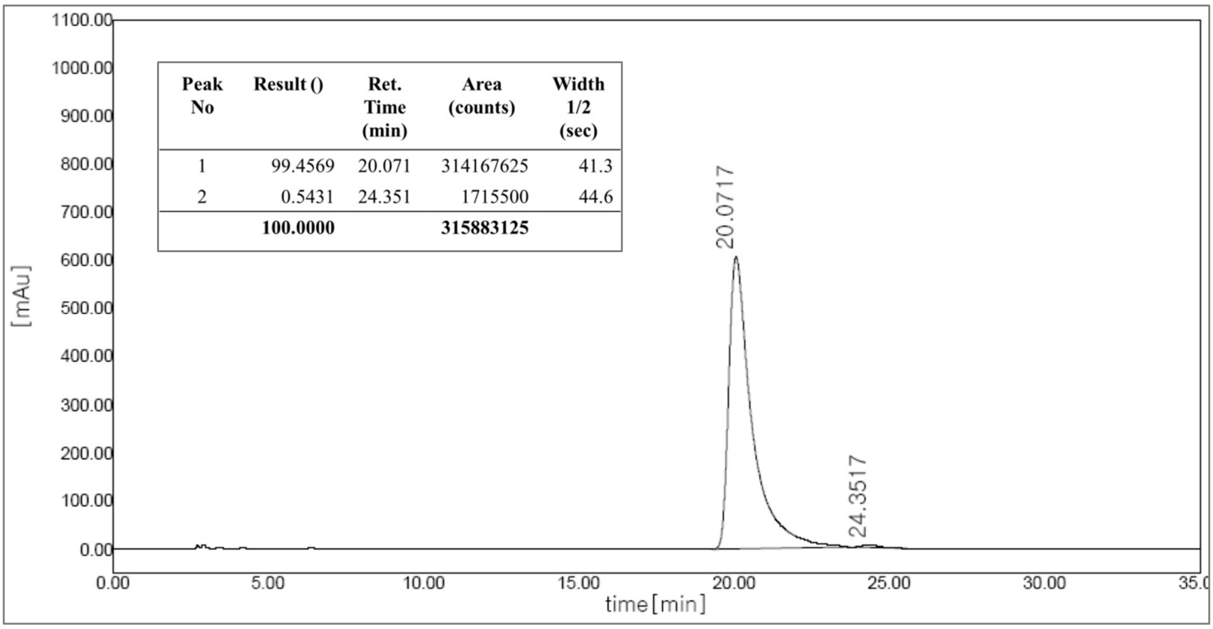

Peak No	Result ()	Ret. Time (min)	Area (counts)	Width 1/2 (sec)
1	99.4569	20.071	314167625	41.3
2	0.5431	24.351	1715500	44.6
	**100.0000**		**315883125**	

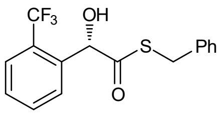

(*S*)-**5r** using 1 mol% of cat **1d**

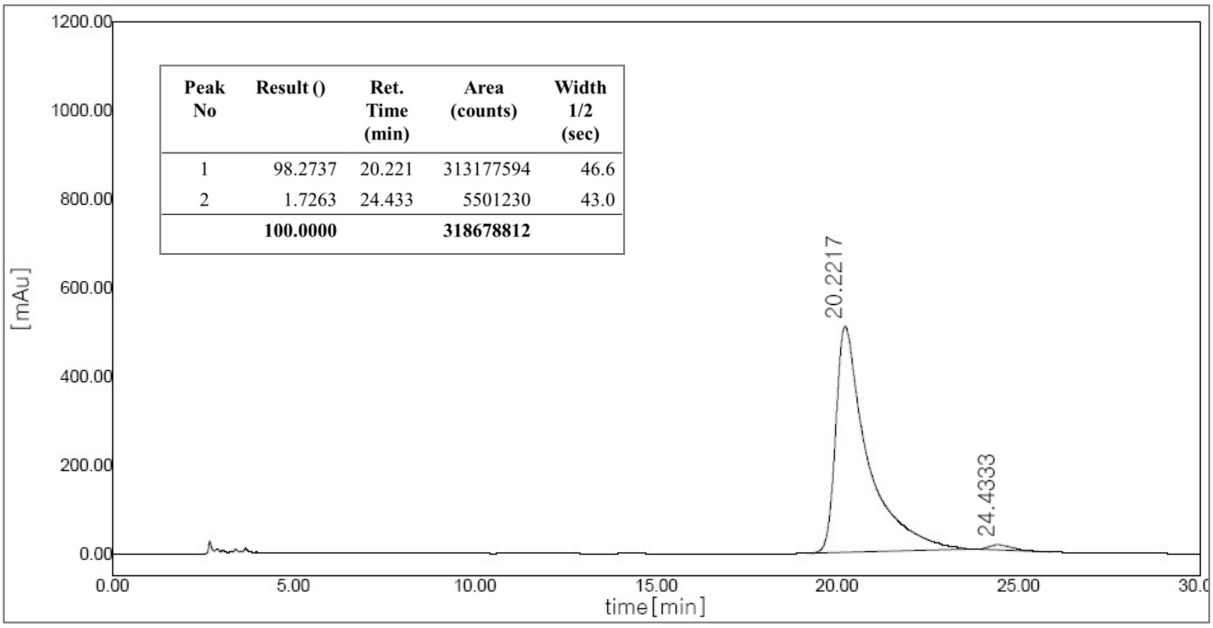

Peak No	Result ()	Ret. Time (min)	Area (counts)	Width 1/2 (sec)
1	98.2737	20.221	313177594	46.6
2	1.7263	24.433	5501230	43.0
	**100.0000**		**318678812**	

Supplementary Figure 79. HPLC spectra of compound **5s**.

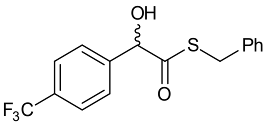

rac-**5s**

Chiralpak AD-H (n-hexane/i-PrOH = 96/4, flow rate: 1.0 mL/min, wave length = 220 nm)

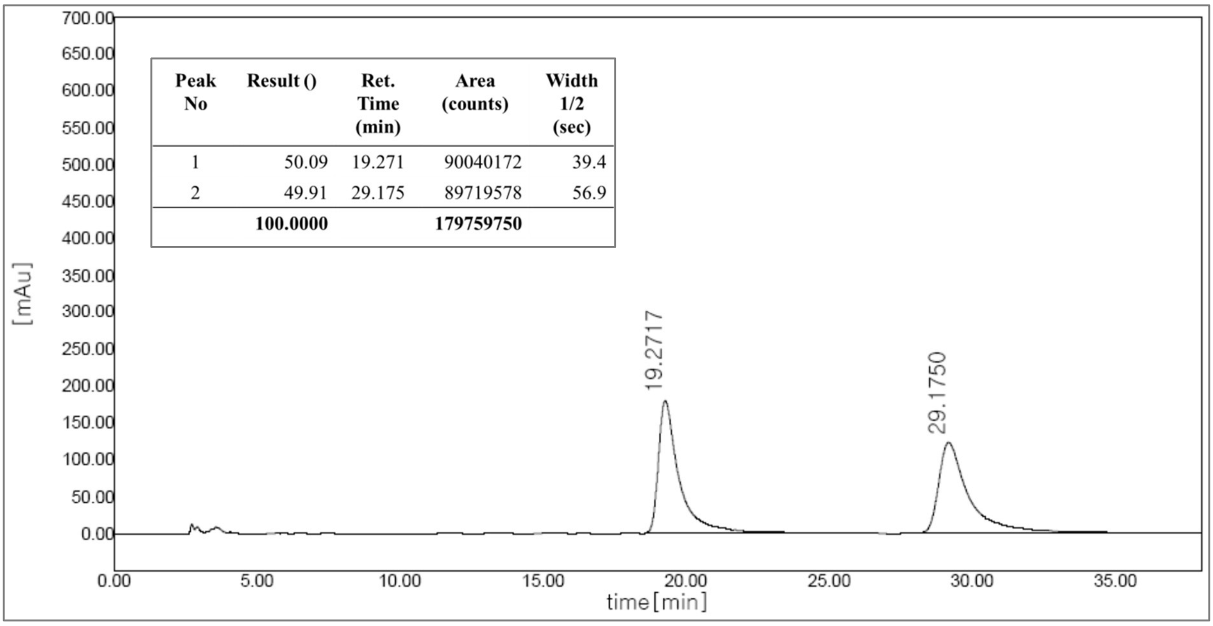

Peak No	Result ()	Ret. Time (min)	Area (counts)	Width 1/2 (sec)
1	50.09	19.271	90040172	39.4
2	49.91	29.175	89719578	56.9
	**100.0000**		**179759750**	

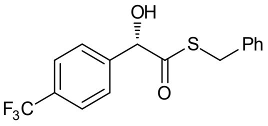

(*S*)-**5s**

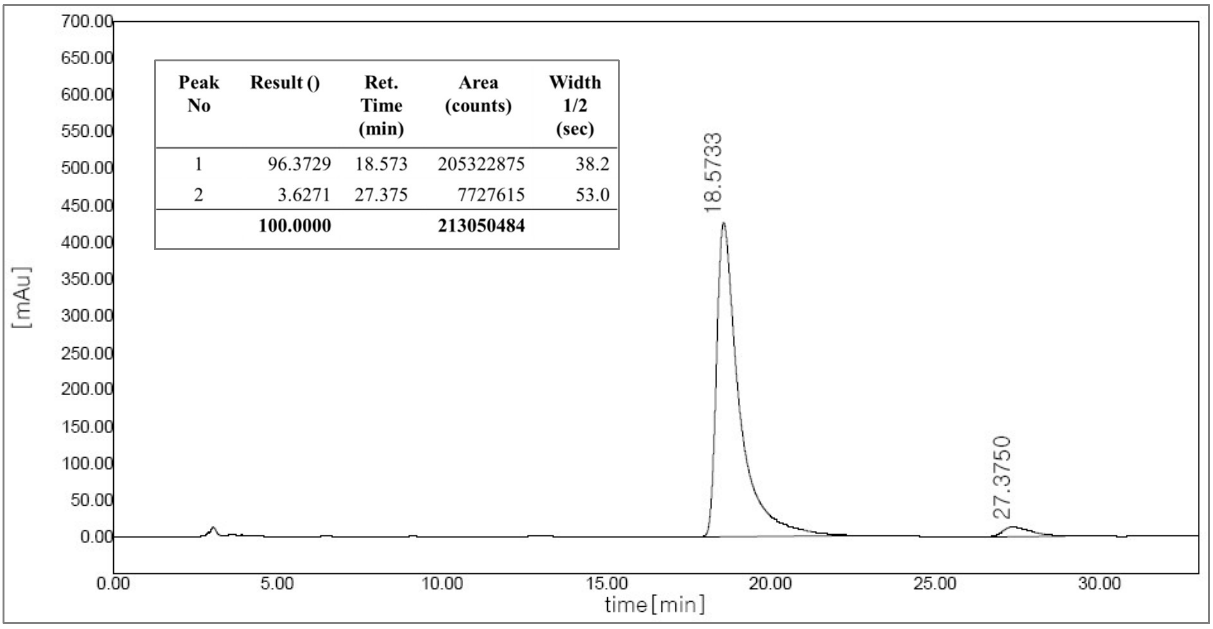

Peak No	Result ()	Ret. Time (min)	Area (counts)	Width 1/2 (sec)
1	96.3729	18.573	205322875	38.2
2	3.6271	27.375	7727615	53.0
	**100.0000**		**213050484**	

Supplementary Figure 80. HPLC spectra of compound **5t**.

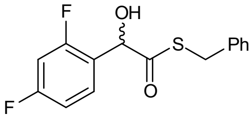

rac-**5t**

Chiralpak AD-H (n-hexane/i-PrOH = 96/4, flow rate: 1.0 mL/min, wave length = 220 nm)

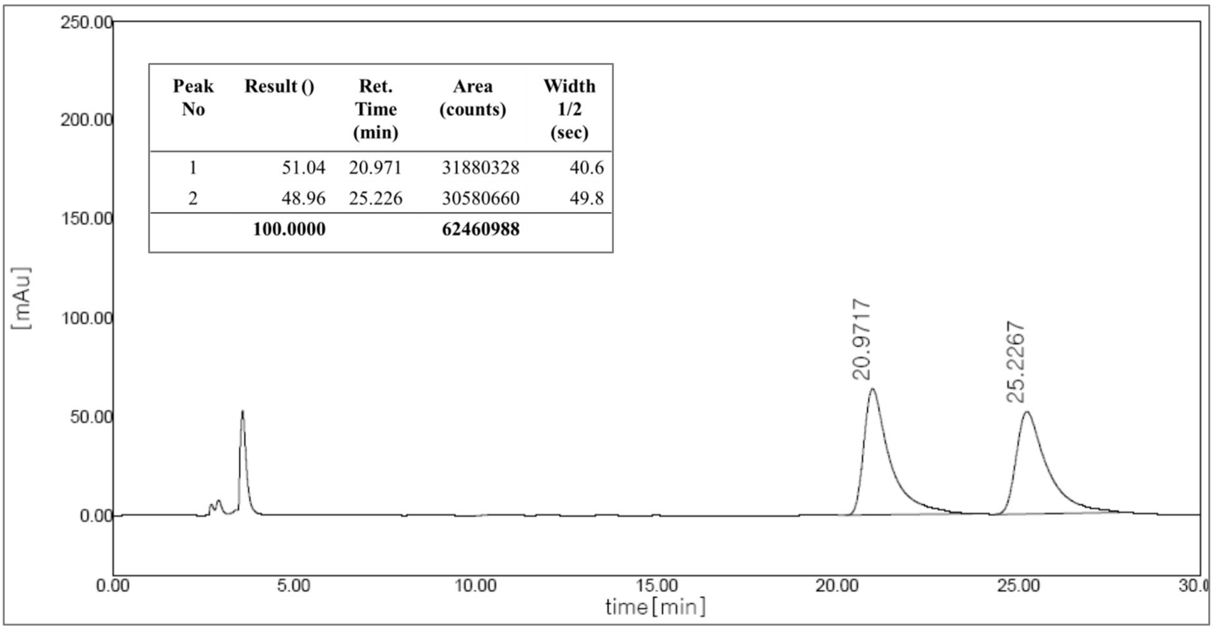

Peak No	Result ()	Ret. Time (min)	Area (counts)	Width 1/2 (sec)
1	51.04	20.971	31880328	40.6
2	48.96	25.226	30580660	49.8
	**100.0000**		**62460988**	

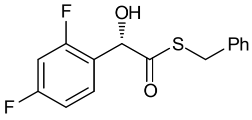

(*S*)-**5t**

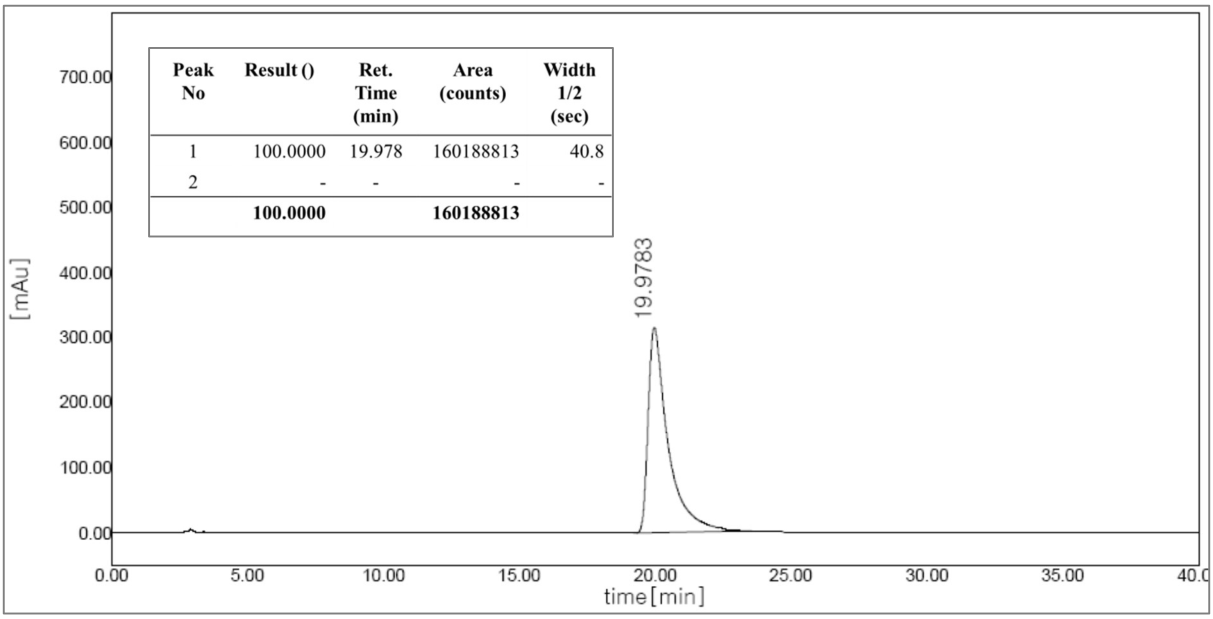

Peak No	Result ()	Ret. Time (min)	Area (counts)	Width 1/2 (sec)
1	100.0000	19.978	160188813	40.8
2	-	-	-	-
	**100.0000**		**160188813**	

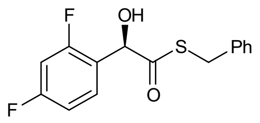

(*R*)-**5t**

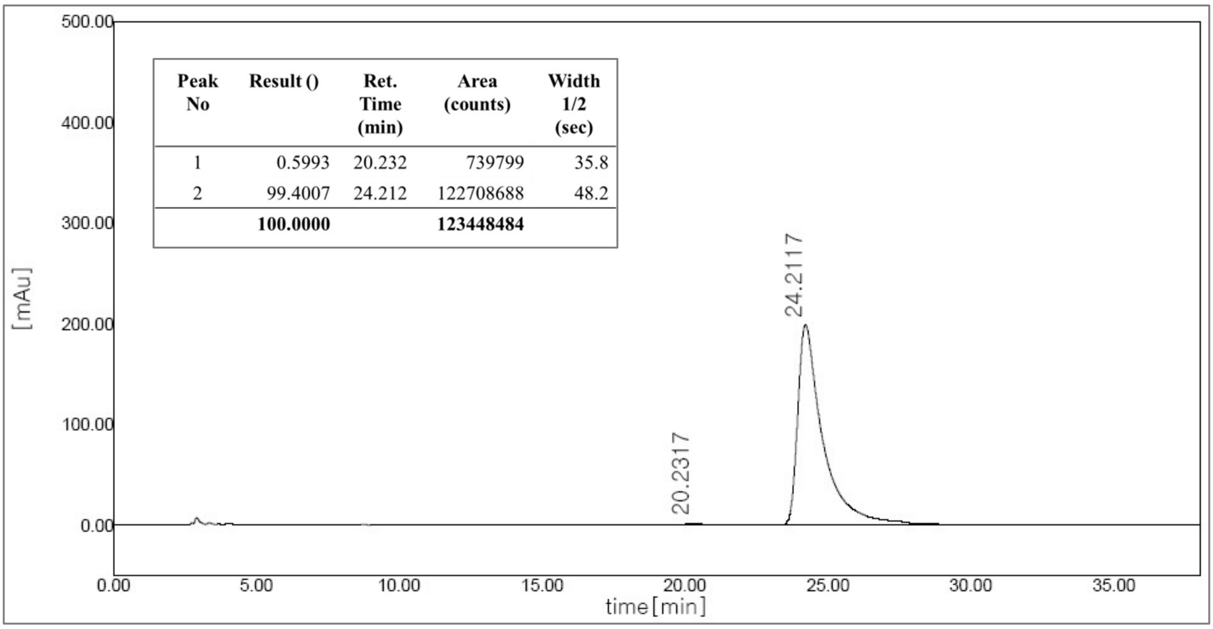

Peak No	Result ()	Ret. Time (min)	Area (counts)	Width 1/2 (sec)
1	0.5993	20.232	739799	35.8
2	99.4007	24.212	122708688	48.2
	**100.0000**		**123448484**	

Supplementary Figure 81. HPLC spectra of compound **5u**.

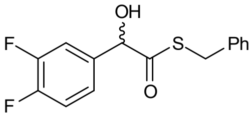

rac-**5u**

Chiralpak AD-H (n-hexane/i-PrOH = 96/4, flow rate: 1.0 mL/min, wave length = 220 nm)

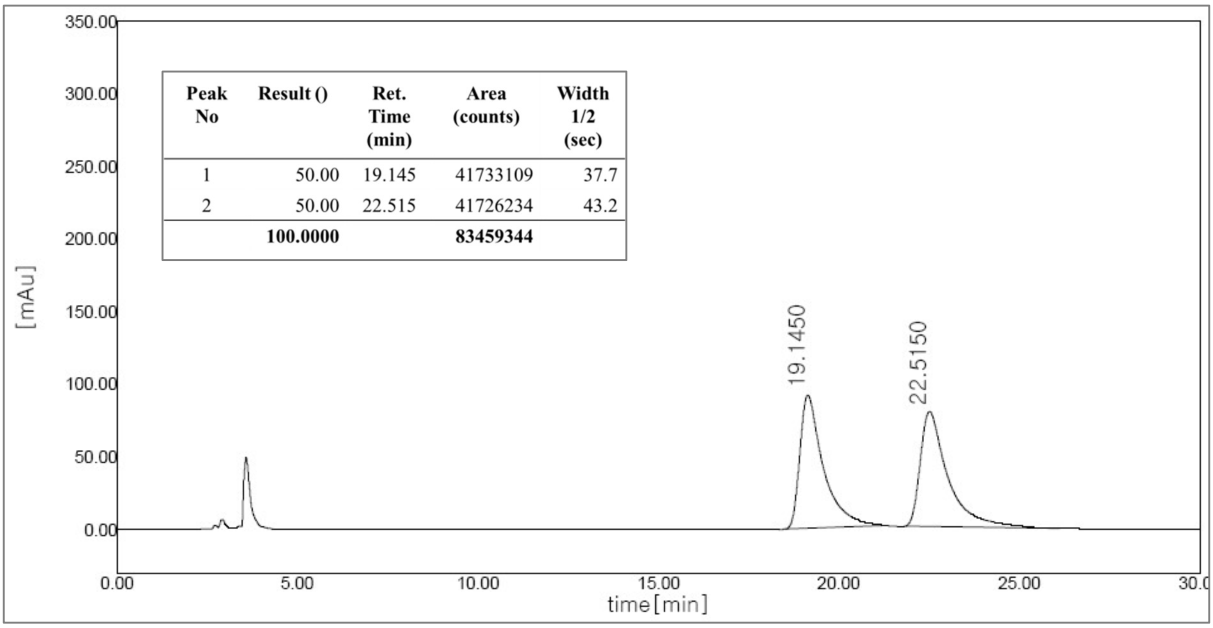

Peak No	Result ()	Ret. Time (min)	Area (counts)	Width 1/2 (sec)
1	50.00	19.145	41733109	37.7
2	50.00	22.515	41726234	43.2
	**100.0000**		**83459344**	

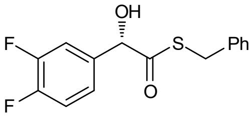

(*S*)-**5u**

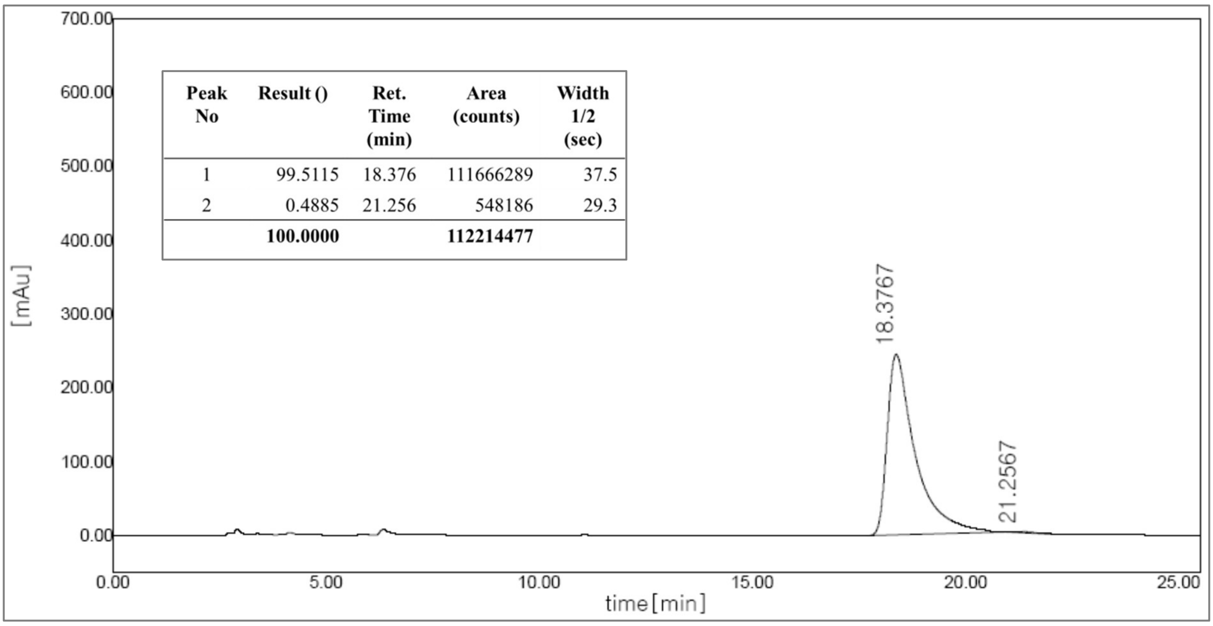

Peak No	Result ()	Ret. Time (min)	Area (counts)	Width 1/2 (sec)
1	99.5115	18.376	111666289	37.5
2	0.4885	21.256	548186	29.3
	**100.0000**		**112214477**	

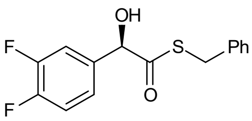

(*R*)-**5u**

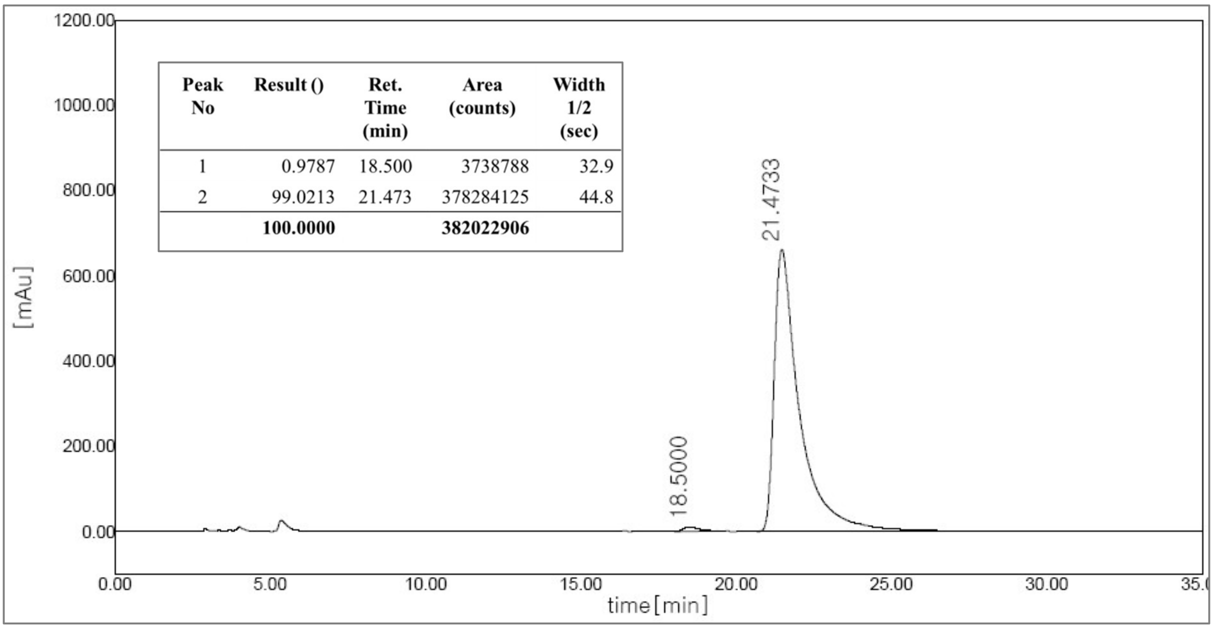

Peak No	Result ()	Ret. Time (min)	Area (counts)	Width 1/2 (sec)
1	0.9787	18.500	3738788	32.9
2	99.0213	21.473	378284125	44.8
	**100.0000**		**382022906**	

Supplementary Figure 82. HPLC spectra of compound **5v**.

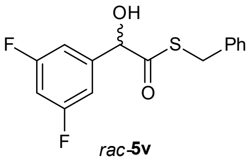

rac-**5v**

Chiralcel OD-H (n-hexane/i-PrOH = 98/2, flow rate: 1.0 mL/min, wave length = 220 nm)

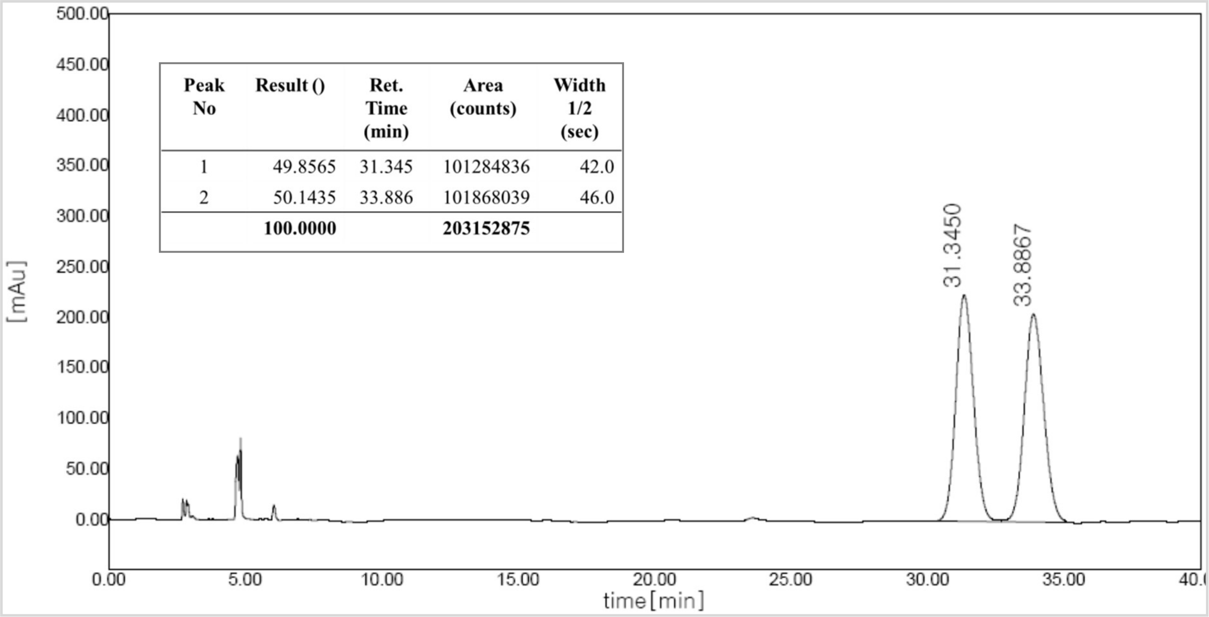

Peak No	Result ()	Ret. Time (min)	Area (counts)	Width 1/2 (sec)
1	49.8565	31.345	101284836	42.0
2	50.1435	33.886	101868039	46.0
	**100.0000**		**203152875**	

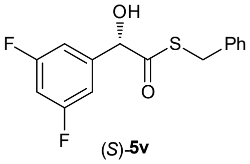

(*S*)-**5v**

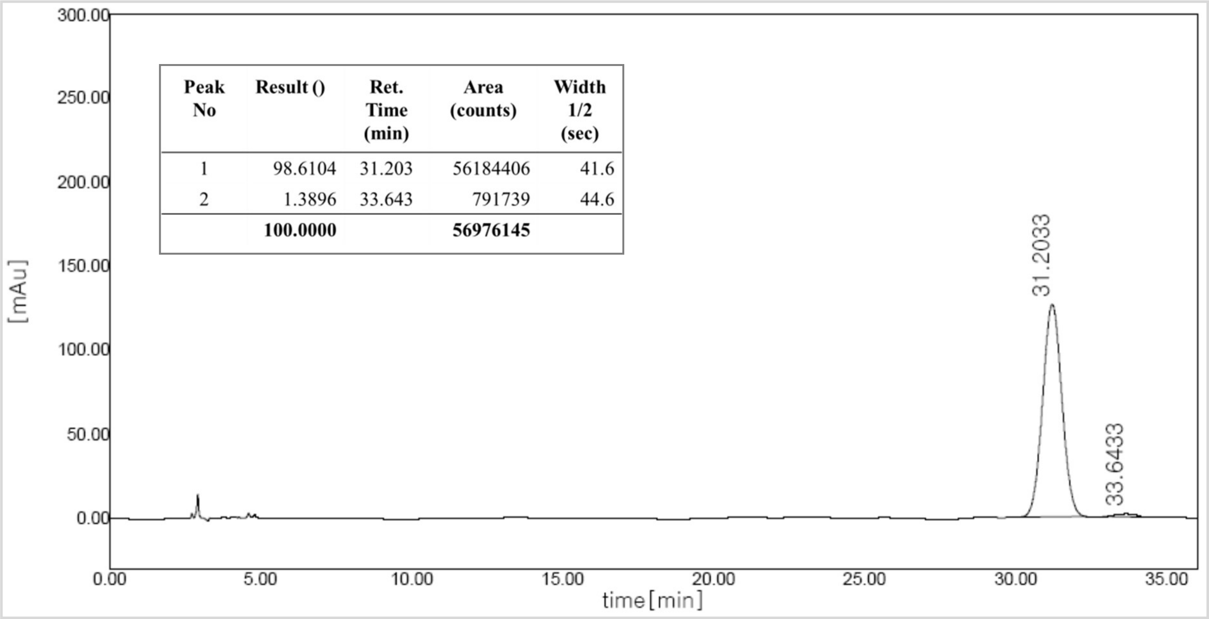

Peak No	Result ()	Ret. Time (min)	Area (counts)	Width 1/2 (sec)
1	98.6104	31.203	56184406	41.6
2	1.3896	33.643	791739	44.6
	**100.0000**		**56976145**	

Supplementary Figure 83. HPLC spectra of compound **5w**.

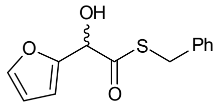

rac-**5w**

Chiralpak AD-H (n-hexane/i-PrOH = 96/4, flow rate: 1.0 mL/min, wave length = 220 nm)

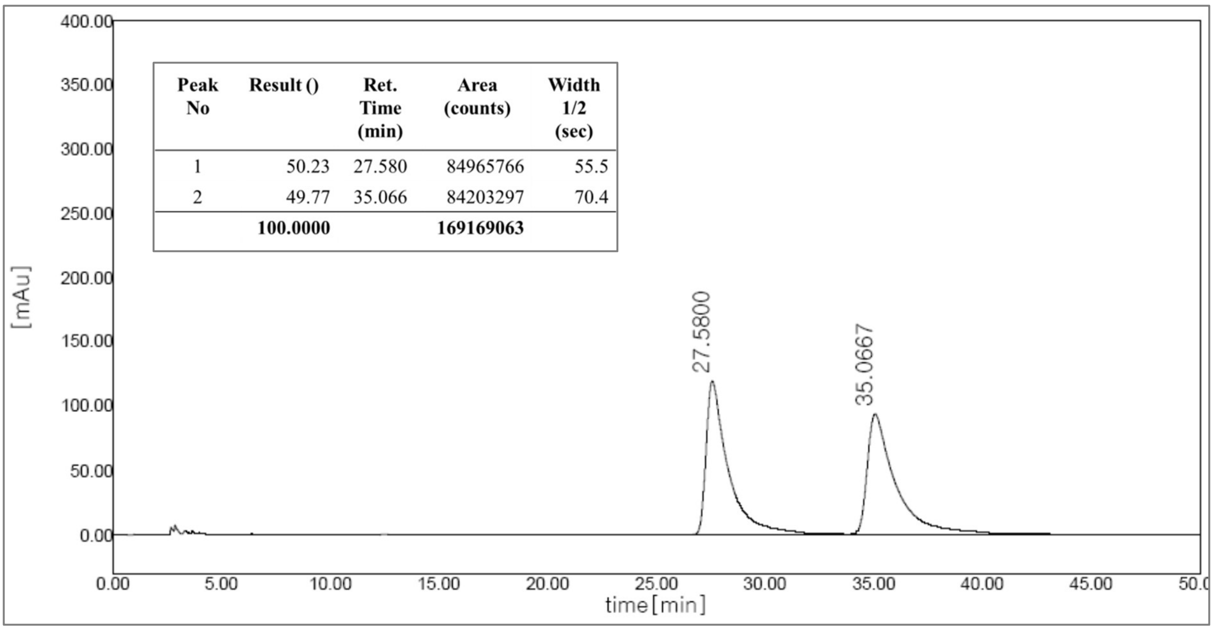

Peak No	Result ()	Ret. Time (min)	Area (counts)	Width 1/2 (sec)
1	50.23	27.580	84965766	55.5
2	49.77	35.066	84203297	70.4
	**100.0000**		**169169063**	

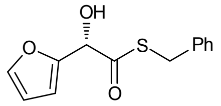

(*S*)-**5w**

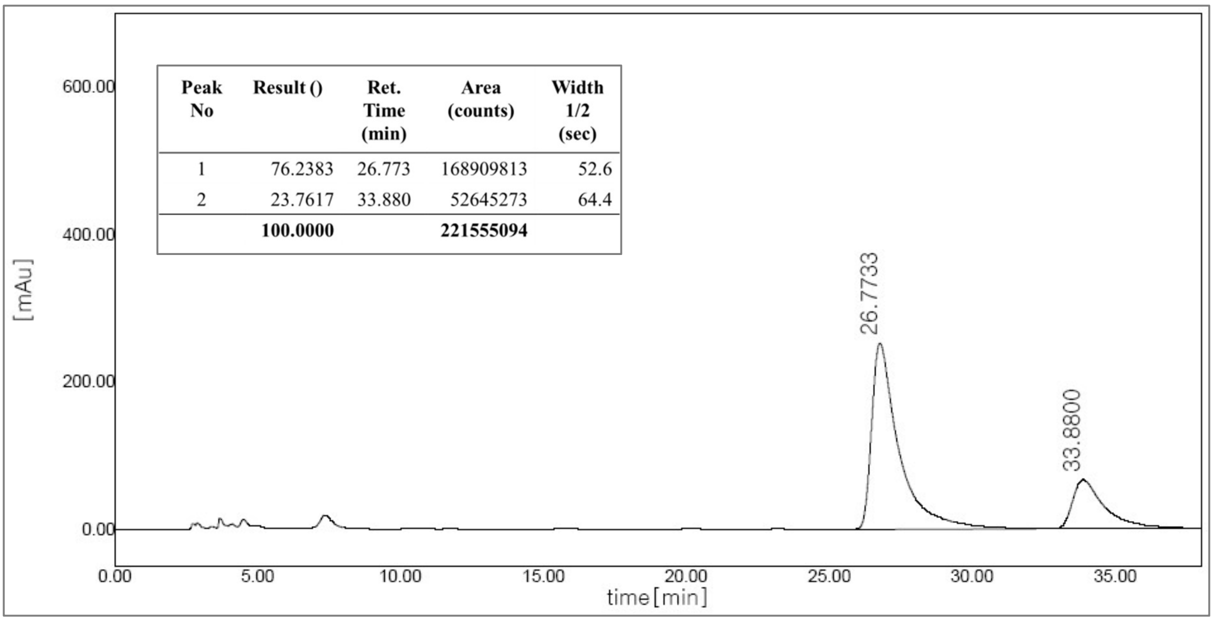

Peak No	Result ()	Ret. Time (min)	Area (counts)	Width 1/2 (sec)
1	76.2383	26.773	168909813	52.6
2	23.7617	33.880	52645273	64.4
	**100.0000**		**221555094**	

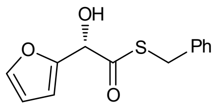

(*S*)-**5w** using 30 mol% of cat **1d** at 0 °C

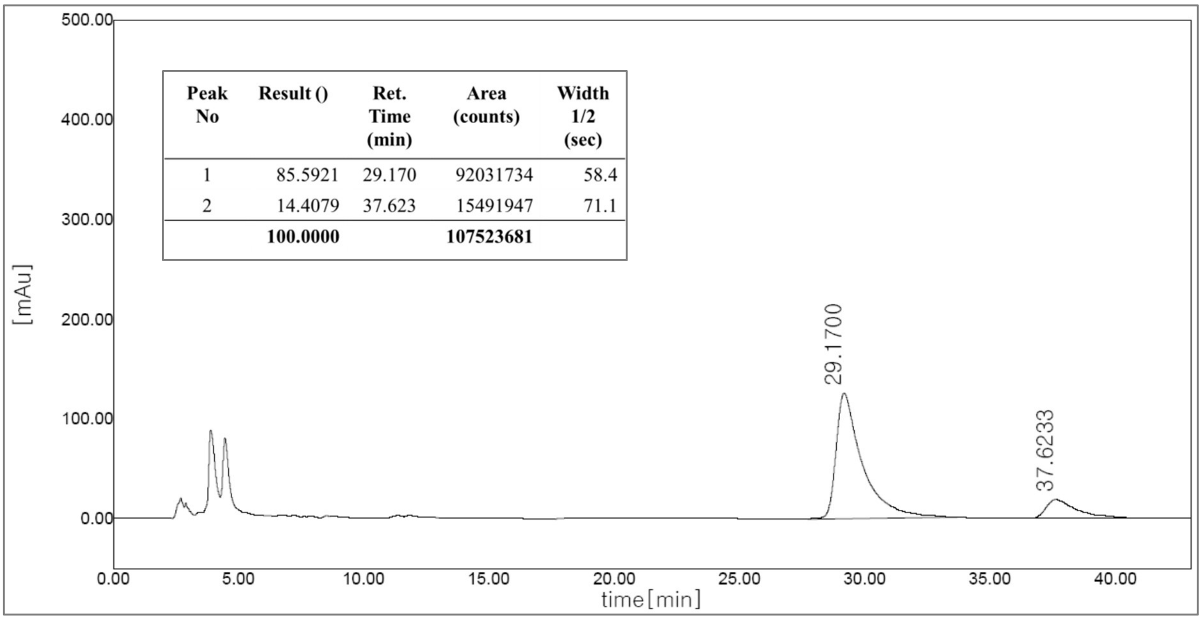

Peak No	Result ()	Ret. Time (min)	Area (counts)	Width 1/2 (sec)
1	85.5921	29.170	92031734	58.4
2	14.4079	37.623	15491947	71.1
	**100.0000**		**107523681**	

Supplementary Figure 84. HPLC spectra of compound **5x**.

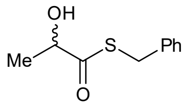

rac-**5x**

Regis (*R*,*R*) Whelk-O1

(n-hexane/i-PrOH = 99/1, flow rate: 0.7 mL/min, wave length = 220 nm)

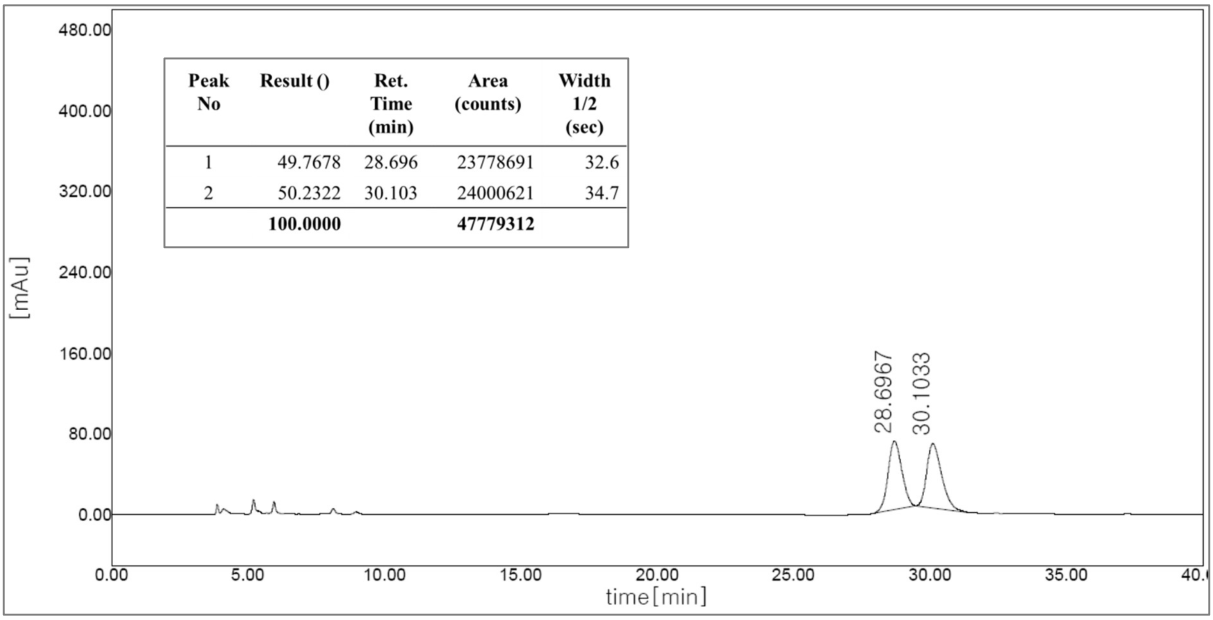

Peak No	Result ()	Ret. Time (min)	Area (counts)	Width 1/2 (sec)
1	49.7678	28.696	23778691	32.6
2	50.2322	30.103	24000621	34.7
	**100.0000**		**47779312**	

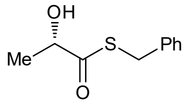

(*S*)-**5x**

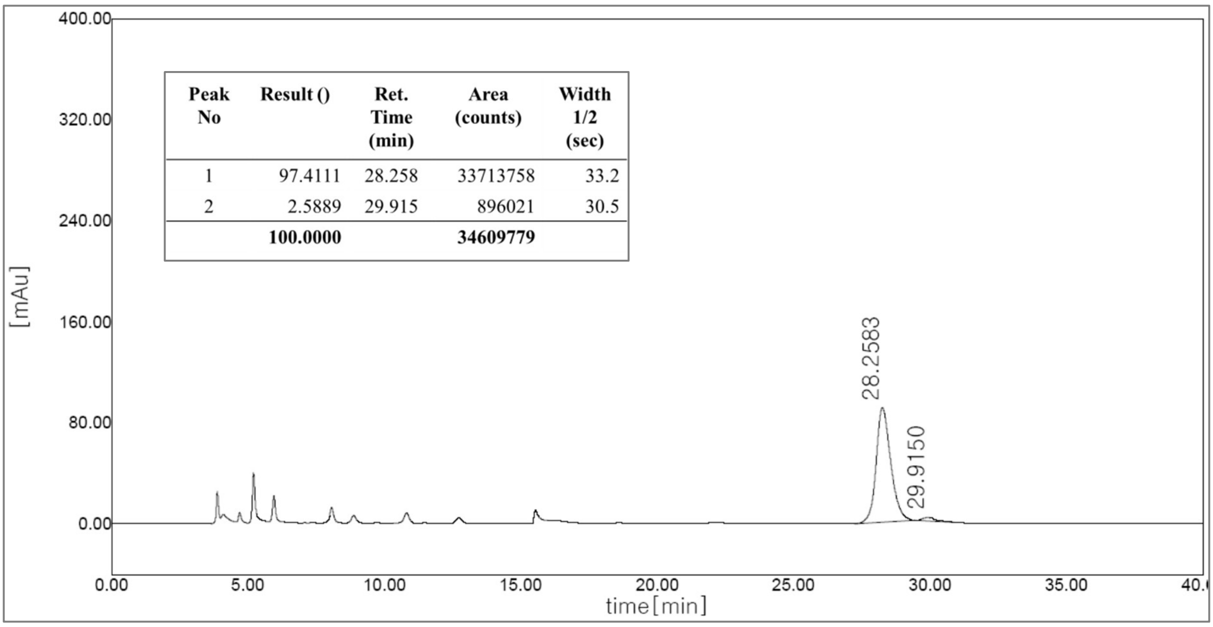

Peak No	Result ()	Ret. Time (min)	Area (counts)	Width 1/2 (sec)
1	97.4111	28.258	33713758	33.2
2	2.5889	29.915	896021	30.5
	**100.0000**		**34609779**	

Supplementary Figure 85. HPLC spectra of compound **5y**.

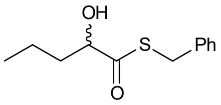

rac-**5y**

Chiralcel OD-H (n-hexane/i-PrOH = 98/2, flow rate: 1.0 mL/min, wave length = 220 nm)

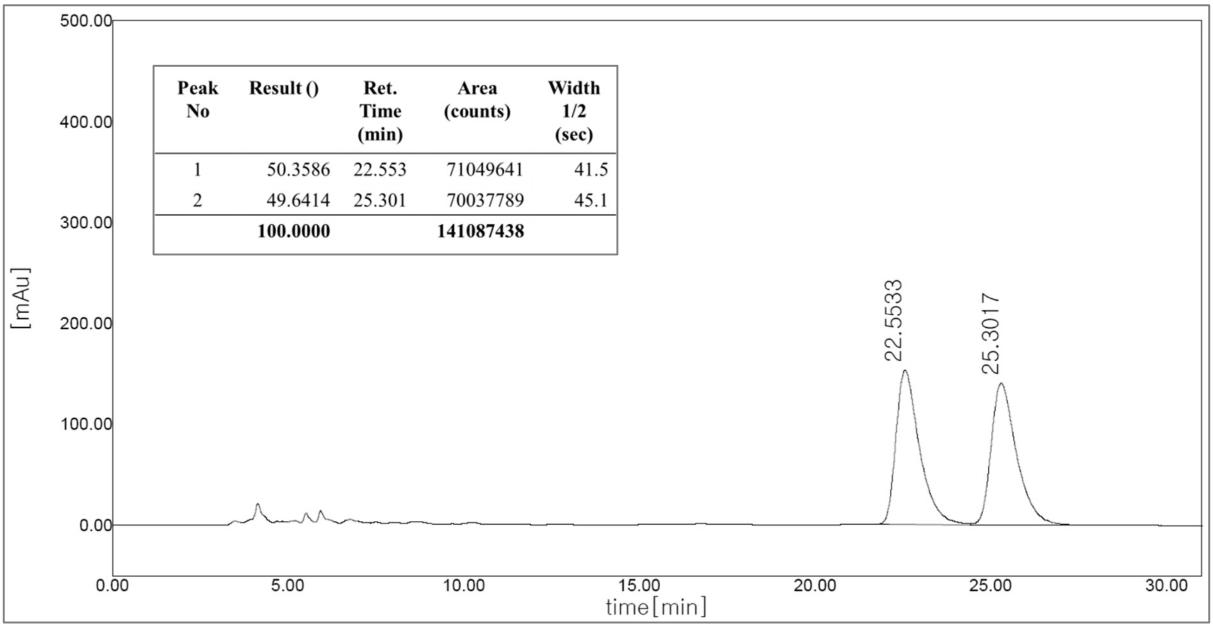

Peak No	Result ()	Ret. Time (min)	Area (counts)	Width 1/2 (sec)
1	50.3586	22.553	71049641	41.5
2	49.6414	25.301	70037789	45.1
	**100.0000**		**141087438**	

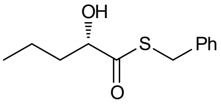

(*S*)-**5y**

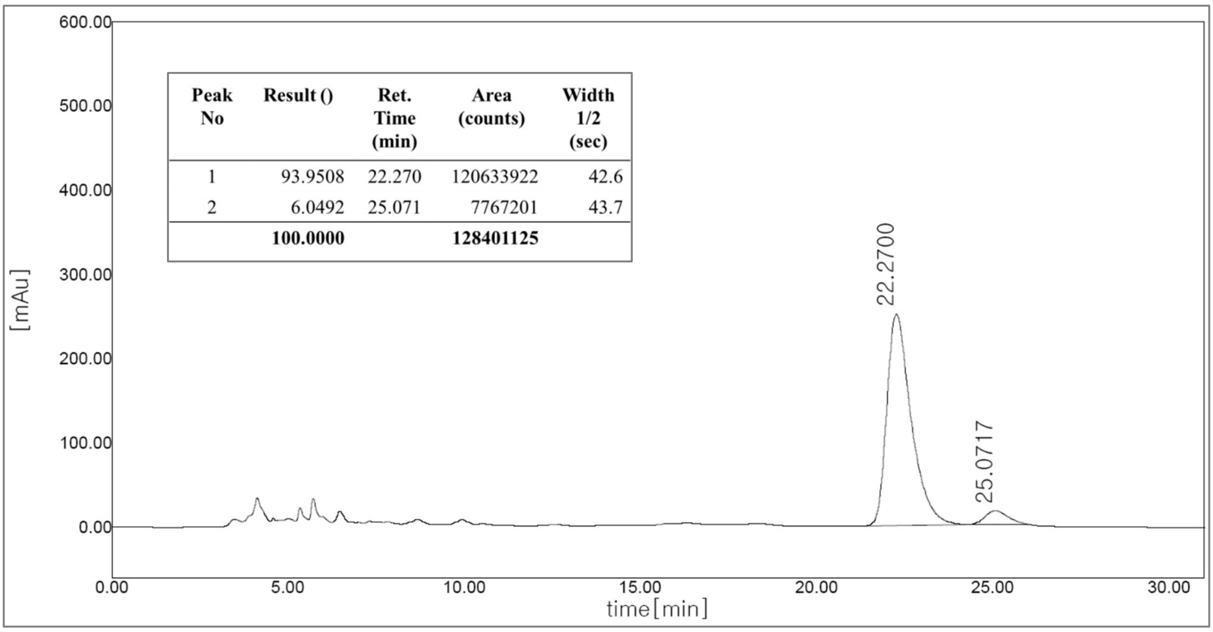

Peak No	Result ()	Ret. Time (min)	Area (counts)	Width 1/2 (sec)
1	93.9508	22.270	120633922	42.6
2	6.0492	25.071	7767201	43.7
	**100.0000**		**128401125**	

Supplementary Figure 86. HPLC spectra of compound **5z**.

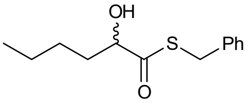

rac-**5z**

Chiralcel OD-H (n-hexane/i-PrOH = 98/2, flow rate: 1.0 mL/min, wave length = 220 nm)

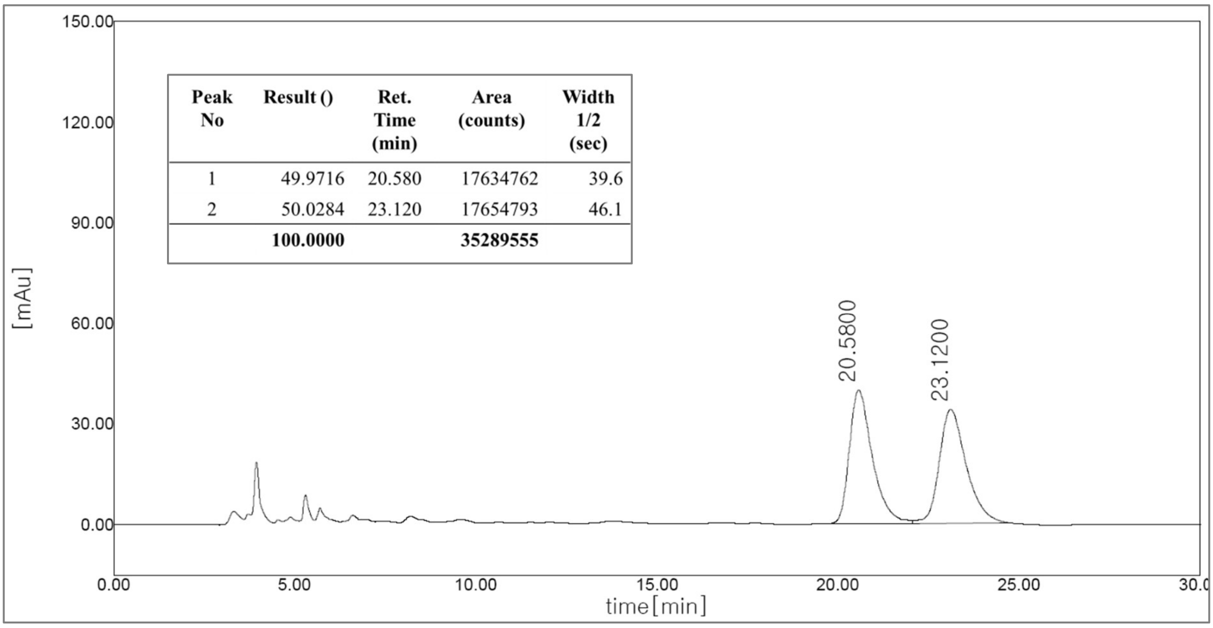

Peak No	Result ()	Ret. Time (min)	Area (counts)	Width 1/2 (sec)
1	49.9716	20.580	17634762	39.6
2	50.0284	23.120	17654793	46.1
	**100.0000**		**35289555**	

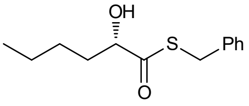

(*S*)-**5z**

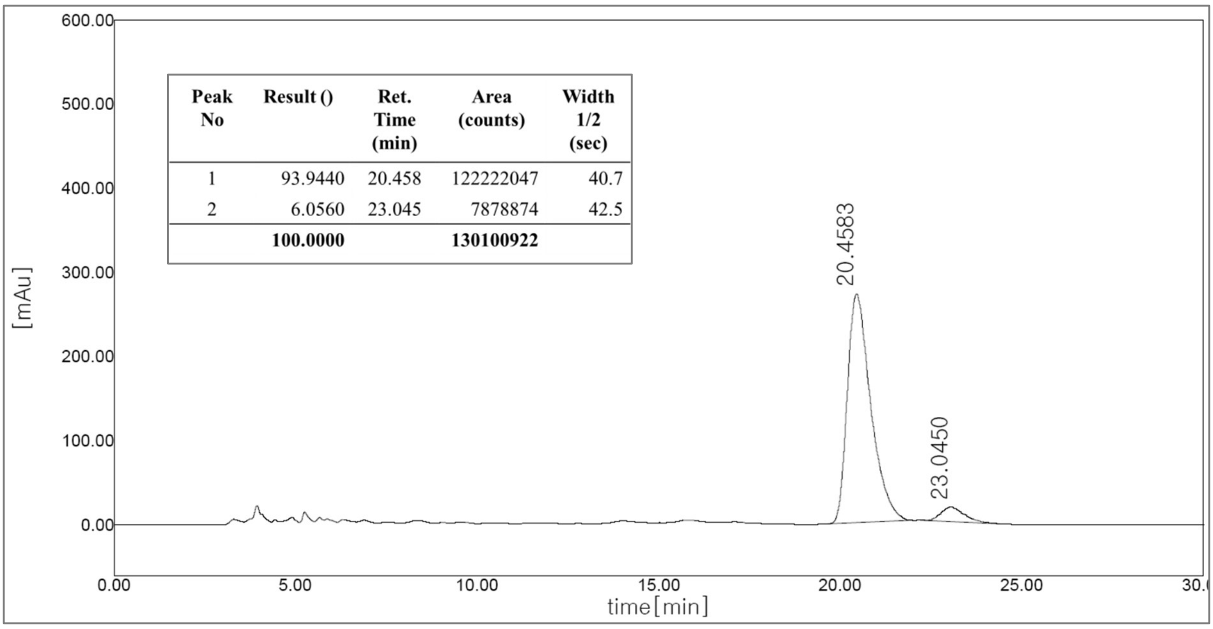

Peak No	Result ()	Ret. Time (min)	Area (counts)	Width 1/2 (sec)
1	93.9440	20.458	122222047	40.7
2	6.0560	23.045	7878874	42.5
	**100.0000**		**130100922**	

Supplementary Table

Supplementary Table 1. Study of the effect of catalyst optical purity on product optical purity

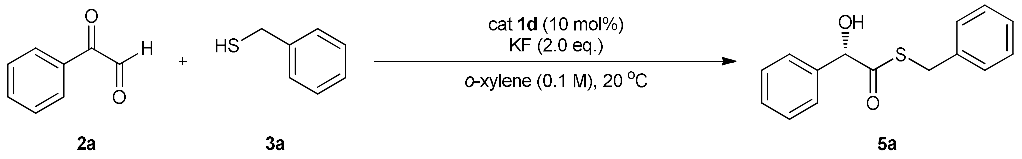

ee of cat **1d** [%]	0	21.5	39.7	61.2	80.2	100
ee of **5a** [%]	0	21.7	39.4	58.9	79.7	98

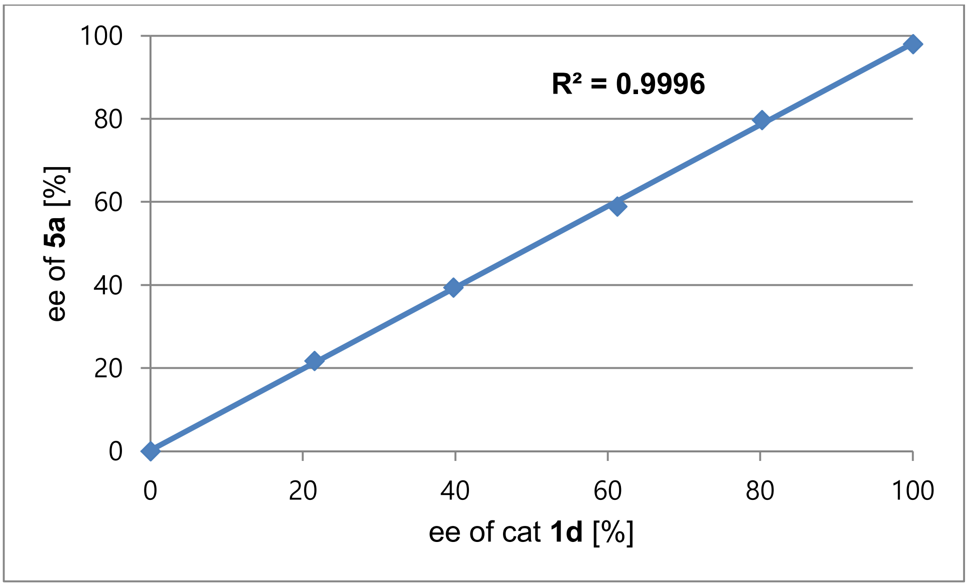

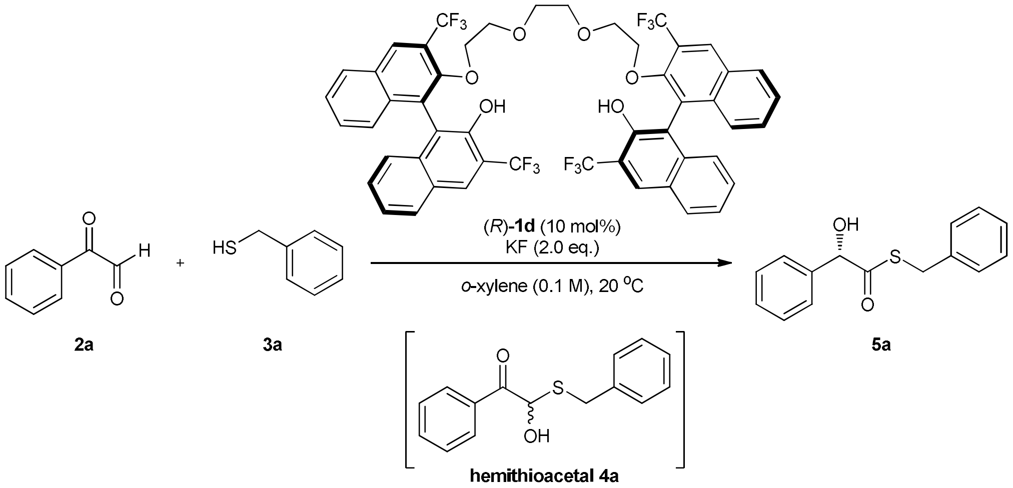

conversion [%]	10	26	52	80
ee of hemithioacetal **4a** [%]	rac	rac	rac	rac

Supplementary Table 2. Optical purity of the hemithioacetal at different reaction times. The reactions were performed with **2a** (0.1 mmol, monohydrate form), **3a** (1.0 equiv.), catalyst (10 mol%) and KF (2.0 equiv.) at 20 °C. The conversions were monitored by ^{1}H NMR analysis of the reaction mixture. HPLC analysis (Chiralcel AD-H; n-hexane/i-PrOH = 96/4, flow rate: 1.0 mL/min, wave length = 220 nm): t_R = 16.3 min, t_R = 17.9 min.

Comments: These results indicate that the isomerization reaction does not proceed through kinetic resolution of the hemithioacetals.

Supplementary Methods

General information

All reactions were carried out under argon atmosphere in oven-dried glassware with magnetic stirring. Unless otherwise stated, all reagents were purchased from commercial suppliers and used without further purification. All solvents employed in the reactions were distilled from appropriate drying agents prior to use. The organocatalysts **1a–1i** were obtained starting from BINOL according to the literature procedure[2,3]. The glyoxals **2a**, **2d**, **2e**, **2j–2o** and **2r–2u** were obtained as a hydrate commercially and used without further purification. The glyoxals **2b**, **2c**, **2f–2i**, **2p**, **2q**, **2v**, **2w**, **2y** and **2z** were prepared from the corresponding ketone or aldehyde according to the literature procedure[4,5]. The methylglyoxal **2x** was purchased in a form of 40% aqueous solution from commercial sources and used after purification according to the literature procedure[6]. Thin-layer chromatography (TLC) was performed using silica gel plates (Merck, Kieselgel 60 F254 0.25 mm). Chromatographic purification of the products was performed by using silica gel 60 (230–400 mesh, Merck). ^{1}H NMR (500 MHz) and ^{13}C NMR (125 MHz) spectra were recorded using a Bruker 500 spectrometer. Chemical shifts are reported in ppm downfield from tetramethylsilane (TMS) as the internal standard or with the solvent reference as the internal standard ($CDCl_3$: δ 7.26 for ^{1}H NMR and δ 77.0 for ^{13}C NMR). ^{19}F NMR (470.4 MHz) spectra were recorded using Bruker 500 spectrometer with benzotrifluoride ($C_6H_5CF_3$) as external standard. Data are reported as follows: chemical shift, multiplicity (s = singlet, d = doublet, t = triplet, q = quartet, m = multiplet, br = broad), coupling constants (Hz) and integration. HPLC analyses for the determination of the enantiomeric excess (ee) of the products were performed using an Agilent 1260 Infinity instrument equipped with an isostatic pump using a chiral column (CHIRALPACK AD-H, Chiralcel OD-H, Chiralcel OZ-H, Regis (*R*,*R*) WHELK-O1; 250 mm × 4.6 mm). IR spectra were recorded using a Bruker Vertex 70 spectrometer with the MIRacle Micro ATR accessory. High-resolution mass spectrometry (HRMS) was recorded using a Bruker Compact mass spectrometer. Melting points (Mps) were determined using a Buchi B-540 melting point apparatus and are uncorrected. Optical rotations were measured using a PerkinElmer Polarimeter 343 plus. Abbreviations: e.e. (enantiomeric excess), n.r. (no reaction), THF (tetrahydrofuran), rac (racemic).

Representative Procedure for the Enantioselective Isomerization of Hemithioacetals

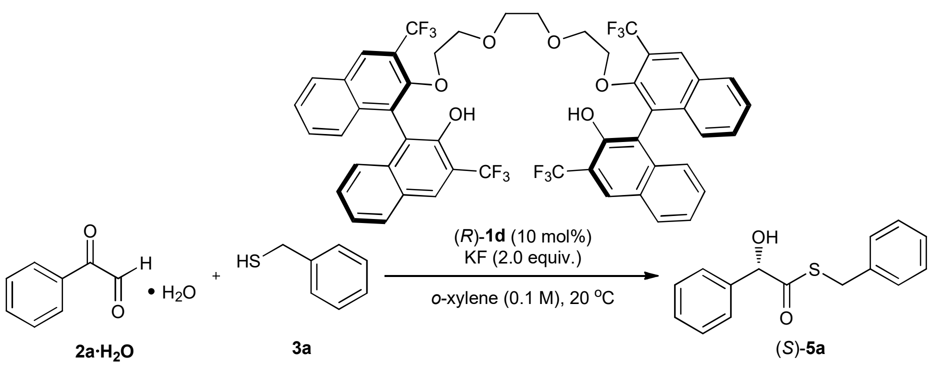

In a capped vial, anhydrous *o*-xylene (1.0 mL) was added to a mixture of phenylglyoxal monohydrate **2a·H$_2$O** (13.4 mg, 0.1 mmol), catalyst (*R*)-**1d** (9.6 mg, 10 mol%) and spray dried KF (11.6 mg, 0.2 mmol, 2.0 equiv.). The reaction mixture was stirred for 1 min at 20 ºC. Next, thiol **3a** (12 μl, 0.1 mmol, 1.0 equiv.) was added to the reaction mixture and was further stirred for 48 h at 20 ºC. After complete consumption of the hemithioacetal **4a** (following by TLC), the mixture was purified using silica gel column chromatography (acetone/n-hexane = 1/10) to obtain the desired product **5a** as colorless liquid in 99% yield and 98% ee.

Determination of the Absolute Configuration of 5a

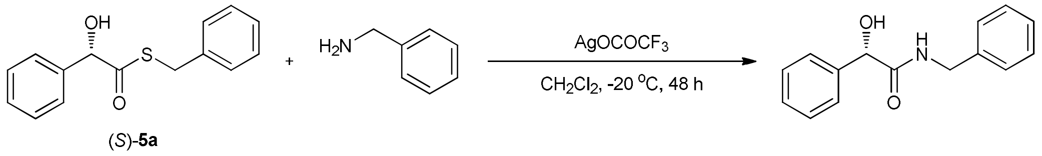

To a stirred solution of (*S*)-**5a** (26 mg, 0.1 mmol, 95% ee) and AgOCOCF$_3$ (22 mg, 0.1 mmol) in CH$_2$Cl$_2$ (0.2 mL), benzylamine (0.022 ml, 0.2 mmol) was added at −20 ºC, and the reaction mixture was allowed to stir for 48 h. Solvent was evaporated in vacuo, and the crude reaction mixture was purified by flash chromatography on silica gel (acetone/n-hexane = 1/6) to get the corresponding (*S*)-α–hydroxyamide product as colorless oil (89%, 22 mg, 92% ee). $[\alpha]_D^{20}$ = +41.3° (c = 0.02 in acetone, 92% ee); ^{1}H NMR (500 MHz, CDCl$_3$): δ 7.37–7.24 (m, 8H), 7.20–7.10 (m, 2H), 6.76 (s, 1H), 4.97 (s, 1H), 4.48–4.29 (m, 2H), 4.16 (bs, 1H); ^{13}C NMR (125 MHz, CDCl$_3$): δ 172.34, 139.53, 137.74, 128.81, 128.73, 128.59, 127.62, 127.59, 126.83, 74.15, 43.39. Enantiomeric excess was determined by using HPLC analysis (Chiralcel AD-H; n-hexane/i-PrOH = 85/15, flow rate: 1.0 mL/min, wave length = 220 nm): t_R = 11.9 min (minor), t_R = 17.9 min (major); 92% ee.

Lit.[7] $[\alpha]_D^{26}$ = −38° (c = 1.0 in acetone; for (*R*)-enantiomer); HPLC analysis (Chiralcel AD-H; n-hexane/i-PrOH = 80/20, flow rate: 1.0 mL/min, wave length = 220 nm): t_R = 7.0 min (major), t_R = 9.7 min (minor); 80% ee.

<u>Analytical Data for the Reaction Products (5a–5ao, 5b–5z)</u>

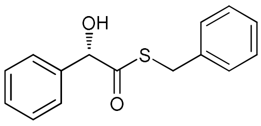

5a

Colorless liquid[8]; TLC (EtOAc/n-hexane, 1/4 v/v): R_f = 0.39; ^{1}H NMR (500 MHz, $CDCl_3$): δ 7.41–7.32 (m, 5H), 7.28–7.20 (m, 5H), 5.20 (d, *J* = 4.4 Hz, 1H), 4.10 (dd, *J* = 34.2, 13.7 Hz, 2H), 3.52 (d, *J* = 4.4 Hz, 1H); ^{13}C NMR (125 MHz, $CDCl_3$): δ 201.38, 137.93, 136.72, 128.99, 128.91, 128.84, 128.69, 127.49, 127.13, 79.91, 33.36; The enantiomeric excess was determined by HPLC using a Chiralpak AD-H (n-hexane/i-PrOH = 96/4, flow rate: 1.0 mL/min, wave length = 220 nm), t_R = 26.0 min (major), t_R = 31.5 min (minor).

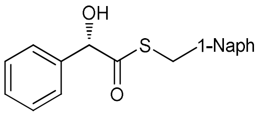

5ab

New compound: Yellow liquid; TLC (EtOAc /n-hexane, 1/4 v/v): R_f = 0.34; $[\alpha]_D^{20}$ = +87.4 (c = 0.64 in $CHCl_3$); ^{1}H NMR (500 MHz, $CDCl_3$): δ 7.87–7.82 (m, 2H), 7.79–7.73 (m, 1H), 7.50–7.44 (m, 3H), 7.41–7.30 (m, 6H), 5.21 (d, *J* = 4.2 Hz, 1H), 4.58 (dd, *J* = 44.4, 13.6 Hz, 2H), 3.47 (d, *J* = 4.4 Hz, 1H); ^{13}C NMR (125 MHz, $CDCl_3$): δ 201.44, 137.90, 133.90, 132.07, 131.36, 128.99, 128.94, 128.84, 128.65, 127.81, 127.13, 126.51, 125.99, 125.43, 123.43, 79.96, 31.18; IR (neat): ν 3424, 3060, 3045, 1681, 1453, 1395, 1095, 1065, 1038, 976, 827, 800, 787, 774, 747, 698, 663, 644; HRMS (m/z, ESI): $[M+Na]^+$ cald. for $C_{19}H_{16}O_2SNa$, 331.0769; found, 331.0764; The enantiomeric excess was determined by HPLC using a Chiralcel OD-H (n-hexane/i-PrOH = 85/15, flow rate: 1.0 mL/min, wave length = 220 nm), t_R = 14.9 min (major), t_R = 23.4 min (minor).

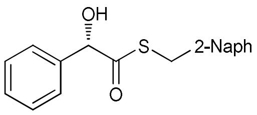

5ac

New compound: White solid; TLC (EtOAc/n-hexane, 1/4 v/v): R_f = 0.33; $[\alpha]_D^{20}$ = +91.6 (c = 0.49 in $CHCl_3$); ^{1}H NMR (500 MHz, $CDCl_3$): δ 7.88–7.64 (m, 4H), 7.54–7.28 (m, 8H), 5.24 (d, *J* = 4.3 Hz, 1H), 4.28 (dd, *J* = 34.9, 13.7 Hz, 2H), 3.45 (d, *J* = 4.4 Hz, 1H); ^{13}C NMR (125 MHz, $CDCl_3$): δ 201.27, 137.91, 134.09, 133.30, 132.63, 129.02, 128.85, 128.53, 127.72, 127.67, 127.66, 127.14, 126.77, 126.34, 126.07, 79.97, 33.62; IR (neat): ν 3415, 3056, 1692, 1680, 1672, 1452, 1189,

1064, 1034, 985, 830, 812, 749, 740, 711, 699, 645; HRMS (m/z, ESI): $[M+Na]^+$ cald. for $C_{19}H_{16}O_2SNa$, 331.0769; found, 331.0765; The enantiomeric excess was determined by HPLC using a Chiralcel OD-H (n-hexane/i-PrOH = 90/10, flow rate: 1.0 mL/min, wave length = 220 nm), t_R = 18.0 min (major), t_R = 22.5 min (minor).

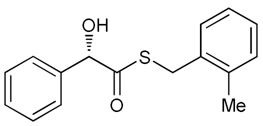

5ad

New compound: Colorless liquid; TLC (EtOAc/n-hexane, 1/4 v/v): R_f = 0.38; $[\alpha]_D^{20}$ = +103 (c = 0.43 in $CHCl_3$); ^1H NMR (500 MHz, $CDCl_3$): δ 7.44–7.32 (m, 5H), 7.24–7.21 (m, 1H), 7.19–7.07 (m, 3H), 5.22 (d, *J* = 4.4 Hz, 1H), 4.14 (dd, *J* = 32.5, 13.4 Hz, 2H), 3.48 (d, *J* = 4.4 Hz, 1H), 2.27 (s, 3H); ^{13}C NMR (125 MHz, $CDCl_3$): δ 201.40, 137.97, 136.73, 134.26, 130.51, 129.98, 128.98, 128.83, 127.89, 127.12, 126.29, 79.95, 31.56, 19.26; IR (neat): ν 3442, 3030, 2916, 1682, 1493, 1453, 1380, 1261, 1189, 1097, 1068, 1050, 1002, 974, 831, 770, 731, 702, 640; HRMS (m/z, ESI): $[M+Na]^+$ cald. for $C_{16}H_{16}O_2SNa$, 295.0769; found, 295.0760; The enantiomeric excess was determined by HPLC using a Chiralcel OD-H (n-hexane/i-PrOH = 95/5, flow rate: 1.0 mL/min, wave length = 220 nm), t_R = 16.9 min (major), t_R = 30.7 min (minor).

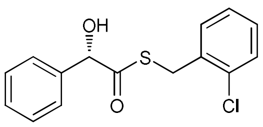

5ae

New compound: Colorless liquid; TLC (EtOAc/n-hexane, 1/4 v/v): R_f = 0.39; $[\alpha]_D^{20}$ = +101 (c = 0.49 in $CHCl_3$); ^1H NMR (500 MHz, $CDCl_3$): δ 7.45–7.29 (m, 7H), 7.22–7.13 (m, 2H), 5.21 (d, *J* = 4.4 Hz, 1H), 4.23 (dd, *J* = 35.4, 13.6 Hz, 2H), 3.45 (d, *J* = 4.5 Hz, 1H); ^{13}C NMR (125 MHz, $CDCl_3$): δ 201.25, 137.84, 134.70, 134.21, 131.16, 129.62, 129.02, 128.84, 127.12, 127.03, 79.92, 31.22; IR (neat): ν 3438, 1678, 1492, 1472, 1447, 1420, 1334, 1120, 1071, 991, 968, 829, 761, 747, 735, 699; HRMS (m/z, ESI): $[M+Na]^+$ cald. for $C_{15}H_{13}ClO_2SNa$, 315.0222; found, 315.0216; The enantiomeric excess was determined by HPLC using a Chiralcel OD-H (n-hexane/i-PrOH = 95/5, flow rate: 1.0 mL/min, wave length = 220 nm), t_R = 16.2 min (major), t_R = 29.2 min (minor).

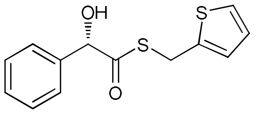

5af

New compound: Colorless liquid; TLC (EtOAc/n-hexane, 1/4 v/v): R_f = 0.34; $[\alpha]_D^{20}$ = +98.2 (c = 0.31 in $CHCl_3$); ^1H NMR (500 MHz, $CDCl_3$): δ 7.46–7.31 (m, 5H), 7.16 (dd, *J* = 5.1, 1.2 Hz, 1H), 6.94–6.91 (m, 1H), 6.88 (dd, *J* = 5.1, 3.5 Hz, 1H), 5.24 (d, *J* = 4.4 Hz, 1H), 4.33 (q, *J* = 14.6 Hz, 2H), 3.42 (d, *J* = 4.4 Hz, 1H); ^{13}C NMR (125 MHz, $CDCl_3$): δ 201.06, 139.15, 137.74, 129.06, 128.86, 127.14,

126.97, 126.89, 125.43, 79.90, 27.85; IR (neat): ν 3445, 3063, 2912, 1682, 1493, 1453, 1238, 1190, 1093, 1068, 1050, 974, 849, 829, 699, 635; HRMS (m/z, ESI): $[M+Na]^+$ cald. for $C_{13}H_{12}O_2S_2Na$, 287.0176; found, 287.0170; The enantiomeric excess was determined by HPLC using a Chiralcel OD-H (n-hexane/i-PrOH = 95/5, flow rate: 1.0 mL/min, wave length = 220 nm), t_R = 23.5 min (major), t_R = 34.0 min (minor).

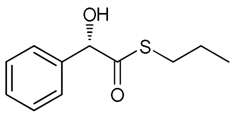

5ag

New compound: Colorless liquid; TLC (EtOAc/n-hexane, 1/4 v/v): R_f = 0.41; $[\alpha]_D^{20}$ = +135 (c = 0.28 in $CHCl_3$); ^1H NMR (500 MHz, $CDCl_3$): δ 7.46–7.32 (m, 5H), 5.22 (d, *J* = 4.4 Hz, 1H), 3.64–3.51 (m, 1H), 2.88 (dtd, *J* = 20.5, 13.2, 7.3 Hz, 2H), 1.58 (dd, *J* = 14.6, 7.3 Hz, 2H), 0.93 (t, *J* = 7.4 Hz, 3H); ^{13}C NMR (125 MHz, $CDCl_3$): δ 201.98, 138.22, 128.84, 128.74, 127.08, 79.95, 30.84, 22.64, 13.29; IR (neat): ν 3438, 3032, 2964, 2932, 2874, 1681, 1494, 1415, 1379, 1278, 1220, 1191, 1097, 1070, 975, 829, 752, 702, 643; HRMS (m/z, ESI): $[M+Na]^+$ cald. for $C_{11}H_{14}O_2SNa$, 233.0612; found, 233.0608; The enantiomeric excess was determined by HPLC using a Chiralcel OD-H (n-hexane/i-PrOH = 95/5, flow rate: 1.0 mL/min, wave length = 220 nm), t_R = 8.8 min (major), t_R = 13.5 min (minor).

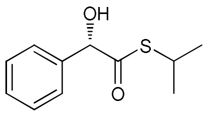

5ah

Colorless liquid[8]; TLC (EtOAc/n-hexane, 1/4 v/v): R_f = 0.46; ^1H NMR (500 MHz, $CDCl_3$): δ 7.44–7.33 (m, 5H), 5.18 (d, *J* = 4.4 Hz, 1H), 3.67 (dt, *J* = 13.8, 6.9 Hz, 1H), 3.60 (t, *J* = 4.2 Hz, 1H), 1.28 (dd, *J* = 16.5, 6.9 Hz, 6H); ^{13}C NMR (125 MHz, $CDCl_3$): δ 201.79, 138.27, 128.86, 128.78, 127.18, 79.96, 35.22, 22.90, 22.84; The enantiomeric excess was determined by HPLC using a Chiralcel OD-H (n-hexane/i-PrOH = 95/5, flow rate: 1.0 mL/min, wave length = 220 nm), t_R = 8.2 min (major), t_R = 15.1 min (minor).

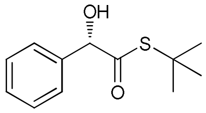

5ai

Colorless liquid[8]; TLC (EtOAc/n-hexane, 1/4 v/v): R_f = 0.51; ^1H NMR (500 MHz, $CDCl_3$): δ 7.42–7.32 (m, 5H), 5.11 (d, *J* = 4.7 Hz, 1H), 3.75 (dd, *J* = 4.7, 1.8 Hz, 2H), 1.45 (s, 9H); ^{13}C NMR (125 MHz, $CDCl_3$): δ 201.94, 138.61, 128.74, 127.26, 79.96, 49.03, 29.86; The enantiomeric excess was determined by HPLC using a Chiralcel OD-H (n-hexane/i-PrOH = 95/5, flow rate: 1.0 mL/min, wave length = 220 nm), t_R = 7.0 min (major), t_R = 13.2 min (minor).

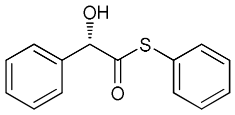

5aj

Colorless liquid[8]; TLC (EtOAc /n-hexane, 1/4 v/v): R_f = 0.30; ^{1}H NMR (500 MHz, $CDCl_3$): δ 7.52–7.45 (m, 2H), 7.44–7.32 (m, 8H), 5.34 (d, *J* = 4.3 Hz, 1H), 3.47 (d, *J* = 4.5 Hz, 1H); ^{13}C NMR (125 MHz, $CDCl_3$): δ 200.13, 137.72, 134.64, 129.67, 129.31, 129.10, 128.92, 127.21, 126.54, 80.03; The enantiomeric excess was determined by HPLC using a Chiralcel OD-H (n-hexane/i-PrOH = 95/5, flow rate: 1.0 mL/min, wave length = 220 nm), t_R = 18.4 min (major), t_R = 22.1 min (minor).

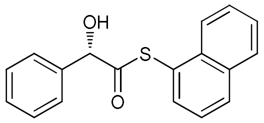

5ak

New compound: White solid; TLC (EtOAc/n-hexane, 1/4 v/v): R_f = 0.28; $[\alpha]_D^{20}$ = +42.4 (c = 0.04 in $CHCl_3$); ^{1}H NMR (500 MHz, $CDCl_3$): δ 7.90 (dd, *J* = 11.4, 8.5 Hz, 2H), 7.84 (d, *J* = 7.6 Hz, 1H), 7.64–7.58 (m, 1H), 7.53–7.35 (m, 8H), 5.38 (s, 1H), 3.60 (s, 1H); ^{13}C NMR (125 MHz, $CDCl_3$): δ 199.90, 137.85, 135.28, 134.25, 134.21, 131.22, 129.15, 128.99, 128.74, 127.32, 127.24, 126.53, 125.63, 125.00, 123.93, 80.17; IR (neat): ν 3447, 3066, 3037, 1689, 1503, 1493, 1454, 1378, 1337, 1271, 1228, 1199, 1096, 1065, 1024, 964, 836, 820, 803, 771, 750, 698, 619; HRMS (m/z, ESI): $[M+Na]^+$ cald. for $C_{18}H_{14}O_2SNa$, 317.0612; found, 317.0610; The enantiomeric excess was determined by HPLC using a Chiralcel OD-H (n-hexane/i-PrOH = 90/10, flow rate: 1.0 mL/min, wave length = 220 nm), t_R = 16.6 min (minor), t_R = 23.6 min (major).

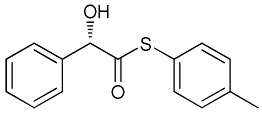

5al

New compound: Colorless liquid; TLC (EtOAc /n-hexane, 1/4 v/v): R_f = 0.30; $[\alpha]_D^{20}$ = +33.3 (c = 0.15 in $CHCl_3$); ^{1}H NMR (500 MHz, $CDCl_3$): δ 7.49–7.46 (m, 2H), 7.44–7.36 (m, 3H), 7.25–7.18 (m, 4H), 5.34 (d, *J* = 4.5 Hz, 1H), 3.46 (d, *J* = 4.5 Hz, 1H), 2.36 (s, 3H); ^{13}C NMR (125 MHz, $CDCl_3$): δ 200.61, 140.05, 137.79, 134.58, 130.18, 129.07, 128.91, 127.24, 122.86, 79.97, 21.38; IR (neat): ν 3275, 3173, 2920, 2866, 1697, 1493, 1447, 1321, 1239, 1179, 1118, 1092, 1071, 810, 701, 654, 637; HRMS (m/z, ESI): $[M+Na]^+$ cald. for $C_{15}H_{14}O_2SNa$, 281.0612; found, 281.0609; The enantiomeric excess was determined by HPLC using a Chiralcel OD-H (n-hexane/i-PrOH = 95/5, flow rate: 1.0 mL/min, wave length = 220 nm), t_R = 24.1 min (major), t_R = 29.2 min (minor).

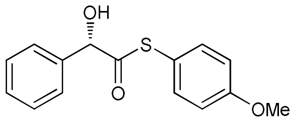

5am

Colorless liquid[8]; TLC (EtOAc /n-hexane, 1/4 v/v): R_f = 0.32; ^{1}H NMR (500 MHz, $CDCl_3$): δ 7.49–7.46 (m, 2H), 7.44–7.36 (m, 3H), 7.28–7.24 (m, 2H), 6.94–6.89 (m, 2H), 5.34 (s, 1H), 3.81 (s, 3H), 3.47 (bs, 1H); ^{13}C NMR (125 MHz, $CDCl_3$): δ 200.99, 160.88, 137.82, 136.19, 129.04, 128.88, 127.22, 116.88, 115.02, 79.93, 55.37; The enantiomeric excess was determined by HPLC using a Regis (*R*,*R*) Whelk-O1 (n-hexane/i-PrOH = 90/10, flow rate: 1.0 mL/min, wave length = 220 nm), t_R = 19.1 min (major), t_R = 28.7 min (minor).

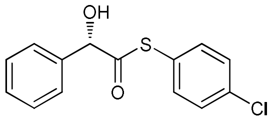

5an

Colorless liquid[8]; TLC (EtOAc /n-hexane, 1/4 v/v): R_f = 0.35; ^{1}H NMR (500 MHz, $CDCl_3$): δ 7.50–7.46 (m, 2H), 7.45–7.39 (m, 3H), 7.38–7.35 (m, 2H), 7.30–7.27 (m, 2H), 5.35 (s, 1H), 3.35 (s, 1H); ^{13}C NMR (125 MHz, $CDCl_3$): δ 199.74, 137.47, 136.13, 135.87, 129.58, 129.23, 128.99, 127.14, 125.04, 80.05; The enantiomeric excess was determined by HPLC using a Chiralcel OD-H (n-hexane/i-PrOH = 95/5, flow rate: 1.0 mL/min, wave length = 220 nm), t_R = 23.0 min (major), t_R = 27.1 min (minor).

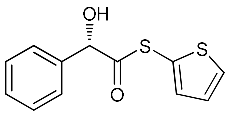

5ao

New compound: Colorless liquid; TLC (EtOAc/n-hexane, 1/4 v/v): R_f = 0.30; $[\alpha]_D^{20}$ = +3.3 (c = 0.01 in $CHCl_3$); ^{1}H NMR (500 MHz, $CDCl_3$): δ 7.58–7.52 (m, 1H), 7.48–7.35 (m, 5H), 7.13–7.05 (m, 2H), 5.33 (s, 1H), 3.44 (s, 1H); ^{13}C NMR (125 MHz, $CDCl_3$): δ 200.08, 137.24, 136.09, 132.11, 129.22, 128.99, 127.92, 127.15, 123.35, 79.92; IR (neat): ν 3438, 3126, 3064, 2925, 1708, 1494, 1453, 1402, 1267, 1220, 1189, 1087, 1066, 1041, 960, 909, 733, 701; HRMS (m/z, ESI): $[M+Na]^+$ cald. for $C_{12}H_{10}O_2S_2Na$, 273.0020; found, 273.0013; The enantiomeric excess was determined by HPLC using a Chiralcel OD-H (n-hexane/i-PrOH = 95/5, flow rate: 1.0 mL/min, wave length = 220 nm), t_R = 26.1 min (major), t_R = 28.3 min (minor).

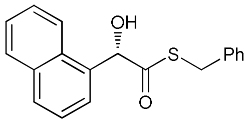

5b

New compound: Colorless liquid; TLC (EtOAc/n-hexane, 1/4 v/v): R_f = 0.33; $[\alpha]_D^{20}$ = +114 (c = 0.65 in $CHCl_3$); ^{1}H NMR (500 MHz, $CDCl_3$): δ 8.16–8.03 (m, 1H), 7.94–7.81 (m, 2H), 7.61–7.39 (m, 4H), 7.28–7.15 (m, 5H), 5.81 (d, *J* = 3.6 Hz, 1H), 4.12 (dd, *J* = 55.7, 13.7 Hz, 2H), 3.60 (d, *J* = 3.7 Hz, 1H); ^{13}C NMR (125 MHz, $CDCl_3$): δ 201.92, 136.78, 134.18, 133.50, 131.01, 130.03, 128.93, 128.90, 128.64, 127.45, 127.36, 126.78, 126.07, 125.24, 123.94, 78.89, 33.46; IR (neat): ν 3456, 3053,

3026, 1666, 1511, 1493, 1454, 1290, 1206, 1164, 1099, 1079, 1084, 782, 717, 705, 696, 645; HRMS (m/z, ESI): $[M+Na]^+$ cald. for $C_{19}H_{16}O_2SNa$, 331.0769; found, 331.0762; The enantiomeric excess was determined by HPLC using a Chiralcel OD-H (n-hexane/i-PrOH = 90/10, flow rate: 1.0 mL/min, wave length = 220 nm), t_R = 23.2min (minor), t_R = 32.3 min (major).

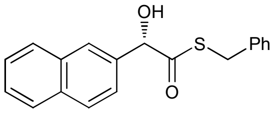

5c

New compound: Colorless liquid; TLC (EtOAc/n-hexane, 1/4 v/v): R_f = 0.34; $[\alpha]_D^{20}$ = +129 (c = 0.25 in $CHCl_3$); ^1H NMR (500 MHz, $CDCl_3$): δ 7.93–7.80 (m, 4H), 7.55–7.45 (m, 3H), 7.31–7.19 (m, 5H), 5.41 (d, *J* = 3.9 Hz, 1H), 4.13 (dd, *J* = 44.3, 13.7 Hz, 2H), 3.61 (d, *J* = 4.2 Hz, 1H); ^{13}C NMR (125 MHz, $CDCl_3$): δ 201.29, 136.60, 135.21, 133.54, 133.16, 128.90, 128.84, 128.69, 128.20, 127.81, 127.51, 126.95, 126.62, 126.50, 124.17, 80.07, 33.46; IR (neat): ν 3438, 3059, 1654, 1278, 1092, 1057, 899, 865, 826, 789, 771, 755, 712, 698, 632; HRMS (m/z, ESI): $[M+Na]^+$ cald. for $C_{19}H_{16}O_2SNa$, 331.0769; found, 331.0765; The enantiomeric excess was determined by HPLC using a Chiralpak AD-H (n-hexane/i-PrOH = 96/4, flow rate: 1.0 mL/min, wave length = 220 nm), t_R = 45.7 min (major), t_R = 51.1 min (minor).

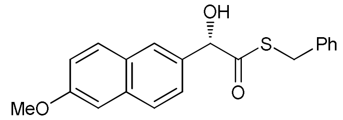

5d

New compound: White solid; TLC (EtOAc/n-hexane, 1/4 v/v): R_f = 0.25; $[\alpha]_D^{20}$ = +132 (c = 0.38 in $CHCl_3$); ^1H NMR (500 MHz, $CDCl_3$): δ 7.78 (s, 1H), 7.72 (t, *J* = 8.5 Hz, 2H), 7.43 (dd, *J* = 8.5, 1.6 Hz, 1H), 7.29–7.19 (m, 5H), 7.15 (dd, *J* = 8.9, 2.5 Hz, 1H), 7.11 (d, *J* = 2.3 Hz, 1H), 5.34 (d, *J* = 4.2 Hz, 1H), 4.11 (dd, *J* = 41.3, 13.7 Hz, 2H), 3.90 (s, 3H), 3.59 (d, *J* = 4.2 Hz, 1H); ^{13}C NMR (125 MHz, $CDCl_3$): δ 201.47, 158.23, 136.71, 134.83, 132.98, 129.68, 128.90, 128.67, 128.63, 127.62, 127.47, 126.79, 124.80, 119.33, 105.76, 80.05, 55.36, 33.43; IR (neat): ν 3479, 3032, 2926, 1671, 1635, 1607, 1485, 1391, 1269, 1230, 1195, 1177, 1073, 1028, 961, 896, 857, 840, 807, 708, 694, 662; HRMS (m/z, ESI): $[M+Na]^+$ cald. for $C_{20}H_{18}O_3SNa$, 361.0874; found, 361.0867; The enantiomeric excess was determined by HPLC using a Chiralcel OD-H (n-hexane/i-PrOH = 90/10, flow rate: 1.0 mL/min, wave length = 220 nm), t_R = 21.3 min (minor), t_R = 24.8 min (major).

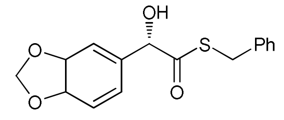

5e

New compound: Colorless liquid; TLC (EtOAc/n-hexane, 1/4 v/v): R_f = 0.24; $[\alpha]_D^{20}$ = +84.6 (c = 0.48 in $CHCl_3$); ^1H NMR (500 MHz, $CDCl_3$): δ 7.31–7.20 (m, 5H), 6.89–6.83 (m, 2H), 6.79–6.76 (m, 1H), 5.95 (s, 2H), 5.11 (s, 1H), 4.11 (dd, *J* = 32.6, 13.7 Hz, 2H), 3.56 (s, 1H); ^{13}C NMR (125 MHz, $CDCl_3$): δ 201.48, 148.24, 148.10, 136.71, 131.71, 128.92, 128.71, 127.51, 121.32, 108.43, 107.31, 101.35, 79.58, 33.42; IR (neat): ν 3437, 3078, 3029, 2897, 1652, 1503, 1491, 1441, 1243, 1090, 1039, 927, 861, 737, 716, 693; HRMS (m/z, ESI): $[M+Na]^+$ cald. for $C_{16}H_{14}O_4SNa$, 325.0510; found, 325.0506; The enantiomeric excess was determined by HPLC using a Chiralcel OD-H (n-hexane/i-PrOH = 95/5, flow rate: 1.0 mL/min, wave length = 220 nm), t_R = 33.2 min (major), t_R = 37.0 min (minor).

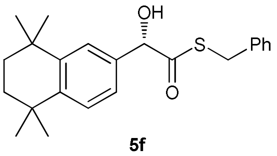

5f

New compound: Yellow liquid; TLC (EtOAc/n-hexane, 1/4 v/v): R_f = 0.51; $[\alpha]_D^{20}$ = +56.8 (c = 0.45 in $CHCl_3$); ^1H NMR (500 MHz, $CDCl_3$): δ 7.33–7.27 (m, 5H), 7.26–7.20 (m, 2H), 7.15–7.13 (m, 1H), 5.21–5.18 (m, 1H), 4.13 (q, *J* = 13.5 Hz, 2H), 1.67 (s, 3H), 1.26–1.22 (m, 12H); ^{13}C NMR (125 MHz, $CDCl_3$): δ 201.50, 145.75, 145.42, 136.99, 134.91, 128.86, 128.63, 127.38, 127.11, 125.45, 124.03, 79.99, 34.99, 34.95, 34.35, 34.21, 33.17, 31.80; IR (neat): ν 3463, 2962, 2930, 2863, 1679, 1495, 1455, 1411, 1363, 1263, 1182, 1092, 1068, 1047, 980, 908, 805, 731, 703, 651; HRMS (m/z, ESI): $[M+Na]^+$ cald. for $C_{23}H_{28}O_2SNa$, 391.1708; found, 391.1702; The enantiomeric excess was determined by HPLC using a Chiralcel OD-H (n-hexane/i-PrOH = 99/1, flow rate: 1.0 mL/min, wave length = 220 nm), t_R = 29.7 min (major), t_R = 37.0 min (minor).

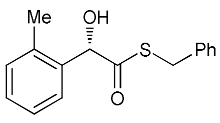

5g

New compound: Colorless liquid; TLC (EtOAc/n-hexane, 1/4 v/v): R_f = 0.43; $[\alpha]_D^{20}$ = +113 (c = 0.59 in $CHCl_3$); ^1H NMR (500 MHz, $CDCl_3$): δ 7.32–7.27 (m, 2H), 7.26–7.13 (m, 7H), 5.40 (s, 1H), 4.14 (dd, *J* = 35.6, 13.7 Hz, 2H), 3.43 (s, 1H), 2.39 (s, 3H); ^{13}C NMR (125 MHz, $CDCl_3$): δ 201.68, 136.87, 136.77, 136.07, 131.12, 129.01, 128.88, 128.65, 128.09, 127.45, 126.40, 77.81, 33.35, 19.44; IR (neat): ν 3455, 3068, 3028, 2923, 1687, 1680, 1461, 1453, 1377, 1280, 1196, 1116, 1074, 1050, 978, 908, 760, 733, 702; HRMS (m/z, ESI): $[M+Na]^+$ cald. for $C_{16}H_{16}O_2SNa$, 295.0769; found, 295.0763; The enantiomeric excess was determined by HPLC using a Chiralpak AD-H (n-hexane/i-PrOH = 96/4, flow rate: 1.0 mL/min, wave length = 220 nm), t_R = 20.2 min

(major), t_R = 23.4 min (minor).

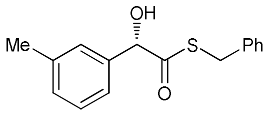

5h

New compound: Colorless liquid; TLC (EtOAc/n-hexane, 1/4 v/v): R_f = 0.37; $[\alpha]_D^{20}$ = +98.7 (c = 0.43 in $CHCl_3$); ^1H NMR (500 MHz, $CDCl_3$): δ 7.30–7.12 (m, 9H), 5.17 (d, *J* = 3.1 Hz, 1H), 4.11 (dd, *J* = 36.9, 13.7 Hz, 2H), 3.48 (d, *J* = 3.6 Hz, 1H), 2.34 (s, 3H); ^{13}C NMR (125 MHz, $CDCl_3$): δ 201.45, 138.63, 137.88, 136.77, 129.78, 128.92, 128.72, 128.68, 127.70, 127.47, 124.28, 79.94, 33.34, 21.44; IR (neat): ν 3454, 3032, 2953, 2923, 2857, 1680, 1605, 1453, 1379, 1242, 1073, 1051, 977, 907, 774, 718, 700, 643; HRMS (m/z, ESI): $[M+Na]^+$ cald. for $C_{16}H_{16}O_2SNa$, 295.0769; found, 295.0763; The enantiomeric excess was determined by HPLC using a Chiralpak AD-H (n-hexane/i-PrOH = 96/4, flow rate: 1.0 mL/min, wave length = 220 nm), t_R = 23.2 min (major), t_R = 27.1 min (minor).

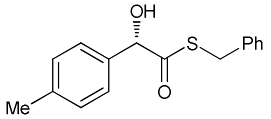

5i

New compound: Colorless liquid; TLC (EtOAc/n-hexane, 1/4 v/v): R_f = 0.33; $[\alpha]_D^{20}$ = +110 (c = 0.38 in $CHCl_3$); ^1H NMR (500 MHz, $CDCl_3$): δ 7.32–7.23 (m, 7H), 7.19–7.17 (m, 2H), 5.20 (d, *J* = 4.4 Hz, 1H), 4.12 (dd, *J* = 41.1, 13.7 Hz, 2H), 3.42 (d, *J* = 4.4 Hz, 1H), 2.35 (s, 1H); ^{13}C NMR (125 MHz, $CDCl_3$): δ 201.46, 138.95, 136.73, 135.01, 129.54, 128.89, 128.66, 127.45, 127.09, 79.78, 33.36, 21.26; IR (neat): ν 3438, 3060, 3031, 2882, 1653, 1513, 1494, 1453, 1407, 1274, 1245, 1219, 1092, 1059, 1029, 826, 767, 733, 694; HRMS (m/z, ESI): $[M+Na]^+$ cald. for $C_{16}H_{16}O_2SNa$, 295.0769; found, 295.0763; The enantiomeric excess was determined by HPLC using a Chiralpak AD-H (n-hexane/i-PrOH = 96/4, flow rate: 1.0 mL/min, wave length = 220 nm), t_R = 25.1 min (major), t_R = 30.1 min (minor).

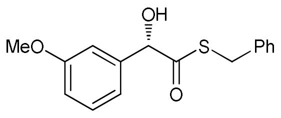

5j

New compound: Colorless liquid; TLC (EtOAc/n-hexane, 1/4 v/v): R_f = 0.24; $[\alpha]_D^{20}$ = +80.8 (c = 0.48 in $CHCl_3$); ^1H NMR (500 MHz, $CDCl_3$): δ 7.26 (dd, *J* = 9.8, 5.5 Hz, 7H), 7.00 (d, *J* = 7.6 Hz, 1H), 6.97–6.92 (m, 1H), 6.88 (ddd, *J* = 8.3, 2.6, 0.8 Hz, 1H), 5.20 (s, 1H), 4.12 (dd, *J* = 37.4, 13.7 Hz, 2H), 3.79 (s, 3H), 3.47 (s, 1H); ^{13}C NMR (125 MHz, $CDCl_3$): δ 201.16, 159.92, 139.37, 136.69, 129.86, 128.90, 128.67, 127.48, 119.49, 114.78, 112.27, 79.81, 55.30, 33.37; IR (neat): ν 3447, 3062, 3026, 2924, 2836, 1683, 1600, 1586, 1490, 1464, 1454, 1436, 1260, 1154, 1073, 1043, 979, 772, 719, 699; HRMS (m/z, ESI): $[M+Na]^+$ cald. for $C_{16}H_{16}O_3SNa$,

311.0718; found, 311.0713; The enantiomeric excess was determined by HPLC using a Chiralpak AD-H (n-hexane/i-PrOH = 96/4, flow rate: 1.0 mL/min, wave length = 220 nm), t_R = 38.9 min (major), t_R = 44.7 min (minor).

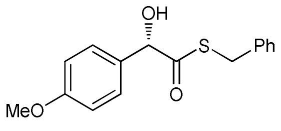

5k

New compound: Colorless liquid; TLC (EtOAc/n-hexane, 1/4 v/v): R_f = 0.20; $[\alpha]_D^{20}$ = +112 (c = 0.32 in $CHCl_3$); ^1H NMR (500 MHz, $CDCl_3$): δ 7.34–7.19 (m, 7H), 6.94–6.84 (m, 2H), 5.16 (d, *J* = 3.8 Hz, 1H), 4.11 (dd, *J* = 36.8, 13.7 Hz, 2H), 3.79 (s, 3H), 3.49–3.42 (m, 1H); ^{13}C NMR (125 MHz, $CDCl_3$): δ 201.62, 160.16, 136.79, 130.07, 128.90, 128.67, 128.56, 127.46, 114.25, 79.49, 55.31, 33.37; IR (neat): ν 3483, 3062, 3036, 2960, 1654, 1611, 1587, 1514, 1495, 1454, 1380, 1255, 1240, 1178, 1079, 1048, 1031, 837, 777, 750, 709, 697; HRMS (m/z, ESI): $[M+Na]^+$ cald. for $C_{16}H_{16}O_3SNa$, 311.0718; found, 311.0710; The enantiomeric excess was determined by HPLC using a Chiralcel OD-H (n-hexane/i-PrOH = 95/5, flow rate: 1.0 mL/min, wave length = 220 nm), t_R = 30.8 min (major), t_R = 38.8 min (minor).

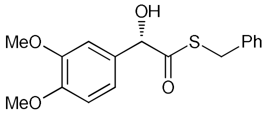

5l

New compound: Colorless liquid; TLC (EtOAc/n-hexane, 1/4 v/v): R_f = 0.08; $[\alpha]_D^{20}$ = +81.0 (c = 0.21 in $CHCl_3$); ^1H NMR (500 MHz, $CDCl_3$): δ 7.35–7.17 (m, 5H), 6.96 (dd, *J* = 8.2, 1.9 Hz, 1H), 6.87 (d, *J* = 1.9 Hz, 1H), 6.84 (d, *J* = 8.2 Hz, 1H), 5.17 (d, *J* = 4.0 Hz, 1H), 4.12 (dd, *J* = 38.5, 13.7 Hz, 2H), 3.86 (d, *J* = 13.8 Hz, 6H), 3.51 (d, *J* = 4.1 Hz, 1H); ^{13}C NMR (125 MHz, $CDCl_3$): δ 201.55, 149.59, 149.28, 136.82, 130.33, 128.89, 128.68, 127.49, 120.09, 110.98, 109.55, 79.65, 55.91, 55.89, 33.36; IR (neat): ν 3494, 3079, 3040, 2935, 2841, 1679, 1596, 1514, 1456, 1342, 1259, 1237, 1184, 1136, 1100, 1021, 853, 817, 766, 696; HRMS (m/z, ESI): $[M+Na]^+$ cald. for $C_{17}H_{18}O_4SNa$, 341.0823; found, 341.0818; The enantiomeric excess was determined by HPLC using a Chiralpak AD-H (n-hexane/i-PrOH = 85/15, flow rate: 1.0 mL/min, wave length = 220 nm), t_R = 17.2 min (major), t_R = 20.2 min (minor).

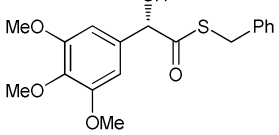

5m

New compound: Yellow liquid; TLC (EtOAc/n-hexane, 1/4 v/v): R_f = 0.06; $[\alpha]_D^{20}$ = +76.5 (c = 0.26 in $CHCl_3$); ^1H NMR (500 MHz, $CDCl_3$): δ 7.33–7.20 (m, 5H), 6.61 (s, 2H), 5.16 (d, *J* = 4.2 Hz, 1H), 4.13 (dd, *J* = 31.5, 13.7 Hz, 2H), 3.83 (s, 9H), 3.54 (d, *J* = 4.2 Hz, 1H); ^{13}C NMR (125 MHz, $CDCl_3$): δ 201.22, 153.48, 138.28, 136.82, 133.41, 128.89, 128.69,

127.51, 103.95, 79.84, 60.88, 56.12, 33.31; IR (neat): ν 3420, 2939, 2836, 1673, 1593, 1504, 1462, 1454, 1417, 1323, 1241, 1123, 1109, 1074, 991, 703, 631; HRMS (m/z, ESI): $[M+Na]^+$ cald. for $C_{18}H_{20}O_5SNa$, 371.0929; found, 31.0926; The enantiomeric excess was determined by HPLC using a Chiralpak AD-H (n-hexane/i-PrOH = 85/15, flow rate: 1.0 mL/min, wave length = 220 nm), t_R = 15.4 min (major), t_R = 27.1 min (minor).

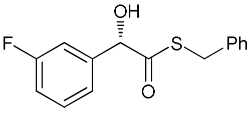

5n

New compound: Colorless liquid; TLC (EtOAc/n-hexane, 1/4 v/v): R_f = 0.33; $[\alpha]_D^{20}$ = +68.0 (c = 0.46 in $CHCl_3$); ^1H NMR (500 MHz, $CDCl_3$): δ 7.38–7.32 (m, 1H), 7.30–7.18 (m, 6H), 7.17–7.12 (m, 1H), 7.08–7.01 (m, 1H), 5.23 (d, J = 4.4 Hz, 1H), 4.13 (q, J = 13.7 Hz, 2H), 3.50 (d, J = 4.4 Hz, 1H); ^{13}C NMR (125 MHz, $CDCl_3$): δ 200.73, 162.93 (d, ^1J(C-F) = 247.1 Hz), 140.20 (d, ^3J(C-C-C-F) = 7.1 Hz), 136.47, 130.33 (d, ^3J(C-C-C-F) = 8.2 Hz), 128.88, 128.72, 127.58, 122.78 (d, ^4J(C-C-C-C-F) = 3.0 Hz), 115.93 (d, ^2J(C-C-F) = 21.2 Hz), 114.01 (d, ^2J(C–F) = 22.6 Hz), 79.19 (d, ^4J(C-C-C-C-F) = 1.9 Hz), 33.43; ^{19}F NMR (470 MHz, $CDCl_3$): δ −111.96 (s, 1F); IR (neat): ν 3452, 3075, 3031, 1679, 1613, 1592, 1487, 1452, 1248, 1136, 1098, 1072, 982, 920, 877, 768, 721, 698, 650, 638; HRMS (m/z, ESI): $[M+Na]^+$ cald. for $C_{15}H_{13}FO_2SNa$, 299.0518; found, 299.0512; The enantiomeric excess was determined by HPLC using a Chiralcel OD-H (n-hexane/i-PrOH = 95/5, flow rate: 1.0 mL/min, wave length = 220 nm), t_R = 17.0 min (major), t_R = 19.5 min (minor).

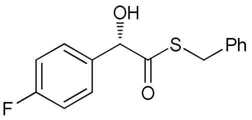

5o

New compound: Colorless liquid; TLC (EtOAc/n-hexane, 1/4 v/v): R_f = 0.32; $[\alpha]_D^{20}$ = +101 (c = 0.41 in $CHCl_3$); ^1H NMR (500 MHz, $CDCl_3$): δ 7.42–7.33 (m, 2H), 7.31–7.19 (m, 5H), 7.10–7.01 (m, 2H), 5.20 (s, 1H), 4.12 (q, J = 13.7 Hz, 2H), 3.53 (s, 1H); ^{13}C NMR (125 MHz, $CDCl_3$): δ 201.22, 163.08 (d, ^1J(C-F) = 247.9 Hz), 136.59, 133.71 (d, ^4J(C-C-C-C-F) = 3.2 Hz), 128.95 (d, ^3J(C-C-C-F) = 8.4 Hz), 128.88, 128.71, 127.55, 115.80 (d, ^2J(C-C-F) = 21.7 Hz), 79.15, 33.40; ^{19}F NMR (470 MHz, $CDCl_3$): δ −112.55 (s, 1F); IR (neat): ν 3454, 3082, 3029, 2933, 2889, 1656, 1602, 1509, 1454, 1274, 1257, 1088, 1057, 839, 770, 740, 697; HRMS (m/z, ESI): $[M+Na]^+$ cald. for $C_{15}H_{13}FO_2SNa$, 299.0518; found, 299.0511; The enantiomeric excess was determined by HPLC using a Chiralpak AD-H (n-hexane/i-PrOH = 96/4, flow rate: 1.0 mL/min, wave length = 220 nm), t_R = 22.6 min (major), t_R = 29.2 min (minor).

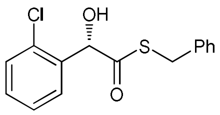

5p

New compound: Yellow liquid; TLC (EtOAc/n-hexane, 1/4 v/v): R_f = 0.35; $[\alpha]_D^{20}$ = +150 (c = 0.55 in $CHCl_3$); ^1H NMR (500 MHz, $CDCl_3$): δ 7.41–7.37 (m, 2H), 7.33–7.26 (m, 5H), 7.26–7.19 (m, 1H), 5.66 (d, *J* = 5.0 Hz, 1H), 4.16 (dd, *J* = 39.4, 13.7 Hz, 1H), 3.67 (d, *J* = 5.0 Hz, 1H); ^{13}C NMR (125 MHz, $CDCl_3$): δ 200.42, 136.56, 135.79, 133.73, 130.20, 130.05, 129.38, 128.90, 128.67, 127.51, 127.35, 77.22, 33.45; IR (neat): ν 3428, 2922, 2853, 1685, 1495, 1454, 1442, 1259, 1193, 1091, 1073, 1056, 1035, 979, 803, 753, 728, 699, 662, 639; HRMS (m/z, ESI): $[M+Na]^+$ cald. for $C_{15}H_{13}ClO_2SNa$, 315.0222; found, 315.0217; The enantiomeric excess was determined by HPLC using a Chiralpak AD-H (n-hexane/i-PrOH = 96/4, flow rate: 1.0 mL/min, wave length = 220 nm), t_R = 24.2 min (major), t_R = 28.3 min (minor).

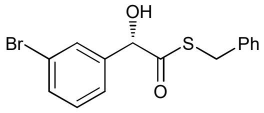

5q

New compound: Yellow liquid; TLC (EtOAc/n-hexane, 1/4 v/v): R_f = 0.32; $[\alpha]_D^{20}$ = +61.5 (c = 0.49 in $CHCl_3$); ^1H NMR (500 MHz, $CDCl_3$): δ 7.58–7.56 (m, 1H), 7.50–7.46 (m, 1H), 7.37–7.34 (m, 1H), 7.32–7.26 (m, 4H), 7.25–7.23 (m, 2H), 5.20 (d, *J* = 3.4 Hz, 1H), 4.14 (q, *J* = 13.7 Hz, 2H), 3.51 (d, *J* = 4.0 Hz, 1H); ^{13}C NMR (125 MHz, $CDCl_3$): δ 200.65, 139.98, 136.42, 132.03, 130.28, 130.03, 128.87, 128.72, 127.58, 125.74, 122.86, 79.09, 33.46; IR (neat): ν 3445, 2953, 2924, 2854, 1682, 1571, 1495, 1454, 1427, 1376, 1260, 1187, 1099, 1072, 1047, 978, 907, 792, 732, 700; HRMS (m/z, ESI): $[M+Na]^+$ cald. for $C_{15}H_{13}BrO_2SNa$, 358.9717; found, 358.9712; The enantiomeric excess was determined by HPLC using a Chiralcel OZ-H (n-hexane/i-PrOH = 98/2, flow rate: 1.0 mL/min, wave length = 220 nm), t_R = 12.3 min (minor), t_R = 15.7 min (major).

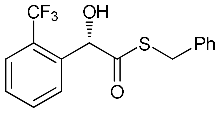

5r

New compound: Colorless liquid; TLC (EtOAc/n-hexane, 1/4 v/v): R_f = 0.37; $[\alpha]_D^{20}$ = +99.4 (c = 0.34 in $CHCl_3$); ^1H NMR (500 MHz, $CDCl_3$): δ 7.73–7.67 (m, 1H), 7.58–7.53 (m, 1H), 7.51–7.43 (m, 2H), 7.32–7.21 (m, 5H), 5.62 (d, *J* = 4.4 Hz, 1H), 4.17 (q, *J* = 13.7 Hz, 2H), 3.61 (d, *J* = 4.4 Hz, 1H); ^{13}C NMR (125 MHz, $CDCl_3$): δ 200.57, 136.80 (q, ^4J(C-C-C-C-F) = 1.2 Hz), 136.47, 132.50, 129.33, 129.06, 128.87, 128.68, 127.54, 126.11 (q, ^2J(C-C-F) = 5.5 Hz), 123.99 (q, ^1J(C-F) = 272.5 Hz), 75.17 (d, ^4J(C-C-C-C-F) = 2.2 Hz), 33.49; ^{19}F NMR (470 MHz, $CDCl_3$): δ −57.25 (s, 3F); IR (neat): ν 3475, 3064, 3031, 1684, 1496, 1454, 1313, 1161, 1120, 1061, 1035, 979, 829, 768, 701, 659; HRMS (m/z, ESI): $[M+Na]^+$ cald. for $C_{16}H_{13}F_3O_2SNa$, 349.0486; found,

39.0482; The enantiomeric excess was determined by HPLC using a Chiralpak AD-H (n-hexane/i-PrOH = 96/4, flow rate: 1.0 mL/min, wave length = 220 nm), t_R = 21.9 min (major), t_R = 26.8 min (minor).

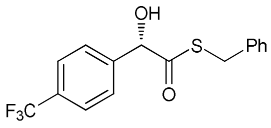

5s

New compound: White solid; TLC (EtOAc/n-hexane, 1/4 v/v): R_f = 0.36; $[\alpha]_D^{20}$ = +60.9 (c = 0.47 in $CHCl_3$); ^1H NMR (500 MHz, $CDCl_3$): δ 7.59 (dd, *J* = 39.2, 8.2 Hz, 4H), 7.39–7.14 (m, 5H), 5.29 (d, *J* = 3.8 Hz, 1H), 4.13 (q, *J* = 13.7 Hz, 2H), 3.57 (d, *J* = 4.2 Hz, 1H); ^{13}C NMR (125 MHz, $CDCl_3$): δ 200.63, 141.56 (q, ^4J(C-C-C-C-F) = 1.1 Hz), 136.37, 131.03 (q, ^2J(C-C-F) = 32.4 Hz), 128.87, 128.73, 127.62, 127.32, 125.70 (q, ^3J(C-C-C-F) = 3.8 Hz), 123.92 (d, ^1J(C-F) = 270.6 Hz), 79.19, 33.44; ^{19}F NMR (470 MHz, $CDCl_3$): δ −62.65 (s, 3F); IR (neat): ν 3419, 1687, 1663, 1496, 1416, 1334, 1168, 1120, 1083, 1071, 1016, 991, 814, 774, 752, 722, 706; HRMS (m/z, ESI): $[M+Na]^+$ cald. for $C_{16}H_{13}F_3O_2SNa$, 349.0486; found, 349.0479; The enantiomeric excess was determined by HPLC using a Chiralpak AD-H (n-hexane/i-PrOH = 96/4, flow rate: 1.0 mL/min, wave length = 220 nm), t_R = 19.2 min (major), t_R = 29.1 min (minor).

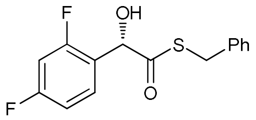

5t

New compound: Colorless liquid; TLC (EtOAc/n-hexane, 1/4 v/v): R_f = 0.32; $[\alpha]_D^{20}$ = +158 (c = 0.29 in $CHCl_3$); ^1H NMR (500 MHz, $CDCl_3$): δ 7.35 (td, *J* = 8.4, 6.3 Hz, 1H), 7.31–7.22 (m, 5H), 6.96–6.77 (m, 2H), 5.45 (d, *J* = 4.8 Hz, 1H), 4.16 (dd, *J* = 33.3, 13.7 Hz, 2H), 3.62–3.57 (m, 1H); ^{13}C NMR (125 MHz, $CDCl_3$): δ 200.29, 163.34 (dd, $^{1,3}J$(C-F) = 251.0, 12.1 Hz), 160.82 (dd, $^{1,3}J$(C-F) = 251.2, 12.2 Hz), 136.41, 130.10 (dd, $^{3,3}J$(C-C-C-F) = 10.0, 5.1 Hz), 128.89, 128.73, 127.61, 121.63 (dd, $^{2,4}J$(C-C-F) = 13.7, 3.9 Hz), 111.95 (dd, $^{2,4}J$(C-C-F) = 21.6, 3.7 Hz), 104.39 (t, $^{2,2}J$(C-C-F) = 25.4 Hz), 73.67 (d, ^3J(C-C-C-F) = 2.2 Hz), 33.48; ^{19}F NMR (470 MHz, $CDCl_3$): δ −108.22 (d, *J* = 8.1 Hz), −113.17 (d, *J* = 8.1 Hz); IR (neat): ν 3437, 3078, 3031, 2927, 1683, 1618, 1605, 1503, 1454, 1431, 1283, 1141, 1099, 1047, 965, 851, 807, 767, 701, 635; HRMS (m/z, ESI): $[M+Na]^+$ cald. for $C_{15}H_{12}F_2O_2SNa$, 317.0424; found, 317.0416; The enantiomeric excess was determined by HPLC using a Chiralpak AD-H (n-hexane/i-PrOH = 96/4, flow rate: 1.0 mL/min, wave length = 220 nm), t_R = 20.9 min (major), t_R = 25.2 min (minor).

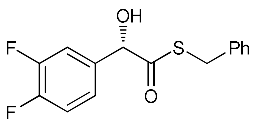

5u

New compound: Colorless liquid; TLC (EtOAc/n-hexane, 1/4 v/v): R_f = 0.31; $[\alpha]_D^{20}$ = +61.6 (c = 0.46 in $CHCl_3$); ^1H NMR (500 MHz, $CDCl_3$): δ 7.33–7.22 (m, 6H), 7.19–7.12 (m, 2H), 5.20 (s, 1H), 4.13 (q, *J* = 13.7 Hz, 2H), 3.56 (s, 1H); ^{13}C NMR (125 MHz, $CDCl_3$): δ 200.66, 151.57 (dd, $^{1,2}J$(C-F) = 25.2, 12.6 Hz), 149.58 (dd, $^{1,2}J$(C-F) = 24.7, 12.7 Hz), 136.36, 134.69 (dd, $^{3,4}J$(C-C-C-F) = 5.2, 4.1 Hz), 128.88, 128.76, 127.65, 123.32 (dd, $^{3,4}J$(C-C-C-F) = 6.5, 3.7 Hz), 117.58 (d, $^{2,3}J$(C-C-F) = 17.5 Hz), 116.10 (d, $^{2,3}J$(C-C-F) = 18.3 Hz), 78.62, 33.48; ^{19}F NMR (470 MHz, $CDCl_3$): δ −136.37 (d, *J* = 21.0 Hz), −137.10 (d, *J* = 21.0 Hz); IR (neat): ν 3445, 3062, 3033, 2916, 2848, 1679, 1612, 1516, 1434, 1283, 1210, 1116, 1093, 1072, 983, 809, 769, 725, 701; HRMS (m/z, ESI): $[M+Na]^+$ cald. for $C_{15}H_{12}F_2O_2SNa$, 317.0424; found, 317.0417; The enantiomeric excess was determined by HPLC using a Chiralpak AD-H (n-hexane/i-PrOH = 96/4, flow rate: 1.0 mL/min, wave length = 220 nm), t_R = 19.1 min (major), t_R = 22.5 min (minor).

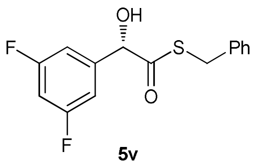

5v

New compound: Yellow liquid; TLC (EtOAc/n-hexane, 1/4 v/v): R_f = 0.42; $[\alpha]_D^{20}$ = +65.3 (c = 0.51 in $CHCl_3$); ^1H NMR (500 MHz, $CDCl_3$): δ 7.38–7.18 (m, 5H), 7.08–6.89 (m, 2H), 6.78 (tt, *J* = 8.8, 2.3 Hz, 1H), 5.20 (d, *J* = 4.4 Hz, 1H), 4.14 (q, *J* = 13.7 Hz, 2H), 3.53 (d, *J* = 4.4 Hz, 1H); ^{13}C NMR (125 MHz, $CDCl_3$): δ 200.15, 163.09 (dd, $^{1,3}J$(C-F) = 249.9, 12.5 Hz), 141.41 (t, $^{3,3}J$(C-C-C-F) = 9.0 Hz), 136.27, 128.88, 128.76, 127.66, 109.98 (dd, $^{2,4}J$(C-C-F) = 20.1, 6.4 Hz), 104.31 (t, $^{2,2}J$(C-C-F) = 25.3 Hz), 78.69, 33.50; ^{19}F NMR (470 MHz, $CDCl_3$): δ −108.54; IR (neat): ν 3452, 3084, 3062, 2923, 1680, 1622, 1597, 1495, 1454, 1314, 1243, 1120, 1069, 990, 859, 817, 763, 701, 661; HRMS (m/z, ESI): $[M+Na]^+$ cald. for $C_{15}H_{12}F_2O_2SNa$, 317.0424; found, 317.0418; The enantiomeric excess was determined by HPLC using a Chiralcel OD-H (n-hexane/i-PrOH = 98/2, flow rate: 1.0 mL/min, wave length = 220 nm), t_R = 31.3 min (major), t_R = 33.8 min (minor).

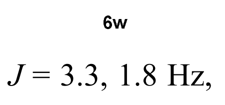

6w

New compound: Colorless liquid; TLC (EtOAc/n-hexane, 1/4 v/v): R_f = 0.23; $[\alpha]_D^{20}$ = +15.3 (c = 0.15 in $CHCl_3$); ^1H NMR (500 MHz, $CDCl_3$): δ 7.44–7.42 (m, 1H), 7.32–7.22 (m, 5H), 6.43 (d, *J* = 3.3 Hz, 1H), 6.38 (dd, *J* = 3.3, 1.8 Hz, 1H), 5.28 (d, *J* = 4.7 Hz, 1H), 4.19 (dd, *J* = 33.4, 13.7 Hz, 2H), 3.46 (d, *J* = 5.3 Hz, 1H); ^{13}C NMR (125 MHz, $CDCl_3$): δ 198.73, 149.98, 143.61, 136.52, 128.90, 128.70,

127.55, 110.74, 110.15, 73.36, 33.46; IR (neat): ν 3444, 3133, 3031, 2928, 1628, 1496, 1411, 1374, 1243, 1222, 1198, 1149, 1071, 1029, 1012, 978, 925, 808, 744, 702, 650, 637; HRMS (m/z, ESI): $[M+Na]^+$ cald. for $C_{13}H_{12}O_3SNa$, 271.0405; found, 271.0400; The enantiomeric excess was determined by HPLC using a Chiralpak AD-H (n-hexane/i-PrOH = 96/4, flow rate: 1.0 mL/min, wave length = 220 nm), t_R = 27.5 min (major), t_R = 35.0 min (minor).

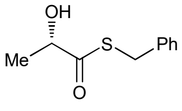

5x

Colorless liquid[9]; TLC (EtOAc/n-hexane, 1/4 v/v): R_f = 0.28; ^{1}H NMR (500 MHz, $CDCl_3$): δ 7.34–7.30 (m, 2H), 7.29–7.22 (m, 3H), 4.37 (quint, *J* = 6.5 Hz, 1H), 4.15 (s, 2H), 2.71 (d, *J* = 5.6 Hz, 1H), 1.45 (d, *J* = 6.9 Hz, 3H); ^{13}C NMR (125 MHz, $CDCl_3$): δ 203.63, 137.09, 129.00, 128.86, 128.70, 127.44, 73.94, 32.83, 21.12; IR (neat): ν 3732, 3623, 2931, 1683, 1496, 1455, 987, 734, 705, 669, 649; HRMS (m/z, ESI): $[M+Na]^+$ cald. for $C_{10}H_{12}O_2SNa$, 219.0456; found, 219.0449; The enantiomeric excess was determined by HPLC using a Regis (*R*,*R*) Whelk-O1 (n-hexane/i-PrOH = 99/1, flow rate: 0.7 mL/min, wave length = 220 nm), t_R = 28.3 min (major), t_R = 29.9 min (minor).

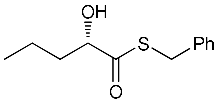

5y

New compound: Colorless liquid; TLC (EtOAc/n-hexane, 1/4 v/v): R_f = 0.38; $[\alpha]_D^{20}$ = −5.9 (c = 0.18 in $CHCl_3$); ^{1}H NMR (500 MHz, $CDCl_3$): δ 7.32–7.28 (m, 4H), 7.27–7.23 (m, 1H), 4.30–4.24 (m, 1H), 4.14 (d, *J* = 2.5 Hz, 2H), 2.72 (d, *J* = 5.0 Hz, 1H), 1.84–1.77 (m, 1H), 1.68–1.59 (m, 1H), 1.52–1.41 (m, 2H), 0.94 (t, *J* = 7.5 Hz, 3H); ^{13}C NMR (125 MHz, $CDCl_3$): δ 203.48, 137.17, 128.87, 128.69, 127.42, 77.45, 37.20, 32.78, 18.01, 13.77; IR (neat): ν 3482, 2958, 2935, 2872, 1663, 1494, 1467, 1454, 1321, 1140, 1107, 1071, 1030, 949, 775, 732, 709, 696, 633; HRMS (m/z, ESI): $[M+Na]^+$ cald. for $C_{12}H_{16}O_2SNa$, 247.0769; found, 247.0762; The enantiomeric excess was determined by HPLC using a Chiralcel OD-H (n-hexane/i-PrOH = 98/2, flow rate: 1.0 mL/min, wave length = 220 nm), t_R = 22.2 min (major), t_R = 25.1 min (minor).

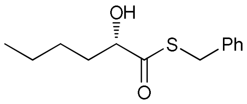

5z

New compound: Colorless liquid; TLC (EtOAc/n-hexane, 1/4 v/v): R_f = 0.48; $[\alpha]_D^{20}$ = −7.0 (c = 0.20 in $CHCl_3$); ^{1}H NMR (500 MHz, $CDCl_3$): δ 7.32–7.28 (m, 4H), 7.27–7.23 (m, 1H), 4.30–4.25 (m, 1H), 4.15 (d, *J* = 2.5 Hz, 2H), 1.90–1.81 (m, 1H), 1.70–1.61 (m, 1H), 1.45–1.25 (m, 4H), 0.89 (t, *J* = 7.0 Hz, 3H); ^{13}C NMR (125 MHz, $CDCl_3$): δ 203.39, 137.17, 128.87, 128.68, 127.42, 77.62, 34.89, 32.79, 26.73, 22.41, 13.91; IR (neat): ν 3468, 2953, 2935, 2858, 1661, 1494, 1454, 1399,

1308, 1272, 1141, 1151, 1071, 1028, 959, 774, 739, 710, 695, 635; HRMS (m/z, ESI): $[M+Na]^+$ cald. for $C_{13}H_{18}O_2SNa$, 261.0925; found, 261.0921; The enantiomeric excess was determined by HPLC using a Chiralcel OD-H (n-hexane/i-PrOH = 98/2, flow rate: 1.0 mL/min, wave length = 220 nm), t_R = 20.4 min (major), t_R = 23.0 min (minor).

General Procedure and Analytical Data for the BMS 270394 Intermediate 7

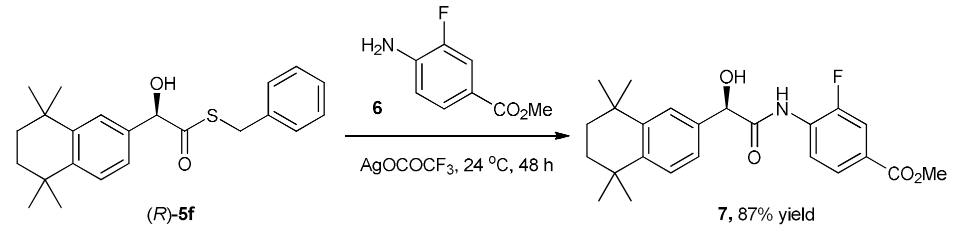

To a solution of (*R*)-**5f** (36.8 mg, 0.1 mmol) in toluene (0.25 mL), amine **6**[10] (25.3 mg, 0.15 mmol, 1.5 equiv.) and $AgOCOCF_3$ (33.1 mg, 0.15 mmol, 1.5 equiv.) were added at 24 °C. The reaction mixture was then stirred for 48 h. Solvent was evaporated in vacuo and the crude mixture was purified by flash column chromatography on silica gel (diethylether/n-hexane = 1/4) to give the corresponding (*R*)-α-hydroxyamide product **7** as white solid (36.1 mg, 87% yield)[11].

^{1}H NMR (500 MHz, $CDCl_3$): δ 8.74 (s, 1H), 8.47 (t, *J* = 8.2 Hz, 1H), 7.82 (d, *J* = 8.7 Hz, 1H), 7.78–7.72 (m, 1H), 7.40 (d, *J* = 1.9 Hz, 1H), 7.36–7.32 (m, 1H), 7.24–7.20 (m, 1H), 5.21 (d, *J* = 2.7 Hz, 1H), 3.90 (s, 3H), 3.13 (d, *J* = 3.0 Hz, 1H), 1.68 (s, 1H), 1.32–1.24 (m, 12H); ^{13}C NMR (125 MHz, $CDCl_3$): δ 170.38, 165.68, 151.66 (d, ^1J(C-F) = 244.5 Hz), 145.90 (d, ^3J(C-C-C-F) = 28.0 Hz), 135.39, 130.18 (d, ^2J(C-C-F) = 10.1 Hz), 127.52, 126.48 (d, ^4J(C-C-C-C-F) = 3.4 Hz), 126.14 (d, ^3J(C-C-C-F) = 7.1 Hz), 125.13, 123.53, 120.32, 116.00 (d, ^2J(C-C-F) = 21.0 Hz), 75.16, 52.31, 34.96, 34.89, 34.39, 34.24, 31.86, 31.81, 31.79, 31.75; ^{19}F NMR (470 MHz, $CDCl_3$): δ −130.98.

General Procedure and Analytical Data for the LY 411575 Intermediate 9

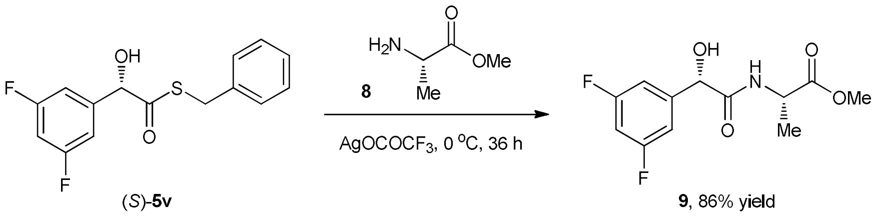

To a solution of (*S*)-**5v** (29.4 mg, 0.1 mmol) in toluene (0.25 mL), *L*-alanine methyl ester **8** (15.5 mg, 0.15 mmol, 1.5 equiv.) and $AgOCOCF_3$ (33.1 mg, 0.15 mmol, 1.5 equiv.) were added at 0 °C. The reaction mixture was then stirred for 12 h. Solvent was evaporated in vacuo and the crude mixture was purified by flash column chromatography on silica gel (acetone/n-hexane = 1/4) to obtain the desired (*S*)-α-hydroxyamide product **9** as a colorless oil (23.6 mg, 86% yield)[12].

^1H NMR (500 MHz, $CDCl_3$) / (8:1 diastereomers): δ 7.04–6.98 (m, 2H, major), 6.96–6.87 (m, 2H, minor), 6.87–6.75 (m, 1H, major), 6.73–6.66 (m, 1H, minor), 5.07 (d, *J* = 5.4 Hz, 1H), 4.57 (quint, *J* = 7.0 Hz, 1H, minor), 4.56 (quint, *J* = 7.0 Hz, 1H, major), 3.75 (s, 3H, minor), 3.74 (s, 3H, major), 1.42 (d, *J* = 7.2 Hz, 3H, major), 1.41 (d, *J* = 7.2 Hz, 3H, minor); ^{13}C NMR (125 MHz, $CDCl_3$): δ 173.12 (major), 172.86 (minor), 170.66 (major), 170.36 (minor), 163.17 (dd, *J* = 247.5, 5.0 Hz, major), 163.07 (dd. *J* = 247.5, 4.7 Hz, minor), 142.86 (t, *J* = 8.7 Hz, major), 142.69 (t, *J* = 8.7 Hz, minor), 109.67 (dd, *J* = 8.8, 26.1 Hz, major), 109.67 (dd, *J* = 21.2, 13.6 Hz, minor), 104.08 (t, *J* = 25.1 Hz, major), 103.92 (t, *J* = 25.1 Hz, minor), 73.17 (t, *J* = 1.8 Hz, major), 72.99 (t, *J* = 1.8 Hz, minor), 52.67 (major), 52.65 (minor), 48.15 (major), 48.10 (minor), 18.20 (major), 18.14 (minor); ^{19}F NMR (470 MHz, $CDCl_3$): δ −108.54 (minor), −108.74 (major).

General Procedure and Analytical Data for the LY 411575

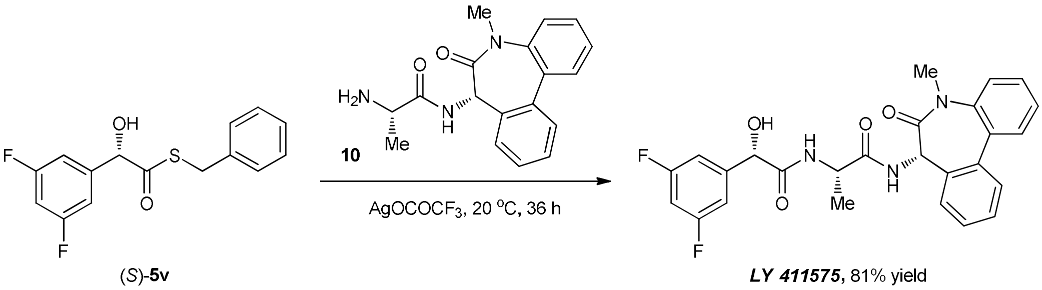

To a solution of (*S*)-**5v** (44.1 mg, 0.15 mmol, 1.5 equiv.) in CH_2Cl_2 (0.5 mL), lactam alanine amide **10**[13] (30.9 mg, 0.1 mmol, 1.0 equiv.) and $AgOCOCF_3$ (33.1 mg, 0.15 mmol, 1.5 equiv.) were added at 20 °C. The reaction mixture was then stirred for 36 h. Solvent was evaporated in vacuo and the crude mixture was purified by flash column chromatography on silica gel (EtOAc/n-hexane = 1/2) to obtain the desired LY 411575 as a white solid (38.6 mg, 81% yield)[14].

$[\alpha]_D^{20}$ = −72.5 (c = 0.53 in MeOH); ^{1}H NMR (500 MHz, $CDCl_3$): δ 7.87 (d, *J* = 6.7 Hz, 1H), 7.50 (d, *J* = 7.6 Hz, 2H), 7.48–7.42 (m, 1H), 7.41–7.30 (m, 4H), 7.30–7.23 (m, 1H), 6.89–6.83 (m, 2H), 6.69 (tt, *J* = 9.0, 2.5 Hz, 1H), 5.20 (d, *J* = 6.6 Hz, 1H), 5.07 (d, *J* = 4.5 Hz, 1H), 4.76 (d, *J* = 3.9 Hz, 1H), 4.64 (quint, *J* = 7.0 Hz, 1H), 3.33 (s, 3H), 1.36 (d, *J* = 7.0 Hz, 3Hz); ^{13}C NMR (125 MHz, $CDCl_3$): δ 172.21, 171.58, 168.96, 162.81 (dd, *J* = 248.6, 12.5 Hz), 143.34 (t, *J* = 8.8 Hz), 139.84, 135.95, 134.35, 133.24, 130.21, 128.96, 128.93, 128.56, 128.14, 126.33, 122.59, 121.99, 109.31 (dd, *J* = 20.1, 6.2 Hz), 103.28 (t, *J* = 25.3 Hz), 72.50 (t, *J* = 2.1 Hz), 53.37, 49.07, 36.51, 18.37; ^{19}F NMR (470 MHz, $CDCl_3$): δ −109.36.

Lit.[14] $[\alpha]_D^{20}$ = −121 (c = 1.0 in MeOH); ^{1}H NMR ($CDCl_3$): δ 7.67 (d, 1H), 7.60–7.28 (m, 8H), 7.15 (d, 1H), 6.98 (m, 2H), 6.74 (m, 1H), 5.21 (d, 1H), 4.94 (d, 1H), 4.61 (m, 1H), 4.56 (m, 1H), 3.34 (s, 3H), 1.42 (d, 3H).

Analytical data for (*S*)-Lactam-*L*-Boc-alanine amide

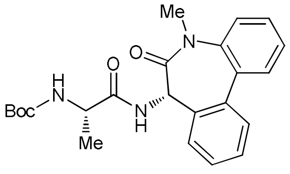

White solid; $[\alpha]_D^{20}$ = −73.6 (c = 1.0 in MeOH); ^{1}H NMR (500 MHz, $CDCl_3$): δ 7.75 (d, *J* = 5.5 Hz, 1H), 7.62–7.58 (m, 1H), 7.56–7.52 (m, 1H), 7.48–7.43 (m, 1H), 7.42–7.32 (m, 5H), 5.29 (d, *J* = 6.5 Hz, 1H), 5.22–5.16 (m, 1H), 4.42–4.32 (m, 1H), 3.35 (s, 3H), 1.49 (s, 9H), 1.39 (d, *J* = 7.0 Hz, 3H); ^{13}C NMR (125 MHz, $CDCl_3$): δ 172.14, 169.25, 155.49, 140.10, 136.71, 134.60, 133.41, 130.36, 128.85, 128.81, 128.48, 127.94, 126.16, 122.64, 122.23, 80.01, 53.07, 50.36, 36.46, 28.38, 18.48.

Lit.[15] $[\alpha]_D^{20}$ = −96 (c = 1.0 in MeOH; for (*S*)-Lactam-*L*-alanine amide); ^{1}H NMR ($CDCl_3$): δ 7.62–7.33 (m, 9H), 5.26 (d, 1H), 5.08 (m, 1H), 4.34 (m, 1H), 3.35 (s, 3H), 1.49 (s, 9H), 1.40 (d, 3H).

Supplementary References

1. Jagt. D. L. V. & Han, L. -P. B. Deuterium isotope effects and chemically modified coenzymes as mechanism probes of yeast glyoxalase I. *Biochemistry* **12**, 5161–5167.
2. Yan, H. *et al.* Scalable organocatalytic asymmetric Strecker reactions catalysed by a chiral cyanide generator. *Nat. Commun.* 3:1212 doi: 10.1038ncomms/2216 (2012).
3. Park, S. Y., Lee, J. -W. & Song, C. E. Parts-per-million level loading organocatalyzed enantioselective silylation of alcohols. *Nat. Commun.* 6:7512 doi: 10.1038/ncomms8512 (2015).
4. Riley, H. A. & Gray, A. R. Phenylglyoxal. *Org. Synth.* **15**, 67 (1935).
5. Lokhande, P. D., Waghmare, S. R., Gaikwad, H. & Hankare, P. P. Oxidation of aryl and heeroaryl methyl ketone to aryl and heteroarylglyoxals by using $CuCl_2$-DMSO. *Indian J. Chem.* **52B**, 300–305 (2013).
6. Ispolnov, K., Gomes, R. A., Silva, M. S. & Freire, A. P. Extracellular methylglyoxal toxicity in Saccharomyces cerevisiae: role of glucose and phosphate ions. *J. Appl. Microbiol.* **140**, 1092–1102 (2008).
7. Mamillapalli, N. C. & Sekar, G. Enantioselective synthesis of α-hydroxy amides and β-amino alcohols from α-keto amides. *Chem. Eur. J.* **21**, 18584–18588 (2015).
8. Chen, C. -T. *et al.* Asymmetric aerobic oxidation of α-hydroxy acid derivatives by C_4-symmetric, vanadate-centered, tetrakisvanadyl(V) clusters derived from *N*-salicylidene-

α-aminocarboxylates. *J. Org. Chem.* **72**, 8175–8185 (2007).

9. Hall, S. S., Doweyko, L. M., Doweyko, A. M. & Zilenovski, J. S. R. Synthesis and evaluation of α-hydroxythiol esters as antitumor agents and Glyoxalase I inhibitors. *J. Med. Chem.* **20**, 1239–1242 (1977).
10. Kazmierski, W. M. *et al.* Preparation of benimidazolylazabicyclooctylethylpiperidines as Ccr5 antagonists for the treatment of HIV infection. PCT Int. Appl. CODEN:PIXXD2; WO2004054974 (2004).
11. Patel, R. N., Chu, L., Chidambaram, R., Zhu, J. & Kant, J. Enantioselective microbial reduction of 2-oxo-2-(1',2',3',4'-tetrahydro-1',1',4',4'-tetramethyl-6'-naphthalenyl) acetic acid and its ethyl ester. *Tetrahedron: Asymmetry* **13**, 349–355 (2002).
12. Leighty, M. W., Shen, B. & Johnston, J. N. Enantioselective synthesis of α-oxy amides via umpolung amide synthesis. *J. Am. Chem. Soc.* **134**, 15233–15236 (2012).
13. Fauq, A. H., Simpson, K., Maharvi, G. M., Golde, T. & Das, P. A multigram chemical synthesis of the γ-secretase inhibitor LY411575 and its diastereoisomers. *Bio. Med. Chem. Lett.* **17**, 6392–6395 (2007).
14. Jing, Wu *et al.* Preparation of cycloalkyl, lactam, lactone and related compounds for inhibiting β–amyloid peptide release and/or its synthesis. PCT Int. Appl. WO2004054974 (1998).
15. James, E *et al.* Polycyclic α–amino-ε-caprolactams and related compounds. PCT Int. Appl. WO1999032453 A1 (1998).
